# Integrative analysis of semaphorins family genes in colorectal cancer: implications for prognosis and immunotherapy

**DOI:** 10.3389/fimmu.2025.1536545

**Published:** 2025-03-04

**Authors:** Jiahao Zhu, Benjie Xu, Zhixing Wu, Zhiwei Yu, Shengjun Ji, Jie Lian, Haibo Lu

**Affiliations:** ^1^ Department of Outpatient Chemotherapy, Harbin Medical University Cancer Hospital, Harbin, Heilongjiang, China; ^2^ Department of Computer Science, University of Liverpool, Liverpool, United Kingdom; ^3^ Department of Biological Sciences, Xi’an Jiaotong-Liverpool University, Suzhou, Jiangsu, China; ^4^ Department of Colorectal Surgery, Harbin Medical University Cancer Hospital, Harbin, Heilongjiang, China; ^5^ Department of Radiotherapy and Oncology, Suzhou Municipal Hospital, The Affiliated Suzhou Hospital of Nanjing Medical University, Gusu School, Nanjing Medical University, Suzhou, Jiangsu, China

**Keywords:** semaphorins, colorectal cancer, immunotherapy, single-cell sequencing, spatial transcriptome sequencing

## Abstract

**Background:**

Semaphorins (SEMAs), originally identified as axon guidance factors, have been found to play crucial roles in tumor growth, invasiveness, neoangiogenesis, and the modulation of immune responses. However, the prognostic value of SEMA-related genes in colorectal cancer (CRC) remains unclear.

**Methods:**

We applied a novel machine learning framework that incorporated 10 machine learning algorithms and their 101 combinations to construct a SEMAs-related score (SRS). Multi-omics analysis was performed, including single-cell RNA sequencing (scRNA-seq), and spatial transcriptome (ST) to gain a more comprehensive understanding of the SRS. A series of cell experiments were conducted to prove the impact of key genes on CRC biological behavior.

**Result:**

A consensus SRS was finally constructed based on a 101-combination machine learning computational framework, demonstrating outstanding performance in predicting overall survival. Moreover, distinct biological functions, mutation burden, immune cell infiltration, and immunotherapy response were observed between the high- and low-SRS groups. scRNA-seq and ST demonstrated unique cellular heterogeneity in CRC. We observed that SRS-high and SRS-low malignant epithelial cells exhibit different biological characteristics. High SRS malignant epithelial cells interact with myeloid and endothelial cells via SPP1 and COL4A2-ITGAV-ITGB8 pathways, respectively. Low SRS cells engage with myeloid and endothelial cells through MIF and JAG1-NOTCH4 pathways. Additionally, knocking down SEMA4C significantly inhibits the proliferation and invasion of CRC cells, while promoting apoptosis *in vitro*.

**Conclusion:**

SRS could serve as an effective tool to predict survival and identify potential patients benefiting from immunotherapy in CRC. It also reveals tumor heterogeneity and provides valuable biological insights in CRC.

## Introduction

Colorectal cancer (CRC) is the third most common cancer globally, with 1.93 million cases reported in 2020. Additionally, it ranks as the second leading cause of global cancer-related deaths, accounting for around 916,000 fatalities that year (1). Despite reduced mortality rates due to screening, about 25% of patients are diagnosed with metastatic disease at initial diagnosis (2). Despite advancements in CRC patient survival through combined surgery, radiotherapy, and chemotherapy, challenges persist with disease recurrence and low survival rates. Recent advancements in immunotherapy, particularly with immune checkpoint inhibitors (ICIs), have demonstrated promising outcomes in the treatment of various cancers (3, 4). ICIs have been integrated into the standard treatment regimen for CRC, but only a limited subset of patients have experienced substantial and lasting benefits. Hence, it is crucial to discover biomarkers or signatures that could reliably predict treatment efficacy in CRC patients.

Semaphorins (SEMAs), initially identified as axon guidance factors, are membrane-bound or secreted proteins involved in cell communication (5). Growing evidence indicates that SEMAs expression is dysregulated in various cancers, contributing to angiogenesis (6), metastasis (7), and immune escape (8). They affect tumor progression by altering immune interactions between tumor cells and infiltrating immune cells within the tumor microenvironment (TME). This study focuses on SEMA3 to SEMA7, the semaphorins expressed in humans. Recent studies indicate that SEMAs may function as attractants, influencing the recruitment of immune cells such as macrophages, natural killer cells, dendritic cells, and cytotoxic T lymphocytes to the TME (9). For instance, Sema3A, Sema4C, and Sema4D promote tumor progression by attracting tumor-associated macrophages (10). Sema4A, found on dendritic and B cells, facilitates T cell priming and differentiation of T helper cells in CD4+ T cells (11). It is associated with increased CD8+ T cell activation, playing a key role in IL-33-mediated anti-tumor immune responses (12).

This study utilized 10 machine learning algorithms to develop a consensus SEMAs-related score (SRS) for the 20-member SEMAs family in CRC. We also analyzed the differences in biological functions, mutation burden, immune cell infiltration, and immunotherapy response between the SRS-high and SRS-low groups. Single-cell RNA sequencing (scRNA-seq) and spatial transcriptomics (ST) analyses were performed to examine cellular heterogeneity in CRC tissue and to investigate the unique biological characteristics of SRS-high and SRS-low malignant epithelial cells. This study highlights the regulatory potential of SEMAs, providing a theoretical basis for future targeted and immunotherapeutic approaches.

## Materials and methods

### Overview design of the study

**Figure 1:**
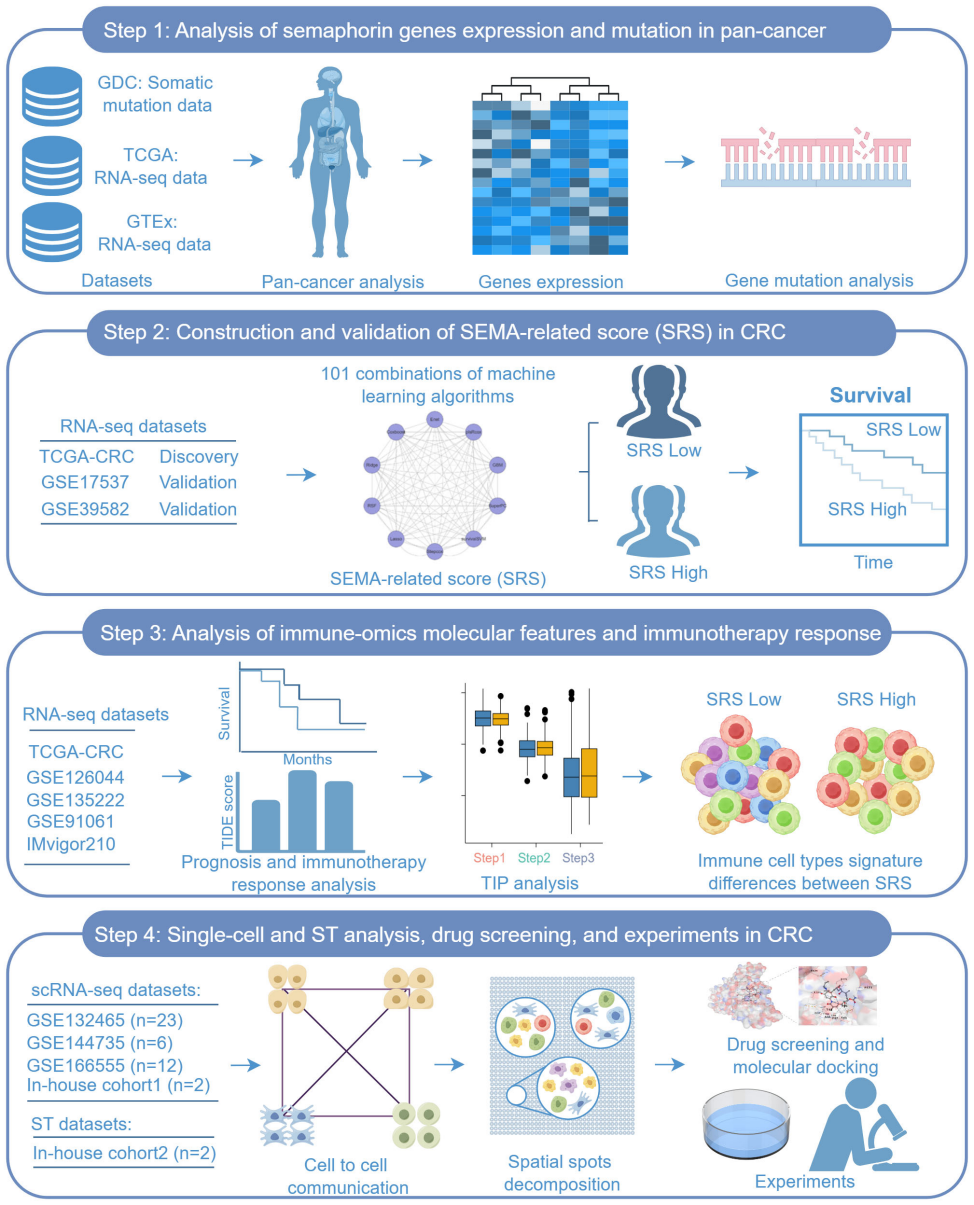
Workflow of the study.

**Figure 1** shows the workflow of this study.

### Landscape of SEMA family expression and mutagenesis in pan-cancer

Transcriptome data for 27 solid tumors were sourced from The Cancer Genome Atlas (TCGA) using the R package “TCGAbiolinks” (13). **Supplementary Table S1** listed the 27 solid tumors used in this study. Transcriptome data from normal tissues were obtained from the Genotype-Tissue Expression (GTEx) project. The somatic mutation profiles from Genomic Data Commons (GDC) were compiled into a mutation annotation format (MAF) file. The trinucleotide W (TCW, W = A or T) mutation and tumor mutation burden (TMB) analysis were used the R package “maftools” (14).

### Prognostic signature construction based on integrative machine learning in CRC

Twenty SEMA genes were selected to develop the prognostic signature. To ensure high accuracy and generalizability of the SRS, we employed an integration of 10 machine learning algorithms (**Supplementary Table S2**). The TCGA-CRC cohort, integrating TCGA-COAD and TCGA-READ datasets, was utilized for initial model training, with GSE17537 (15) and GSE39582 (16) cohorts employed for validation. To improve comparability across different cohorts, we applied Z-score normalization to all data in advance.

The development pipeline for the SRS involved constructing models using 101 combinations of these 10 machine learning algorithms to identify the optimal predictive SRS with the best concordance index (C-index) (17). The TCGA-CRC cohort served as the training set for model development. After establishing the models on the training set, they were tested on the validation cohorts (GSE17537 and GSE39582). The model with the highest average C-index, calculated across the validation datasets, was deemed optimal.

### Comprehensive analysis of immune-omics molecular characterization and immunotherapy response based on SRS

The Immuno-Oncology Biological Research (IOBR) R package was utilized to gather diverse signatures associated with TME cell types, immunotherapy responses, immune suppression, and immune exclusion (18). We computed the enrichment score for each sample to thoroughly examine the immunological differences between patients with high and low SRS. We compared the distribution of tumor mutational burden (TMB) and tumor neoantigen burden (TNB) across the two groups. We utilized the tracking tumor immunophenotype (TIP) and tumor immune dysfunction and exclusion (TIDE) algorithms to explore biological mechanisms linked to SRS and assess the response to immunotherapy (19, 20). Predictive value of SRS was further verified in GSE126044 (21), GSE135222 (22), GSE91061 (23) and IMvigor210 cohort (24).

### scRNA-seq data collecting and pre-processing

To enhance the comprehensiveness of our study, we integrated our previous scRNA-seq cohort (In-house cohort1), which includes two rectal cancer tissue samples (25), with publicly available scRNA-seq datasets (GSE132465, GSE144735, and GSE166555) (26, 27). A total of 43 tumor tissue samples were included. We utilized Seurat to import the count data and create a Seurat object. After scaling the data as previously described (28), we selected 2000 highly variable genes and merged all single-cell data. Principal component analysis (PCA) was conducted using 50 principal components. To address batch effects present in both our study and public data, we applied the Harmony package. The merged scRNA-seq data underwent clustering analysis at a resolution of 0.2, and was visualized using UMAP with 50 principal components. Clusters containing fewer than 100 cells were excluded to ensure robust results.

### Celltypes annotation and copy number variation inferring

For the first step, cells were primarily annotated according to marker genes (29), including epithelial cells (EPCAM, KRT8, KRT19), endothelial cells (VWF, COL1A2, CLDN5), myeloid cells (CD68, CD14, C1QA), T/NK cells (CD3D, CD3E, CD7), B cells (MS4A1, CD79A, BANK1), plasma cells (JCHAIN, IGHA2, MZB1), fibroblast cells (COL1A1, COL3A1, DCN), mast cells (CPA3, MS4A2, TPSAB1). In the second step, we used the “FindNeighbors” and “FindClusters” functions in Seurat to identify cell subtypes. Specific cell types in CRC were defined using gene signatures and known lineage markers as auxiliary tools. To identify malignant cells from epithelial cells, the CopyKAT algorithm (version 0.1.0) (30) was employed to estimate CNVs. Aneuploid epithelial cells are considered malignant cells, whereas diploid epithelial cells are regarded as normal cells.

### scRNA-seq data scores calculating

We applied five distinct algorithms, including “AddModuleScore” “AUCell” “ssGSEA” “singscore” and “UCell”, to calculate the SRS of malignant epithelial cells. The AddModuleScore (31) algorithm calculates enrichment scores by averaging the expression values of genes in a set, providing insights into biological processes. The ssGSEA algorithm evaluates the enrichment levels of specific gene sets within individual samples or cells. AUCell assesses gene set enrichment in scRNA-seq data by ranking gene expression and calculating the area under the curve. UCell (32) enables unsupervised cell type identification without the need for prior labels. SingScore quantifies the activity of biological functions or processes within individual cells, aiding in the assessment of cell states. By integrating the scores from these five algorithms, we derived a comprehensive score. Cells with a comprehensive score greater than 1 were classified as SRS high malignant tumor cells, whereas those with a score of 1 or less were categorized as SRS low malignant tumor cells.

### Cell communication analysis

To infer cell-to-cell interactions, we employed two tools: “CellChat” (33) and “CellCall” (34). The CellChat R package employs an extensive signaling interaction database that includes various receptor-ligand interactions, such as multimeric complexes, soluble agonists and antagonists, and both stimulatory and inhibitory membrane-bound coreceptors. This enables detailed mapping of the cellular communication landscape. Conversely, CellCall aggregates ligand-receptor-transcription factor axis datasets using KEGG pathway information. By combining intracellular with intercellular signals, CellCall elucidates specific pathways involved in cellular interactions. A comprehensive understanding of specific cellular communication pathways was attained through CellCall, thereby enhancing the broader interaction network identified through CellChat.

### ST data processing

In this study, we utilized the 10× spatial transcriptomic sequencing method to capture spatial data from two intestinal cancer tissue samples (In-house cohort2). Detailed information on these samples can be found in our previous study (25). To process the ST data, we followed a series of steps. First, data standardization was performed using the “SCTransform” function within the “Seurat” package. Next, dimensionality reduction was achieved by applying the “RunPCA” function to conduct PCA, which reduces the dimensionality of the data and highlights the most significant variations. Following PCA, the “RunUMAP” function was employed to carry out UMAP for clustering the data.

### Re−localization for ST data

The RCTD method was employed to assign cell types from the reference scRNA-seq dataset to spatial transcriptomic data (35). Cell type-specific marker genes were identified using the ‘FindAllMarkers’ function in Seurat. This step ensured the accurate identification of cell type-specific markers. Following this, the standard RCTD analysis protocol was meticulously followed, applying it to both the reference scRNA-seq data and the Visium spatial transcriptomics data.

### ST heteromorphic cell spatial network analysis

Heterotypic score algorithm was used according to the previous study (36). The kNN function in the dbscan package was used to calculate the distances between spots containing SRS high malignant tumor cells and spots containing myeloid or endothelial cells. Specifically, if all six neighboring spots around a SRS high malignant tumor cell contained myeloid or endothelial cells, these spots were selected for further analysis. The threshold for a spot to be considered as containing tumor cells was set at 0.2, while the threshold for myeloid or endothelial cells was set at 0.1. This analysis uncovered numerous associations between specific cell types, providing insight into the intricate cellular interactions within the tissue microenvironment.

### Spatial map of cell dependencies

MISTy is an interpretable multi-view machine learning framework designed to analyze highly multiplexed intercellular relationships within data and thereby facilitate the understanding of cell interaction patterns and mechanisms (37). Cell-type RCTD estimates were modeled using three spatial contexts: (1) intrinsic correlations within a locale, (2) juxta estimations for nearby neighbors (maximum distance of 5), and (3) para estimations for distant neighbors (radius of 15 spots). The median standardized importance of each context were interpreted as cell-type dependencies, indicating colocalization or mutual exclusion, without implying causation.

### Spatial signaling analysis

The “COMMOT” package (38) was employed to analyze signaling directions within spatial transcriptome data. COMMOT, short for COMMunication analysis by Optimal Transport, was designed to infer cell-cell communication by concurrently evaluating multiple ligand-receptor pairs. This package facilitates the summarization and comparison of spatial signaling directions and identifies the downstream impacts of cell-cell communication on gene expression through ensemble of trees models.

### Potential therapy agents for patients with high SRS

Gene Set Enrichment Analysis (GSEA) (39) was employed to compare oncogenic pathway activation between high- and low-SRS patients. Drug sensitivity data for cancer cell lines were obtained using the Cancer Therapeutics Response Portal (CTRP) and Profiling Relative Inhibition Simultaneously in Mixtures (PRISM). The AUC was utilized to assess drug sensitivity. Potential drugs for high-SRS patients were explored by referencing to previous studies (40), and the area under the AUC were used as a indicators for evaluating drug sensitivity. Agents with lower AUC in the high-SRS group (logFC > 0.2) and those with negative correlation coefficients (r < -0.15) were selected as the final potential therapeutic agents. Additionally, molecular docking was also performed. Protein structures were obtained from the Protein Data Bank, and small molecule information was retrieved from PubChem. Molecular docking was carried out using the online tool CB-Dock2.

### Cell culture and siRNA transfections

HT29 and SW480 cells, obtained from ATCC, were maintained in Dulbecco’s Modified Eagle Medium (Gibco, USA) with 10% fetal bovine serum (CellMax, China) and 1% penicillin-streptomycin. The cells were maintained at 37°C in a 5% CO2 environment, with routine testing to ensure they were free of mycoplasma contamination. Lipofectamine 3000 (Invitrogen, USA) was used for siRNA transfection according to the manufacturer’s guidelines. The siRNA, sourced from Ribio, China, is detailed in **Supplementary Table S3** with the specific sequences used in the study.

### Western blotting

Total proteins were extracted using radio immunoprecipitation assay lysis (RIPA) buffer supplemented with phenylmethylsulfonyl fluoride (PMSF) and a Protease Inhibitor Cocktail (APEXBIO, USA). Protein concentrations were measured using a BCA kit from Beyotime, China. Protein lysates (30 µg) were separated on a 10% SDS-PAGE gel and transferred to polyvinylidene fluoride (PVDF) membranes. Membranes were blocked using 5% skim milk in TBST and incubated with primary antibodies against SEMA4C (1:3000, Cat. no: 28402-1-AP; Proteintech) and β-actin (1:1000, Cat. no: TA09; OriGene Technologies, Inc.). Following the washing step, membranes were treated with HRP-conjugated secondary antibodies (1:10,000, Cat. no: 92632210/92632211; LI-COR Biosciences). Protein bands were detected with an ECL kit (Beyotime, China).

### CCK-8 assay and colony formation assays

To evaluate cell proliferation, CRC cells transfected with either sh-NC or si-SEMA4C lentivirus were seeded into 96-well plates.Cell proliferation was assessed with the Cell Counting Kit-8 (CCK-8, Beyotime, China) following the manufacturer’s guidelines. Optical density (OD) readings at 450 nm were recorded to determine the cell proliferation rate.

Transfected CRC cell lines were plated at 1,000 cells per well in 6-well plates and cultured in RPMI-1640 medium supplemented with 10% fetal bovine serum for 10 days to facilitate colony formation. Colonies were then fixed with methanol and stained with 1% crystal violet (Beyotime, China). Stained colonies were imaged for analysis.

### Transwell assay

Cell migration was evaluated using a Transwell assay.Cells (1×10^5^ cells/mL) were suspended in serum-free DMEM and seeded into the upper chamber of an 8-µm pore insert (Corning, NY, USA), while the lower chamber contained DMEM with 10% FBS. After 24 hours of incubation, cotton swabs were used to remove non-migrated cells, and migrated cells were imaged at 200× magnification in five random fields. Migration was measured as the average cell count per field, expressed with the standard deviation. For the invasion assay, the Transwell membrane was pre-coated with 1 mg/mL Matrigel (BD Biosciences, NJ, USA) before following the same protocol as the migration assay.

### Cell apoptosis assay

Cell apoptosis was evaluated with an Annexin V-FITC/PI Detection Kit. Four hours after transfection, cells were exposed to 50 μmol/L 6-OHDA and incubated for 24 hours at 37°C with 5% CO2. Following incubation, cells were harvested, rinsed twice with sterile PBS, and labeled with Annexin V-FITC/PI. Apoptosis levels were then analyzed by flow cytometry.

### Statistical analysis

Unpaired Student’s t-test was used for normally distributed variables, and the Wilcoxon rank-sum test was applied for non-normally distributed variables when comparing two groups. For datasets with more than two groups, one-way ANOVA was employed for parametric data, and the Kruskal-Wallis test was utilized for non-parametric data. Two-sided Fisher’s exact test was used to analyze contingency tables. The SRS threshold value was set based on the mean. Statistical analyses were conducted using R (v4.2.1), GraphPad Prism (v9.3.1), and Python (v3.8). (ns: P > 0.05, *: P < 0.05, **: P < 0.01, ***: P < 0.001, ****: P < 0.0001).

## Results

### Landscape of SEMA family expression and SEMA mutagenesis in pan-cancer

**Figure 2:**
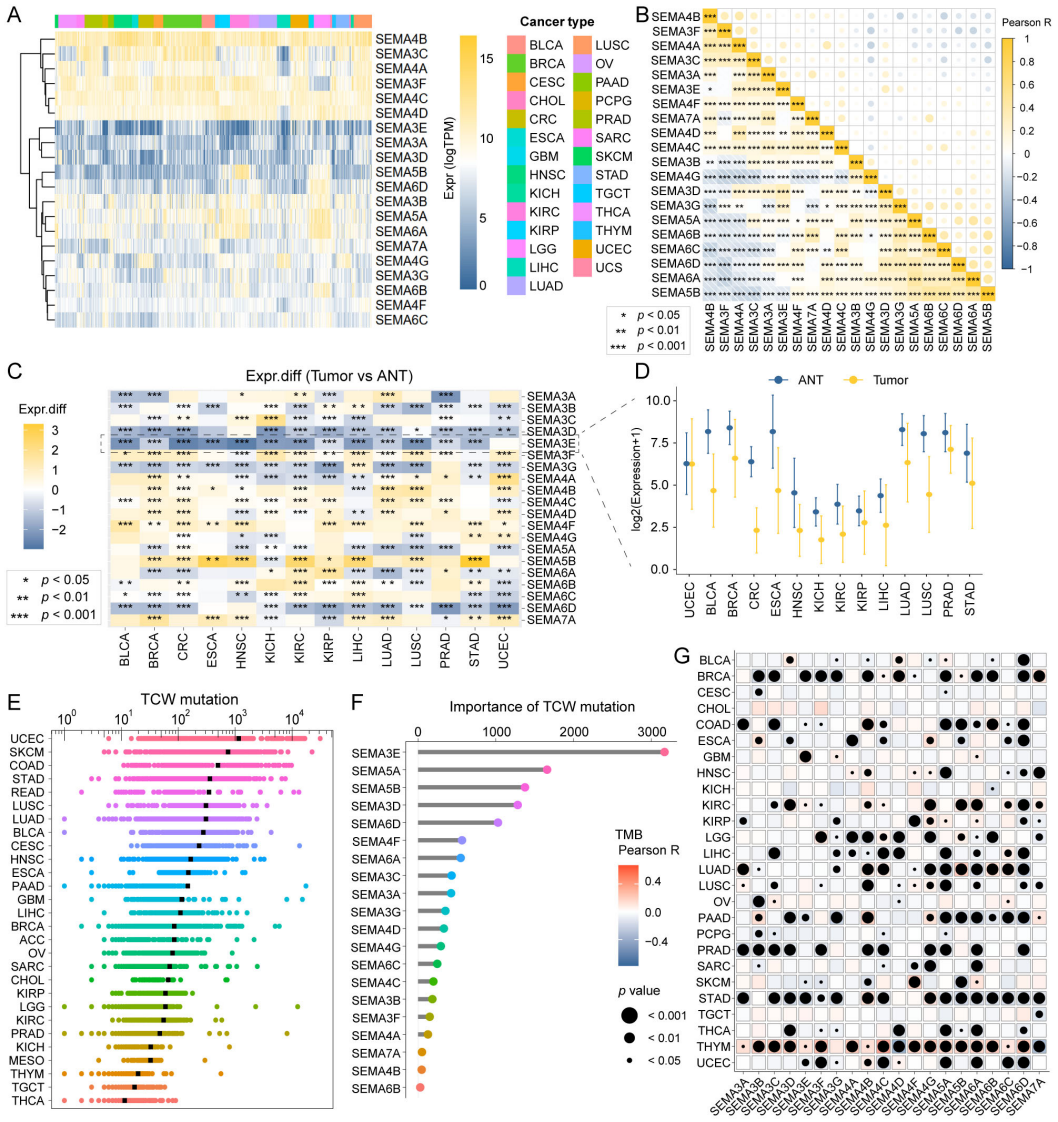
Landscape of SEMAs family expression and mutagenesis in pan-cancer. **(A)** A comprehensive heatmap illustrates the expression pattern of SEMAs across solid cancer types from TCGA. **(B)** The Pearson correlation analysis was performed among all 20 SEMAs family members in pan-cancer. **(C)** All 20 SEMAs family members were compared in primary tumors versus adjacent normal tissues (ANT). **(D)** SEMA3E was significantly downregulated in 13 of 14 cancer types compared with paracancerous tissue. **(E)** TCW mutation count in pan-cancer. **(F)** SEMA3E emerged as the most important contributor to TCW mutation in pan-cancer. **(G)** The correlations among TMB and SEMAs were analyzed in pan-cancer. All SEMAs, except SEMA3G, had significant correlations with TMB in THYM.

To investigate the genomic characteristics of the 20 SEMA family genes across multiple cancers, we generated a comprehensive heatmap. The heatmap illustrated expression profiles across 9,765 samples, comprising 9,398 primary tumors and 367 metastatic tumors, spanning 27 solid cancer types from TCGA (**Figure 2A**). In the pan-cancer analysis, SEMA4B showed the highest expression levels among all the SEMAs. Additionally, certain SEMAs showed notably high expression in specific cancer types, such as SEMA5B in KIRC. A Pearson correlation analysis of all 20 SEMA family members showed that most SEMAs were significantly positively correlated (**Figure 2B**). We also assessed the expression of the 20 SEMA family members by comparing their levels in primary tumors and adjacent normal tissues (ANTs) across 14 cancer types. **Figure 2C** illustrates significant dysregulation of SEMAs in these cancer types relative to their corresponding ANTs. SEMA3E was notably downregulated in 13 cancer types, except for uterine corpus endometrial carcinoma (UCEC), where no significant downregulation was observed. The detailed expression of SEMA3E in the 14 cancer types and their corresponding ANTs is exhibited in **Figure 2D**.

We calculated the TCW mutation count for each tumor sample across all cancer types, considering changes from TCW to TTW and TCW to TGW, where W represents A or T.UCEC, skin cutaneous melanoma, and colon cancer exhibited the highest TCW mutation counts (**Figure 2E**). Using a random forest algorithm, we identified SEMA3E as the most significant contributor to TCW mutations in pan-cancer analysis (**Figure 2F**). Additionally, we evaluated the associations between TMB and SEMA family members across different cancer types. The comprehensive heatmap (**Figure 2G**) illustrates these correlations, where the color intensity reflecting the magnitude of the correlation and point size indicating the level of statistical significance. Notably, we observed that all SEMAs, except SEMA3G, had significant correlations with TMB in thymoma.

### SRS signature predicts prognosis and immunotherapy response in CRC

**Figure 3:**
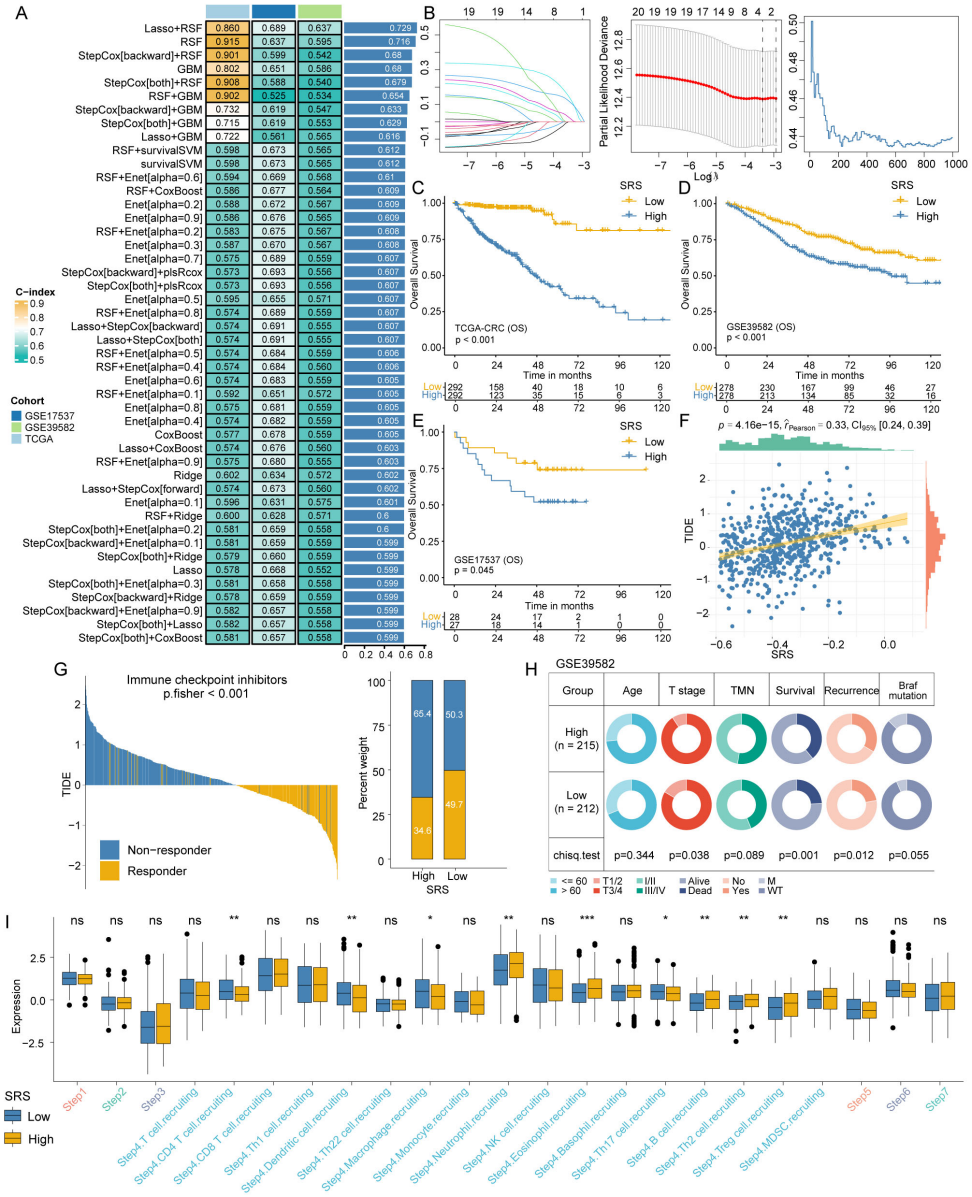
SRS construction and differences analysis between SRS-high and SRS-low groups. **(A)** A combination of 101 machine learning algorithms was generated through a comprehensive computational framework. The C-index of each model was calculated form the TCGA-CRC, GSE17537, and GSE39582 cohorts and sorted by the average C-index. **(B)** Determination of the best λ when the penalized likelihood deviation reached its minimum value in the TCGA-CRC training cohort (left); error rate in the random survival forest as a function of the classification tree (middle); and calculation of the out-of-bag importance values for the predicted genes (right). **(C–E)** Kaplan-Meier survival curves for SRS in the TCGA-CRC training cohort and the GSE17537 and GSE39582 validation cohorts, illustrating overall survival differences. **(F)** A significant positive association exists between SRS and the TIDE score. **(G)** The TIDE algorithm predicts response to immunotherapy between SRS-high and SRS-low groups. **(H)** Pie charts showing the chi-squared test results between SRS and clinical characteristics in CRC patients from the GSE39582 cohort. **(I)** Differences in the degree of activation between SRS-high and SRS-low groups at each step of TIP. *: P < 0.05, **: P < 0.01, ***: P < 0.001. ns: not significant.

All 20 SEMA family members were used to develop a SRS using a machine learning-based technique within a Leave-One-Out Cross-Validation framework to fit 101 models. The TCGA-CRC cohort was utilized as the training dataset, with the GSE39582 and GSE17537 datasets employed for validation. The optimal model, which integrated Lasso regression and RSF, achieved an average C-index of 0.729 (**Figure 3A**). To determine the optimal model parameters, we selected the λ value that minimized the penalized likelihood deviation in the TCGA-CRC training cohort (**Figure 3B**, left). For the RSF component, the error rate was assessed as a function of the classification tree (**Figure 3B**, middle), and out-of-bag importance values were used to evaluate gene contributions (**Figure 3B**, right). The SRS for each patient was determined by applying the regression coefficient-weighted expression of SEMA3E, SEMA4C, and SEMA6C within the RSF model. Patients in the training and validation cohorts were divided into SRS-high and SRS-low groups based on the median SRS value. It is noteworthy that the overall survival (OS) of patients with high SRS levels was significantly lower compared to those with low SRS levels (all P-values < 0.05, **Figures 3C–E**).

The TIDE algorithm assessed patient responses to immunotherapy. The analysis revealed that SRS was also positively associated with the TIDE score (**Figure 3F**) and patients in the low SRS group exhibited significantly better responsiveness to immunotherapy in comparison to those in the high SRS group (P < 0.001; **Figure 3G** for Fisher’s exact test results). Chi-square tests showed that the SRS-low group had a lower proportion of patients with III/IV T stage (P = 0.038), lower mortality (P = 0.001), and lower recurrence (P = 0.012, **Figure 3H**).

We calculated the TIP to explore potential biological mechanisms associated with the SRS. The SRS-low group exhibited significant differences primarily at step 4, which involves the recruitment of tumor immune-infiltrating cells (**Figure 3I**). The SRS-low group exhibited significantly higher proportions of T cells, dendritic cells, macrophages, and Th17 cells, whereas the SRS-high group showed significantly higher proportions of neutrophils, eosinophils, B cells, Th2 cells, and regulatory T cells (Tregs).

### The validation of SRS predictive power for immunotherapy response

**Figure 4:**
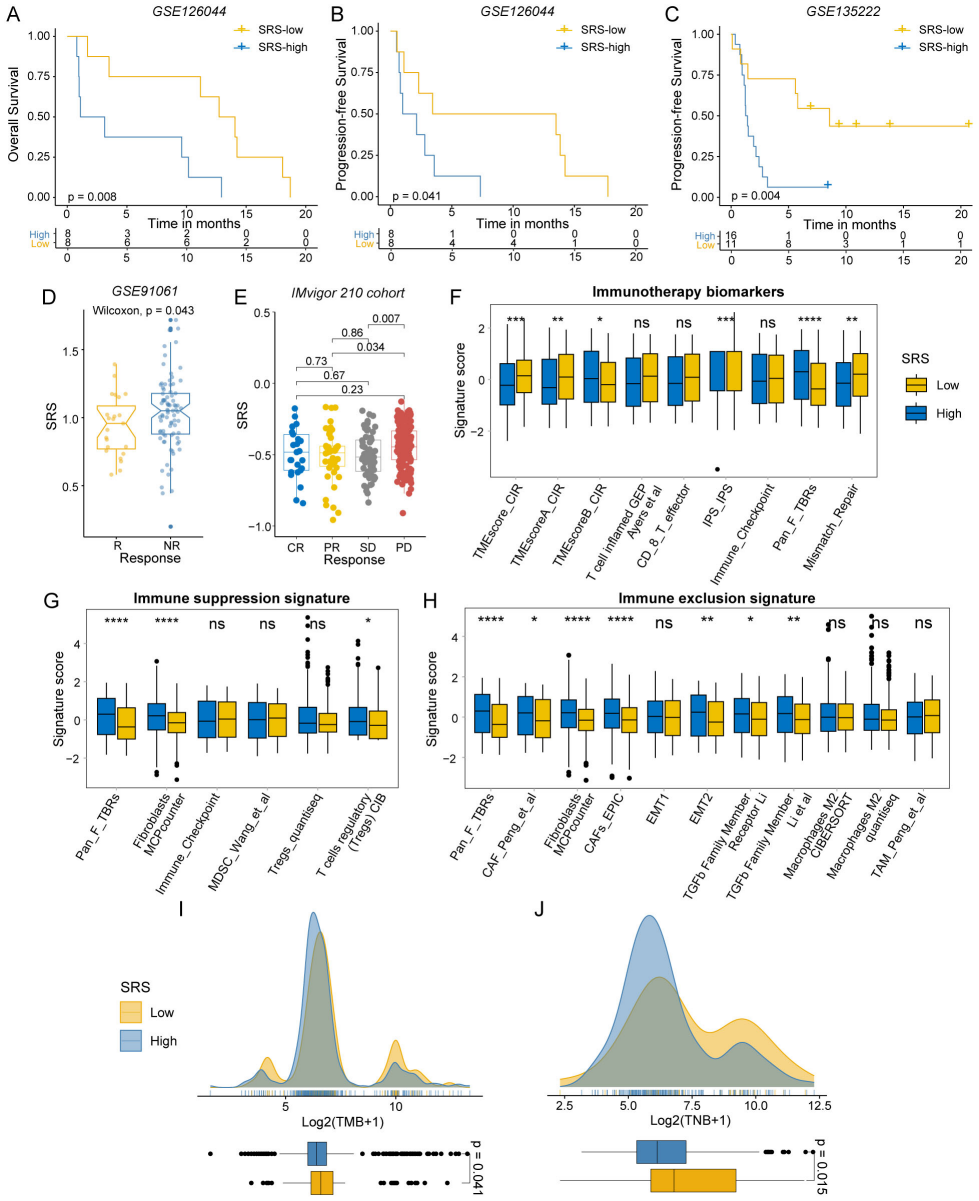
Immunotherapy response differences between SRS-high and SRS-low groups. **(A, B)** Survival analysis of SRS-high and SRS-low groups in GSE126044. **(C)** Survival analysis of SRS-high and SRS-low groups in GSE135222. **(D)** Distribution of SRS in different immunotherapy response groups of GSE91061. **(E)** Distribution of SRS in different immunotherapy response groups of IMvigor 210. **(F)** The distribution of immunotherapy biomarkers between SRS-high and SRS-low groups. **(G)** The distribution of immune suppression signatures between SRS-high and SRS-low groups. **(H)** The distribution of immune exclusion signatures SRS-high and SRS-low groups. **(I)** The distribution of TMB between SRS-high and SRS-low groups. **(J)** The distribution of TNB between SRS-high and SRS-low groups. (ns: P > 0.05, *: P < 0.05, **: P < 0.01, ***: P < 0.001, ****: P < 0.0001).

We further substantiated our conclusions by examining multiple immunotherapy validation cohorts. A lower SRS was linked to better OS and progression-free survival (PFS), with statistical significance observed in GSE126044 for OS (P = 0.008; **Figure 4A**) and PFS (P = 0.041; **Figure 4B**), and in GSE135222 for PFS (P = 0.004; **Figure 4C**). Furthermore, a lower SRS was generally correlated with a better response to immunotherapy (GSE91061, P = 0.043; **Figure 4D**). Post-immunotherapy patients with progressive disease had higher SRS compared to those with partial response (P = 0.034) and stable disease (P = 0.007) in IMvigor 210 cohort (**Figure 4E**).

### Immune characteristics related to SRS

We utilized the IOBR R package to examine the tumor microenvironment in colorectal cancer. As expected, signatures linked to favorable immunotherapy outcomes were significantly enriched in the SRS-low group (**Figure 4F**). CRCs with low SRS levels were more likely to exhibit characteristics of hot tumors with heightened immune responses. In contrast, fibroblasts and Tregs were predominantly enriched in the SRS-high group. The SRS-high group exhibited enrichment of immunosuppressive and immune-exclusion markers, including the epithelial-mesenchymal transition (EMT) pathway, suggesting an immunosuppressive state (**Figures 4G, H**). These findings suggest that SRS-high CRCs are more likely to be cold tumors, which typically respond poorly to immunotherapy.

TMB and TNB are well-recognized biomarkers for assessing patient responses to immunotherapy. To investigate the differences in these biomarkers between the SRS-high and SRS-low groups, we conducted a comprehensive analysis. Our findings revealed that the SRS-low group demonstrated significantly elevated levels of TMB (P = 0.041) and TNB (P = 0.015), indicating that this group may possess greater immunogenicity (**Figures 4I, J**).

### Heterogeneity of malignant tumor epithelial cells in CRC

**Figure 5:**
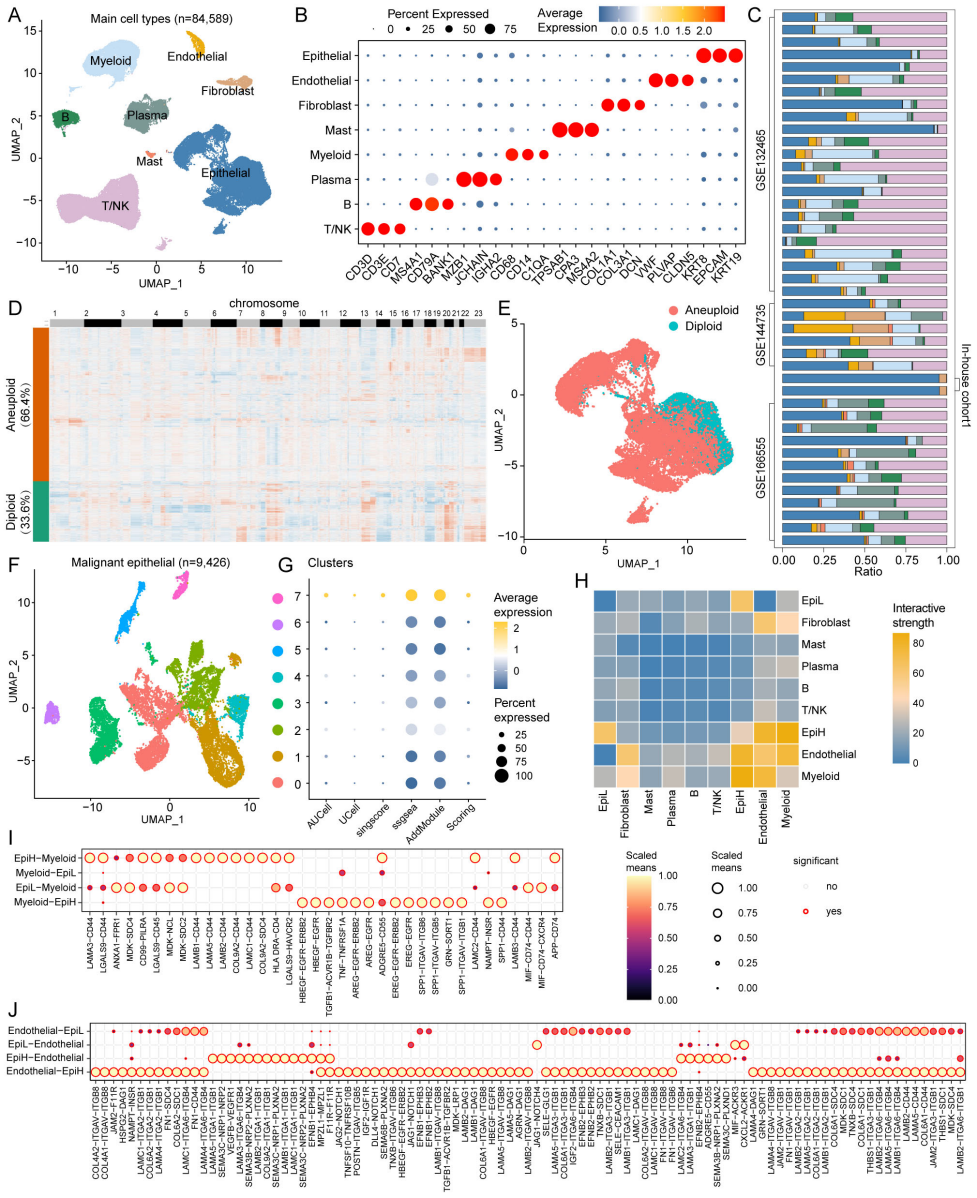
Single-cell RNA sequencing analysis in CRC. **(A)** UMAP plots of 84,589 cells from tumor tissues of 43 CRC patients in the 4 cohorts (GSE132465, GSE144735, GSE166555, and In-house cohort1), showing 8 major cell types. **(B)** Dot plots displaying marker genes for each major cell type in the 4 cohorts. **(C)** Proportional distribution of the nine major cell types across the 43 samples. **(D)** Heatmap of inferred copy number variations in epithelial cells with CopyKAT algorithm, showing aneuploid (66.4%) and diploid (33.6%) epithelial cells. **(E)** UMAP plots of aneuploid and diploid epithelial cells. **(F)** UMAP plots of Reclustered 9,426 malignant epithelial cells, showing 8 cell types. **(G)** Bubble plots demonstrating the enrichment scores of SRS for each reclustered malignant epithelial cell types. **(H)** Interactive strength of cell-to-cell interactions among the 9 major cell types. **(I)** Potential ligand-receptor interactions between malignant epithelial cells and myeloid cells. **(J)** Potential ligand-receptor interactions between malignant epithelial cells and endothelial cells.

scRNA-seq data from CRC tissues were extracted from four datasets comprising 43 CRC patients and 84,589 cells (**Figure 5A**). Major cell clusters were annotated using marker genes, identifying T/NK cells, B cells, plasma cells, myeloid cells, mast cells, fibroblasts, endothelial cells, and epithelial cells (**Figure 5B**). The proportional composition of these nine cell types across these samples is shown in **Figure 5C**.

Malignant tumor epithelial cells were identified by using the CopyKAT algorithm based on CNV. Among the 14,195 epithelial cells, 9,426 (66.4%) were predicted to be aneuploid, while the remaining 4,769 (33.6%) cells were predicted to be diploid (**Figures 5D, E**). We re-clustered 9,426 malignant epithelial cells to differentiate those with high and low SRS. Eight clusters of malignant epithelial cells were identified, with clusters 7 classified as SRS high cluster (EpiH) and the remaining clusters as SRS low cluster (EpiL) (**Figures 5F, G**). Cell-to-cell interaction analyses were conducted to investigate ligand-receptor interactions between malignant epithelial cells with high or low SRS and other cell types. Results from Cellchat demonstrated varying interactive strengths among the 10 cell subtypes, with the greatest interactions between EpiH and myeloid cells, followed by endothelial cells (**Figure 5H**).

Then, cell-to-cell communication between malignant epithelial cells with different SRS and myeloid cells or endothelial cells was analyzed. The results demonstrate significant ligand-receptor interactions among different cell types (**Figures 5I, J**). In the interactions between EpiH and myeloid cells, SPP1 signaling pathways and several ligands such as LAMB1-CD44 and LAMA5-CD44, among others, show notable signaling capabilities, indicating that EpiH may play a crucial role in regulating myeloid cell functions. Similarly, the communication between EpiL and myeloid cells also exhibits significant interactions, particularly through MIF signaling pathways. Regarding the endothelial cell analysis, EpiH demonstrates strong signaling interactions with endothelial cells, especially with the COL4A2-ITGAV-ITGB8 and LAMA5-ITGA1-ITGB1 ligand-receptor pairs. The interactions between EpiL and endothelial cells also show significance, especially with the JAG1-NOTCH4 ligand-receptor pairs.

### Cell-to-cell communication between malignant epithelial cells and subtypes of myeloid or endothelial cells

**Figure 6:**
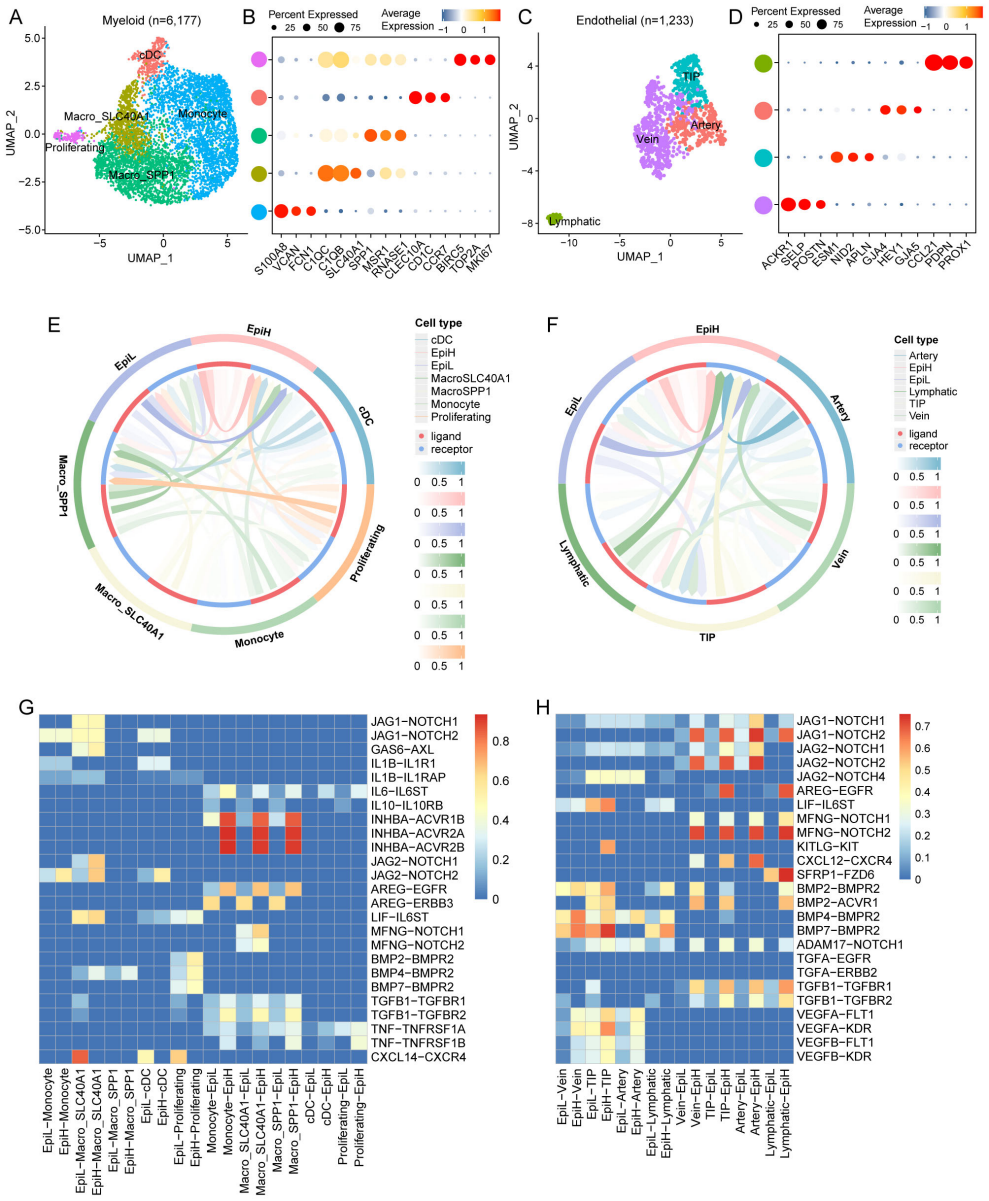
Communication between malignant epithelial cells and myeloid and endothelial cell subtypes. **(A)** UMAP plots of 6,177 myeloid cells from tumor tissues of 43 CRC patients, showing 5 cell subtypes. **(B)** Dot plots of marker genes for each myeloid cell subtypes. **(C)** UMAP plots of 1,233 endothelial cells from tumor tissues of 43 CRC patients, showing 4 cell subtypes. **(D)** Dot plots of marker genes for each endothelial cell subtypes. **(E)** Circle diagram illustrating the strength of ligand-receptor interactions among malignant epithelial and myeloid cell subtypes. **(F)** Circle diagram illustrating the strength of ligand-receptor interactions among malignant epithelial and endothelial cell subtypes. **(G)** Identification of highly ranked ligand-receptor pairs and their associated transcription factors among malignant epithelial and myeloid cell subtypes. **(H)** Identification of highly ranked ligand-receptor pairs and their associated transcription factors among malignant epithelial and endothelial cell subtypes.

In this study, myeloid cells were re-clustered into five distinct subtypes in accordance with a previous study (41): monocytes, conventional DCs (cDCs), SPP1-positive macrophages (Macro_SPP1), SLC40A1-positive macrophages (Macro_SLC40A1), and proliferating cells (**Figures 6A, B**; **Supplementary Table S4**). Endothelial cells were re-clustered into four subtypes—tip cells, vein endothelial cells, artery endothelial cells, and lymphatic endothelial cells—according to another study (42) (**Figures 6C, D**; **Supplementary Table S5**). The CellCall analysis demonstrated significant interactions between malignant epithelial cells with different SRS and the various subtypes of myeloid and endothelial cells (**Figures 6E, F**).

Notably, the interaction between EpiL and monocytes displays a strong signaling potential, particularly through the JAG1-NOTCH1 and JAG2-NOTCH2 pathways. These interactions suggest that EpiL may actively engage with monocytes, influencing their differentiation and function. Moreover, the presence of AREG-EGFR and AREG-ERBB3 interactions indicates potential regulatory mechanisms in epithelial-myeloid communication. The identified ligand-receptor pairs, such as INHBA-ACVR1B and INHBA-ACVR2A/B, further emphasize the functional cross-talk between epithelial and myeloid cells, potentially affecting cellular proliferation and signaling cascades. The heatmap highlights significant interactions between IL-10 and TNF pathways, indicating a balance of pro-inflammatory and anti-inflammatory signals between epithelial and myeloid cells (**Figure 6G**).

Noteworthy interactions include the JAG1-NOTCH1 and JAG2-NOTCH pathways, which are prominently involved in mediating communication between EpiH and various endothelial cell types. These interactions suggest a role for EpiH in modulating endothelial cell fate and responsiveness. Additionally, the presence of AREG-EGFR and LIF-IL6ST interactions indicates potential pathways by which EpiL may regulate endothelial cell activities, including survival and proliferation. The involvement of BMP and TGFA signaling—particularly BMP2-BMPR2 and TGFA-EGFR—further highlights the importance of these pathways in endothelial function and tissue repair processes. Furthermore, the heatmap reveals interactions involving chemokines, such as CXCL12-CXCR4, which may be critical for endothelial cell migration and recruitment in response to inflammatory stimuli (**Figure 6H**).

### Cell spatial network analysis and spatial signaling analysis with ST

**Figure 7:**
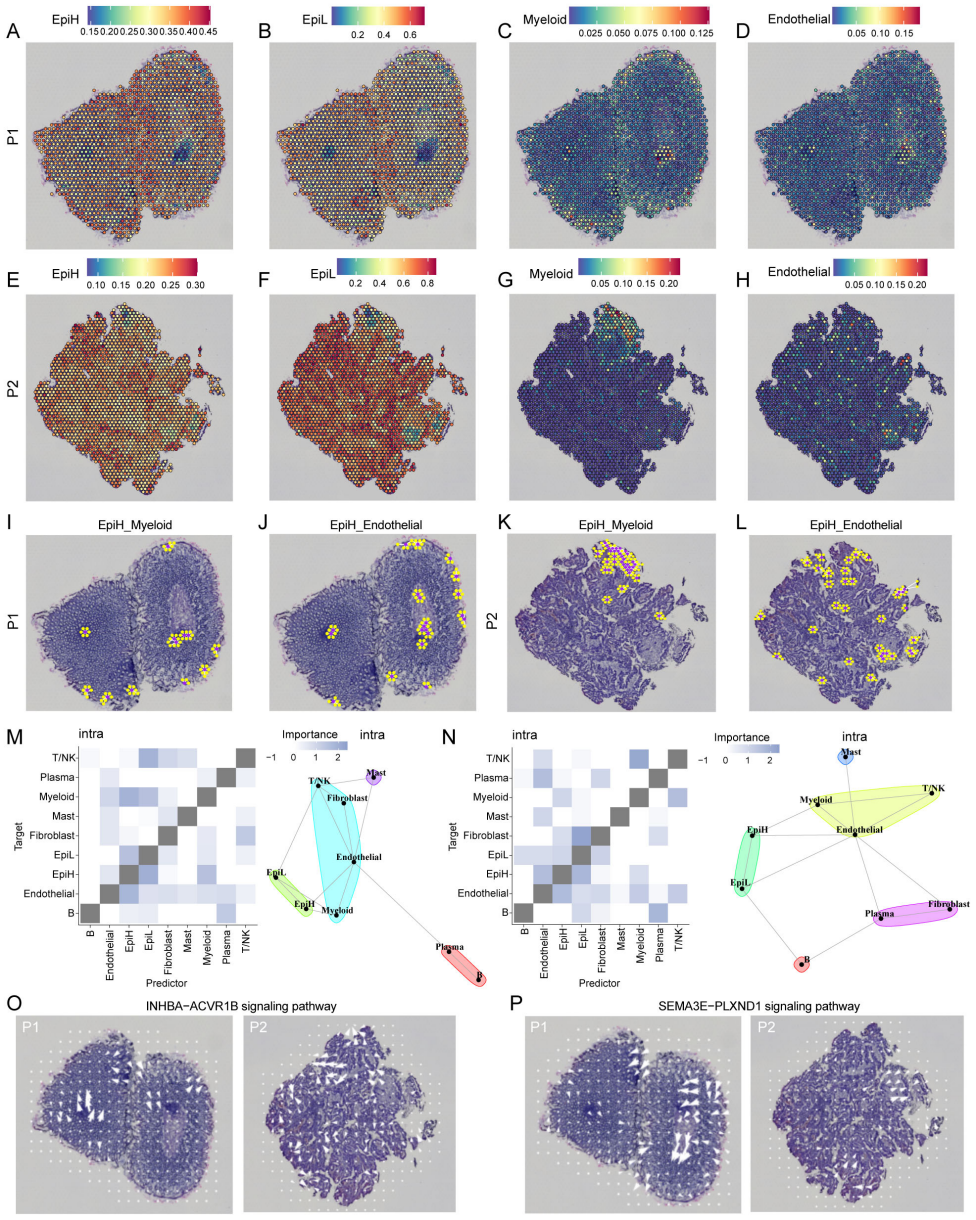
ST analysis of malignant epithelial, myeloid and endothelial cells in two CRC examples. **(A–H)** Co-localization of malignant epithelial cells with SRS-high (EpiH), SRS-low (EpiH), myeloid and endothelial cells. **(I–L)** The spatial network analysis of EpiH in relation to myeloid and endothelial cells. Purple dots represent spots containing EpiH and yellow dots denote the presence of myeloid or endothelial cells. **(M, N)** Extrapolation of spatial clustering and projection of spatial correlations among different cell types based on the MISTy algorithm. **(O)** Spatial signaling directionality analysis of the INHBA-ACVR1B pathways. **(P)** Spatial signaling directionality analysis of the SEMA3E-PLXND1 pathways.

To further assess the spatial organization of EpiH, EpiL, myeloid, and endothelial cells, we performed ST analysis on tumor tissue sections from our previous ST dataset, which includes two intestinal cancer tissue examples (25). We utilized the RCTD methodology to deconvolute annotated cell types from scRNA-seq data and integrate them with ST data. This approach enabled us to infer the predominant cell types present at each spatial location (**Figures 7A–H**).

The spatial network analysis of EpiH in relation to myeloid and endothelial cells is illustrated in the **Figures 7I–L**, where purple dots represent spots containing EpiH and yellow dots denote the presence of myeloid or endothelial cells. In the EpiH_Myeloid panel, the clustering of yellow spots in proximity to EpiH regions suggests potential spatial interactions that may facilitate communication and functional modulation between these cell types. This spatial arrangement likely reflects the dynamic role of EpiH in influencing myeloid cell behavior, including recruitment and activation within the local microenvironment. Similarly, the EpiH_Endothelial panel reveals a comparable pattern of endothelial cells adjacent to EpiH spots, with the presence of yellow dots near EpiH indicating potential interactions critical for angiogenesis, tissue repair, and overall vascular integrity. Based on the results from MISTy, epithelial cells with high SRS demonstrated a congruence in clustering patterns and a heightened correlation in spatial interactions with myeloid and endothelial cells within the internal space (**Figures 7M, N**). Considering the significance of the INHBA-ACVR1B and SEMA3E-PLXND1 signaling pathways in the interaction between EpiH and myeloid or endothelial cells, COMMOT software was utilized to infer the spatial signaling directionality of these two pathways. **Figure 7O** illustrates the INHBA-ACVR1B signaling pathway, and **Figure 7P** depicts the SEMA3E-PLXND1 signaling pathway.

### Potential therapeutic drugs screening

**Figure 8:**
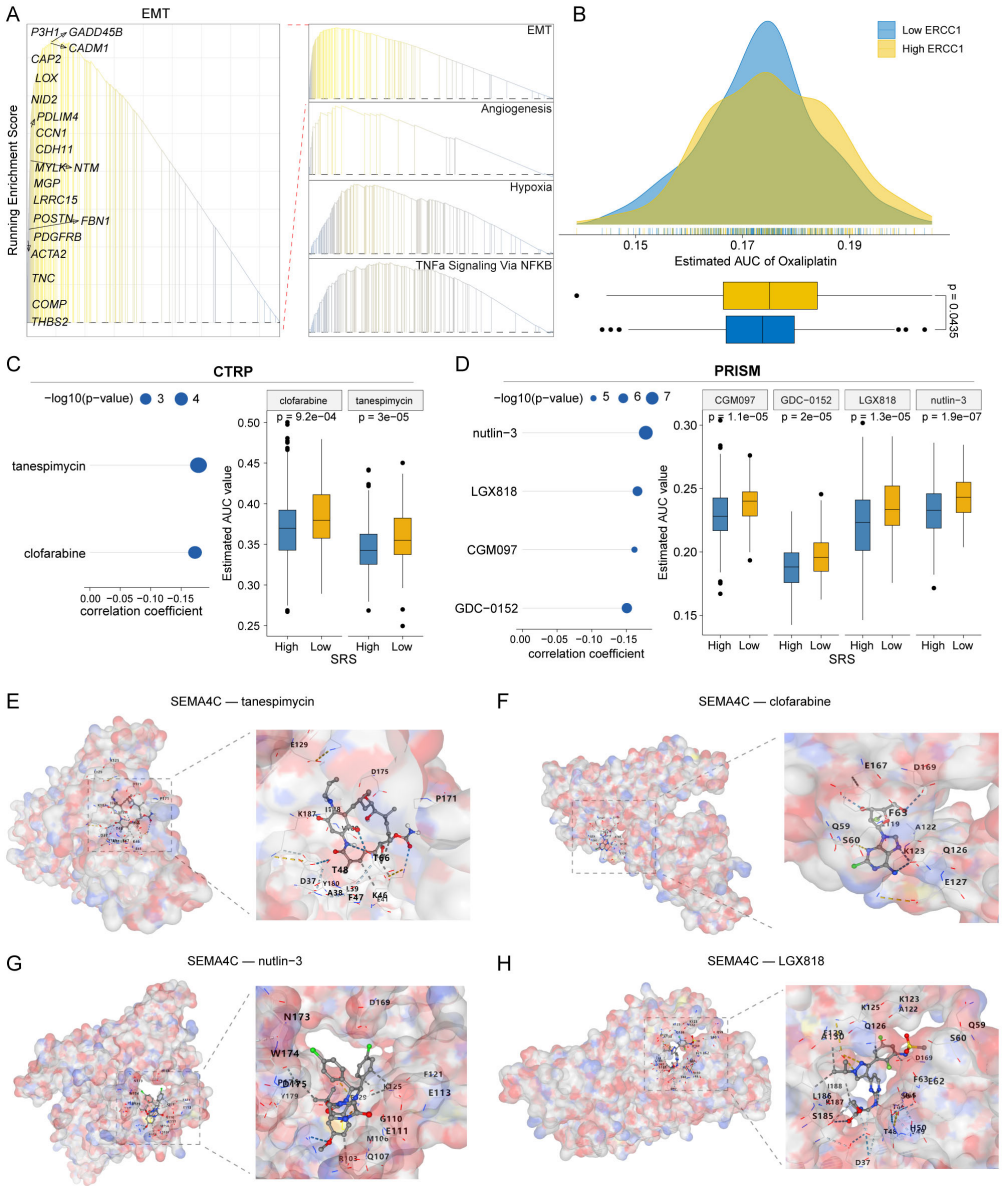
Potential agents for patients with high SRS. **(A)** Identification of significantly activated pathways in the SRS-high group using the GSEA algorithm. **(B)** Predicting oxaliplatin sensitivity to assess the computational algorithm’s feasibility. **(C, D)** Analyzing correlation and differences in drug sensitivity for potential candidates from the CTRP and PRISM datasets. **(E–H)** Conducting molecular docking studies between SEMA4C and the compounds tanespimycin, clofarabine, nutlin-3, and LGX818.

Notable prognostic disparities were observed between populations characterized by high and low SRS levels. GSEA indicated that pathways related to EMT, angiogenesis, hypoxia, and TNF-alpha signaling via the NF-kB pathway were markedly activated in SRS-high patients (**Figure 8A**). Due to the inadequate immunotherapy response in patients with high SRS, we utilized the CTRP and PRISM platforms to identify potential therapeutic drugs for these individuals. We employed Oxaliplatin, a standard CRC treatment, as a benchmark to confirm the robustness of our methodology. Our algorithm revealed that patients exhibiting low ERCC1 expression levels responded more effectively to cisplatin therapy, corroborating established clinical findings and indicating possible advantages for chemotherapy (**Figure 8B**). Finally, we screened two CTRP-derived agent (tanespimycin and clofarabine; **Figure 8C**) and four PRISM-derived agents (nutlin-3, LGX818, CGM097, and GDC-0152; **Figure 8D**). We further explored the molecular docking interactions of the target gene SEMA4C with tanespimycin, clofarabine, nutlin-3, and LGX818. The simulations revealed that the binding energy of SEMA4C with tanespimycin was -8.0 kcal/mol, with clofarabine was -6.5 kcal/mol, with nutlin-3 was -6.3 kcal/mol, and with LGX818 was -6.6 kcal/mol (**Figures 8E–H**). These results indicate that their potential as therapeutic agents for targeting SEMA4C.

### Cell function experiments

**Figure 9:**
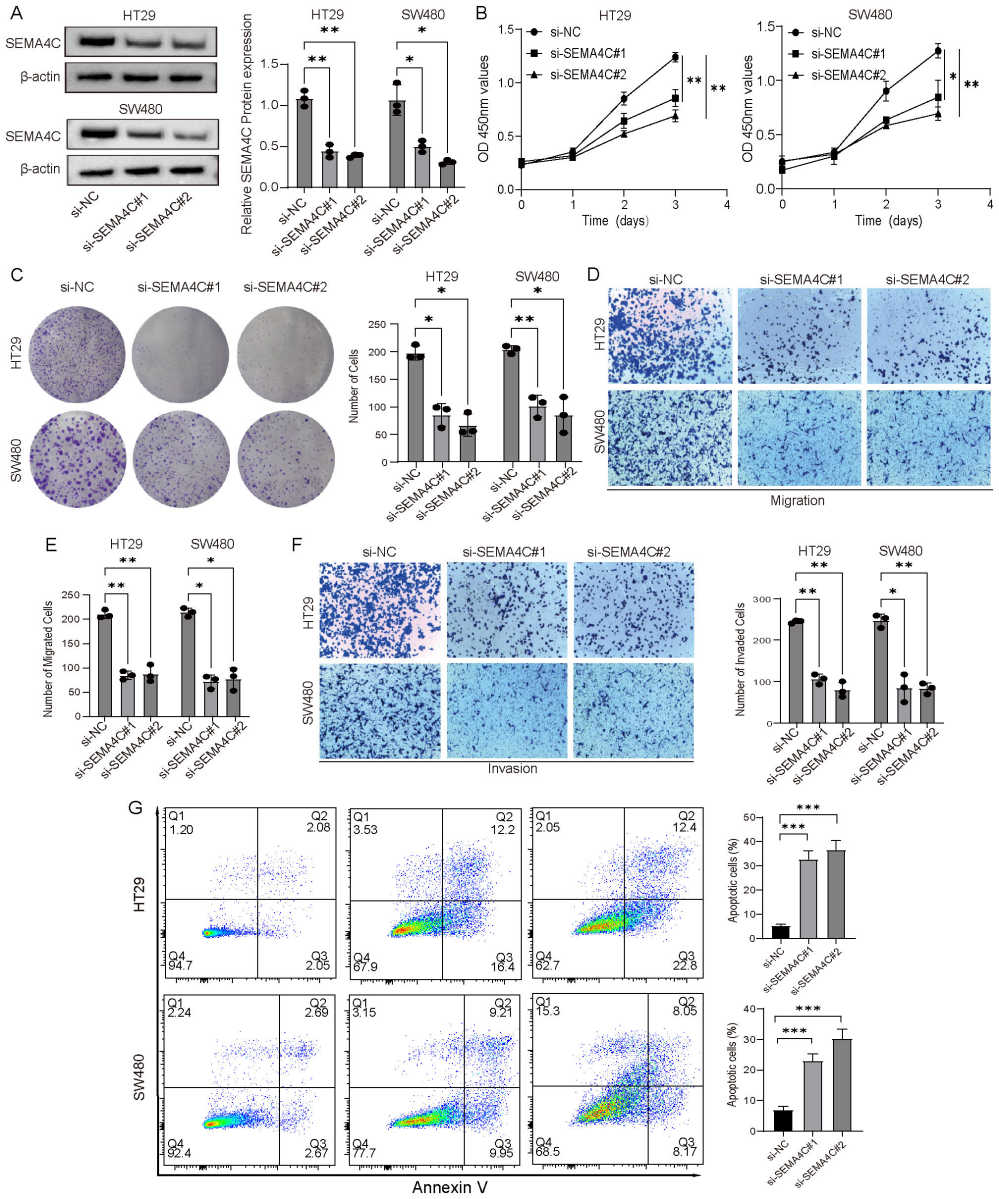
Verification of the tumor-promoting effect of SEMA4C in CRC. **(A)** Western blot analysis of SEMA4C expression in HT29 and SW480 cell lines. **(B, C)** CCK-8 and colony formation assays were conducted to evaluate the impact of SEMA4C downregulation on the proliferation and viability of HT29 and SW480 cell lines. **(D–F)** Transwell assays were performed to evaluate the influence of SEMA4C downregulation on the cell migration and invasion. **(G)** Impact of SEMA4C downregulation on the cell apoptosis was analyzed by flow cytometry assay. (*: P < 0.05; **: P < 0.01; ***: P < 0.001).

Drug screening analysis revealed that SEMA4C could be targeted by multiple drugs, whereas no potential intervention drugs were identified for the other two genes. Additionally, research on the biological role of SEMA4C in colorectal cancer remains limited. Given this, SEMA4C was selected for further investigation to assess its function in CRC progression at the cellular level. To demonstrate the tumor-promoting role of SEMA4C in CRC, we developed knockdown vectors for SEMAC4 (si-SEMA4C#1, si-SEMA4C#2) alongside RNAi negative control vectors (si-NC) and transfected them into HT29 and SW480 cell lines. Western blot analysis confirmed the successful transfection and a significant reduction in SEMAC4 expression in both cell lines when using si-SEMA4C#1 and si-SEMA4C#2 (**Figure 9A**). CCK-8 and colony formation assays demonstrated significantly reduced cell viability and proliferation in the knockdown groups relative to the controls (**Figures 9B, C**). Transwell assays demonstrated a marked decrease in the migration and invasion abilities of HT29 and SW480 cells in the knockdown groups compared to the control group (**Figures 9D–F**). Additionally, flow cytometry results indicated an increase in cell apoptosis in the knockdown groups (**Figure 9G**). These findings suggest that SEMA4C contributes to CRC cell growth and migration, highlighting its potential role as a tumor promoter.

## Discussion

This study presents a novel computational framework that incorporates ten unique machine learning algorithms and their 101 potential combinations. This extensive analysis led to the identification of the SRS, which exhibits high predictive accuracy for the prognosis of colorectal cancer CRC. Moreover, we utilized the SRS for risk stratification of CRC patients, evaluating their response to immunotherapy. Our findings suggest that CRC patients exhibiting a low SRS demonstrate enhanced survival rates and exhibit a greater likelihood of benefiting from immunotherapy. These insights offer a rational framework for the administration of immunotherapy in clinical practice, marking a substantial advancement toward more effective personalized medicine strategies. Furthermore, we identified potential pharmacological agents that may inhibit the progression of CRC to a high SRS phenotype, thereby presenting novel opportunities for CRC preventive strategies. In contrast to previous research, which primarily focused on the prognostic implications of the signature, this study incorporated bulk transcriptome analysis, scRNA-seq, and ST to attain a more comprehensive understanding of the SRS. These findings provide robust biological evidence and explanations for the SRS, emphasizing its potential in guiding personalized medicine approaches.

Our study utilized an innovative computational framework to identify a strong prognostic signature, SRS. Kaplan-Meier curve analysis demonstrated that the SRS effectively stratifies CRC patients’ risk concerning OS. Additionally, SRS is associated with more advanced CRC stages and metastasis occurrence, linked to adverse clinical outcomes. Approximately 30% of patients diagnosed with locally advanced CRC eventually progress to metastatic disease, underscoring the critical need for early predictive measures to enhance prognosis and therapeutic outcomes (43). Notably, our study revealed that the SRS predicts not only prognosis but also the occurrence and progression of CRC. It demonstrated outstanding diagnostic performance across various datasets, affirming its broad applicability. SRS is a crucial tool for assessing CRC patient survival rates and enhancing personalized medicine approaches.

In constructing the SRS, three SEMAs family genes were ultimately included: SEMA3E, SEMA4C, and SEMA6C. Class-3 SEMAs are a subfamily of seven vertebrate SEMAs known for being the only secreted type (44). Initially identified as axon guidance factors, they also play roles in immune responses, angiogenesis, lymphangiogenesis, and other physiological and developmental functions. Notably, SEMA3E is a particularly intriguing gene due to its dual role in cancer biology. Research has demonstrated that SEMA3E could inhibit tumor angiogenesis (45). However, SEMA3E also has an important role in promoting tumor progression. This pro-tumorigenic effect is mediated by activating the oncogenic tyrosine kinase ERBB2 and the PI3K/AKT signaling pathways (46, 47). Furin-like pro-protein convertases, commonly found in advanced invasive and metastatic cancers, regulate SEMA3E (48). These enzymes cleave SEMA3E into the p61 fragment, revealing a new signaling function. This fragment allows PLEXIND1 to interact with the oncogenic kinase ERBB2, initiating its signaling cascade. Although both SEMA3E and p61 bind to PlexinD1, only p61 can complex with ERBB2 and activate downstream pathways. Consequently, cancer cells may increase the conversion of precursor-SEMA3E into the pro-metastatic p61, promoting tumor progression (46). Additionally, A recent study revealed that SEMA3E knockout inhibits dendritic cell (DC) migration by modulating CCR7 expression and increasing programmed death ligand 2 levels, compared to wild-type mice (49). These findings illustrate that SEMA3E plays a role in regulating DC migration and function during inflammation. The transmembrane protein SEMA4C is overexpressed in several malignant tumors, including breast, esophageal, gastric, and colorectal cancers. The biological function of SEMA4C in macrophage recruitment was reported to contribute to the malignant nature of tumors. It could interact with the PLEXINB2 receptor to activate the NF-κB pathway, which induced the production of colony-stimulating factor 1, thereby promoting tumor growth and progression (50). Research on the role of SEMA6C in tumors is scarce. SEMA6C has recently been identified as a novel activator of focal adhesion kinase and Yes-associated protein that operates independently of cell adhesion mechanisms. This activation is essential for the viability and growth of cancer cells (51). By influencing key signaling pathways, SEMA6C plays a significant role in promoting tumor progression and survival, making it a potential target for therapeutic interventions in cancer treatment.

We analyzed immune-related signatures between the two groups using the GSEA algorithm and the IOBR R package. The SRS-high group showed significant activation of various oncogenic pathways and exhibited a greater propensity for a cold tumor phenotype (52). In contrast, the SRS-low group exhibited elevated TMB and TNB, meaning a richer variety of immune cell types and an increased presence of tumor neoantigens. These factors may enhance the immune response, making the tumor more easily recognized by the immune system, thereby improving the response to immunotherapy (53). Survival analysis indicated better prognostic outcomes for the SRS-low group, validated across multiple immunotherapy cohorts. Furthermore, TIDE analysis revealed an enhanced response to immunotherapy within the SRS-low group, indicating that SRS may serve as a valuable tool for the early identification of populations sensitive to immunotherapy.

scRNA-seq and ST were employed to explore the heterogeneity of malignant epithelial cells for a comprehensive understanding of the SRS in CRC. This study sought to elucidate the influence of SEMAs on the progression of CRC and its implications for immunotherapeutic strategies. Malignant epithelial cells with high SRS interacts strongly with myeloid and endothelial cells through SPP1 and COL4A2-ITGAV-ITGB8 signaling pathways, respectively. Similarly, Malignant epithelial cells with low SRS shows significant interactions with myeloid cells via MIF signaling and with endothelial cells through JAG1-NOTCH4 signaling pathways. ST analysis provided insights into the interaction patterns between various cell types within the tumor microenvironment. Malignant epithelial cells exhibiting high SRS demonstrated stronger communication with myeloid and endothelial cells compared to those with low SRS, aligning with scRNA-seq analysis findings. The heterogeneity observed indicates that malignant epithelial cells with varying SRS levels possess unique biological traits, highlighting SEMAs as a potential therapeutic target for CRC.

Our study conducted a comprehensive analysis of SEMAs gene expressions in CRC using bulk RNA, scRNA-seq and ST, revealing expression disparities among cellular subpopulations. The study highlights SEMAs’ involvement in tumor immune evasion and drug resistance. Nonetheless, we recognize various limitations within this study. Initially, we assessed and confirmed the SRS signature in both the training and external validation sets. These cohorts differed in size and sequencing platforms, although we utilized Z-score normalization to mitigate these differences. Validating our findings requires a large-scale, multi-center prospective study. Additionally, *in vivo* studies are required to elucidate the biological functions of SRS-related genes in CRC. In conclusion, although we have assessed the sensitivity of SRS-high subgroups to various small molecule drugs, further validation through *in vitro* drug assays and clinical trials is necessary.

## Conclusion

We developed the SRS using a machine learning framework, which improved performance in predicting patient prognosis across different cohorts and showed significant correlations with immunotherapy response. The SRS has the potential to be an effective tool for predicting prognosis and tailoring personalized treatment for CRC patients. Our study provides new insights into the molecular mechanisms of CRC development and progression through bulk RNA sequencing, scRNA-seq, and ST.

## Data Availability

The data used in this study are publicly available. The TCGA RNA-seq data and corresponding clinical data were downloaded from the "TCGAbiolinks" R package. The GSE17537, GSE39582, GSE126044, GSE135222, GSE91061, GSE132465, GSE144735, and GSE166555 were downloaded from the GEO (https://www.ncbi.nlm.nih.gov/geo/). IMvigor210 cohort was downloaded from the "IMvigor210CoreBiologies" R package. In-house cohort1 and In-house cohort2 is available from our previous study (https://data.mendeley.com/drafts/mdr6mgmvz3). Further inquiries can be directed to the corresponding authors.

## Glossary

ANT: adjacent normal tissue
AUC: area under the dose-response curve
CCK8: cell counting kit-8
CCLE: broad institute cancer cell line encyclopedia
CCLs: cancer cell lines
cDCs: conventional DCs
C-index: concordance index
CNV: copy number variation
COMMOT: COMMunication analysis by Optimal Transport
CRC: colorectal cancer
CTRP: cancer therapeutics response portal
DCs: dendritic cells
EMT: epithelial-mesenchymal transition
EpiH: malignant epithelial cells with SRS-high
EpiL: malignant epithelial cells with SRS-low
GDC: genomic data commons
GSEA: gene set enrichment analysis
GTEx: genotype-tissue expression
ICIs: immune checkpoint inhibitors
IOBR: Immuno-Oncology Biological Research
KEGG: Kyoto Encyclopedia of Genes and Genomes
Macro_SLC40A1: SLC40A1-positive macrophages
Macro_SPP1: SPP1-positive macrophages
MAF: mutation annotation format
OS: overall survival
PCA: principal component analysis
PCs: principal components
PMSF: phenylmethylsulfonyl fluoride
PRISM: profiling relative inhibition simultaneously in mixtures
PVDF: polyvinylidene fluoride
RCTD: robust cell type decomposition
RIPA: radio immunoprecipitation assay lysis
scRNA-seq: single-cell RNA sequencing
SEMAs: semaphorins
ST: spatial transcriptomic
TCGA: the cancer genome atlas
TIDE: tumor immune dysfunction and exclusion
TIP: tracking tumor immunophenotype
TMB: tumor mutation burden
TME: tumor microenvironment
TNB: tumor neoantigen burden
Tregs: regulatory T cells
UCEC: uterine corpus endometrial carcinoma
UMAP: uniform manifold approximation and projection
