# Supplementary material for: Integrative analysis of semaphorins family genes in colorectal cancer: implications for prognosis and immunotherapy
Source: Front Immunol. 2025 Mar 4;16:1536545. doi: 10.3389/fimmu.2025.1536545 (PMC11913869; doi:10.3389/fimmu.2025.1536545)
Supplement: Supplementary file 1 [file Table1.docx]

**Integrative Analysis of Semaphorins Family Genes in Colorectal Cancer: Implications for Prognosis and Immunotherapy**

Jiahao Zhu, Benjie Xu, Zhixing Wu, Zhiwei Yu, Shengjun Ji, Jie Lian, Haibo Lu

**Supplementary files**

Table S1. 27 solid tumors used in this study

Table S2. 10 machine learning algorithms applied in this study

Table S3. Sequences used in this study.

Table S4. Characteristic genes of myeloid cell subtypes.

Table S5. Characteristic genes of Endothelial cell subtypes.

Table S1. 27 solid tumors used in this study

| Tumor Type | Abbreviation |
| --- | --- |
| Bladder Urothelial Carcinoma | BLCA |
| Breast Invasive Carcinoma | BRCA |
| Cervical Squamous Cell Carcinoma and Endocervical Adenocarcinoma | CESC |
| Cholangiocarcinoma | CHOL |
| Colorectal Cancer | CRC |
| Esophageal Carcinoma | ESCA |
| Glioblastoma Multiforme | GBM |
| Head and Neck Squamous Cell Carcinoma | HNSC |
| Kidney Chromophobe | KICH |
| Kidney Renal Clear Cell Carcinoma | KIRC |
| Kidney Renal Papillary Cell Carcinoma | KIRP |
| Lower Grade Glioma | LGG |
| Liver Hepatocellular Carcinoma | LIHC |
| Lung Adenocarcinoma | LUAD |
| Lung Squamous Cell Carcinoma | LUSC |
| Ovarian Serous Cystadenocarcinoma | OV |
| Pancreatic Adenocarcinoma | PAAD |
| Pheochromocytoma and Paraganglioma | PCPG |
| Prostate Adenocarcinoma | PRAD |
| Sarcoma | SARC |
| Skin Cutaneous Melanoma | SKCM |
| Stomach Adenocarcinoma | STAD |
| Testicular Germ Cell Tumors | TGCT |
| Thyroid Carcinoma | THCA |
| Thymoma | THYM |
| Uterine Corpus Endometrial Carcinoma | UCEC |
| Uterine Carcinosarcoma | UCS |

Table S2. 10 machine learning algorithms applied in this study

| Method Name | Abbreviation |
| --- | --- |
| Cox Model with Gradient Boosting | CoxBoost |
| Stepwise Cox | Stepwise Cox |
| Least Absolute Shrinkage and Selection Operator | Lasso |
| Ridge | Ridge |
| Elastic Net Regularization | Enet |
| Survival Support Vector Machines (SVM) | Survival-SVMs |
| Generalized Boosted Regression Models (GBMs) | GBMs |
| Supervised Principal Components | SuperPC |
| Partial Least Squares Cox Regression | plsRcox |
| Random Survival Forests | RSF |

Table S3. Sequences used in this study.

| Category | Sequences |
| --- | --- |
| SEMA4C-siRNA #1 | 5'-GTGAGTACCAGTTGGAAGAGA -3' |
| SEMA4C-siRNA #2 | 5'-GGCCATCTGTGAGTACCAGTT -3' |
| SEMA4C-NC | 5'-TTCTCCGAACGTGTCACGT-3' |

Table S4.Characteristic genes of myeloid cell subtypes.

|  | p_val | avg_log2FC | pct.1 | pct.2 | p_val_adj | cluster | gene |
| --- | --- | --- | --- | --- | --- | --- | --- |
| TIMP1 | 0 | 2.931362431 | 0.962 | 0.77 | 0 | Monocyte | TIMP1 |
| S100A8 | 0 | 2.655222936 | 0.808 | 0.399 | 0 | Monocyte | S100A8 |
| S100A9 | 0 | 2.247626914 | 0.907 | 0.749 | 0 | Monocyte | S100A9 |
| S100A12 | 0 | 2.155502079 | 0.448 | 0.047 | 0 | Monocyte | S100A12 |
| IL1RN | 0 | 2.147983936 | 0.807 | 0.492 | 0 | Monocyte | IL1RN |
| FCN1 | 0 | 2.033556197 | 0.513 | 0.087 | 0 | Monocyte | FCN1 |
| SOD2 | 1.09E-303 | 1.314900795 | 0.972 | 0.932 | 3.38E-299 | Monocyte | SOD2 |
| BCL2A1 | 1.12E-294 | 1.531736964 | 0.907 | 0.732 | 3.47E-290 | Monocyte | BCL2A1 |
| SRGN | 1.97E-283 | 1.012951552 | 0.993 | 0.985 | 6.13E-279 | Monocyte | SRGN |
| IL1B | 4.88E-264 | 1.78316526 | 0.898 | 0.72 | 1.52E-259 | Monocyte | IL1B |
| EIF1 | 1.07E-243 | 0.780405742 | 0.99 | 0.975 | 3.33E-239 | Monocyte | EIF1 |
| EREG | 4.26E-229 | 1.691125625 | 0.744 | 0.429 | 1.33E-224 | Monocyte | EREG |
| FTH1 | 6.46E-211 | 0.933128729 | 1 | 1 | 2.01E-206 | Monocyte | FTH1 |
| VCAN | 3.59E-209 | 1.472257048 | 0.51 | 0.167 | 1.12E-204 | Monocyte | VCAN |
| NAMPT | 1.81E-202 | 0.939009862 | 0.935 | 0.854 | 5.63E-198 | Monocyte | NAMPT |
| SLC25A37 | 1.45E-201 | 1.463200692 | 0.504 | 0.193 | 4.51E-197 | Monocyte | SLC25A37 |
| CD44 | 6.08E-196 | 0.938556243 | 0.942 | 0.887 | 1.89E-191 | Monocyte | CD44 |
| C15orf48 | 2.39E-194 | 1.530865082 | 0.886 | 0.842 | 7.45E-190 | Monocyte | C15orf48 |
| AQP9 | 1.98E-192 | 1.313915588 | 0.555 | 0.242 | 6.17E-188 | Monocyte | AQP9 |
| CD300E | 1.34E-188 | 1.205872747 | 0.434 | 0.127 | 4.18E-184 | Monocyte | CD300E |
| RPS16 | 7.47E-179 | 0.60279588 | 0.99 | 0.977 | 2.32E-174 | Monocyte | RPS16 |
| SERPINA1 | 8.57E-177 | 0.926977982 | 0.814 | 0.644 | 2.67E-172 | Monocyte | SERPINA1 |
| MCEMP1 | 5.93E-164 | 1.10173251 | 0.374 | 0.096 | 1.85E-159 | Monocyte | MCEMP1 |
| APOBEC3A | 1.06E-160 | 1.476244649 | 0.268 | 0.033 | 3.30E-156 | Monocyte | APOBEC3A |
| MALAT1 | 2.36E-159 | 0.662314023 | 0.998 | 0.99 | 7.35E-155 | Monocyte | MALAT1 |
| BTG1 | 1.39E-157 | 0.862444557 | 0.933 | 0.893 | 4.32E-153 | Monocyte | BTG1 |
| SERPINB2 | 5.88E-153 | 2.118048328 | 0.245 | 0.027 | 1.83E-148 | Monocyte | SERPINB2 |
| PTGS2 | 1.06E-150 | 1.662044207 | 0.648 | 0.425 | 3.29E-146 | Monocyte | PTGS2 |
| H3F3A | 2.79E-145 | 0.58304477 | 0.972 | 0.974 | 8.68E-141 | Monocyte | H3F3A |
| LINC00936 | 6.21E-144 | 1.200146766 | 0.581 | 0.345 | 1.93E-139 | Monocyte | LINC00936 |
| SLC2A3 | 1.79E-143 | 1.358637875 | 0.707 | 0.538 | 5.57E-139 | Monocyte | SLC2A3 |
| PLAUR | 1.80E-142 | 0.921640412 | 0.923 | 0.886 | 5.61E-138 | Monocyte | PLAUR |
| CXCL8 | 9.92E-140 | 1.319606156 | 0.92 | 0.878 | 3.09E-135 | Monocyte | CXCL8 |
| RPS9 | 4.20E-137 | 0.510372643 | 0.993 | 0.98 | 1.31E-132 | Monocyte | RPS9 |
| TNIP3 | 4.65E-134 | 1.335078587 | 0.357 | 0.115 | 1.45E-129 | Monocyte | TNIP3 |
| TREM1 | 6.24E-133 | 1.099652115 | 0.56 | 0.326 | 1.94E-128 | Monocyte | TREM1 |
| G0S2 | 4.79E-130 | 1.058550698 | 0.834 | 0.658 | 1.49E-125 | Monocyte | G0S2 |
| RPL35A | 2.82E-129 | 0.518833979 | 0.977 | 0.962 | 8.78E-125 | Monocyte | RPL35A |
| UBA52 | 2.42E-123 | 0.445460083 | 0.988 | 0.976 | 7.53E-119 | Monocyte | UBA52 |
| RPS27 | 1.56E-119 | 0.510017017 | 0.995 | 0.989 | 4.85E-115 | Monocyte | RPS27 |
| MIR3945HG | 1.64E-119 | 1.233144446 | 0.335 | 0.112 | 5.09E-115 | Monocyte | MIR3945HG |
| SAT1 | 2.66E-114 | 0.603592701 | 0.996 | 0.994 | 8.28E-110 | Monocyte | SAT1 |
| TNFAIP6 | 6.65E-114 | 1.589354065 | 0.386 | 0.158 | 2.07E-109 | Monocyte | TNFAIP6 |
| EHD1 | 1.83E-113 | 0.996995368 | 0.501 | 0.286 | 5.68E-109 | Monocyte | EHD1 |
| H3F3B | 5.76E-107 | 0.541798364 | 0.986 | 0.977 | 1.79E-102 | Monocyte | H3F3B |
| ATP2B1 | 8.64E-104 | 1.051233239 | 0.598 | 0.452 | 2.69E-99 | Monocyte | ATP2B1 |
| SERPINB9 | 3.48E-101 | 0.911586188 | 0.656 | 0.494 | 1.08E-96 | Monocyte | SERPINB9 |
| PPIF | 9.00E-97 | 1.056011662 | 0.66 | 0.533 | 2.80E-92 | Monocyte | PPIF |
| FAU | 1.15E-96 | 0.399893617 | 0.981 | 0.971 | 3.57E-92 | Monocyte | FAU |
| CCL20 | 1.42E-95 | 1.797545531 | 0.659 | 0.508 | 4.43E-91 | Monocyte | CCL20 |
| TPT1 | 3.12E-95 | 0.434596231 | 0.997 | 0.996 | 9.70E-91 | Monocyte | TPT1 |
| CTB-61M7.2 | 2.80E-94 | 0.685684762 | 0.24 | 0.063 | 8.70E-90 | Monocyte | CTB-61M7.2 |
| RPL10 | 6.63E-94 | 0.367672065 | 0.999 | 0.996 | 2.06E-89 | Monocyte | RPL10 |
| CD48 | 3.61E-92 | 0.844943861 | 0.641 | 0.512 | 1.12E-87 | Monocyte | CD48 |
| WTAP | 4.07E-91 | 0.890961263 | 0.709 | 0.606 | 1.27E-86 | Monocyte | WTAP |
| GK | 3.87E-88 | 0.902930648 | 0.573 | 0.42 | 1.20E-83 | Monocyte | GK |
| CD55 | 2.50E-87 | 1.010103508 | 0.489 | 0.322 | 7.79E-83 | Monocyte | CD55 |
| NINJ1 | 6.10E-87 | 0.81424053 | 0.782 | 0.755 | 1.90E-82 | Monocyte | NINJ1 |
| RPS24 | 5.52E-86 | 0.453993477 | 0.985 | 0.986 | 1.72E-81 | Monocyte | RPS24 |
| CSTA | 1.52E-85 | 0.917682306 | 0.596 | 0.47 | 4.73E-81 | Monocyte | CSTA |
| RETN | 3.37E-84 | 0.974258617 | 0.292 | 0.105 | 1.05E-79 | Monocyte | RETN |
| CFP | 7.95E-84 | 0.741713211 | 0.295 | 0.112 | 2.47E-79 | Monocyte | CFP |
| RPL8 | 2.75E-81 | 0.371111527 | 0.976 | 0.976 | 8.55E-77 | Monocyte | RPL8 |
| LILRA5 | 1.04E-79 | 0.871374658 | 0.319 | 0.145 | 3.24E-75 | Monocyte | LILRA5 |
| RPL34 | 1.40E-77 | 0.458321457 | 0.993 | 0.991 | 4.37E-73 | Monocyte | RPL34 |
| METTL9 | 9.48E-76 | 0.871920068 | 0.404 | 0.243 | 2.95E-71 | Monocyte | METTL9 |
| STXBP2 | 4.40E-73 | 0.921534105 | 0.54 | 0.433 | 1.37E-68 | Monocyte | STXBP2 |
| FPR2 | 6.78E-73 | 0.630208721 | 0.202 | 0.057 | 2.11E-68 | Monocyte | FPR2 |
| RPL21 | 8.56E-73 | 0.371019762 | 0.994 | 0.992 | 2.66E-68 | Monocyte | RPL21 |
| UPP1 | 8.74E-73 | 0.798384311 | 0.65 | 0.55 | 2.72E-68 | Monocyte | UPP1 |
| RPL28 | 2.61E-71 | 0.32961952 | 0.995 | 0.996 | 8.12E-67 | Monocyte | RPL28 |
| CARD16 | 1.16E-70 | 0.853530824 | 0.599 | 0.517 | 3.60E-66 | Monocyte | CARD16 |
| CCL3L3 | 1.94E-70 | 1.610000009 | 0.804 | 0.707 | 6.05E-66 | Monocyte | CCL3L3 |
| RPL32 | 6.86E-70 | 0.387097126 | 0.988 | 0.991 | 2.13E-65 | Monocyte | RPL32 |
| NFKBIA | 1.24E-68 | 0.523558671 | 0.952 | 0.918 | 3.87E-64 | Monocyte | NFKBIA |
| ISG20 | 2.16E-65 | 0.995563962 | 0.43 | 0.273 | 6.73E-61 | Monocyte | ISG20 |
| CXCL2 | 2.34E-65 | 1.087693224 | 0.829 | 0.785 | 7.28E-61 | Monocyte | CXCL2 |
| PNRC1 | 3.27E-65 | 0.579421267 | 0.891 | 0.867 | 1.02E-60 | Monocyte | PNRC1 |
| THBS1 | 3.77E-65 | 1.098298289 | 0.338 | 0.169 | 1.17E-60 | Monocyte | THBS1 |
| PFDN5 | 2.66E-64 | 0.394186261 | 0.944 | 0.945 | 8.27E-60 | Monocyte | PFDN5 |
| RPLP2 | 3.94E-64 | 0.31405896 | 0.99 | 0.993 | 1.23E-59 | Monocyte | RPLP2 |
| RPL39 | 6.09E-64 | 0.367349896 | 0.996 | 0.992 | 1.89E-59 | Monocyte | RPL39 |
| OLR1 | 8.15E-63 | 0.926286952 | 0.566 | 0.446 | 2.54E-58 | Monocyte | OLR1 |
| CLEC4E | 2.22E-62 | 0.739254909 | 0.387 | 0.225 | 6.90E-58 | Monocyte | CLEC4E |
| S100A4 | 5.18E-62 | 0.559550684 | 0.914 | 0.903 | 1.61E-57 | Monocyte | S100A4 |
| FPR1 | 2.59E-60 | 0.775906972 | 0.443 | 0.305 | 8.07E-56 | Monocyte | FPR1 |
| IL1A | 3.03E-60 | 0.961670879 | 0.369 | 0.212 | 9.41E-56 | Monocyte | IL1A |
| EEF1D | 1.32E-59 | 0.391209654 | 0.909 | 0.906 | 4.09E-55 | Monocyte | EEF1D |
| CCL3 | 2.59E-59 | 1.096752563 | 0.869 | 0.822 | 8.06E-55 | Monocyte | CCL3 |
| PLAC8 | 1.34E-58 | 1.052489948 | 0.267 | 0.118 | 4.18E-54 | Monocyte | PLAC8 |
| ACSL1 | 3.15E-58 | 0.856530778 | 0.423 | 0.287 | 9.80E-54 | Monocyte | ACSL1 |
| GCH1 | 2.33E-57 | 0.707991709 | 0.329 | 0.185 | 7.26E-53 | Monocyte | GCH1 |
| KYNU | 1.52E-56 | 0.746124888 | 0.548 | 0.454 | 4.73E-52 | Monocyte | KYNU |
| TNFSF14 | 5.22E-56 | 0.594241986 | 0.164 | 0.047 | 1.63E-51 | Monocyte | TNFSF14 |
| SAMSN1 | 9.74E-56 | 0.678911772 | 0.619 | 0.541 | 3.03E-51 | Monocyte | SAMSN1 |
| GAPDH | 2.53E-55 | 0.455516774 | 0.986 | 0.983 | 7.89E-51 | Monocyte | GAPDH |
| PID1 | 3.85E-55 | 0.55703383 | 0.188 | 0.065 | 1.20E-50 | Monocyte | PID1 |
| EIF1B | 6.02E-55 | 1.009974675 | 0.52 | 0.446 | 1.87E-50 | Monocyte | EIF1B |
| RPL26 | 1.36E-49 | 0.385703633 | 0.979 | 0.973 | 4.23E-45 | Monocyte | RPL26 |
| TNFRSF1B | 5.40E-49 | 0.71613314 | 0.537 | 0.453 | 1.68E-44 | Monocyte | TNFRSF1B |
| MIR4435-2HG | 5.23E-48 | 0.894157137 | 0.442 | 0.337 | 1.63E-43 | Monocyte | MIR4435-2HG |
| DNAAF1 | 2.06E-47 | 0.666748077 | 0.172 | 0.062 | 6.40E-43 | Monocyte | DNAAF1 |
| RPL30 | 2.31E-47 | 0.359725059 | 0.965 | 0.956 | 7.19E-43 | Monocyte | RPL30 |
| RPS13 | 4.47E-47 | 0.379997281 | 0.966 | 0.974 | 1.39E-42 | Monocyte | RPS13 |
| LINC00152 | 5.10E-47 | 0.924103232 | 0.566 | 0.505 | 1.59E-42 | Monocyte | LINC00152 |
| C4orf3 | 6.79E-47 | 0.610618198 | 0.757 | 0.764 | 2.11E-42 | Monocyte | C4orf3 |
| PTX3 | 8.43E-47 | 0.820936018 | 0.136 | 0.038 | 2.62E-42 | Monocyte | PTX3 |
| SERPINB1 | 1.17E-46 | 0.560152684 | 0.644 | 0.603 | 3.64E-42 | Monocyte | SERPINB1 |
| CCRL2 | 7.95E-46 | 0.734568099 | 0.484 | 0.379 | 2.47E-41 | Monocyte | CCRL2 |
| CDC42EP3 | 9.21E-46 | 0.627340807 | 0.234 | 0.113 | 2.87E-41 | Monocyte | CDC42EP3 |
| RPL24 | 4.09E-45 | 0.354412918 | 0.928 | 0.939 | 1.27E-40 | Monocyte | RPL24 |
| GSTO1 | 9.43E-45 | 0.43293606 | 0.791 | 0.777 | 2.93E-40 | Monocyte | GSTO1 |
| RPL11 | 9.69E-45 | 0.30343384 | 0.988 | 0.98 | 3.01E-40 | Monocyte | RPL11 |
| INHBA | 1.49E-43 | 0.92208417 | 0.399 | 0.267 | 4.62E-39 | Monocyte | INHBA |
| AC013461.1 | 2.29E-43 | 0.497985186 | 0.194 | 0.083 | 7.14E-39 | Monocyte | AC013461.1 |
| ATP13A3 | 6.79E-43 | 0.640791549 | 0.542 | 0.458 | 2.11E-38 | Monocyte | ATP13A3 |
| S100A10 | 1.80E-42 | 0.355043499 | 0.935 | 0.941 | 5.59E-38 | Monocyte | S100A10 |
| TNIP1 | 3.81E-42 | 0.673873474 | 0.472 | 0.387 | 1.18E-37 | Monocyte | TNIP1 |
| LDHA | 3.86E-42 | 0.55040432 | 0.812 | 0.813 | 1.20E-37 | Monocyte | LDHA |
| ANXA5 | 5.35E-41 | 0.476906792 | 0.856 | 0.881 | 1.66E-36 | Monocyte | ANXA5 |
| EMP3 | 2.83E-40 | 0.42435633 | 0.822 | 0.816 | 8.82E-36 | Monocyte | EMP3 |
| RPL13 | 1.06E-39 | 0.273223686 | 0.994 | 0.998 | 3.31E-35 | Monocyte | RPL13 |
| SEC61G | 1.23E-39 | 0.43359677 | 0.8 | 0.815 | 3.81E-35 | Monocyte | SEC61G |
| MAP4K4 | 1.58E-39 | 0.5470729 | 0.243 | 0.128 | 4.90E-35 | Monocyte | MAP4K4 |
| RPS12 | 3.56E-39 | 0.31528198 | 0.986 | 0.99 | 1.11E-34 | Monocyte | RPS12 |
| RPS15A | 1.05E-38 | 0.323835089 | 0.981 | 0.983 | 3.26E-34 | Monocyte | RPS15A |
| RP11-670E13.6 | 1.51E-38 | 0.721514302 | 0.298 | 0.184 | 4.70E-34 | Monocyte | RP11-670E13.6 |
| SH3BGRL3 | 1.73E-38 | 0.303757961 | 0.96 | 0.958 | 5.37E-34 | Monocyte | SH3BGRL3 |
| NLRP3 | 2.09E-38 | 0.748053584 | 0.391 | 0.279 | 6.50E-34 | Monocyte | NLRP3 |
| RPL18A | 3.06E-38 | 0.302482702 | 0.981 | 0.987 | 9.53E-34 | Monocyte | RPL18A |
| S100A6 | 3.70E-38 | 0.286873368 | 0.979 | 0.981 | 1.15E-33 | Monocyte | S100A6 |
| RPL29 | 4.24E-37 | 0.311988327 | 0.954 | 0.954 | 1.32E-32 | Monocyte | RPL29 |
| MARCKSL1 | 2.06E-36 | 0.693201021 | 0.473 | 0.38 | 6.42E-32 | Monocyte | MARCKSL1 |
| WARS | 7.26E-36 | 0.730842532 | 0.402 | 0.307 | 2.26E-31 | Monocyte | WARS |
| ANPEP | 6.58E-35 | 0.610546724 | 0.381 | 0.282 | 2.05E-30 | Monocyte | ANPEP |
| ITGB8 | 1.17E-34 | 0.359878023 | 0.117 | 0.038 | 3.64E-30 | Monocyte | ITGB8 |
| VEGFA | 3.65E-34 | 0.621299989 | 0.496 | 0.41 | 1.13E-29 | Monocyte | VEGFA |
| BASP1 | 4.35E-34 | 0.670455787 | 0.502 | 0.432 | 1.35E-29 | Monocyte | BASP1 |
| RPL9 | 1.16E-33 | 0.276424232 | 0.978 | 0.979 | 3.61E-29 | Monocyte | RPL9 |
| NUMB | 2.19E-33 | 0.663005717 | 0.395 | 0.313 | 6.80E-29 | Monocyte | NUMB |
| IER3 | 9.72E-33 | 0.340050002 | 0.883 | 0.857 | 3.02E-28 | Monocyte | IER3 |
| RPL7 | 2.87E-31 | 0.29597665 | 0.975 | 0.975 | 8.92E-27 | Monocyte | RPL7 |
| BZW1 | 5.41E-31 | 0.583494018 | 0.53 | 0.501 | 1.68E-26 | Monocyte | BZW1 |
| CREM | 8.25E-31 | 0.511944308 | 0.719 | 0.707 | 2.57E-26 | Monocyte | CREM |
| IRG1 | 5.44E-30 | 0.391917704 | 0.12 | 0.044 | 1.69E-25 | Monocyte | IRG1 |
| VIM-AS1 | 1.41E-29 | 0.42225283 | 0.16 | 0.075 | 4.40E-25 | Monocyte | VIM-AS1 |
| PTGER2 | 1.62E-29 | 0.429054683 | 0.201 | 0.108 | 5.05E-25 | Monocyte | PTGER2 |
| RPS28 | 2.23E-29 | 0.250815504 | 0.984 | 0.983 | 6.93E-25 | Monocyte | RPS28 |
| AC133644.2 | 3.17E-29 | 0.369711029 | 0.132 | 0.054 | 9.85E-25 | Monocyte | AC133644.2 |
| RPL37 | 3.65E-29 | 0.281435166 | 0.961 | 0.957 | 1.14E-24 | Monocyte | RPL37 |
| IFITM2 | 2.46E-28 | 0.678241149 | 0.508 | 0.448 | 7.66E-24 | Monocyte | IFITM2 |
| IVNS1ABP | 3.09E-28 | 0.641545816 | 0.441 | 0.378 | 9.60E-24 | Monocyte | IVNS1ABP |
| NRG1 | 3.26E-28 | 0.419132339 | 0.18 | 0.09 | 1.02E-23 | Monocyte | NRG1 |
| CD52 | 3.81E-28 | 0.60945545 | 0.477 | 0.386 | 1.18E-23 | Monocyte | CD52 |
| PNP | 4.65E-28 | 0.568572555 | 0.324 | 0.236 | 1.45E-23 | Monocyte | PNP |
| PPP1R15A | 1.20E-27 | 0.37296576 | 0.793 | 0.757 | 3.73E-23 | Monocyte | PPP1R15A |
| NFKB1 | 1.50E-27 | 0.455818565 | 0.623 | 0.581 | 4.66E-23 | Monocyte | NFKB1 |
| SERP1 | 2.79E-27 | 0.389251115 | 0.753 | 0.781 | 8.69E-23 | Monocyte | SERP1 |
| PRELID1 | 8.09E-27 | 0.447355169 | 0.678 | 0.684 | 2.52E-22 | Monocyte | PRELID1 |
| DUSP6 | 2.64E-26 | 0.675638083 | 0.429 | 0.353 | 8.21E-22 | Monocyte | DUSP6 |
| RPS8 | 8.43E-26 | 0.298162079 | 0.945 | 0.959 | 2.62E-21 | Monocyte | RPS8 |
| CFLAR | 1.51E-25 | 0.378785143 | 0.692 | 0.686 | 4.69E-21 | Monocyte | CFLAR |
| SLC7A5 | 2.52E-25 | 0.503151552 | 0.335 | 0.251 | 7.84E-21 | Monocyte | SLC7A5 |
| LCP1 | 2.56E-24 | 0.37480401 | 0.7 | 0.707 | 7.96E-20 | Monocyte | LCP1 |
| FCER1G | 5.28E-24 | 0.263895891 | 0.978 | 0.986 | 1.64E-19 | Monocyte | FCER1G |
| CASP1 | 7.13E-24 | 0.503847534 | 0.462 | 0.423 | 2.22E-19 | Monocyte | CASP1 |
| FLT1 | 2.19E-23 | 0.378954519 | 0.138 | 0.067 | 6.82E-19 | Monocyte | FLT1 |
| ETS2 | 2.25E-23 | 0.455849106 | 0.598 | 0.583 | 7.01E-19 | Monocyte | ETS2 |
| AK4 | 5.37E-23 | 0.322559343 | 0.11 | 0.046 | 1.67E-18 | Monocyte | AK4 |
| LINC01272 | 6.19E-23 | 0.47867724 | 0.45 | 0.382 | 1.93E-18 | Monocyte | LINC01272 |
| IRAK2 | 1.09E-22 | 0.409550715 | 0.239 | 0.156 | 3.39E-18 | Monocyte | IRAK2 |
| AREG | 3.02E-22 | 0.838226985 | 0.478 | 0.404 | 9.40E-18 | Monocyte | AREG |
| SLC11A1 | 3.77E-22 | 0.357951463 | 0.47 | 0.389 | 1.17E-17 | Monocyte | SLC11A1 |
| DDX60L | 4.12E-22 | 0.422558829 | 0.193 | 0.117 | 1.28E-17 | Monocyte | DDX60L |
| F3 | 1.09E-21 | 0.587254628 | 0.128 | 0.063 | 3.40E-17 | Monocyte | F3 |
| IDO1 | 2.33E-21 | 0.4289629 | 0.137 | 0.067 | 7.26E-17 | Monocyte | IDO1 |
| OASL | 3.36E-21 | 0.451006997 | 0.207 | 0.13 | 1.04E-16 | Monocyte | OASL |
| TWISTNB | 3.07E-20 | 0.916735778 | 0.364 | 0.296 | 9.57E-16 | Monocyte | TWISTNB |
| PLEK | 2.63E-19 | 0.515649986 | 0.639 | 0.655 | 8.18E-15 | Monocyte | PLEK |
| HPSE | 3.15E-19 | 0.510331979 | 0.229 | 0.159 | 9.79E-15 | Monocyte | HPSE |
| ARFGAP3 | 4.60E-19 | 0.520396471 | 0.348 | 0.296 | 1.43E-14 | Monocyte | ARFGAP3 |
| DENND5A | 5.61E-19 | 0.432820563 | 0.265 | 0.194 | 1.75E-14 | Monocyte | DENND5A |
| RILPL2 | 1.15E-18 | 0.452724755 | 0.527 | 0.514 | 3.57E-14 | Monocyte | RILPL2 |
| NBPF19 | 2.07E-18 | 0.360677041 | 0.169 | 0.102 | 6.45E-14 | Monocyte | NBPF19 |
| DSE | 2.67E-18 | 0.482874261 | 0.448 | 0.42 | 8.31E-14 | Monocyte | DSE |
| XBP1 | 3.91E-18 | 0.453094902 | 0.519 | 0.507 | 1.22E-13 | Monocyte | XBP1 |
| PHACTR1 | 6.01E-18 | 0.505836488 | 0.44 | 0.401 | 1.87E-13 | Monocyte | PHACTR1 |
| PGK1 | 6.83E-18 | 0.354395914 | 0.737 | 0.769 | 2.13E-13 | Monocyte | PGK1 |
| PNPLA8 | 8.80E-18 | 0.458077649 | 0.349 | 0.294 | 2.74E-13 | Monocyte | PNPLA8 |
| TPI1 | 2.30E-17 | 0.374187033 | 0.839 | 0.893 | 7.14E-13 | Monocyte | TPI1 |
| CCL4 | 2.73E-17 | 0.627275053 | 0.762 | 0.73 | 8.50E-13 | Monocyte | CCL4 |
| IL1R1 | 4.14E-17 | 0.293535936 | 0.124 | 0.067 | 1.29E-12 | Monocyte | IL1R1 |
| IFITM3 | 5.41E-17 | 0.561641338 | 0.735 | 0.773 | 1.68E-12 | Monocyte | IFITM3 |
| SLC2A6 | 6.59E-17 | 0.357540201 | 0.179 | 0.115 | 2.05E-12 | Monocyte | SLC2A6 |
| HSPB1 | 1.31E-16 | 0.610538851 | 0.562 | 0.752 | 4.09E-12 | Monocyte | HSPB1 |
| MAP3K8 | 1.71E-16 | 0.539255811 | 0.53 | 0.533 | 5.33E-12 | Monocyte | MAP3K8 |
| IL3RA | 1.82E-16 | 0.335613926 | 0.171 | 0.107 | 5.66E-12 | Monocyte | IL3RA |
| SELL | 8.21E-16 | 0.380073671 | 0.117 | 0.064 | 2.55E-11 | Monocyte | SELL |
| BNIP3L | 1.89E-15 | 0.633262969 | 0.53 | 0.55 | 5.90E-11 | Monocyte | BNIP3L |
| GLIPR2 | 2.44E-15 | 0.433911975 | 0.454 | 0.434 | 7.58E-11 | Monocyte | GLIPR2 |
| CRADD | 5.89E-15 | 0.369481258 | 0.148 | 0.091 | 1.83E-10 | Monocyte | CRADD |
| IRAK3 | 1.19E-14 | 0.376437029 | 0.234 | 0.175 | 3.69E-10 | Monocyte | IRAK3 |
| CKAP4 | 1.22E-14 | 0.28157359 | 0.119 | 0.067 | 3.81E-10 | Monocyte | CKAP4 |
| B4GALT5 | 2.51E-14 | 0.430326574 | 0.25 | 0.194 | 7.82E-10 | Monocyte | B4GALT5 |
| JHDM1D-AS1 | 2.66E-14 | 0.286834319 | 0.119 | 0.068 | 8.26E-10 | Monocyte | JHDM1D-AS1 |
| SUB1 | 3.77E-14 | 0.250644231 | 0.801 | 0.829 | 1.17E-09 | Monocyte | SUB1 |
| MT2A | 9.57E-14 | 1.039792989 | 0.644 | 0.666 | 2.98E-09 | Monocyte | MT2A |
| IGHM | 1.48E-13 | 0.288199378 | 0.066 | 0.126 | 4.61E-09 | Monocyte | IGHM |
| RP11-295G20.2 | 1.76E-13 | 0.310661355 | 0.113 | 0.065 | 5.49E-09 | Monocyte | RP11-295G20.2 |
| RIPK2 | 3.86E-13 | 0.457196601 | 0.372 | 0.339 | 1.20E-08 | Monocyte | RIPK2 |
| YWHAZ | 3.96E-13 | 0.291835267 | 0.712 | 0.765 | 1.23E-08 | Monocyte | YWHAZ |
| EAF1 | 8.73E-13 | 0.432646204 | 0.257 | 0.206 | 2.72E-08 | Monocyte | EAF1 |
| LCP2 | 9.48E-13 | 0.452906003 | 0.52 | 0.539 | 2.95E-08 | Monocyte | LCP2 |
| PLSCR1 | 1.10E-12 | 0.346174744 | 0.568 | 0.593 | 3.44E-08 | Monocyte | PLSCR1 |
| SPHK1 | 1.86E-12 | 0.426940825 | 0.349 | 0.313 | 5.78E-08 | Monocyte | SPHK1 |
| HSPA1A | 2.04E-12 | 1.296597748 | 0.59 | 0.624 | 6.34E-08 | Monocyte | HSPA1A |
| AC058791.1 | 2.50E-12 | 0.353825767 | 0.195 | 0.142 | 7.79E-08 | Monocyte | AC058791.1 |
| ZEB2 | 2.63E-12 | 0.346847865 | 0.574 | 0.603 | 8.19E-08 | Monocyte | ZEB2 |
| TNFAIP8 | 2.84E-12 | 0.389958349 | 0.494 | 0.48 | 8.83E-08 | Monocyte | TNFAIP8 |
| AP1S2 | 3.53E-12 | 0.480135977 | 0.448 | 0.447 | 1.10E-07 | Monocyte | AP1S2 |
| CLEC12A | 4.09E-12 | 0.361090838 | 0.182 | 0.132 | 1.27E-07 | Monocyte | CLEC12A |
| CDA | 4.31E-12 | 0.256819111 | 0.101 | 0.057 | 1.34E-07 | Monocyte | CDA |
| RPS4Y1 | 4.41E-12 | 0.35182024 | 0.285 | 0.222 | 1.37E-07 | Monocyte | RPS4Y1 |
| SLC7A11 | 5.32E-12 | 0.417754311 | 0.148 | 0.096 | 1.66E-07 | Monocyte | SLC7A11 |
| PDE4B | 5.50E-12 | 0.391776125 | 0.469 | 0.457 | 1.71E-07 | Monocyte | PDE4B |
| CCL4L2 | 6.98E-12 | 0.841554183 | 0.68 | 0.676 | 2.17E-07 | Monocyte | CCL4L2 |
| GPCPD1 | 7.21E-12 | 0.462154609 | 0.351 | 0.324 | 2.24E-07 | Monocyte | GPCPD1 |
| HS3ST3B1 | 1.60E-11 | 0.319285842 | 0.143 | 0.095 | 4.99E-07 | Monocyte | HS3ST3B1 |
| CXCL3 | 2.45E-11 | 0.890760454 | 0.73 | 0.751 | 7.62E-07 | Monocyte | CXCL3 |
| CEBPB | 2.46E-11 | 0.316787183 | 0.766 | 0.798 | 7.64E-07 | Monocyte | CEBPB |
| RNF19B | 3.28E-11 | 0.423202721 | 0.319 | 0.282 | 1.02E-06 | Monocyte | RNF19B |
| EMP1 | 3.57E-11 | 0.47434226 | 0.28 | 0.235 | 1.11E-06 | Monocyte | EMP1 |
| THAP2 | 4.25E-11 | 0.397192967 | 0.391 | 0.352 | 1.32E-06 | Monocyte | THAP2 |
| GPR84 | 5.29E-11 | 0.455752024 | 0.248 | 0.205 | 1.64E-06 | Monocyte | GPR84 |
| IL23A | 5.36E-11 | 0.470564443 | 0.145 | 0.094 | 1.67E-06 | Monocyte | IL23A |
| MYO1G | 5.61E-11 | 0.349595841 | 0.282 | 0.239 | 1.74E-06 | Monocyte | MYO1G |
| TRAF1 | 6.12E-11 | 0.339236195 | 0.262 | 0.214 | 1.90E-06 | Monocyte | TRAF1 |
| CDKN1A | 7.37E-11 | 0.353407497 | 0.552 | 0.559 | 2.29E-06 | Monocyte | CDKN1A |
| SEMA6B | 4.22E-10 | 0.285895223 | 0.154 | 0.107 | 1.31E-05 | Monocyte | SEMA6B |
| QPCT | 5.51E-10 | 0.261590383 | 0.171 | 0.123 | 1.71E-05 | Monocyte | QPCT |
| TMEM167A | 9.87E-10 | 0.385328514 | 0.509 | 0.541 | 3.07E-05 | Monocyte | TMEM167A |
| ICAM3 | 3.13E-09 | 0.273419368 | 0.131 | 0.091 | 9.74E-05 | Monocyte | ICAM3 |
| CASP4 | 3.39E-09 | 0.409350508 | 0.382 | 0.381 | 0.000105513 | Monocyte | CASP4 |
| ADAM19 | 4.31E-09 | 0.314209568 | 0.145 | 0.102 | 0.000134168 | Monocyte | ADAM19 |
| JARID2 | 5.97E-09 | 0.381694582 | 0.281 | 0.248 | 0.000185751 | Monocyte | JARID2 |
| HSP90AA1 | 1.08E-08 | 1.160864699 | 0.87 | 0.93 | 0.000336315 | Monocyte | HSP90AA1 |
| BACH1 | 1.11E-08 | 0.370865031 | 0.292 | 0.265 | 0.00034609 | Monocyte | BACH1 |
| LYZ | 1.26E-08 | 0.272256901 | 0.912 | 0.955 | 0.000392915 | Monocyte | LYZ |
| FNDC3B | 1.66E-08 | 0.358700193 | 0.442 | 0.447 | 0.000518003 | Monocyte | FNDC3B |
| BTG3 | 1.67E-08 | 0.358577756 | 0.282 | 0.253 | 0.00051942 | Monocyte | BTG3 |
| ATP1B3 | 1.87E-08 | 0.262133715 | 0.72 | 0.771 | 0.000580354 | Monocyte | ATP1B3 |
| RYBP | 2.14E-08 | 0.32957478 | 0.201 | 0.163 | 0.000666987 | Monocyte | RYBP |
| HCAR3 | 2.40E-08 | 0.304145807 | 0.113 | 0.076 | 0.000745894 | Monocyte | HCAR3 |
| STX11 | 4.48E-08 | 0.335422537 | 0.518 | 0.531 | 0.001393196 | Monocyte | STX11 |
| SRSF5 | 4.84E-08 | 0.348204515 | 0.573 | 0.636 | 0.001506295 | Monocyte | SRSF5 |
| IRF7 | 5.42E-08 | 0.400090786 | 0.279 | 0.254 | 0.001686991 | Monocyte | IRF7 |
| IL6 | 6.85E-08 | 1.052991594 | 0.359 | 0.331 | 0.002132462 | Monocyte | IL6 |
| PHLDA2 | 9.47E-08 | 0.383581348 | 0.461 | 0.462 | 0.002945953 | Monocyte | PHLDA2 |
| HOTAIRM1 | 1.07E-07 | 0.271120089 | 0.154 | 0.117 | 0.003344285 | Monocyte | HOTAIRM1 |
| PLD1 | 1.63E-07 | 0.272342428 | 0.119 | 0.085 | 0.005084314 | Monocyte | PLD1 |
| PFKFB3 | 1.83E-07 | 0.357346875 | 0.405 | 0.403 | 0.005707421 | Monocyte | PFKFB3 |
| GBP5 | 2.92E-07 | 0.382828898 | 0.19 | 0.154 | 0.009091124 | Monocyte | GBP5 |
| ZFAS1 | 4.82E-07 | 0.273649959 | 0.637 | 0.694 | 0.015012929 | Monocyte | ZFAS1 |
| DNTTIP2 | 5.52E-07 | 0.362432051 | 0.271 | 0.25 | 0.017169256 | Monocyte | DNTTIP2 |
| KDM7A | 6.74E-07 | 0.294917942 | 0.164 | 0.131 | 0.020987203 | Monocyte | KDM7A |
| RP11-796E2.4 | 7.28E-07 | 0.266223595 | 0.153 | 0.118 | 0.02263927 | Monocyte | RP11-796E2.4 |
| ELL2 | 9.38E-07 | 0.312635737 | 0.381 | 0.373 | 0.029202004 | Monocyte | ELL2 |
| FLNA | 9.40E-07 | 0.304341841 | 0.516 | 0.542 | 0.029261421 | Monocyte | FLNA |
| C1QC | 0 | 1.995445603 | 0.982 | 0.412 | 0 | Macro_SLC40A1 | C1QC |
| C1QB | 0 | 1.977508317 | 0.982 | 0.444 | 0 | Macro_SLC40A1 | C1QB |
| C1QA | 3.22E-301 | 1.82516609 | 0.989 | 0.476 | 1.00E-296 | Macro_SLC40A1 | C1QA |
| ADAMDEC1 | 7.00E-253 | 1.218426884 | 0.61 | 0.123 | 2.18E-248 | Macro_SLC40A1 | ADAMDEC1 |
| PLD3 | 1.78E-208 | 1.289822804 | 0.899 | 0.392 | 5.54E-204 | Macro_SLC40A1 | PLD3 |
| SLC40A1 | 2.69E-208 | 1.350497226 | 0.603 | 0.141 | 8.36E-204 | Macro_SLC40A1 | SLC40A1 |
| TMEM176B | 3.74E-208 | 1.337175805 | 0.933 | 0.506 | 1.16E-203 | Macro_SLC40A1 | TMEM176B |
| ENPP2 | 8.88E-190 | 0.790016454 | 0.48 | 0.092 | 2.76E-185 | Macro_SLC40A1 | ENPP2 |
| HLA-DMB | 1.68E-183 | 1.16549568 | 0.932 | 0.507 | 5.21E-179 | Macro_SLC40A1 | HLA-DMB |
| CD74 | 4.99E-177 | 1.077215885 | 1 | 0.958 | 1.55E-172 | Macro_SLC40A1 | CD74 |
| TSPAN4 | 1.78E-172 | 0.881866181 | 0.683 | 0.218 | 5.54E-168 | Macro_SLC40A1 | TSPAN4 |
| AKR1B1 | 9.10E-171 | 0.949184693 | 0.78 | 0.295 | 2.83E-166 | Macro_SLC40A1 | AKR1B1 |
| FUCA1 | 6.07E-166 | 0.919594789 | 0.543 | 0.139 | 1.89E-161 | Macro_SLC40A1 | FUCA1 |
| GRN | 2.63E-165 | 0.967862221 | 0.986 | 0.748 | 8.20E-161 | Macro_SLC40A1 | GRN |
| PSAP | 6.06E-163 | 0.956792891 | 0.996 | 0.938 | 1.89E-158 | Macro_SLC40A1 | PSAP |
| CTSC | 5.53E-159 | 1.281740484 | 0.961 | 0.616 | 1.72E-154 | Macro_SLC40A1 | CTSC |
| TMEM176A | 1.59E-157 | 1.019981229 | 0.87 | 0.426 | 4.94E-153 | Macro_SLC40A1 | TMEM176A |
| C1orf54 | 3.59E-157 | 0.786916995 | 0.709 | 0.24 | 1.12E-152 | Macro_SLC40A1 | C1orf54 |
| ITM2B | 5.52E-157 | 1.01153412 | 0.996 | 0.843 | 1.72E-152 | Macro_SLC40A1 | ITM2B |
| ACP5 | 2.21E-156 | 1.169474863 | 0.892 | 0.477 | 6.88E-152 | Macro_SLC40A1 | ACP5 |
| C2 | 5.22E-156 | 0.751447983 | 0.576 | 0.16 | 1.62E-151 | Macro_SLC40A1 | C2 |
| AXL | 3.67E-154 | 0.848948585 | 0.555 | 0.153 | 1.14E-149 | Macro_SLC40A1 | AXL |
| MS4A6A | 1.03E-149 | 1.049218394 | 0.945 | 0.569 | 3.19E-145 | Macro_SLC40A1 | MS4A6A |
| A2M | 1.14E-147 | 0.847842386 | 0.674 | 0.228 | 3.55E-143 | Macro_SLC40A1 | A2M |
| APOE | 5.42E-146 | 1.682499035 | 0.862 | 0.458 | 1.69E-141 | Macro_SLC40A1 | APOE |
| CTSD | 1.33E-145 | 1.020606702 | 0.998 | 0.759 | 4.15E-141 | Macro_SLC40A1 | CTSD |
| LGALS3BP | 1.68E-144 | 0.73345009 | 0.672 | 0.228 | 5.24E-140 | Macro_SLC40A1 | LGALS3BP |
| RGS1 | 5.13E-141 | 1.12804323 | 0.938 | 0.563 | 1.60E-136 | Macro_SLC40A1 | RGS1 |
| IL18 | 7.69E-141 | 0.748548976 | 0.633 | 0.211 | 2.39E-136 | Macro_SLC40A1 | IL18 |
| PTMS | 2.35E-138 | 0.746275398 | 0.822 | 0.344 | 7.30E-134 | Macro_SLC40A1 | PTMS |
| SLCO2B1 | 7.08E-138 | 0.640810286 | 0.698 | 0.24 | 2.20E-133 | Macro_SLC40A1 | SLCO2B1 |
| HEXA | 1.74E-137 | 0.74140751 | 0.755 | 0.301 | 5.42E-133 | Macro_SLC40A1 | HEXA |
| HLA-DPA1 | 1.76E-132 | 0.985746935 | 0.998 | 0.856 | 5.46E-128 | Macro_SLC40A1 | HLA-DPA1 |
| HLA-DPB1 | 5.27E-132 | 0.860822762 | 0.996 | 0.864 | 1.64E-127 | Macro_SLC40A1 | HLA-DPB1 |
| HLA-DMA | 6.01E-131 | 0.863896977 | 0.969 | 0.675 | 1.87E-126 | Macro_SLC40A1 | HLA-DMA |
| PRDX1 | 1.90E-130 | 0.979202106 | 0.973 | 0.727 | 5.92E-126 | Macro_SLC40A1 | PRDX1 |
| MGST2 | 4.89E-130 | 0.666737135 | 0.696 | 0.257 | 1.52E-125 | Macro_SLC40A1 | MGST2 |
| ABI3 | 4.66E-129 | 0.646181945 | 0.587 | 0.19 | 1.45E-124 | Macro_SLC40A1 | ABI3 |
| ADAP2 | 6.60E-129 | 0.633932291 | 0.609 | 0.202 | 2.05E-124 | Macro_SLC40A1 | ADAP2 |
| FCGRT | 1.05E-127 | 0.877254686 | 0.967 | 0.663 | 3.27E-123 | Macro_SLC40A1 | FCGRT |
| HLA-DQB1 | 1.78E-127 | 0.938780013 | 0.985 | 0.748 | 5.54E-123 | Macro_SLC40A1 | HLA-DQB1 |
| GPR34 | 5.99E-127 | 0.633889352 | 0.517 | 0.15 | 1.86E-122 | Macro_SLC40A1 | GPR34 |
| AKR1A1 | 2.75E-126 | 0.756450252 | 0.841 | 0.404 | 8.55E-122 | Macro_SLC40A1 | AKR1A1 |
| HLA-DQA1 | 4.18E-126 | 1.125425114 | 0.975 | 0.691 | 1.30E-121 | Macro_SLC40A1 | HLA-DQA1 |
| CAPG | 8.64E-123 | 0.8791532 | 0.97 | 0.704 | 2.69E-118 | Macro_SLC40A1 | CAPG |
| CTSZ | 3.23E-122 | 0.792489444 | 0.983 | 0.647 | 1.01E-117 | Macro_SLC40A1 | CTSZ |
| SEPP1 | 7.44E-122 | 1.068984565 | 0.508 | 0.152 | 2.31E-117 | Macro_SLC40A1 | SEPP1 |
| MMP12 | 1.28E-120 | 0.938745687 | 0.604 | 0.21 | 3.97E-116 | Macro_SLC40A1 | MMP12 |
| SGPL1 | 2.73E-119 | 0.588243895 | 0.627 | 0.224 | 8.50E-115 | Macro_SLC40A1 | SGPL1 |
| CTSA | 3.14E-119 | 0.783721567 | 0.861 | 0.442 | 9.76E-115 | Macro_SLC40A1 | CTSA |
| CSF1R | 1.49E-118 | 0.708421445 | 0.817 | 0.375 | 4.64E-114 | Macro_SLC40A1 | CSF1R |
| DNASE2 | 2.34E-117 | 0.723933527 | 0.592 | 0.213 | 7.27E-113 | Macro_SLC40A1 | DNASE2 |
| DNPH1 | 4.36E-117 | 0.618862915 | 0.688 | 0.264 | 1.36E-112 | Macro_SLC40A1 | DNPH1 |
| SERPINF1 | 7.63E-117 | 0.645965354 | 0.421 | 0.112 | 2.37E-112 | Macro_SLC40A1 | SERPINF1 |
| SYNGR2 | 4.50E-116 | 0.705864616 | 0.844 | 0.415 | 1.40E-111 | Macro_SLC40A1 | SYNGR2 |
| SCPEP1 | 1.24E-115 | 0.692474774 | 0.753 | 0.323 | 3.87E-111 | Macro_SLC40A1 | SCPEP1 |
| YWHAH | 2.66E-113 | 0.898689119 | 0.879 | 0.478 | 8.29E-109 | Macro_SLC40A1 | YWHAH |
| PLAU | 3.88E-113 | 0.722399737 | 0.687 | 0.27 | 1.21E-108 | Macro_SLC40A1 | PLAU |
| ACP2 | 9.77E-113 | 0.514249793 | 0.559 | 0.181 | 3.04E-108 | Macro_SLC40A1 | ACP2 |
| LGMN | 2.71E-111 | 0.998085087 | 0.863 | 0.459 | 8.43E-107 | Macro_SLC40A1 | LGMN |
| CD68 | 3.50E-109 | 0.747459108 | 0.979 | 0.77 | 1.09E-104 | Macro_SLC40A1 | CD68 |
| VOPP1 | 6.99E-109 | 0.627334064 | 0.694 | 0.284 | 2.18E-104 | Macro_SLC40A1 | VOPP1 |
| KCNMA1 | 7.46E-108 | 0.523523668 | 0.47 | 0.138 | 2.32E-103 | Macro_SLC40A1 | KCNMA1 |
| NPC2 | 5.37E-107 | 0.605943244 | 0.999 | 0.926 | 1.67E-102 | Macro_SLC40A1 | NPC2 |
| PEBP1 | 5.50E-107 | 0.616027681 | 0.766 | 0.342 | 1.71E-102 | Macro_SLC40A1 | PEBP1 |
| PLTP | 5.56E-107 | 0.537244542 | 0.556 | 0.188 | 1.73E-102 | Macro_SLC40A1 | PLTP |
| HLA-DOA | 1.58E-106 | 0.441112027 | 0.467 | 0.138 | 4.91E-102 | Macro_SLC40A1 | HLA-DOA |
| SLAMF8 | 1.60E-106 | 0.531803686 | 0.558 | 0.193 | 4.98E-102 | Macro_SLC40A1 | SLAMF8 |
| HLA-DRB1 | 1.57E-105 | 0.980603789 | 0.996 | 0.904 | 4.90E-101 | Macro_SLC40A1 | HLA-DRB1 |
| RASSF4 | 5.28E-105 | 0.595429908 | 0.669 | 0.267 | 1.64E-100 | Macro_SLC40A1 | RASSF4 |
| RNASE6 | 5.80E-104 | 0.700327986 | 0.817 | 0.397 | 1.80E-99 | Macro_SLC40A1 | RNASE6 |
| CD4 | 1.71E-103 | 0.658265588 | 0.78 | 0.372 | 5.32E-99 | Macro_SLC40A1 | CD4 |
| TNFSF12 | 7.00E-103 | 0.514481048 | 0.48 | 0.152 | 2.18E-98 | Macro_SLC40A1 | TNFSF12 |
| FGL2 | 7.26E-103 | 0.765170292 | 0.799 | 0.4 | 2.26E-98 | Macro_SLC40A1 | FGL2 |
| APOC1 | 7.75E-103 | 1.179015298 | 0.831 | 0.428 | 2.41E-98 | Macro_SLC40A1 | APOC1 |
| LINC00996 | 8.00E-103 | 0.274917356 | 0.178 | 0.019 | 2.49E-98 | Macro_SLC40A1 | LINC00996 |
| GAL3ST4 | 1.48E-99 | 0.435669119 | 0.361 | 0.091 | 4.59E-95 | Macro_SLC40A1 | GAL3ST4 |
| GIMAP4 | 4.71E-99 | 0.479060659 | 0.49 | 0.159 | 1.47E-94 | Macro_SLC40A1 | GIMAP4 |
| DAB2 | 4.76E-99 | 0.56133314 | 0.734 | 0.308 | 1.48E-94 | Macro_SLC40A1 | DAB2 |
| GALM | 5.97E-98 | 0.432270421 | 0.448 | 0.137 | 1.86E-93 | Macro_SLC40A1 | GALM |
| ERP29 | 2.41E-97 | 0.607607742 | 0.819 | 0.404 | 7.51E-93 | Macro_SLC40A1 | ERP29 |
| STAB1 | 5.58E-97 | 0.539810225 | 0.65 | 0.253 | 1.74E-92 | Macro_SLC40A1 | STAB1 |
| ZNF331 | 1.07E-96 | 0.853852425 | 0.63 | 0.27 | 3.33E-92 | Macro_SLC40A1 | ZNF331 |
| OLFML3 | 1.44E-96 | 0.420921103 | 0.249 | 0.045 | 4.49E-92 | Macro_SLC40A1 | OLFML3 |
| MS4A4A | 1.88E-95 | 0.718379809 | 0.781 | 0.382 | 5.84E-91 | Macro_SLC40A1 | MS4A4A |
| PCBD1 | 5.47E-95 | 0.510555606 | 0.653 | 0.264 | 1.70E-90 | Macro_SLC40A1 | PCBD1 |
| ABHD12 | 1.03E-94 | 0.476713106 | 0.585 | 0.216 | 3.22E-90 | Macro_SLC40A1 | ABHD12 |
| MPEG1 | 1.15E-94 | 0.620228639 | 0.719 | 0.311 | 3.58E-90 | Macro_SLC40A1 | MPEG1 |
| CD300A | 7.64E-94 | 0.592856591 | 0.654 | 0.279 | 2.38E-89 | Macro_SLC40A1 | CD300A |
| S100B | 2.21E-93 | 0.738350596 | 0.36 | 0.099 | 6.88E-89 | Macro_SLC40A1 | S100B |
| SIGLEC10 | 3.14E-93 | 0.388521478 | 0.422 | 0.126 | 9.76E-89 | Macro_SLC40A1 | SIGLEC10 |
| TMEM37 | 1.10E-92 | 0.338715776 | 0.32 | 0.076 | 3.43E-88 | Macro_SLC40A1 | TMEM37 |
| DNASE1L3 | 1.94E-92 | 1.019929952 | 0.169 | 0.02 | 6.04E-88 | Macro_SLC40A1 | DNASE1L3 |
| LPAR6 | 4.16E-92 | 0.477792882 | 0.405 | 0.12 | 1.29E-87 | Macro_SLC40A1 | LPAR6 |
| LIPA | 4.60E-92 | 0.652953169 | 0.766 | 0.357 | 1.43E-87 | Macro_SLC40A1 | LIPA |
| FRMD4A | 6.14E-92 | 0.348588311 | 0.304 | 0.071 | 1.91E-87 | Macro_SLC40A1 | FRMD4A |
| IGSF6 | 4.05E-91 | 0.820558165 | 0.798 | 0.435 | 1.26E-86 | Macro_SLC40A1 | IGSF6 |
| CAPZB | 4.76E-91 | 0.647367221 | 0.923 | 0.606 | 1.48E-86 | Macro_SLC40A1 | CAPZB |
| CTSH | 5.12E-91 | 0.718131407 | 0.93 | 0.622 | 1.59E-86 | Macro_SLC40A1 | CTSH |
| CORO1B | 7.86E-91 | 0.556529824 | 0.686 | 0.305 | 2.45E-86 | Macro_SLC40A1 | CORO1B |
| VAMP8 | 8.05E-91 | 0.627612247 | 0.967 | 0.693 | 2.50E-86 | Macro_SLC40A1 | VAMP8 |
| LAIR1 | 1.95E-90 | 0.591323991 | 0.769 | 0.373 | 6.08E-86 | Macro_SLC40A1 | LAIR1 |
| CD81 | 2.04E-90 | 0.630584227 | 0.862 | 0.445 | 6.34E-86 | Macro_SLC40A1 | CD81 |
| ADAM28 | 5.24E-90 | 0.365046354 | 0.366 | 0.099 | 1.63E-85 | Macro_SLC40A1 | ADAM28 |
| AP1B1 | 1.15E-89 | 0.434330119 | 0.551 | 0.201 | 3.59E-85 | Macro_SLC40A1 | AP1B1 |
| RB1 | 3.20E-89 | 0.511280108 | 0.614 | 0.243 | 9.96E-85 | Macro_SLC40A1 | RB1 |
| LAG3 | 4.22E-89 | 0.31595566 | 0.203 | 0.032 | 1.31E-84 | Macro_SLC40A1 | LAG3 |
| NR1H3 | 4.84E-89 | 0.497435078 | 0.376 | 0.109 | 1.51E-84 | Macro_SLC40A1 | NR1H3 |
| TCN2 | 5.91E-89 | 0.491534586 | 0.457 | 0.153 | 1.84E-84 | Macro_SLC40A1 | TCN2 |
| FYB | 7.02E-89 | 0.590868152 | 0.841 | 0.435 | 2.18E-84 | Macro_SLC40A1 | FYB |
| TNFAIP8L2 | 1.99E-88 | 0.381365004 | 0.339 | 0.09 | 6.20E-84 | Macro_SLC40A1 | TNFAIP8L2 |
| CMKLR1 | 6.28E-88 | 0.327815205 | 0.316 | 0.078 | 1.96E-83 | Macro_SLC40A1 | CMKLR1 |
| ARL4C | 1.16E-87 | 0.564538359 | 0.753 | 0.345 | 3.60E-83 | Macro_SLC40A1 | ARL4C |
| NEU1 | 1.68E-87 | 0.482387111 | 0.574 | 0.22 | 5.22E-83 | Macro_SLC40A1 | NEU1 |
| CD84 | 2.00E-87 | 0.458846831 | 0.579 | 0.221 | 6.22E-83 | Macro_SLC40A1 | CD84 |
| ARHGDIB | 2.13E-87 | 0.706099053 | 0.938 | 0.67 | 6.63E-83 | Macro_SLC40A1 | ARHGDIB |
| SDC3 | 2.70E-86 | 0.417955036 | 0.391 | 0.116 | 8.40E-82 | Macro_SLC40A1 | SDC3 |
| SERPING1 | 3.71E-86 | 0.571600492 | 0.533 | 0.202 | 1.15E-81 | Macro_SLC40A1 | SERPING1 |
| IGHA1 | 6.28E-86 | 0.896217121 | 0.643 | 0.284 | 1.95E-81 | Macro_SLC40A1 | IGHA1 |
| TTYH3 | 1.62E-85 | 0.452777263 | 0.654 | 0.272 | 5.04E-81 | Macro_SLC40A1 | TTYH3 |
| SLC2A8 | 3.57E-85 | 0.302681563 | 0.263 | 0.058 | 1.11E-80 | Macro_SLC40A1 | SLC2A8 |
| TNFRSF14 | 6.21E-85 | 0.439280226 | 0.654 | 0.273 | 1.93E-80 | Macro_SLC40A1 | TNFRSF14 |
| CD72 | 7.16E-85 | 0.34173324 | 0.394 | 0.119 | 2.23E-80 | Macro_SLC40A1 | CD72 |
| GIMAP7 | 9.46E-85 | 0.343266684 | 0.235 | 0.046 | 2.94E-80 | Macro_SLC40A1 | GIMAP7 |
| DHRSX | 2.22E-84 | 0.288298045 | 0.361 | 0.101 | 6.92E-80 | Macro_SLC40A1 | DHRSX |
| GM2A | 3.95E-84 | 0.483118844 | 0.68 | 0.291 | 1.23E-79 | Macro_SLC40A1 | GM2A |
| FAM105A | 5.32E-84 | 0.382802256 | 0.427 | 0.138 | 1.65E-79 | Macro_SLC40A1 | FAM105A |
| GSN | 6.66E-84 | 0.614032348 | 0.845 | 0.454 | 2.07E-79 | Macro_SLC40A1 | GSN |
| NAGK | 3.11E-83 | 0.452135887 | 0.574 | 0.227 | 9.68E-79 | Macro_SLC40A1 | NAGK |
| IFITM10 | 1.45E-82 | 0.327848362 | 0.272 | 0.063 | 4.52E-78 | Macro_SLC40A1 | IFITM10 |
| FAM26F | 1.94E-82 | 0.621560405 | 0.794 | 0.41 | 6.03E-78 | Macro_SLC40A1 | FAM26F |
| SCAMP2 | 2.53E-82 | 0.381980696 | 0.542 | 0.203 | 7.86E-78 | Macro_SLC40A1 | SCAMP2 |
| C3AR1 | 2.56E-82 | 0.518084132 | 0.644 | 0.274 | 7.98E-78 | Macro_SLC40A1 | C3AR1 |
| VSIG4 | 4.30E-82 | 0.649991454 | 0.578 | 0.24 | 1.34E-77 | Macro_SLC40A1 | VSIG4 |
| CREG1 | 4.46E-82 | 0.610955274 | 0.901 | 0.545 | 1.39E-77 | Macro_SLC40A1 | CREG1 |
| CST3 | 6.28E-82 | 0.620733333 | 1 | 0.912 | 1.95E-77 | Macro_SLC40A1 | CST3 |
| GATM | 1.30E-81 | 0.350944665 | 0.297 | 0.075 | 4.05E-77 | Macro_SLC40A1 | GATM |
| GPNMB | 1.48E-81 | 0.754974703 | 0.826 | 0.445 | 4.60E-77 | Macro_SLC40A1 | GPNMB |
| HLA-E | 1.72E-81 | 0.573415112 | 0.976 | 0.819 | 5.34E-77 | Macro_SLC40A1 | HLA-E |
| HN1 | 1.73E-81 | 0.543644607 | 0.834 | 0.452 | 5.39E-77 | Macro_SLC40A1 | HN1 |
| KLHDC8B | 4.64E-79 | 0.315672456 | 0.246 | 0.054 | 1.44E-74 | Macro_SLC40A1 | KLHDC8B |
| IDH1 | 1.26E-78 | 0.381313069 | 0.53 | 0.201 | 3.92E-74 | Macro_SLC40A1 | IDH1 |
| GPX1 | 2.94E-78 | 0.527103634 | 0.996 | 0.924 | 9.15E-74 | Macro_SLC40A1 | GPX1 |
| GSTM4 | 3.27E-78 | 0.257404636 | 0.266 | 0.063 | 1.02E-73 | Macro_SLC40A1 | GSTM4 |
| CHCHD10 | 5.82E-78 | 0.515258587 | 0.837 | 0.442 | 1.81E-73 | Macro_SLC40A1 | CHCHD10 |
| GAA | 8.65E-78 | 0.430368801 | 0.542 | 0.215 | 2.69E-73 | Macro_SLC40A1 | GAA |
| TREM2 | 1.48E-77 | 0.691454113 | 0.621 | 0.275 | 4.60E-73 | Macro_SLC40A1 | TREM2 |
| RAB42 | 2.37E-77 | 0.261945299 | 0.197 | 0.035 | 7.38E-73 | Macro_SLC40A1 | RAB42 |
| HLA-A | 3.10E-77 | 0.666248268 | 0.996 | 0.957 | 9.66E-73 | Macro_SLC40A1 | HLA-A |
| GLMP | 7.67E-77 | 0.31939411 | 0.455 | 0.158 | 2.39E-72 | Macro_SLC40A1 | GLMP |
| TMED9 | 2.42E-76 | 0.526923033 | 0.747 | 0.377 | 7.53E-72 | Macro_SLC40A1 | TMED9 |
| P2RY6 | 2.98E-76 | 0.291001702 | 0.283 | 0.072 | 9.29E-72 | Macro_SLC40A1 | P2RY6 |
| HLA-DRA | 1.24E-75 | 0.635966964 | 0.999 | 0.982 | 3.84E-71 | Macro_SLC40A1 | HLA-DRA |
| DAPK1 | 2.74E-75 | 0.356997028 | 0.463 | 0.167 | 8.54E-71 | Macro_SLC40A1 | DAPK1 |
| FAM213A | 2.77E-75 | 0.322292865 | 0.329 | 0.095 | 8.61E-71 | Macro_SLC40A1 | FAM213A |
| NFKBIE | 8.66E-75 | 0.542897403 | 0.629 | 0.284 | 2.69E-70 | Macro_SLC40A1 | NFKBIE |
| ITPR2 | 9.31E-75 | 0.351277347 | 0.468 | 0.168 | 2.90E-70 | Macro_SLC40A1 | ITPR2 |
| ITM2C | 9.89E-75 | 0.386650146 | 0.275 | 0.071 | 3.08E-70 | Macro_SLC40A1 | ITM2C |
| MERTK | 1.52E-74 | 0.31693397 | 0.391 | 0.125 | 4.74E-70 | Macro_SLC40A1 | MERTK |
| SDHD | 1.77E-74 | 0.432869439 | 0.649 | 0.289 | 5.49E-70 | Macro_SLC40A1 | SDHD |
| STMN1 | 3.92E-74 | 0.252216457 | 0.515 | 0.194 | 1.22E-69 | Macro_SLC40A1 | STMN1 |
| UCP2 | 6.97E-74 | 0.597060769 | 0.807 | 0.458 | 2.17E-69 | Macro_SLC40A1 | UCP2 |
| PEPD | 7.06E-74 | 0.349031982 | 0.515 | 0.2 | 2.20E-69 | Macro_SLC40A1 | PEPD |
| SPRED1 | 1.92E-73 | 0.357781018 | 0.465 | 0.169 | 5.98E-69 | Macro_SLC40A1 | SPRED1 |
| CCDC85B | 2.56E-73 | 0.424325274 | 0.624 | 0.274 | 7.96E-69 | Macro_SLC40A1 | CCDC85B |
| NARS | 4.03E-73 | 0.337447891 | 0.489 | 0.183 | 1.25E-68 | Macro_SLC40A1 | NARS |
| HSD17B14 | 4.16E-73 | 0.317269026 | 0.306 | 0.085 | 1.29E-68 | Macro_SLC40A1 | HSD17B14 |
| ATOX1 | 7.18E-73 | 0.691143478 | 0.923 | 0.632 | 2.23E-68 | Macro_SLC40A1 | ATOX1 |
| TEX264 | 7.89E-73 | 0.353896863 | 0.463 | 0.17 | 2.46E-68 | Macro_SLC40A1 | TEX264 |
| APEX1 | 9.42E-73 | 0.381164256 | 0.522 | 0.206 | 2.93E-68 | Macro_SLC40A1 | APEX1 |
| LINC00998 | 1.96E-72 | 0.436054203 | 0.702 | 0.333 | 6.10E-68 | Macro_SLC40A1 | LINC00998 |
| CBR1 | 2.26E-72 | 0.31913365 | 0.43 | 0.15 | 7.04E-68 | Macro_SLC40A1 | CBR1 |
| LILRB4 | 2.35E-72 | 0.626713367 | 0.755 | 0.404 | 7.32E-68 | Macro_SLC40A1 | LILRB4 |
| FCGR1A | 2.76E-72 | 0.520923228 | 0.618 | 0.278 | 8.58E-68 | Macro_SLC40A1 | FCGR1A |
| BST2 | 3.41E-72 | 0.589617299 | 0.869 | 0.531 | 1.06E-67 | Macro_SLC40A1 | BST2 |
| CD14 | 3.57E-72 | 0.9107411 | 0.927 | 0.678 | 1.11E-67 | Macro_SLC40A1 | CD14 |
| CXCL9 | 4.67E-72 | 1.114632844 | 0.314 | 0.093 | 1.45E-67 | Macro_SLC40A1 | CXCL9 |
| CTSB | 6.60E-72 | 0.595429529 | 0.999 | 0.908 | 2.05E-67 | Macro_SLC40A1 | CTSB |
| LAMP1 | 9.30E-72 | 0.526649516 | 0.857 | 0.481 | 2.89E-67 | Macro_SLC40A1 | LAMP1 |
| MRPL34 | 6.64E-71 | 0.320965497 | 0.438 | 0.157 | 2.07E-66 | Macro_SLC40A1 | MRPL34 |
| SLC7A7 | 1.08E-70 | 0.468016387 | 0.755 | 0.389 | 3.37E-66 | Macro_SLC40A1 | SLC7A7 |
| DDRGK1 | 1.34E-70 | 0.367928864 | 0.473 | 0.182 | 4.16E-66 | Macro_SLC40A1 | DDRGK1 |
| TMEM14C | 1.67E-70 | 0.451726001 | 0.768 | 0.394 | 5.18E-66 | Macro_SLC40A1 | TMEM14C |
| IDH2 | 2.18E-70 | 0.409443996 | 0.605 | 0.263 | 6.80E-66 | Macro_SLC40A1 | IDH2 |
| CD38 | 5.81E-70 | 0.321697965 | 0.308 | 0.088 | 1.81E-65 | Macro_SLC40A1 | CD38 |
| MLEC | 1.09E-69 | 0.387667285 | 0.591 | 0.251 | 3.39E-65 | Macro_SLC40A1 | MLEC |
| LAMP2 | 1.18E-69 | 0.496677022 | 0.743 | 0.38 | 3.68E-65 | Macro_SLC40A1 | LAMP2 |
| GUSB | 1.37E-69 | 0.366745928 | 0.504 | 0.201 | 4.25E-65 | Macro_SLC40A1 | GUSB |
| C20orf27 | 1.62E-69 | 0.361250457 | 0.518 | 0.206 | 5.05E-65 | Macro_SLC40A1 | C20orf27 |
| TMED3 | 1.95E-69 | 0.339171047 | 0.51 | 0.202 | 6.08E-65 | Macro_SLC40A1 | TMED3 |
| MFSD1 | 2.19E-69 | 0.431382613 | 0.811 | 0.422 | 6.83E-65 | Macro_SLC40A1 | MFSD1 |
| UNC93B1 | 2.37E-69 | 0.356067664 | 0.508 | 0.202 | 7.37E-65 | Macro_SLC40A1 | UNC93B1 |
| ATP13A2 | 2.68E-69 | 0.260032154 | 0.199 | 0.04 | 8.33E-65 | Macro_SLC40A1 | ATP13A2 |
| LDHB | 3.94E-69 | 0.495788852 | 0.697 | 0.344 | 1.23E-64 | Macro_SLC40A1 | LDHB |
| RGS10 | 4.42E-69 | 0.529501661 | 0.917 | 0.612 | 1.37E-64 | Macro_SLC40A1 | RGS10 |
| PMVK | 4.97E-69 | 0.315059875 | 0.486 | 0.187 | 1.55E-64 | Macro_SLC40A1 | PMVK |
| AKAP9 | 5.13E-69 | 0.319491655 | 0.43 | 0.155 | 1.60E-64 | Macro_SLC40A1 | AKAP9 |
| ARL2 | 2.96E-68 | 0.329033455 | 0.526 | 0.21 | 9.21E-64 | Macro_SLC40A1 | ARL2 |
| MDH1 | 7.53E-68 | 0.390743556 | 0.578 | 0.248 | 2.34E-63 | Macro_SLC40A1 | MDH1 |
| FRMD4B | 1.13E-67 | 0.308292314 | 0.414 | 0.146 | 3.53E-63 | Macro_SLC40A1 | FRMD4B |
| RARRES1 | 1.86E-67 | 0.288376711 | 0.188 | 0.037 | 5.80E-63 | Macro_SLC40A1 | RARRES1 |
| PPT1 | 2.13E-67 | 0.506781745 | 0.886 | 0.531 | 6.64E-63 | Macro_SLC40A1 | PPT1 |
| EPB41L2 | 3.45E-67 | 0.301061448 | 0.322 | 0.098 | 1.07E-62 | Macro_SLC40A1 | EPB41L2 |
| APMAP | 5.60E-67 | 0.33870146 | 0.471 | 0.183 | 1.74E-62 | Macro_SLC40A1 | APMAP |
| HVCN1 | 1.67E-66 | 0.269217268 | 0.308 | 0.092 | 5.18E-62 | Macro_SLC40A1 | HVCN1 |
| OLFML2B | 2.53E-66 | 0.270629817 | 0.366 | 0.121 | 7.88E-62 | Macro_SLC40A1 | OLFML2B |
| IGF1 | 1.15E-65 | 0.28543172 | 0.141 | 0.021 | 3.57E-61 | Macro_SLC40A1 | IGF1 |
| RGS12 | 2.46E-65 | 0.279757793 | 0.234 | 0.058 | 7.67E-61 | Macro_SLC40A1 | RGS12 |
| CCT3 | 2.83E-65 | 0.342154582 | 0.551 | 0.235 | 8.79E-61 | Macro_SLC40A1 | CCT3 |
| HERPUD1 | 3.98E-65 | 0.589051702 | 0.807 | 0.47 | 1.24E-60 | Macro_SLC40A1 | HERPUD1 |
| LRPAP1 | 5.08E-65 | 0.441230201 | 0.711 | 0.364 | 1.58E-60 | Macro_SLC40A1 | LRPAP1 |
| TPD52 | 5.85E-65 | 0.260757998 | 0.276 | 0.078 | 1.82E-60 | Macro_SLC40A1 | TPD52 |
| FMNL2 | 1.02E-64 | 0.306093203 | 0.394 | 0.139 | 3.18E-60 | Macro_SLC40A1 | FMNL2 |
| ISCU | 1.26E-64 | 0.421354793 | 0.705 | 0.351 | 3.91E-60 | Macro_SLC40A1 | ISCU |
| TBXAS1 | 1.64E-64 | 0.402499302 | 0.564 | 0.251 | 5.11E-60 | Macro_SLC40A1 | TBXAS1 |
| PSMB5 | 1.92E-64 | 0.329337722 | 0.508 | 0.207 | 5.99E-60 | Macro_SLC40A1 | PSMB5 |
| EMP2 | 2.58E-64 | 0.311682913 | 0.239 | 0.061 | 8.04E-60 | Macro_SLC40A1 | EMP2 |
| DPP7 | 2.63E-64 | 0.425861152 | 0.779 | 0.419 | 8.17E-60 | Macro_SLC40A1 | DPP7 |
| ASAH1 | 5.71E-64 | 0.578966326 | 0.942 | 0.709 | 1.78E-59 | Macro_SLC40A1 | ASAH1 |
| CEBPD | 8.92E-64 | 0.569851905 | 0.64 | 0.317 | 2.77E-59 | Macro_SLC40A1 | CEBPD |
| EVA1B | 1.07E-63 | 0.252455311 | 0.256 | 0.068 | 3.34E-59 | Macro_SLC40A1 | EVA1B |
| ECHS1 | 2.77E-63 | 0.340233274 | 0.571 | 0.25 | 8.62E-59 | Macro_SLC40A1 | ECHS1 |
| MIF4GD | 5.33E-63 | 0.282338266 | 0.373 | 0.131 | 1.66E-58 | Macro_SLC40A1 | MIF4GD |
| NCF1 | 5.41E-63 | 0.318140377 | 0.535 | 0.221 | 1.68E-58 | Macro_SLC40A1 | NCF1 |
| NECAP2 | 6.08E-63 | 0.344412363 | 0.512 | 0.212 | 1.89E-58 | Macro_SLC40A1 | NECAP2 |
| LACC1 | 6.75E-63 | 0.284926913 | 0.287 | 0.084 | 2.10E-58 | Macro_SLC40A1 | LACC1 |
| SCARB2 | 6.92E-63 | 0.369070986 | 0.655 | 0.306 | 2.15E-58 | Macro_SLC40A1 | SCARB2 |
| RPN2 | 7.00E-63 | 0.447691349 | 0.704 | 0.358 | 2.18E-58 | Macro_SLC40A1 | RPN2 |
| NCF4 | 7.35E-63 | 0.321163727 | 0.49 | 0.196 | 2.29E-58 | Macro_SLC40A1 | NCF4 |
| MKNK1 | 8.41E-63 | 0.334884242 | 0.435 | 0.168 | 2.62E-58 | Macro_SLC40A1 | MKNK1 |
| NUDT14 | 4.52E-62 | 0.289631164 | 0.466 | 0.184 | 1.41E-57 | Macro_SLC40A1 | NUDT14 |
| RHOB | 5.32E-62 | 0.791791273 | 0.71 | 0.386 | 1.66E-57 | Macro_SLC40A1 | RHOB |
| PARP1 | 1.29E-61 | 0.299472656 | 0.39 | 0.141 | 4.02E-57 | Macro_SLC40A1 | PARP1 |
| DNMT1 | 2.46E-61 | 0.311697038 | 0.421 | 0.161 | 7.66E-57 | Macro_SLC40A1 | DNMT1 |
| RENBP | 2.50E-61 | 0.340554728 | 0.49 | 0.202 | 7.79E-57 | Macro_SLC40A1 | RENBP |
| STAT1 | 2.72E-61 | 0.592814624 | 0.699 | 0.379 | 8.47E-57 | Macro_SLC40A1 | STAT1 |
| DOK2 | 4.07E-61 | 0.399350086 | 0.591 | 0.271 | 1.27E-56 | Macro_SLC40A1 | DOK2 |
| MMP14 | 5.00E-61 | 0.338416632 | 0.746 | 0.365 | 1.56E-56 | Macro_SLC40A1 | MMP14 |
| CLN8 | 5.09E-61 | 0.335402534 | 0.459 | 0.182 | 1.59E-56 | Macro_SLC40A1 | CLN8 |
| LAPTM5 | 6.19E-61 | 0.495282777 | 0.988 | 0.871 | 1.93E-56 | Macro_SLC40A1 | LAPTM5 |
| PLEKHO1 | 6.41E-61 | 0.308464957 | 0.528 | 0.224 | 1.99E-56 | Macro_SLC40A1 | PLEKHO1 |
| AOAH | 7.81E-61 | 0.326967429 | 0.409 | 0.154 | 2.43E-56 | Macro_SLC40A1 | AOAH |
| SNX6 | 1.78E-60 | 0.355349926 | 0.63 | 0.297 | 5.55E-56 | Macro_SLC40A1 | SNX6 |
| NAIP | 3.70E-60 | 0.288374888 | 0.464 | 0.183 | 1.15E-55 | Macro_SLC40A1 | NAIP |
| SPINT2 | 4.65E-60 | 0.387004802 | 0.809 | 0.425 | 1.45E-55 | Macro_SLC40A1 | SPINT2 |
| COMT | 6.72E-60 | 0.420456798 | 0.75 | 0.4 | 2.09E-55 | Macro_SLC40A1 | COMT |
| TECR | 8.29E-60 | 0.345233405 | 0.491 | 0.208 | 2.58E-55 | Macro_SLC40A1 | TECR |
| MMP9 | 1.11E-59 | 0.623736979 | 0.674 | 0.355 | 3.45E-55 | Macro_SLC40A1 | MMP9 |
| AP2A2 | 1.91E-59 | 0.25462009 | 0.37 | 0.13 | 5.95E-55 | Macro_SLC40A1 | AP2A2 |
| CD209 | 2.06E-59 | 0.284774596 | 0.291 | 0.09 | 6.39E-55 | Macro_SLC40A1 | CD209 |
| SEMA4A | 2.12E-59 | 0.298146529 | 0.408 | 0.154 | 6.61E-55 | Macro_SLC40A1 | SEMA4A |
| SCIMP | 2.36E-59 | 0.254732428 | 0.356 | 0.123 | 7.35E-55 | Macro_SLC40A1 | SCIMP |
| ENTPD1 | 3.78E-59 | 0.296534695 | 0.404 | 0.151 | 1.18E-54 | Macro_SLC40A1 | ENTPD1 |
| ADI1 | 4.11E-59 | 0.315656828 | 0.498 | 0.212 | 1.28E-54 | Macro_SLC40A1 | ADI1 |
| CMTM3 | 5.51E-59 | 0.335920463 | 0.56 | 0.249 | 1.71E-54 | Macro_SLC40A1 | CMTM3 |
| PLBD1 | 9.18E-59 | 0.298471576 | 0.497 | 0.208 | 2.86E-54 | Macro_SLC40A1 | PLBD1 |
| NAA20 | 1.02E-58 | 0.308565132 | 0.516 | 0.22 | 3.17E-54 | Macro_SLC40A1 | NAA20 |
| BMP2K | 1.40E-58 | 0.323032812 | 0.442 | 0.177 | 4.35E-54 | Macro_SLC40A1 | BMP2K |
| DDOST | 1.95E-58 | 0.338093375 | 0.507 | 0.219 | 6.08E-54 | Macro_SLC40A1 | DDOST |
| NDUFS3 | 2.92E-58 | 0.257990624 | 0.471 | 0.193 | 9.07E-54 | Macro_SLC40A1 | NDUFS3 |
| PLA2G7 | 3.42E-58 | 0.550486694 | 0.703 | 0.382 | 1.06E-53 | Macro_SLC40A1 | PLA2G7 |
| CTSS | 3.43E-58 | 0.527302844 | 0.985 | 0.866 | 1.07E-53 | Macro_SLC40A1 | CTSS |
| TMSB4X | 4.35E-58 | 0.401244184 | 1 | 1 | 1.35E-53 | Macro_SLC40A1 | TMSB4X |
| NDFIP1 | 4.69E-58 | 0.333655204 | 0.725 | 0.369 | 1.46E-53 | Macro_SLC40A1 | NDFIP1 |
| THEMIS2 | 5.01E-58 | 0.351036205 | 0.625 | 0.301 | 1.56E-53 | Macro_SLC40A1 | THEMIS2 |
| ITGB2 | 5.94E-58 | 0.493356448 | 0.954 | 0.663 | 1.85E-53 | Macro_SLC40A1 | ITGB2 |
| SMPDL3A | 8.08E-58 | 0.304204345 | 0.354 | 0.125 | 2.51E-53 | Macro_SLC40A1 | SMPDL3A |
| DBNL | 9.64E-58 | 0.355799339 | 0.678 | 0.339 | 3.00E-53 | Macro_SLC40A1 | DBNL |
| DHRS3 | 1.08E-57 | 0.258869916 | 0.347 | 0.121 | 3.38E-53 | Macro_SLC40A1 | DHRS3 |
| TCF4 | 1.16E-57 | 0.317882152 | 0.377 | 0.14 | 3.60E-53 | Macro_SLC40A1 | TCF4 |
| COMMD9 | 1.43E-57 | 0.269757759 | 0.451 | 0.179 | 4.44E-53 | Macro_SLC40A1 | COMMD9 |
| LGALS9 | 1.76E-57 | 0.394051445 | 0.755 | 0.402 | 5.47E-53 | Macro_SLC40A1 | LGALS9 |
| CYB561A3 | 1.78E-57 | 0.281359212 | 0.396 | 0.148 | 5.54E-53 | Macro_SLC40A1 | CYB561A3 |
| GLA | 2.01E-57 | 0.455034314 | 0.605 | 0.287 | 6.24E-53 | Macro_SLC40A1 | GLA |
| NCKAP1L | 2.49E-57 | 0.311266991 | 0.522 | 0.229 | 7.73E-53 | Macro_SLC40A1 | NCKAP1L |
| ST14 | 3.71E-57 | 0.326181814 | 0.439 | 0.178 | 1.16E-52 | Macro_SLC40A1 | ST14 |
| GNPDA1 | 5.03E-57 | 0.274709074 | 0.405 | 0.155 | 1.57E-52 | Macro_SLC40A1 | GNPDA1 |
| TM2D2 | 5.36E-57 | 0.263535289 | 0.361 | 0.13 | 1.67E-52 | Macro_SLC40A1 | TM2D2 |
| LAMTOR1 | 6.05E-57 | 0.441605931 | 0.804 | 0.473 | 1.88E-52 | Macro_SLC40A1 | LAMTOR1 |
| NUPR1 | 6.82E-57 | 0.447868464 | 0.514 | 0.235 | 2.12E-52 | Macro_SLC40A1 | NUPR1 |
| CCND1 | 8.17E-57 | 0.254279239 | 0.283 | 0.088 | 2.54E-52 | Macro_SLC40A1 | CCND1 |
| VCP | 1.87E-56 | 0.311264448 | 0.524 | 0.229 | 5.83E-52 | Macro_SLC40A1 | VCP |
| SNX2 | 2.13E-56 | 0.345385523 | 0.609 | 0.291 | 6.63E-52 | Macro_SLC40A1 | SNX2 |
| SDSL | 3.14E-56 | 0.284930834 | 0.521 | 0.226 | 9.78E-52 | Macro_SLC40A1 | SDSL |
| CD40 | 4.49E-56 | 0.468870821 | 0.589 | 0.28 | 1.40E-51 | Macro_SLC40A1 | CD40 |
| PSMC2 | 5.03E-56 | 0.3113787 | 0.442 | 0.182 | 1.56E-51 | Macro_SLC40A1 | PSMC2 |
| SDHB | 6.49E-56 | 0.36147822 | 0.592 | 0.284 | 2.02E-51 | Macro_SLC40A1 | SDHB |
| LRRC25 | 7.04E-56 | 0.293643213 | 0.44 | 0.177 | 2.19E-51 | Macro_SLC40A1 | LRRC25 |
| TOR3A | 7.04E-56 | 0.30473831 | 0.347 | 0.126 | 2.19E-51 | Macro_SLC40A1 | TOR3A |
| GADD45G | 8.28E-56 | 0.560040389 | 0.384 | 0.149 | 2.58E-51 | Macro_SLC40A1 | GADD45G |
| ARHGAP18 | 9.03E-56 | 0.314689566 | 0.64 | 0.305 | 2.81E-51 | Macro_SLC40A1 | ARHGAP18 |
| NPL | 1.05E-55 | 0.325720852 | 0.53 | 0.234 | 3.26E-51 | Macro_SLC40A1 | NPL |
| LY86 | 1.13E-55 | 0.401835741 | 0.672 | 0.338 | 3.52E-51 | Macro_SLC40A1 | LY86 |
| ATP5A1 | 1.22E-55 | 0.425602473 | 0.679 | 0.356 | 3.81E-51 | Macro_SLC40A1 | ATP5A1 |
| UFC1 | 1.33E-55 | 0.328141292 | 0.662 | 0.326 | 4.12E-51 | Macro_SLC40A1 | UFC1 |
| SMIM7 | 1.44E-55 | 0.253721203 | 0.464 | 0.189 | 4.47E-51 | Macro_SLC40A1 | SMIM7 |
| TNFSF13B | 1.46E-55 | 0.432792977 | 0.822 | 0.469 | 4.56E-51 | Macro_SLC40A1 | TNFSF13B |
| MSR1 | 1.53E-55 | 0.264585824 | 0.673 | 0.318 | 4.77E-51 | Macro_SLC40A1 | MSR1 |
| NAGA | 2.00E-55 | 0.269713305 | 0.489 | 0.207 | 6.23E-51 | Macro_SLC40A1 | NAGA |
| JCHAIN | 2.15E-55 | 0.605433292 | 0.347 | 0.126 | 6.69E-51 | Macro_SLC40A1 | JCHAIN |
| 1-Mar | 2.48E-55 | 0.282456922 | 0.451 | 0.184 | 7.71E-51 | Macro_SLC40A1 | 1-Mar |
| NDUFB5 | 4.43E-55 | 0.381407756 | 0.725 | 0.387 | 1.38E-50 | Macro_SLC40A1 | NDUFB5 |
| SIL1 | 4.46E-55 | 0.253391328 | 0.392 | 0.149 | 1.39E-50 | Macro_SLC40A1 | SIL1 |
| ZFP36L2 | 5.23E-55 | 0.415681775 | 0.693 | 0.364 | 1.63E-50 | Macro_SLC40A1 | ZFP36L2 |
| SNX5 | 1.75E-54 | 0.281081222 | 0.507 | 0.218 | 5.46E-50 | Macro_SLC40A1 | SNX5 |
| CREBL2 | 1.91E-54 | 0.274682064 | 0.493 | 0.213 | 5.94E-50 | Macro_SLC40A1 | CREBL2 |
| HLA-C | 2.54E-54 | 0.517778663 | 0.99 | 0.93 | 7.90E-50 | Macro_SLC40A1 | HLA-C |
| ARPC1B | 5.23E-54 | 0.482694893 | 0.957 | 0.732 | 1.63E-49 | Macro_SLC40A1 | ARPC1B |
| NUCB1 | 8.78E-54 | 0.376357531 | 0.69 | 0.358 | 2.73E-49 | Macro_SLC40A1 | NUCB1 |
| RAP2B | 9.62E-54 | 0.268338605 | 0.499 | 0.212 | 2.99E-49 | Macro_SLC40A1 | RAP2B |
| ZDHHC12 | 9.86E-54 | 0.269933437 | 0.454 | 0.189 | 3.07E-49 | Macro_SLC40A1 | ZDHHC12 |
| FUCA2 | 1.12E-53 | 0.264891417 | 0.466 | 0.195 | 3.49E-49 | Macro_SLC40A1 | FUCA2 |
| FAM110A | 1.24E-53 | 0.294083806 | 0.303 | 0.103 | 3.85E-49 | Macro_SLC40A1 | FAM110A |
| ATRAID | 1.51E-53 | 0.345603506 | 0.636 | 0.311 | 4.69E-49 | Macro_SLC40A1 | ATRAID |
| CD59 | 1.84E-53 | 0.402742871 | 0.742 | 0.412 | 5.74E-49 | Macro_SLC40A1 | CD59 |
| OS9 | 2.40E-53 | 0.330587467 | 0.621 | 0.304 | 7.47E-49 | Macro_SLC40A1 | OS9 |
| NUDT3 | 2.74E-53 | 0.286893376 | 0.479 | 0.206 | 8.52E-49 | Macro_SLC40A1 | NUDT3 |
| RGS19 | 2.97E-53 | 0.329546891 | 0.505 | 0.227 | 9.23E-49 | Macro_SLC40A1 | RGS19 |
| SDS | 4.42E-53 | 0.396787316 | 0.574 | 0.274 | 1.37E-48 | Macro_SLC40A1 | SDS |
| HMOX1 | 9.04E-53 | 0.318459666 | 0.678 | 0.347 | 2.81E-48 | Macro_SLC40A1 | HMOX1 |
| SNHG12 | 9.04E-53 | 0.320155078 | 0.297 | 0.101 | 2.81E-48 | Macro_SLC40A1 | SNHG12 |
| BRI3 | 1.01E-52 | 0.44097859 | 0.856 | 0.527 | 3.14E-48 | Macro_SLC40A1 | BRI3 |
| ARL6IP5 | 1.07E-52 | 0.456500756 | 0.81 | 0.504 | 3.33E-48 | Macro_SLC40A1 | ARL6IP5 |
| PLXNC1 | 1.24E-52 | 0.266469298 | 0.442 | 0.182 | 3.85E-48 | Macro_SLC40A1 | PLXNC1 |
| NAAA | 1.27E-52 | 0.259992436 | 0.439 | 0.18 | 3.96E-48 | Macro_SLC40A1 | NAAA |
| ARL5A | 1.30E-52 | 0.299055605 | 0.547 | 0.251 | 4.05E-48 | Macro_SLC40A1 | ARL5A |
| KDELR1 | 2.08E-52 | 0.303596559 | 0.583 | 0.274 | 6.46E-48 | Macro_SLC40A1 | KDELR1 |
| MYDGF | 2.51E-52 | 0.433770078 | 0.818 | 0.497 | 7.82E-48 | Macro_SLC40A1 | MYDGF |
| IGLC3 | 2.67E-52 | 1.139718697 | 0.486 | 0.219 | 8.30E-48 | Macro_SLC40A1 | IGLC3 |
| FOLR2 | 3.30E-52 | 0.439622984 | 0.364 | 0.141 | 1.03E-47 | Macro_SLC40A1 | FOLR2 |
| TFPT | 7.89E-52 | 0.299154841 | 0.549 | 0.254 | 2.46E-47 | Macro_SLC40A1 | TFPT |
| MPC1 | 8.50E-52 | 0.29548282 | 0.599 | 0.29 | 2.64E-47 | Macro_SLC40A1 | MPC1 |
| TIMP2 | 1.20E-51 | 0.306707191 | 0.746 | 0.38 | 3.73E-47 | Macro_SLC40A1 | TIMP2 |
| NENF | 1.33E-51 | 0.291702938 | 0.659 | 0.325 | 4.13E-47 | Macro_SLC40A1 | NENF |
| RNASET2 | 1.75E-51 | 0.463966677 | 0.923 | 0.662 | 5.43E-47 | Macro_SLC40A1 | RNASET2 |
| ABCG1 | 2.34E-51 | 0.257524179 | 0.358 | 0.134 | 7.29E-47 | Macro_SLC40A1 | ABCG1 |
| IQGAP2 | 5.04E-51 | 0.297114264 | 0.49 | 0.215 | 1.57E-46 | Macro_SLC40A1 | IQGAP2 |
| BLVRA | 5.67E-51 | 0.32054605 | 0.592 | 0.288 | 1.76E-46 | Macro_SLC40A1 | BLVRA |
| PRCP | 7.75E-51 | 0.284281358 | 0.533 | 0.243 | 2.41E-46 | Macro_SLC40A1 | PRCP |
| WASF2 | 2.21E-50 | 0.281551899 | 0.564 | 0.265 | 6.88E-46 | Macro_SLC40A1 | WASF2 |
| ECH1 | 2.45E-50 | 0.285816068 | 0.53 | 0.246 | 7.61E-46 | Macro_SLC40A1 | ECH1 |
| OTOA | 3.22E-50 | 0.296986917 | 0.345 | 0.13 | 1.00E-45 | Macro_SLC40A1 | OTOA |
| APH1A | 3.33E-50 | 0.298955241 | 0.633 | 0.313 | 1.04E-45 | Macro_SLC40A1 | APH1A |
| HEXB | 3.40E-50 | 0.411424244 | 0.83 | 0.489 | 1.06E-45 | Macro_SLC40A1 | HEXB |
| FCGR3A | 3.81E-50 | 0.567971856 | 0.835 | 0.53 | 1.19E-45 | Macro_SLC40A1 | FCGR3A |
| HNMT | 3.92E-50 | 0.326046539 | 0.742 | 0.392 | 1.22E-45 | Macro_SLC40A1 | HNMT |
| SLAMF7 | 5.96E-50 | 0.286891189 | 0.388 | 0.155 | 1.85E-45 | Macro_SLC40A1 | SLAMF7 |
| PIGT | 8.35E-50 | 0.250055065 | 0.397 | 0.161 | 2.60E-45 | Macro_SLC40A1 | PIGT |
| ATP2C1 | 1.65E-49 | 0.269282964 | 0.512 | 0.229 | 5.14E-45 | Macro_SLC40A1 | ATP2C1 |
| TWF2 | 3.32E-49 | 0.335626179 | 0.623 | 0.314 | 1.03E-44 | Macro_SLC40A1 | TWF2 |
| ITGB7 | 4.76E-49 | 0.334115331 | 0.279 | 0.098 | 1.48E-44 | Macro_SLC40A1 | ITGB7 |
| SLC25A5 | 1.04E-48 | 0.463362333 | 0.904 | 0.623 | 3.23E-44 | Macro_SLC40A1 | SLC25A5 |
| RARRES3 | 1.41E-48 | 0.26547247 | 0.476 | 0.21 | 4.38E-44 | Macro_SLC40A1 | RARRES3 |
| C6orf48 | 2.19E-48 | 0.355755464 | 0.573 | 0.288 | 6.81E-44 | Macro_SLC40A1 | C6orf48 |
| UQCC2 | 2.49E-48 | 0.271060319 | 0.509 | 0.232 | 7.74E-44 | Macro_SLC40A1 | UQCC2 |
| RNF130 | 3.26E-48 | 0.405075678 | 0.891 | 0.579 | 1.01E-43 | Macro_SLC40A1 | RNF130 |
| GNB4 | 4.10E-48 | 0.255384535 | 0.505 | 0.231 | 1.28E-43 | Macro_SLC40A1 | GNB4 |
| SMIM4 | 4.37E-48 | 0.270476781 | 0.447 | 0.193 | 1.36E-43 | Macro_SLC40A1 | SMIM4 |
| LY96 | 4.71E-48 | 0.366115106 | 0.864 | 0.542 | 1.46E-43 | Macro_SLC40A1 | LY96 |
| IFI16 | 5.11E-48 | 0.264326484 | 0.559 | 0.263 | 1.59E-43 | Macro_SLC40A1 | IFI16 |
| SLC25A39 | 9.61E-48 | 0.293833708 | 0.573 | 0.282 | 2.99E-43 | Macro_SLC40A1 | SLC25A39 |
| MYO1F | 1.75E-47 | 0.26307836 | 0.52 | 0.242 | 5.44E-43 | Macro_SLC40A1 | MYO1F |
| ALKBH7 | 1.87E-47 | 0.275826482 | 0.617 | 0.311 | 5.80E-43 | Macro_SLC40A1 | ALKBH7 |
| RPS19BP1 | 2.42E-47 | 0.260757336 | 0.678 | 0.344 | 7.52E-43 | Macro_SLC40A1 | RPS19BP1 |
| ID3 | 2.77E-47 | 0.585010944 | 0.377 | 0.158 | 8.62E-43 | Macro_SLC40A1 | ID3 |
| SERPINH1 | 3.69E-47 | 0.335124773 | 0.264 | 0.089 | 1.15E-42 | Macro_SLC40A1 | SERPINH1 |
| MGST3 | 3.74E-47 | 0.367589312 | 0.806 | 0.479 | 1.16E-42 | Macro_SLC40A1 | MGST3 |
| SCP2 | 5.00E-47 | 0.315026909 | 0.712 | 0.385 | 1.56E-42 | Macro_SLC40A1 | SCP2 |
| RHOG | 5.33E-47 | 0.395637431 | 0.83 | 0.528 | 1.66E-42 | Macro_SLC40A1 | RHOG |
| TFDP2 | 6.09E-47 | 0.260558696 | 0.225 | 0.068 | 1.90E-42 | Macro_SLC40A1 | TFDP2 |
| TMEM147 | 8.75E-47 | 0.316280217 | 0.742 | 0.405 | 2.72E-42 | Macro_SLC40A1 | TMEM147 |
| CAMK1 | 1.08E-46 | 0.316784355 | 0.426 | 0.188 | 3.35E-42 | Macro_SLC40A1 | CAMK1 |
| NRP1 | 2.29E-46 | 0.275807092 | 0.536 | 0.255 | 7.12E-42 | Macro_SLC40A1 | NRP1 |
| CYBB | 2.93E-46 | 0.387097039 | 0.86 | 0.539 | 9.11E-42 | Macro_SLC40A1 | CYBB |
| NDRG2 | 6.85E-46 | 0.27851283 | 0.243 | 0.079 | 2.13E-41 | Macro_SLC40A1 | NDRG2 |
| JUNB | 7.19E-46 | 0.620736909 | 0.902 | 0.68 | 2.24E-41 | Macro_SLC40A1 | JUNB |
| IL18BP | 1.14E-45 | 0.261696297 | 0.285 | 0.102 | 3.54E-41 | Macro_SLC40A1 | IL18BP |
| MRPS7 | 1.21E-45 | 0.255226324 | 0.451 | 0.203 | 3.76E-41 | Macro_SLC40A1 | MRPS7 |
| TXN2 | 1.93E-45 | 0.269262579 | 0.527 | 0.252 | 6.01E-41 | Macro_SLC40A1 | TXN2 |
| SLC39A1 | 1.94E-45 | 0.284156556 | 0.515 | 0.245 | 6.05E-41 | Macro_SLC40A1 | SLC39A1 |
| TFRC | 2.39E-45 | 0.487819795 | 0.61 | 0.328 | 7.43E-41 | Macro_SLC40A1 | TFRC |
| C10orf54 | 2.53E-45 | 0.363559663 | 0.693 | 0.379 | 7.86E-41 | Macro_SLC40A1 | C10orf54 |
| RGS2 | 2.91E-45 | 0.56263176 | 0.835 | 0.557 | 9.05E-41 | Macro_SLC40A1 | RGS2 |
| SEC11C | 3.46E-45 | 0.25310257 | 0.498 | 0.233 | 1.08E-40 | Macro_SLC40A1 | SEC11C |
| CYFIP1 | 7.12E-45 | 0.312588814 | 0.508 | 0.243 | 2.21E-40 | Macro_SLC40A1 | CYFIP1 |
| MRPS34 | 1.23E-44 | 0.25077924 | 0.477 | 0.221 | 3.84E-40 | Macro_SLC40A1 | MRPS34 |
| SNRPC | 1.42E-44 | 0.261807506 | 0.534 | 0.257 | 4.43E-40 | Macro_SLC40A1 | SNRPC |
| PPIB | 2.16E-44 | 0.46214696 | 0.883 | 0.609 | 6.72E-40 | Macro_SLC40A1 | PPIB |
| ISOC2 | 2.45E-44 | 0.25957484 | 0.402 | 0.173 | 7.63E-40 | Macro_SLC40A1 | ISOC2 |
| PHB | 4.02E-44 | 0.250492461 | 0.536 | 0.257 | 1.25E-39 | Macro_SLC40A1 | PHB |
| MTDH | 6.67E-44 | 0.352958635 | 0.825 | 0.499 | 2.07E-39 | Macro_SLC40A1 | MTDH |
| TUFM | 7.65E-44 | 0.302697838 | 0.653 | 0.343 | 2.38E-39 | Macro_SLC40A1 | TUFM |
| ATPIF1 | 8.49E-44 | 0.296599037 | 0.709 | 0.381 | 2.64E-39 | Macro_SLC40A1 | ATPIF1 |
| MPC2 | 8.70E-44 | 0.264960173 | 0.547 | 0.265 | 2.71E-39 | Macro_SLC40A1 | MPC2 |
| LMAN2 | 9.52E-44 | 0.335787534 | 0.703 | 0.397 | 2.96E-39 | Macro_SLC40A1 | LMAN2 |
| HS3ST1 | 1.25E-43 | 0.276301254 | 0.319 | 0.124 | 3.90E-39 | Macro_SLC40A1 | HS3ST1 |
| M6PR | 1.76E-43 | 0.333447719 | 0.772 | 0.45 | 5.47E-39 | Macro_SLC40A1 | M6PR |
| HES1 | 1.87E-43 | 0.595036035 | 0.369 | 0.162 | 5.82E-39 | Macro_SLC40A1 | HES1 |
| DRAM2 | 2.09E-43 | 0.288115924 | 0.623 | 0.324 | 6.51E-39 | Macro_SLC40A1 | DRAM2 |
| GPR137B | 2.35E-43 | 0.252625764 | 0.612 | 0.311 | 7.33E-39 | Macro_SLC40A1 | GPR137B |
| FKBP2 | 2.73E-43 | 0.339353734 | 0.788 | 0.472 | 8.48E-39 | Macro_SLC40A1 | FKBP2 |
| ATP5C1 | 2.94E-43 | 0.363420108 | 0.787 | 0.468 | 9.14E-39 | Macro_SLC40A1 | ATP5C1 |
| MRPL57 | 3.30E-43 | 0.252962702 | 0.596 | 0.3 | 1.03E-38 | Macro_SLC40A1 | MRPL57 |
| TBCB | 4.63E-43 | 0.272201678 | 0.571 | 0.285 | 1.44E-38 | Macro_SLC40A1 | TBCB |
| CALR | 4.84E-43 | 0.435465258 | 0.937 | 0.689 | 1.51E-38 | Macro_SLC40A1 | CALR |
| UQCR10 | 6.90E-43 | 0.381781061 | 0.892 | 0.589 | 2.15E-38 | Macro_SLC40A1 | UQCR10 |
| ATP6AP1 | 7.22E-43 | 0.330506207 | 0.757 | 0.442 | 2.25E-38 | Macro_SLC40A1 | ATP6AP1 |
| UBE2L6 | 9.12E-43 | 0.264450599 | 0.624 | 0.322 | 2.84E-38 | Macro_SLC40A1 | UBE2L6 |
| SCD | 1.24E-42 | 0.257063168 | 0.363 | 0.153 | 3.87E-38 | Macro_SLC40A1 | SCD |
| TMED10 | 4.46E-42 | 0.31017992 | 0.787 | 0.463 | 1.39E-37 | Macro_SLC40A1 | TMED10 |
| COX14 | 5.33E-42 | 0.267462972 | 0.669 | 0.355 | 1.66E-37 | Macro_SLC40A1 | COX14 |
| CLTA | 6.64E-42 | 0.386638195 | 0.822 | 0.533 | 2.07E-37 | Macro_SLC40A1 | CLTA |
| C1QBP | 7.37E-42 | 0.300215461 | 0.604 | 0.311 | 2.29E-37 | Macro_SLC40A1 | C1QBP |
| ATP6AP2 | 1.25E-41 | 0.360919096 | 0.859 | 0.564 | 3.89E-37 | Macro_SLC40A1 | ATP6AP2 |
| PARK7 | 1.42E-41 | 0.369564108 | 0.828 | 0.525 | 4.41E-37 | Macro_SLC40A1 | PARK7 |
| RNH1 | 1.84E-41 | 0.382944625 | 0.759 | 0.462 | 5.72E-37 | Macro_SLC40A1 | RNH1 |
| LMNA | 3.20E-41 | 0.527838244 | 0.779 | 0.507 | 9.94E-37 | Macro_SLC40A1 | LMNA |
| PRMT9 | 4.05E-41 | 0.26715661 | 0.317 | 0.127 | 1.26E-36 | Macro_SLC40A1 | PRMT9 |
| NDUFC2 | 4.05E-41 | 0.314855868 | 0.779 | 0.454 | 1.26E-36 | Macro_SLC40A1 | NDUFC2 |
| AP2M1 | 6.04E-41 | 0.325263298 | 0.741 | 0.43 | 1.88E-36 | Macro_SLC40A1 | AP2M1 |
| TUBA1B | 6.99E-41 | 0.395092738 | 0.881 | 0.59 | 2.17E-36 | Macro_SLC40A1 | TUBA1B |
| HAVCR2 | 7.03E-41 | 0.26346269 | 0.549 | 0.276 | 2.19E-36 | Macro_SLC40A1 | HAVCR2 |
| COX6C | 1.34E-40 | 0.364460404 | 0.941 | 0.686 | 4.16E-36 | Macro_SLC40A1 | COX6C |
| CNPY3 | 1.53E-40 | 0.282768266 | 0.688 | 0.38 | 4.76E-36 | Macro_SLC40A1 | CNPY3 |
| PYCARD | 1.68E-40 | 0.36375832 | 0.85 | 0.555 | 5.24E-36 | Macro_SLC40A1 | PYCARD |
| COX5A | 3.80E-40 | 0.383675782 | 0.843 | 0.554 | 1.18E-35 | Macro_SLC40A1 | COX5A |
| NANS | 4.26E-40 | 0.283751105 | 0.629 | 0.335 | 1.33E-35 | Macro_SLC40A1 | NANS |
| ATP5G3 | 4.41E-40 | 0.384934359 | 0.895 | 0.63 | 1.37E-35 | Macro_SLC40A1 | ATP5G3 |
| ATP6V0E1 | 4.57E-40 | 0.366243638 | 0.979 | 0.829 | 1.42E-35 | Macro_SLC40A1 | ATP6V0E1 |
| NDUFA12 | 7.95E-40 | 0.25030043 | 0.635 | 0.329 | 2.47E-35 | Macro_SLC40A1 | NDUFA12 |
| NR4A2 | 1.38E-39 | 0.551134904 | 0.749 | 0.498 | 4.30E-35 | Macro_SLC40A1 | NR4A2 |
| SEPW1 | 1.39E-39 | 0.265816505 | 0.683 | 0.374 | 4.32E-35 | Macro_SLC40A1 | SEPW1 |
| IGHG1 | 3.34E-39 | 1.224317205 | 0.457 | 0.222 | 1.04E-34 | Macro_SLC40A1 | IGHG1 |
| VIMP | 4.03E-39 | 0.301324003 | 0.647 | 0.357 | 1.25E-34 | Macro_SLC40A1 | VIMP |
| EID1 | 7.43E-39 | 0.274306574 | 0.702 | 0.392 | 2.31E-34 | Macro_SLC40A1 | EID1 |
| SAMHD1 | 8.37E-39 | 0.318221456 | 0.766 | 0.45 | 2.60E-34 | Macro_SLC40A1 | SAMHD1 |
| RAB20 | 1.06E-38 | 0.282597316 | 0.623 | 0.335 | 3.31E-34 | Macro_SLC40A1 | RAB20 |
| GPR183 | 1.36E-38 | 0.581349768 | 0.86 | 0.638 | 4.23E-34 | Macro_SLC40A1 | GPR183 |
| IER5 | 1.74E-38 | 0.561399029 | 0.627 | 0.359 | 5.42E-34 | Macro_SLC40A1 | IER5 |
| CYC1 | 1.81E-38 | 0.281933677 | 0.63 | 0.342 | 5.63E-34 | Macro_SLC40A1 | CYC1 |
| HINT1 | 1.93E-38 | 0.367351959 | 0.93 | 0.699 | 6.01E-34 | Macro_SLC40A1 | HINT1 |
| FERMT3 | 2.20E-38 | 0.267885578 | 0.609 | 0.323 | 6.85E-34 | Macro_SLC40A1 | FERMT3 |
| BANF1 | 2.53E-38 | 0.290938621 | 0.68 | 0.379 | 7.87E-34 | Macro_SLC40A1 | BANF1 |
| NRP2 | 2.89E-38 | 0.273226945 | 0.564 | 0.291 | 9.00E-34 | Macro_SLC40A1 | NRP2 |
| REEP5 | 3.30E-38 | 0.314393892 | 0.751 | 0.448 | 1.03E-33 | Macro_SLC40A1 | REEP5 |
| HSPB11 | 3.49E-38 | 0.489779513 | 0.882 | 0.635 | 1.09E-33 | Macro_SLC40A1 | HSPB1 |
| GPX3 | 6.22E-38 | 0.270096242 | 0.415 | 0.194 | 1.94E-33 | Macro_SLC40A1 | GPX3 |
| NDUFB10 | 7.17E-38 | 0.257528398 | 0.719 | 0.407 | 2.23E-33 | Macro_SLC40A1 | NDUFB10 |
| NDUFS8 | 8.99E-38 | 0.254961092 | 0.717 | 0.404 | 2.80E-33 | Macro_SLC40A1 | NDUFS8 |
| SOD1 | 1.12E-37 | 0.308288065 | 0.825 | 0.52 | 3.50E-33 | Macro_SLC40A1 | SOD1 |
| B2M | 1.33E-37 | 0.32349074 | 1 | 1 | 4.14E-33 | Macro_SLC40A1 | B2M |
| NDUFS7 | 1.43E-36 | 0.286413947 | 0.759 | 0.445 | 4.45E-32 | Macro_SLC40A1 | NDUFS7 |
| IL2RA | 2.10E-36 | 0.317062098 | 0.252 | 0.097 | 6.53E-32 | Macro_SLC40A1 | IL2RA |
| OAZ2 | 2.84E-36 | 0.255468214 | 0.738 | 0.43 | 8.83E-32 | Macro_SLC40A1 | OAZ2 |
| TMEM219 | 8.73E-36 | 0.266251031 | 0.782 | 0.46 | 2.72E-31 | Macro_SLC40A1 | TMEM219 |
| HSPA8 | 1.50E-35 | 0.36505431 | 0.891 | 0.636 | 4.68E-31 | Macro_SLC40A1 | HSPA8 |
| BLVRB | 1.78E-35 | 0.295182122 | 0.773 | 0.467 | 5.55E-31 | Macro_SLC40A1 | BLVRB |
| CD9 | 2.24E-35 | 0.252700285 | 0.784 | 0.476 | 6.96E-31 | Macro_SLC40A1 | CD9 |
| SGK1 | 3.31E-35 | 0.364520575 | 0.854 | 0.595 | 1.03E-30 | Macro_SLC40A1 | SGK1 |
| GNAI2 | 3.73E-35 | 0.327658304 | 0.829 | 0.529 | 1.16E-30 | Macro_SLC40A1 | GNAI2 |
| SDHC | 6.15E-35 | 0.258829248 | 0.551 | 0.296 | 1.91E-30 | Macro_SLC40A1 | SDHC |
| HSP90AB1 | 8.73E-35 | 0.343581517 | 0.952 | 0.736 | 2.72E-30 | Macro_SLC40A1 | HSP90AB1 |
| TMBIM6 | 9.49E-35 | 0.352591409 | 0.945 | 0.752 | 2.95E-30 | Macro_SLC40A1 | TMBIM6 |
| BCAP31 | 1.54E-34 | 0.288097715 | 0.785 | 0.487 | 4.79E-30 | Macro_SLC40A1 | BCAP31 |
| GSTK1 | 1.70E-34 | 0.273995875 | 0.707 | 0.421 | 5.29E-30 | Macro_SLC40A1 | GSTK1 |
| IGKC | 2.61E-34 | 0.974648322 | 0.919 | 0.738 | 8.12E-30 | Macro_SLC40A1 | IGKC |
| CYP27A1 | 3.19E-34 | 0.28456572 | 0.254 | 0.101 | 9.91E-30 | Macro_SLC40A1 | CYP27A1 |
| HSPA1B | 3.94E-34 | 0.289075112 | 0.63 | 0.398 | 1.23E-29 | Macro_SLC40A1 | HSPA1B |
| PDIA4 | 4.31E-34 | 0.270367246 | 0.591 | 0.322 | 1.34E-29 | Macro_SLC40A1 | PDIA4 |
| IER2 | 5.14E-34 | 0.461795941 | 0.773 | 0.51 | 1.60E-29 | Macro_SLC40A1 | IER2 |
| SRSF7 | 1.67E-33 | 0.294741703 | 0.58 | 0.327 | 5.20E-29 | Macro_SLC40A1 | SRSF7 |
| PSMA5 | 3.25E-33 | 0.255747788 | 0.547 | 0.298 | 1.01E-28 | Macro_SLC40A1 | PSMA5 |
| JUN | 4.05E-33 | 0.805627375 | 0.825 | 0.609 | 1.26E-28 | Macro_SLC40A1 | JUN |
| FAM46A | 4.75E-33 | 0.311757724 | 0.289 | 0.124 | 1.48E-28 | Macro_SLC40A1 | FAM46A |
| LAP3 | 5.57E-33 | 0.278656759 | 0.702 | 0.423 | 1.73E-28 | Macro_SLC40A1 | LAP3 |
| P4HB | 7.19E-33 | 0.314818654 | 0.849 | 0.564 | 2.24E-28 | Macro_SLC40A1 | P4HB |
| H2AFJ | 8.31E-33 | 0.25441016 | 0.734 | 0.444 | 2.59E-28 | Macro_SLC40A1 | H2AFJ |
| NDUFB8 | 1.17E-32 | 0.299794312 | 0.804 | 0.515 | 3.63E-28 | Macro_SLC40A1 | NDUFB8 |
| BAG31 | 4.89E-32 | 0.639544787 | 0.371 | 0.188 | 1.52E-27 | Macro_SLC40A1 | BAG3 |
| SPI1 | 5.30E-32 | 0.324435066 | 0.892 | 0.636 | 1.65E-27 | Macro_SLC40A1 | SPI1 |
| LAPTM4A | 6.43E-32 | 0.298867244 | 0.836 | 0.563 | 2.00E-27 | Macro_SLC40A1 | LAPTM4A |
| IFRD1 | 7.52E-32 | 0.321533095 | 0.515 | 0.277 | 2.34E-27 | Macro_SLC40A1 | IFRD1 |
| CANX | 8.38E-32 | 0.294749468 | 0.812 | 0.516 | 2.61E-27 | Macro_SLC40A1 | CANX |
| LAMTOR2 | 1.15E-31 | 0.260485748 | 0.817 | 0.503 | 3.57E-27 | Macro_SLC40A1 | LAMTOR2 |
| PSMB8 | 1.70E-31 | 0.25586629 | 0.643 | 0.38 | 5.30E-27 | Macro_SLC40A1 | PSMB8 |
| MS4A7 | 1.85E-31 | 0.341425397 | 0.862 | 0.606 | 5.75E-27 | Macro_SLC40A1 | MS4A7 |
| OSTF1 | 2.16E-31 | 0.270664134 | 0.694 | 0.421 | 6.73E-27 | Macro_SLC40A1 | OSTF1 |
| RAC1 | 3.07E-31 | 0.307839211 | 0.97 | 0.834 | 9.54E-27 | Macro_SLC40A1 | RAC1 |
| MAFB | 6.68E-31 | 0.532938989 | 0.718 | 0.464 | 2.08E-26 | Macro_SLC40A1 | MAFB |
| PTGES3 | 1.31E-30 | 0.256672811 | 0.786 | 0.494 | 4.09E-26 | Macro_SLC40A1 | PTGES3 |
| NKG7 | 2.83E-30 | 0.296936671 | 0.307 | 0.138 | 8.79E-26 | Macro_SLC40A1 | NKG7 |
| EIF4A2 | 3.65E-30 | 0.29828116 | 0.696 | 0.431 | 1.14E-25 | Macro_SLC40A1 | EIF4A2 |
| CALM2 | 1.01E-29 | 0.326363942 | 0.932 | 0.739 | 3.15E-25 | Macro_SLC40A1 | CALM2 |
| HES4 | 3.19E-29 | 0.282939043 | 0.484 | 0.269 | 9.91E-25 | Macro_SLC40A1 | HES4 |
| PSME2 | 3.37E-29 | 0.297590782 | 0.882 | 0.623 | 1.05E-24 | Macro_SLC40A1 | PSME2 |
| IL32 | 1.32E-28 | 0.383368101 | 0.448 | 0.248 | 4.11E-24 | Macro_SLC40A1 | IL32 |
| ATP5B | 1.54E-28 | 0.306922733 | 0.801 | 0.545 | 4.81E-24 | Macro_SLC40A1 | ATP5B |
| IGLC2 | 2.68E-28 | 2.07362595 | 0.693 | 0.437 | 8.35E-24 | Macro_SLC40A1 | IGLC2 |
| UBC | 4.44E-28 | 0.339504086 | 0.981 | 0.877 | 1.38E-23 | Macro_SLC40A1 | UBC |
| ATF3 | 7.26E-28 | 0.364184849 | 0.576 | 0.355 | 2.26E-23 | Macro_SLC40A1 | ATF3 |
| IL4I1 | 7.30E-28 | 0.327732971 | 0.52 | 0.299 | 2.27E-23 | Macro_SLC40A1 | IL4I1 |
| DNAJB1 | 9.86E-28 | 0.281258862 | 0.734 | 0.499 | 3.07E-23 | Macro_SLC40A1 | DNAJB1 |
| AIF1 | 1.84E-27 | 0.271352141 | 0.983 | 0.891 | 5.73E-23 | Macro_SLC40A1 | AIF1 |
| CXCL16 | 1.99E-26 | 0.257205029 | 0.803 | 0.539 | 6.21E-22 | Macro_SLC40A1 | CXCL16 |
| HSP90B1 | 3.97E-26 | 0.333591324 | 0.907 | 0.671 | 1.23E-21 | Macro_SLC40A1 | HSP90B1 |
| EIF4A3 | 6.13E-26 | 0.310567061 | 0.661 | 0.408 | 1.91E-21 | Macro_SLC40A1 | EIF4A3 |
| TSC22D1 | 9.03E-25 | 0.317529815 | 0.562 | 0.341 | 2.81E-20 | Macro_SLC40A1 | TSC22D1 |
| ATP5J2 | 2.66E-24 | 0.264890187 | 0.876 | 0.627 | 8.28E-20 | Macro_SLC40A1 | ATP5J2 |
| TMEM59 | 8.66E-24 | 0.270271698 | 0.837 | 0.594 | 2.69E-19 | Macro_SLC40A1 | TMEM59 |
| CKS2 | 1.81E-23 | 0.288215539 | 0.578 | 0.354 | 5.63E-19 | Macro_SLC40A1 | CKS2 |
| GSTP1 | 3.52E-23 | 0.259662438 | 0.951 | 0.776 | 1.10E-18 | Macro_SLC40A1 | GSTP1 |
| DNAJA4 | 3.87E-23 | 0.425560695 | 0.303 | 0.157 | 1.20E-18 | Macro_SLC40A1 | DNAJA4 |
| PDIA6 | 6.44E-23 | 0.261029185 | 0.757 | 0.502 | 2.00E-18 | Macro_SLC40A1 | PDIA6 |
| ZFAND5 | 1.07E-22 | 0.279801821 | 0.82 | 0.572 | 3.34E-18 | Macro_SLC40A1 | ZFAND5 |
| KLF2 | 3.81E-22 | 0.441932852 | 0.345 | 0.19 | 1.19E-17 | Macro_SLC40A1 | KLF2 |
| IFI27 | 4.53E-22 | 0.78116161 | 0.524 | 0.341 | 1.41E-17 | Macro_SLC40A1 | IFI27 |
| IGHG4 | 8.14E-22 | 0.664259467 | 0.669 | 0.424 | 2.53E-17 | Macro_SLC40A1 | IGHG4 |
| RPSA | 1.27E-21 | 0.292914702 | 0.963 | 0.81 | 3.94E-17 | Macro_SLC40A1 | RPSA |
| LGALS3 | 1.78E-21 | 0.263182551 | 0.942 | 0.755 | 5.54E-17 | Macro_SLC40A1 | LGALS3 |
| PPIA | 1.88E-21 | 0.264028284 | 0.975 | 0.845 | 5.84E-17 | Macro_SLC40A1 | PPIA |
| NAP1L1 | 3.83E-21 | 0.260024565 | 0.85 | 0.629 | 1.19E-16 | Macro_SLC40A1 | NAP1L1 |
| HLA-DQA2 | 4.02E-21 | 0.321426307 | 0.555 | 0.354 | 1.25E-16 | Macro_SLC40A1 | HLA-DQA2 |
| NDUFA4 | 1.48E-19 | 0.260108851 | 0.93 | 0.729 | 4.62E-15 | Macro_SLC40A1 | NDUFA4 |
| CCL18 | 2.47E-19 | 1.078696718 | 0.36 | 0.214 | 7.69E-15 | Macro_SLC40A1 | CCL18 |
| DUSP2 | 2.66E-19 | 0.356105428 | 0.679 | 0.496 | 8.29E-15 | Macro_SLC40A1 | DUSP2 |
| HLA-DRB5 | 8.75E-19 | 0.437216651 | 0.791 | 0.668 | 2.72E-14 | Macro_SLC40A1 | HLA-DRB5 |
| HAMP | 4.82E-17 | 0.276567951 | 0.126 | 0.051 | 1.50E-12 | Macro_SLC40A1 | HAMP |
| IGHG3 | 2.12E-16 | 1.083760721 | 0.577 | 0.372 | 6.58E-12 | Macro_SLC40A1 | IGHG3 |
| LYZ1 | 2.49E-13 | 0.417115096 | 0.988 | 0.927 | 7.76E-09 | Macro_SLC40A1 | LYZ |
| KCNQ1OT1 | 2.79E-13 | 0.281627557 | 0.229 | 0.128 | 8.67E-09 | Macro_SLC40A1 | KCNQ1OT1 |
| ZFAND2A | 2.59E-12 | 0.442586164 | 0.353 | 0.232 | 8.05E-08 | Macro_SLC40A1 | ZFAND2A |
| APOC11 | 0 | 1.720159118 | 0.794 | 0.323 | 0 | Macro_SPP1 | APOC1 |
| CTSD1 | 0 | 1.461464669 | 0.97 | 0.7 | 0 | Macro_SPP1 | CTSD |
| APOE1 | 0 | 1.435281709 | 0.837 | 0.347 | 0 | Macro_SPP1 | APOE |
| FTL | 0 | 1.095205096 | 1 | 1 | 0 | Macro_SPP1 | FTL |
| GPNMB1 | 1.53E-306 | 1.473451404 | 0.795 | 0.344 | 4.77E-302 | Macro_SPP1 | GPNMB |
| RNASE1 | 5.76E-278 | 2.205089457 | 0.637 | 0.211 | 1.79E-273 | Macro_SPP1 | RNASE1 |
| CTSB1 | 2.16E-256 | 1.034781232 | 0.982 | 0.888 | 6.71E-252 | Macro_SPP1 | CTSB |
| SPP1 | 4.71E-234 | 2.15166401 | 0.728 | 0.334 | 1.46E-229 | Macro_SPP1 | SPP1 |
| MSR11 | 4.09E-214 | 1.266380116 | 0.604 | 0.244 | 1.27E-209 | Macro_SPP1 | MSR1 |
| CD91 | 3.15E-210 | 1.209025735 | 0.748 | 0.399 | 9.79E-206 | Macro_SPP1 | CD9 |
| CTSZ1 | 1.16E-191 | 1.063124092 | 0.861 | 0.607 | 3.61E-187 | Macro_SPP1 | CTSZ |
| C1QA1 | 6.69E-171 | 0.864607239 | 0.825 | 0.403 | 2.08E-166 | Macro_SPP1 | C1QA |
| TREM21 | 1.61E-166 | 1.061131847 | 0.539 | 0.211 | 5.00E-162 | Macro_SPP1 | TREM2 |
| NUPR11 | 2.94E-164 | 1.724868584 | 0.476 | 0.169 | 9.14E-160 | Macro_SPP1 | NUPR1 |
| CCL181 | 4.47E-148 | 1.806002265 | 0.42 | 0.138 | 1.39E-143 | Macro_SPP1 | CCL18 |
| C1QB1 | 1.79E-145 | 0.722775586 | 0.786 | 0.38 | 5.57E-141 | Macro_SPP1 | C1QB |
| DAB21 | 3.18E-145 | 0.974436805 | 0.563 | 0.265 | 9.89E-141 | Macro_SPP1 | DAB2 |
| C1QC1 | 1.38E-142 | 0.706991965 | 0.753 | 0.355 | 4.30E-138 | Macro_SPP1 | C1QC |
| CD811 | 3.46E-135 | 0.81861609 | 0.69 | 0.405 | 1.08E-130 | Macro_SPP1 | CD81 |
| LGMN1 | 1.53E-133 | 0.981813229 | 0.694 | 0.421 | 4.76E-129 | Macro_SPP1 | LGMN |
| TIMP21 | 4.73E-133 | 0.847101747 | 0.614 | 0.336 | 1.47E-128 | Macro_SPP1 | TIMP2 |
| SLCO2B11 | 5.94E-130 | 0.85607466 | 0.484 | 0.208 | 1.85E-125 | Macro_SPP1 | SLCO2B1 |
| FABP5 | 2.44E-129 | 1.127516585 | 0.745 | 0.515 | 7.60E-125 | Macro_SPP1 | FABP5 |
| PLD31 | 1.52E-128 | 0.811739626 | 0.665 | 0.357 | 4.74E-124 | Macro_SPP1 | PLD3 |
| GPX11 | 9.96E-128 | 0.584517613 | 0.977 | 0.911 | 3.10E-123 | Macro_SPP1 | GPX1 |
| NGFRAP1 | 9.63E-125 | 0.935002296 | 0.425 | 0.166 | 3.00E-120 | Macro_SPP1 | NGFRAP1 |
| LIPA1 | 1.27E-114 | 0.886546803 | 0.588 | 0.323 | 3.94E-110 | Macro_SPP1 | LIPA |
| A2M1 | 4.72E-111 | 0.754985806 | 0.462 | 0.2 | 1.47E-106 | Macro_SPP1 | A2M |
| PSAP1 | 1.05E-108 | 0.508276586 | 0.977 | 0.93 | 3.27E-104 | Macro_SPP1 | PSAP |
| FCGR3A1 | 3.06E-108 | 0.769558465 | 0.745 | 0.482 | 9.54E-104 | Macro_SPP1 | FCGR3A |
| CCL13 | 1.41E-106 | 1.246811347 | 0.221 | 0.044 | 4.39E-102 | Macro_SPP1 | CCL13 |
| LAMP11 | 1.16E-98 | 0.752048901 | 0.678 | 0.457 | 3.62E-94 | Macro_SPP1 | LAMP1 |
| GCHFR | 4.03E-94 | 0.970427741 | 0.379 | 0.165 | 1.25E-89 | Macro_SPP1 | GCHFR |
| CD163 | 4.20E-94 | 0.792227291 | 0.567 | 0.32 | 1.31E-89 | Macro_SPP1 | CD163 |
| MRC1 | 3.17E-93 | 0.759745697 | 0.515 | 0.269 | 9.87E-89 | Macro_SPP1 | MRC1 |
| GLUL | 5.05E-91 | 0.587511327 | 0.92 | 0.812 | 1.57E-86 | Macro_SPP1 | GLUL |
| FPR3 | 7.39E-91 | 0.769623504 | 0.589 | 0.361 | 2.30E-86 | Macro_SPP1 | FPR3 |
| VAT1 | 2.69E-90 | 0.561432567 | 0.308 | 0.111 | 8.38E-86 | Macro_SPP1 | VAT1 |
| ACP51 | 2.88E-89 | 0.833495949 | 0.693 | 0.451 | 8.96E-85 | Macro_SPP1 | ACP5 |
| GPX31 | 1.37E-88 | 0.654465571 | 0.365 | 0.152 | 4.26E-84 | Macro_SPP1 | GPX3 |
| PLTP1 | 2.26E-87 | 0.865027055 | 0.379 | 0.166 | 7.03E-83 | Macro_SPP1 | PLTP |
| GM2A1 | 3.59E-80 | 0.672865703 | 0.487 | 0.271 | 1.12E-75 | Macro_SPP1 | GM2A |
| NPC21 | 1.93E-78 | 0.430254736 | 0.97 | 0.918 | 6.00E-74 | Macro_SPP1 | NPC2 |
| MS4A4A1 | 5.87E-77 | 0.703427103 | 0.585 | 0.361 | 1.83E-72 | Macro_SPP1 | MS4A4A |
| COLEC12 | 4.42E-76 | 0.445240489 | 0.163 | 0.033 | 1.38E-71 | Macro_SPP1 | COLEC12 |
| SEPP11 | 6.23E-76 | 1.363395269 | 0.327 | 0.135 | 1.94E-71 | Macro_SPP1 | SEPP1 |
| CAPG1 | 1.26E-75 | 0.566256301 | 0.835 | 0.691 | 3.91E-71 | Macro_SPP1 | CAPG |
| CD681 | 2.16E-75 | 0.568455524 | 0.852 | 0.771 | 6.73E-71 | Macro_SPP1 | CD68 |
| NPL1 | 3.03E-72 | 0.542803715 | 0.41 | 0.205 | 9.42E-68 | Macro_SPP1 | NPL |
| MAF | 4.65E-72 | 0.709263164 | 0.356 | 0.166 | 1.45E-67 | Macro_SPP1 | MAF |
| MFSD11 | 1.53E-70 | 0.61108896 | 0.604 | 0.409 | 4.75E-66 | Macro_SPP1 | MFSD1 |
| MMP141 | 2.75E-70 | 0.693715532 | 0.55 | 0.348 | 8.55E-66 | Macro_SPP1 | MMP14 |
| MMP121 | 2.29E-68 | 1.764094629 | 0.389 | 0.199 | 7.12E-64 | Macro_SPP1 | MMP12 |
| OTOA1 | 7.00E-68 | 0.51135133 | 0.268 | 0.103 | 2.18E-63 | Macro_SPP1 | OTOA |
| ASAH11 | 1.33E-67 | 0.535204508 | 0.829 | 0.696 | 4.14E-63 | Macro_SPP1 | ASAH1 |
| CD841 | 3.51E-67 | 0.562340097 | 0.396 | 0.205 | 1.09E-62 | Macro_SPP1 | CD84 |
| NRP11 | 2.41E-66 | 0.581052834 | 0.421 | 0.228 | 7.49E-62 | Macro_SPP1 | NRP1 |
| LAPTM51 | 5.45E-65 | 0.446782508 | 0.917 | 0.872 | 1.70E-60 | Macro_SPP1 | LAPTM5 |
| FOLR21 | 1.88E-64 | 0.852937738 | 0.279 | 0.116 | 5.85E-60 | Macro_SPP1 | FOLR2 |
| VAMP81 | 4.49E-64 | 0.462824508 | 0.856 | 0.665 | 1.40E-59 | Macro_SPP1 | VAMP8 |
| CREG11 | 2.00E-63 | 0.496392502 | 0.718 | 0.529 | 6.21E-59 | Macro_SPP1 | CREG1 |
| ADAM9 | 4.06E-61 | 0.544956278 | 0.449 | 0.262 | 1.26E-56 | Macro_SPP1 | ADAM9 |
| RCAN1 | 5.87E-61 | 0.746613356 | 0.287 | 0.126 | 1.83E-56 | Macro_SPP1 | RCAN1 |
| MGST31 | 1.88E-59 | 0.559375678 | 0.639 | 0.464 | 5.86E-55 | Macro_SPP1 | MGST3 |
| CD591 | 2.49E-59 | 0.552219889 | 0.583 | 0.392 | 7.76E-55 | Macro_SPP1 | CD59 |
| CTSL | 1.14E-58 | 0.674250887 | 0.844 | 0.721 | 3.55E-54 | Macro_SPP1 | CTSL |
| KCNMA11 | 5.31E-58 | 0.526666684 | 0.288 | 0.129 | 1.65E-53 | Macro_SPP1 | KCNMA1 |
| HEXB1 | 8.67E-58 | 0.584844189 | 0.632 | 0.486 | 2.70E-53 | Macro_SPP1 | HEXB |
| LHFPL2 | 8.63E-56 | 0.526937244 | 0.379 | 0.205 | 2.69E-51 | Macro_SPP1 | LHFPL2 |
| SLC7A8 | 1.10E-55 | 0.431299184 | 0.202 | 0.072 | 3.41E-51 | Macro_SPP1 | SLC7A8 |
| RNF1301 | 1.60E-55 | 0.488824732 | 0.721 | 0.571 | 4.98E-51 | Macro_SPP1 | RNF130 |
| ITM2B1 | 4.56E-55 | 0.457778318 | 0.913 | 0.838 | 1.42E-50 | Macro_SPP1 | ITM2B |
| ATOX11 | 4.74E-55 | 0.459274891 | 0.777 | 0.617 | 1.47E-50 | Macro_SPP1 | ATOX1 |
| GAPLINC | 8.11E-55 | 0.35427934 | 0.155 | 0.043 | 2.52E-50 | Macro_SPP1 | GAPLINC |
| MMP91 | 1.09E-54 | 1.124368689 | 0.512 | 0.34 | 3.38E-50 | Macro_SPP1 | MMP9 |
| GRN1 | 1.09E-54 | 0.428461893 | 0.863 | 0.738 | 3.39E-50 | Macro_SPP1 | GRN |
| RP11-1008C21.1 | 1.76E-54 | 0.370633527 | 0.151 | 0.041 | 5.48E-50 | Macro_SPP1 | RP11-1008C21.1 |
| TSC22D11 | 9.59E-54 | 0.627878246 | 0.492 | 0.309 | 2.98E-49 | Macro_SPP1 | TSC22D1 |
| CTSC1 | 1.07E-53 | 0.359256038 | 0.785 | 0.6 | 3.34E-49 | Macro_SPP1 | CTSC |
| FABP3 | 2.66E-53 | 0.390380697 | 0.167 | 0.051 | 8.29E-49 | Macro_SPP1 | FABP3 |
| RPS27L | 9.32E-53 | 0.541188634 | 0.765 | 0.646 | 2.90E-48 | Macro_SPP1 | RPS27L |
| SPRED11 | 4.65E-52 | 0.494906279 | 0.311 | 0.157 | 1.45E-47 | Macro_SPP1 | SPRED1 |
| GSN1 | 5.69E-52 | 0.536731316 | 0.632 | 0.443 | 1.77E-47 | Macro_SPP1 | GSN |
| ARID5B | 3.94E-51 | 0.532490221 | 0.333 | 0.174 | 1.22E-46 | Macro_SPP1 | ARID5B |
| CCL2 | 1.10E-50 | 0.819349415 | 0.47 | 0.287 | 3.41E-46 | Macro_SPP1 | CCL2 |
| MATK | 1.26E-50 | 0.337347454 | 0.162 | 0.05 | 3.93E-46 | Macro_SPP1 | MATK |
| LAMP21 | 4.25E-50 | 0.484894932 | 0.545 | 0.371 | 1.32E-45 | Macro_SPP1 | LAMP2 |
| SDC2 | 9.48E-50 | 0.550991276 | 0.399 | 0.224 | 2.95E-45 | Macro_SPP1 | SDC2 |
| ARHGAP181 | 2.45E-49 | 0.499402752 | 0.461 | 0.294 | 7.62E-45 | Macro_SPP1 | ARHGAP18 |
| MT-ND4 | 1.19E-48 | 0.348185542 | 0.993 | 0.997 | 3.72E-44 | Macro_SPP1 | MT-ND4 |
| TNS3 | 2.11E-48 | 0.447587848 | 0.277 | 0.133 | 6.58E-44 | Macro_SPP1 | TNS3 |
| TTYH31 | 2.71E-48 | 0.55549694 | 0.43 | 0.27 | 8.43E-44 | Macro_SPP1 | TTYH3 |
| STAB11 | 3.11E-48 | 0.652880014 | 0.413 | 0.252 | 9.67E-44 | Macro_SPP1 | STAB1 |
| LY961 | 3.48E-48 | 0.507854439 | 0.686 | 0.535 | 1.08E-43 | Macro_SPP1 | LY96 |
| HSD3B7 | 6.64E-48 | 0.351956489 | 0.227 | 0.095 | 2.07E-43 | Macro_SPP1 | HSD3B7 |
| KCTD12 | 7.49E-48 | 0.482718358 | 0.551 | 0.369 | 2.33E-43 | Macro_SPP1 | KCTD12 |
| LPL | 9.02E-48 | 0.368472771 | 0.11 | 0.023 | 2.81E-43 | Macro_SPP1 | LPL |
| CXCL161 | 9.71E-48 | 0.491120393 | 0.669 | 0.527 | 3.02E-43 | Macro_SPP1 | CXCL16 |
| VMO1 | 1.44E-47 | 0.656563754 | 0.359 | 0.201 | 4.49E-43 | Macro_SPP1 | VMO1 |
| SLC16A10 | 6.29E-47 | 0.539287299 | 0.473 | 0.301 | 1.96E-42 | Macro_SPP1 | SLC16A10 |
| CSTB | 6.72E-47 | 0.429136503 | 0.918 | 0.867 | 2.09E-42 | Macro_SPP1 | CSTB |
| HCFC1R1 | 2.56E-46 | 0.488118288 | 0.373 | 0.221 | 7.98E-42 | Macro_SPP1 | HCFC1R1 |
| SCD1 | 3.77E-46 | 0.514060918 | 0.274 | 0.134 | 1.17E-41 | Macro_SPP1 | SCD |
| LMNA1 | 4.90E-46 | 0.509148345 | 0.665 | 0.482 | 1.52E-41 | Macro_SPP1 | LMNA |
| ACP21 | 2.67E-45 | 0.422761675 | 0.332 | 0.181 | 8.31E-41 | Macro_SPP1 | ACP2 |
| GPR341 | 8.13E-45 | 0.512883239 | 0.295 | 0.152 | 2.53E-40 | Macro_SPP1 | GPR34 |
| PLAU1 | 2.10E-44 | 0.602754595 | 0.435 | 0.271 | 6.53E-40 | Macro_SPP1 | PLAU |
| TDP2 | 3.22E-44 | 0.386483566 | 0.238 | 0.108 | 1.00E-39 | Macro_SPP1 | TDP2 |
| IDH11 | 3.63E-44 | 0.455742732 | 0.344 | 0.196 | 1.13E-39 | Macro_SPP1 | IDH1 |
| RGS11 | 2.12E-43 | 0.453631923 | 0.742 | 0.549 | 6.59E-39 | Macro_SPP1 | RGS1 |
| FN1 | 6.92E-43 | 0.860442557 | 0.284 | 0.143 | 2.15E-38 | Macro_SPP1 | FN1 |
| MT-ND5 | 1.26E-42 | 0.41737968 | 0.931 | 0.916 | 3.91E-38 | Macro_SPP1 | MT-ND5 |
| C4orf48 | 1.94E-42 | 0.53378099 | 0.623 | 0.482 | 6.05E-38 | Macro_SPP1 | C4orf48 |
| SDC31 | 5.70E-42 | 0.424223138 | 0.236 | 0.111 | 1.77E-37 | Macro_SPP1 | SDC3 |
| TFRC1 | 6.61E-42 | 0.552964238 | 0.472 | 0.312 | 2.06E-37 | Macro_SPP1 | TFRC |
| DYNLL1 | 1.25E-41 | 0.417168066 | 0.806 | 0.711 | 3.89E-37 | Macro_SPP1 | DYNLL1 |
| SPARC | 1.60E-41 | 0.464221645 | 0.255 | 0.124 | 4.99E-37 | Macro_SPP1 | SPARC |
| MT-ATP6 | 2.16E-41 | 0.371919536 | 0.989 | 0.996 | 6.73E-37 | Macro_SPP1 | MT-ATP6 |
| VSIG41 | 1.69E-40 | 0.497503337 | 0.389 | 0.233 | 5.27E-36 | Macro_SPP1 | VSIG4 |
| GNS | 3.84E-40 | 0.437533716 | 0.542 | 0.384 | 1.19E-35 | Macro_SPP1 | GNS |
| MERTK1 | 4.00E-40 | 0.42112939 | 0.243 | 0.12 | 1.25E-35 | Macro_SPP1 | MERTK |
| FCGRT1 | 5.05E-40 | 0.404237889 | 0.777 | 0.667 | 1.57E-35 | Macro_SPP1 | FCGRT |
| AHNAK | 1.08E-39 | 0.480335334 | 0.461 | 0.31 | 3.35E-35 | Macro_SPP1 | AHNAK |
| SLC38A6 | 1.52E-39 | 0.329159476 | 0.209 | 0.094 | 4.74E-35 | Macro_SPP1 | SLC38A6 |
| EGR1 | 1.72E-39 | 0.70204133 | 0.49 | 0.335 | 5.34E-35 | Macro_SPP1 | EGR1 |
| JUND | 1.86E-39 | 0.578501022 | 0.749 | 0.639 | 5.79E-35 | Macro_SPP1 | JUND |
| DBI | 1.94E-39 | 0.402255852 | 0.825 | 0.75 | 6.04E-35 | Macro_SPP1 | DBI |
| C21 | 2.62E-39 | 0.414314733 | 0.31 | 0.169 | 8.14E-35 | Macro_SPP1 | C2 |
| LRP1 | 4.25E-39 | 0.384042961 | 0.351 | 0.205 | 1.32E-34 | Macro_SPP1 | LRP1 |
| PDPN | 8.93E-39 | 0.304162089 | 0.178 | 0.07 | 2.78E-34 | Macro_SPP1 | PDPN |
| HNMT1 | 1.29E-38 | 0.429019858 | 0.542 | 0.387 | 4.00E-34 | Macro_SPP1 | HNMT |
| MS4A71 | 2.40E-38 | 0.504102152 | 0.723 | 0.599 | 7.47E-34 | Macro_SPP1 | MS4A7 |
| FAM198B | 2.44E-38 | 0.377184442 | 0.245 | 0.122 | 7.61E-34 | Macro_SPP1 | FAM198B |
| ATP6V0E11 | 2.89E-38 | 0.33742222 | 0.88 | 0.833 | 9.01E-34 | Macro_SPP1 | ATP6V0E1 |
| CXCL1 | 3.59E-38 | 0.374512092 | 0.561 | 0.409 | 1.12E-33 | Macro_SPP1 | CXCL1 |
| SCARB21 | 4.54E-38 | 0.410411068 | 0.455 | 0.302 | 1.41E-33 | Macro_SPP1 | SCARB2 |
| TMSB4X1 | 7.18E-38 | 0.260441713 | 1 | 1 | 2.24E-33 | Macro_SPP1 | TMSB4X |
| BCAP311 | 2.06E-37 | 0.435845238 | 0.612 | 0.485 | 6.42E-33 | Macro_SPP1 | BCAP31 |
| ATP6V1F | 2.30E-37 | 0.364142055 | 0.85 | 0.818 | 7.15E-33 | Macro_SPP1 | ATP6V1F |
| NR1H31 | 4.81E-37 | 0.355850333 | 0.223 | 0.106 | 1.50E-32 | Macro_SPP1 | NR1H3 |
| IFI271 | 1.18E-36 | 0.981334479 | 0.459 | 0.319 | 3.68E-32 | Macro_SPP1 | IFI27 |
| PLXND1 | 1.24E-36 | 0.398110136 | 0.323 | 0.191 | 3.87E-32 | Macro_SPP1 | PLXND1 |
| TGFBI | 1.72E-36 | 0.560355666 | 0.734 | 0.618 | 5.34E-32 | Macro_SPP1 | TGFBI |
| OLFML2B1 | 2.29E-36 | 0.383530683 | 0.232 | 0.115 | 7.13E-32 | Macro_SPP1 | OLFML2B |
| TFPT1 | 3.15E-36 | 0.41498088 | 0.384 | 0.247 | 9.80E-32 | Macro_SPP1 | TFPT |
| LINC01094 | 5.56E-36 | 0.285676263 | 0.19 | 0.084 | 1.73E-31 | Macro_SPP1 | LINC01094 |
| HTRA1 | 7.13E-36 | 0.429290054 | 0.187 | 0.082 | 2.22E-31 | Macro_SPP1 | HTRA1 |
| CHI3L1 | 1.28E-35 | 0.533313378 | 0.125 | 0.041 | 3.99E-31 | Macro_SPP1 | CHI3L1 |
| TMEM176A1 | 1.36E-35 | 0.380319059 | 0.599 | 0.429 | 4.22E-31 | Macro_SPP1 | TMEM176A |
| FCGR2A | 1.99E-35 | 0.510241387 | 0.781 | 0.719 | 6.20E-31 | Macro_SPP1 | FCGR2A |
| COMT1 | 2.42E-35 | 0.430506583 | 0.534 | 0.403 | 7.54E-31 | Macro_SPP1 | COMT |
| PPIC | 7.58E-35 | 0.254387491 | 0.14 | 0.051 | 2.36E-30 | Macro_SPP1 | PPIC |
| GOLIM4 | 1.06E-34 | 0.387901101 | 0.248 | 0.132 | 3.28E-30 | Macro_SPP1 | GOLIM4 |
| RBP1 | 1.74E-34 | 0.316306448 | 0.147 | 0.056 | 5.41E-30 | Macro_SPP1 | RBP1 |
| HMOX11 | 2.56E-34 | 0.622728634 | 0.492 | 0.341 | 7.97E-30 | Macro_SPP1 | HMOX1 |
| TXNIP | 4.06E-34 | 0.570747983 | 0.418 | 0.274 | 1.26E-29 | Macro_SPP1 | TXNIP |
| CYP27A11 | 6.23E-34 | 0.33335453 | 0.191 | 0.086 | 1.94E-29 | Macro_SPP1 | CYP27A1 |
| MITF | 7.19E-34 | 0.299729893 | 0.21 | 0.101 | 2.24E-29 | Macro_SPP1 | MITF |
| PAPSS1 | 7.26E-34 | 0.361062168 | 0.346 | 0.213 | 2.26E-29 | Macro_SPP1 | PAPSS1 |
| ATP6AP21 | 2.89E-33 | 0.420021414 | 0.679 | 0.565 | 8.99E-29 | Macro_SPP1 | ATP6AP2 |
| LGALS1 | 6.26E-33 | 0.329104152 | 0.978 | 0.982 | 1.95E-28 | Macro_SPP1 | LGALS1 |
| CD276 | 8.11E-33 | 0.287519155 | 0.208 | 0.102 | 2.52E-28 | Macro_SPP1 | CD276 |
| NTAN1 | 1.64E-32 | 0.327860883 | 0.259 | 0.144 | 5.11E-28 | Macro_SPP1 | NTAN1 |
| S100A11 | 1.96E-32 | 0.314604239 | 0.972 | 0.974 | 6.10E-28 | Macro_SPP1 | S100A11 |
| LINC009981 | 2.77E-32 | 0.41542939 | 0.471 | 0.338 | 8.63E-28 | Macro_SPP1 | LINC00998 |
| ATP6AP11 | 3.72E-32 | 0.39687359 | 0.574 | 0.439 | 1.16E-27 | Macro_SPP1 | ATP6AP1 |
| ISCU1 | 4.54E-32 | 0.404661839 | 0.489 | 0.354 | 1.41E-27 | Macro_SPP1 | ISCU |
| ZFP36L1 | 6.11E-32 | 0.356027357 | 0.673 | 0.548 | 1.90E-27 | Macro_SPP1 | ZFP36L1 |
| ERRFI1 | 6.88E-32 | 0.424580149 | 0.203 | 0.1 | 2.14E-27 | Macro_SPP1 | ERRFI1 |
| ITGAM | 9.03E-32 | 0.360144742 | 0.34 | 0.209 | 2.81E-27 | Macro_SPP1 | ITGAM |
| FMNL21 | 1.87E-31 | 0.329951329 | 0.249 | 0.135 | 5.81E-27 | Macro_SPP1 | FMNL2 |
| SLC1A3 | 2.25E-31 | 0.316125551 | 0.257 | 0.139 | 7.01E-27 | Macro_SPP1 | SLC1A3 |
| ANXA4 | 2.49E-31 | 0.407724837 | 0.362 | 0.238 | 7.76E-27 | Macro_SPP1 | ANXA4 |
| HEXA1 | 2.90E-31 | 0.377701372 | 0.451 | 0.317 | 9.01E-27 | Macro_SPP1 | HEXA |
| ARL4C1 | 6.42E-31 | 0.508794282 | 0.489 | 0.355 | 2.00E-26 | Macro_SPP1 | ARL4C |
| FAM20C | 9.02E-31 | 0.270269849 | 0.187 | 0.089 | 2.81E-26 | Macro_SPP1 | FAM20C |
| PLA2G71 | 1.21E-30 | 0.53695447 | 0.511 | 0.382 | 3.77E-26 | Macro_SPP1 | PLA2G7 |
| SERINC1 | 2.72E-30 | 0.388197391 | 0.44 | 0.307 | 8.47E-26 | Macro_SPP1 | SERINC1 |
| BMP2K1 | 3.20E-30 | 0.365536741 | 0.292 | 0.173 | 9.97E-26 | Macro_SPP1 | BMP2K |
| MPC21 | 3.92E-30 | 0.399612717 | 0.385 | 0.261 | 1.22E-25 | Macro_SPP1 | MPC2 |
| DPP71 | 3.95E-30 | 0.391501404 | 0.552 | 0.425 | 1.23E-25 | Macro_SPP1 | DPP7 |
| CTSA1 | 5.47E-30 | 0.361451157 | 0.585 | 0.455 | 1.70E-25 | Macro_SPP1 | CTSA |
| FAM96A | 5.66E-30 | 0.403717373 | 0.448 | 0.324 | 1.76E-25 | Macro_SPP1 | FAM96A |
| NDUFB7 | 6.79E-30 | 0.369640346 | 0.623 | 0.506 | 2.11E-25 | Macro_SPP1 | NDUFB7 |
| F13A1 | 7.20E-30 | 0.830709768 | 0.21 | 0.112 | 2.24E-25 | Macro_SPP1 | F13A1 |
| ITGB5 | 7.63E-30 | 0.303937253 | 0.16 | 0.072 | 2.37E-25 | Macro_SPP1 | ITGB5 |
| ALCAM | 8.40E-30 | 0.399005518 | 0.336 | 0.216 | 2.61E-25 | Macro_SPP1 | ALCAM |
| HSD17B141 | 9.27E-30 | 0.283208883 | 0.177 | 0.083 | 2.88E-25 | Macro_SPP1 | HSD17B14 |
| CYFIP11 | 1.31E-29 | 0.340517919 | 0.362 | 0.236 | 4.07E-25 | Macro_SPP1 | CYFIP1 |
| MT-CYB | 2.87E-29 | 0.298826361 | 0.984 | 0.989 | 8.94E-25 | Macro_SPP1 | MT-CYB |
| MT-CO3 | 2.89E-29 | 0.293534606 | 0.99 | 0.996 | 8.98E-25 | Macro_SPP1 | MT-CO3 |
| MT-CO2 | 3.84E-29 | 0.274683629 | 0.993 | 0.997 | 1.20E-24 | Macro_SPP1 | MT-CO2 |
| NCEH1 | 4.06E-29 | 0.311029376 | 0.203 | 0.104 | 1.26E-24 | Macro_SPP1 | NCEH1 |
| CMTM31 | 4.45E-29 | 0.372558723 | 0.372 | 0.25 | 1.38E-24 | Macro_SPP1 | CMTM3 |
| TSPAN41 | 5.21E-29 | 0.330932562 | 0.367 | 0.238 | 1.62E-24 | Macro_SPP1 | TSPAN4 |
| CALM21 | 8.73E-29 | 0.29689727 | 0.834 | 0.73 | 2.72E-24 | Macro_SPP1 | CALM2 |
| MT-ND2 | 1.80E-28 | 0.310678216 | 0.99 | 0.996 | 5.60E-24 | Macro_SPP1 | MT-ND2 |
| CD109 | 1.84E-28 | 0.323324021 | 0.235 | 0.127 | 5.71E-24 | Macro_SPP1 | CD109 |
| CYB5A | 2.43E-28 | 0.334248326 | 0.32 | 0.202 | 7.55E-24 | Macro_SPP1 | CYB5A |
| BRI31 | 2.51E-28 | 0.416710373 | 0.632 | 0.541 | 7.81E-24 | Macro_SPP1 | BRI3 |
| C1orf541 | 2.76E-28 | 0.454877745 | 0.385 | 0.262 | 8.57E-24 | Macro_SPP1 | C1orf54 |
| NENF1 | 3.71E-28 | 0.4053369 | 0.449 | 0.33 | 1.15E-23 | Macro_SPP1 | NENF |
| IFI27L2 | 3.71E-28 | 0.368945273 | 0.567 | 0.448 | 1.16E-23 | Macro_SPP1 | IFI27L2 |
| SCCPDH | 5.81E-28 | 0.318551861 | 0.211 | 0.112 | 1.81E-23 | Macro_SPP1 | SCCPDH |
| AKR1B11 | 6.91E-28 | 0.337486903 | 0.45 | 0.315 | 2.15E-23 | Macro_SPP1 | AKR1B1 |
| ABHD2 | 6.98E-28 | 0.339458951 | 0.278 | 0.168 | 2.17E-23 | Macro_SPP1 | ABHD2 |
| ADAP21 | 7.82E-28 | 0.332612362 | 0.338 | 0.216 | 2.43E-23 | Macro_SPP1 | ADAP2 |
| TMBIM61 | 9.59E-28 | 0.341601004 | 0.827 | 0.754 | 2.98E-23 | Macro_SPP1 | TMBIM6 |
| GNAS | 1.07E-27 | 0.408183955 | 0.604 | 0.496 | 3.32E-23 | Macro_SPP1 | GNAS |
| SGK11 | 1.76E-27 | 0.503168339 | 0.691 | 0.599 | 5.47E-23 | Macro_SPP1 | SGK1 |
| CLTC | 1.96E-27 | 0.423268911 | 0.419 | 0.3 | 6.09E-23 | Macro_SPP1 | CLTC |
| CCL8 | 2.18E-27 | 0.720390975 | 0.136 | 0.056 | 6.79E-23 | Macro_SPP1 | CCL8 |
| PMP22 | 2.23E-27 | 0.362054545 | 0.57 | 0.442 | 6.94E-23 | Macro_SPP1 | PMP22 |
| LGALS3BP1 | 3.91E-27 | 0.362033311 | 0.37 | 0.246 | 1.22E-22 | Macro_SPP1 | LGALS3BP |
| GLMP1 | 1.17E-26 | 0.294624703 | 0.27 | 0.161 | 3.65E-22 | Macro_SPP1 | GLMP |
| CD164 | 1.70E-26 | 0.349464218 | 0.61 | 0.517 | 5.29E-22 | Macro_SPP1 | CD164 |
| FCHO2 | 2.46E-26 | 0.269130726 | 0.171 | 0.084 | 7.65E-22 | Macro_SPP1 | FCHO2 |
| TMEM176B1 | 4.02E-26 | 0.253980992 | 0.668 | 0.511 | 1.25E-21 | Macro_SPP1 | TMEM176B |
| GAA1 | 4.92E-26 | 0.306230395 | 0.336 | 0.22 | 1.53E-21 | Macro_SPP1 | GAA |
| ALOX5AP | 6.89E-26 | 0.346811042 | 0.649 | 0.522 | 2.14E-21 | Macro_SPP1 | ALOX5AP |
| MT-CO1 | 1.08E-25 | 0.252718782 | 0.995 | 0.998 | 3.36E-21 | Macro_SPP1 | MT-CO1 |
| YWHAH1 | 1.08E-25 | 0.285310312 | 0.631 | 0.482 | 3.37E-21 | Macro_SPP1 | YWHAH |
| ATP5I | 1.81E-25 | 0.379238832 | 0.714 | 0.622 | 5.63E-21 | Macro_SPP1 | ATP5I |
| RAP2B1 | 3.38E-25 | 0.365839141 | 0.323 | 0.214 | 1.05E-20 | Macro_SPP1 | RAP2B |
| EGR2 | 5.82E-25 | 0.440145349 | 0.192 | 0.101 | 1.81E-20 | Macro_SPP1 | EGR2 |
| ATPIF11 | 9.22E-25 | 0.35819924 | 0.498 | 0.388 | 2.87E-20 | Macro_SPP1 | ATPIF1 |
| GBA | 9.79E-25 | 0.303856508 | 0.266 | 0.162 | 3.05E-20 | Macro_SPP1 | GBA |
| SLC40A11 | 1.11E-24 | 0.561110647 | 0.274 | 0.169 | 3.45E-20 | Macro_SPP1 | SLC40A1 |
| ARL6IP1 | 1.72E-24 | 0.320697629 | 0.631 | 0.514 | 5.36E-20 | Macro_SPP1 | ARL6IP1 |
| LAPTM4A1 | 2.05E-24 | 0.381509041 | 0.661 | 0.569 | 6.38E-20 | Macro_SPP1 | LAPTM4A |
| LILRB5 | 2.15E-24 | 0.33750366 | 0.14 | 0.064 | 6.70E-20 | Macro_SPP1 | LILRB5 |
| APP | 2.31E-24 | 0.315378443 | 0.333 | 0.219 | 7.20E-20 | Macro_SPP1 | APP |
| C6orf62 | 2.51E-24 | 0.387546579 | 0.447 | 0.335 | 7.82E-20 | Macro_SPP1 | C6orf62 |
| MFSD12 | 2.93E-24 | 0.360301345 | 0.308 | 0.204 | 9.12E-20 | Macro_SPP1 | MFSD12 |
| NDUFB2 | 3.07E-24 | 0.314412863 | 0.744 | 0.657 | 9.56E-20 | Macro_SPP1 | NDUFB2 |
| APLP2 | 3.78E-24 | 0.319017277 | 0.704 | 0.631 | 1.18E-19 | Macro_SPP1 | APLP2 |
| UGCG | 4.03E-24 | 0.31796003 | 0.243 | 0.145 | 1.25E-19 | Macro_SPP1 | UGCG |
| PEBP11 | 5.16E-24 | 0.345705348 | 0.479 | 0.358 | 1.61E-19 | Macro_SPP1 | PEBP1 |
| PRDX11 | 6.06E-24 | 0.283832978 | 0.821 | 0.729 | 1.89E-19 | Macro_SPP1 | PRDX1 |
| CD41 | 7.81E-24 | 0.34690094 | 0.511 | 0.384 | 2.43E-19 | Macro_SPP1 | CD4 |
| TMEM2191 | 7.97E-24 | 0.335291001 | 0.57 | 0.47 | 2.48E-19 | Macro_SPP1 | TMEM219 |
| ANXA2 | 9.74E-24 | 0.313388667 | 0.885 | 0.868 | 3.03E-19 | Macro_SPP1 | ANXA2 |
| IGF2R | 1.04E-23 | 0.294420075 | 0.266 | 0.164 | 3.23E-19 | Macro_SPP1 | IGF2R |
| RENBP1 | 1.45E-23 | 0.323950345 | 0.312 | 0.205 | 4.52E-19 | Macro_SPP1 | RENBP |
| TMIGD3 | 1.78E-23 | 0.352762453 | 0.115 | 0.048 | 5.53E-19 | Macro_SPP1 | TMIGD3 |
| NRP21 | 2.03E-23 | 0.333640851 | 0.405 | 0.289 | 6.33E-19 | Macro_SPP1 | NRP2 |
| S100A13 | 2.36E-23 | 0.29230873 | 0.186 | 0.1 | 7.35E-19 | Macro_SPP1 | S100A13 |
| TGFBR2 | 2.58E-23 | 0.295100861 | 0.214 | 0.122 | 8.04E-19 | Macro_SPP1 | TGFBR2 |
| CD2091 | 2.76E-23 | 0.353394613 | 0.172 | 0.09 | 8.59E-19 | Macro_SPP1 | CD209 |
| ASPH | 3.42E-23 | 0.317188053 | 0.294 | 0.189 | 1.07E-18 | Macro_SPP1 | ASPH |
| DSTN | 3.45E-23 | 0.346288517 | 0.462 | 0.355 | 1.07E-18 | Macro_SPP1 | DSTN |
| AP2S1 | 3.99E-23 | 0.304570135 | 0.824 | 0.767 | 1.24E-18 | Macro_SPP1 | AP2S1 |
| AP1B11 | 1.00E-22 | 0.388138301 | 0.315 | 0.215 | 3.12E-18 | Macro_SPP1 | AP1B1 |
| NPTN | 1.06E-22 | 0.301169742 | 0.291 | 0.186 | 3.29E-18 | Macro_SPP1 | NPTN |
| ACOT13 | 2.91E-22 | 0.268370788 | 0.219 | 0.13 | 9.06E-18 | Macro_SPP1 | ACOT13 |
| CSF1R1 | 3.29E-22 | 0.325194154 | 0.514 | 0.395 | 1.02E-17 | Macro_SPP1 | CSF1R |
| UQCR101 | 4.89E-22 | 0.299818363 | 0.695 | 0.596 | 1.52E-17 | Macro_SPP1 | UQCR10 |
| GAL3ST41 | 4.89E-22 | 0.263918202 | 0.183 | 0.1 | 1.52E-17 | Macro_SPP1 | GAL3ST4 |
| MT-ND3 | 5.21E-22 | 0.328059131 | 0.976 | 0.977 | 1.62E-17 | Macro_SPP1 | MT-ND3 |
| PECAM1 | 6.29E-22 | 0.271215776 | 0.353 | 0.243 | 1.96E-17 | Macro_SPP1 | PECAM1 |
| PTMS1 | 8.00E-22 | 0.329436469 | 0.485 | 0.37 | 2.49E-17 | Macro_SPP1 | PTMS |
| SEPW11 | 1.09E-21 | 0.349662825 | 0.482 | 0.382 | 3.39E-17 | Macro_SPP1 | SEPW1 |
| COX6C1 | 1.25E-21 | 0.331285715 | 0.772 | 0.695 | 3.88E-17 | Macro_SPP1 | COX6C |
| FBP1 | 1.54E-21 | 0.627008075 | 0.37 | 0.271 | 4.79E-17 | Macro_SPP1 | FBP1 |
| ATP6V0B | 1.67E-21 | 0.276370247 | 0.82 | 0.771 | 5.21E-17 | Macro_SPP1 | ATP6V0B |
| FUCA11 | 2.33E-21 | 0.281479913 | 0.258 | 0.161 | 7.26E-17 | Macro_SPP1 | FUCA1 |
| VEGFB | 3.97E-21 | 0.278641225 | 0.19 | 0.107 | 1.23E-16 | Macro_SPP1 | VEGFB |
| SELM | 6.07E-21 | 0.262638607 | 0.254 | 0.158 | 1.89E-16 | Macro_SPP1 | SELM |
| PTGS1 | 6.50E-21 | 0.27448088 | 0.262 | 0.169 | 2.02E-16 | Macro_SPP1 | PTGS1 |
| KIAA0930 | 7.32E-21 | 0.315408724 | 0.256 | 0.165 | 2.28E-16 | Macro_SPP1 | KIAA0930 |
| AGR2 | 9.10E-21 | 0.367873954 | 0.302 | 0.204 | 2.83E-16 | Macro_SPP1 | AGR2 |
| UQCR11 | 1.01E-20 | 0.264090507 | 0.782 | 0.716 | 3.15E-16 | Macro_SPP1 | UQCR11 |
| SCP21 | 2.25E-20 | 0.310015628 | 0.503 | 0.391 | 6.99E-16 | Macro_SPP1 | SCP2 |
| SNX61 | 2.34E-20 | 0.3620213 | 0.41 | 0.308 | 7.29E-16 | Macro_SPP1 | SNX6 |
| PHACTR4 | 2.89E-20 | 0.284365348 | 0.224 | 0.137 | 8.99E-16 | Macro_SPP1 | PHACTR4 |
| SLC12A5 | 2.93E-20 | 0.250425066 | 0.128 | 0.061 | 9.10E-16 | Macro_SPP1 | SLC12A5 |
| CHCHD101 | 3.35E-20 | 0.332905286 | 0.564 | 0.461 | 1.04E-15 | Macro_SPP1 | CHCHD10 |
| PPDPF | 3.91E-20 | 0.334174667 | 0.691 | 0.625 | 1.22E-15 | Macro_SPP1 | PPDPF |
| ABHD121 | 3.93E-20 | 0.304369053 | 0.329 | 0.234 | 1.22E-15 | Macro_SPP1 | ABHD12 |
| RABAC1 | 4.38E-20 | 0.317739583 | 0.536 | 0.437 | 1.36E-15 | Macro_SPP1 | RABAC1 |
| CCR1 | 4.45E-20 | 0.251322046 | 0.286 | 0.184 | 1.39E-15 | Macro_SPP1 | CCR1 |
| C19orf60 | 4.56E-20 | 0.306981197 | 0.536 | 0.437 | 1.42E-15 | Macro_SPP1 | C19orf60 |
| ZFAND51 | 5.07E-20 | 0.34644588 | 0.665 | 0.575 | 1.58E-15 | Macro_SPP1 | ZFAND5 |
| CALM1 | 1.27E-19 | 0.262077243 | 0.834 | 0.803 | 3.94E-15 | Macro_SPP1 | CALM1 |
| LAMTOR21 | 1.30E-19 | 0.293227264 | 0.61 | 0.513 | 4.04E-15 | Macro_SPP1 | LAMTOR2 |
| ETV5 | 1.82E-19 | 0.292846513 | 0.22 | 0.137 | 5.65E-15 | Macro_SPP1 | ETV5 |
| LGALS31 | 1.87E-19 | 0.353464774 | 0.808 | 0.766 | 5.82E-15 | Macro_SPP1 | LGALS3 |
| CYBB1 | 4.04E-19 | 0.342626463 | 0.649 | 0.549 | 1.26E-14 | Macro_SPP1 | CYBB |
| TMEM160 | 5.90E-19 | 0.309038266 | 0.453 | 0.348 | 1.84E-14 | Macro_SPP1 | TMEM160 |
| AP2A21 | 6.03E-19 | 0.297369892 | 0.216 | 0.136 | 1.88E-14 | Macro_SPP1 | AP2A2 |
| IFIT3 | 6.32E-19 | 0.32510592 | 0.233 | 0.144 | 1.97E-14 | Macro_SPP1 | IFIT3 |
| C9orf16 | 6.72E-19 | 0.30503572 | 0.524 | 0.429 | 2.09E-14 | Macro_SPP1 | C9orf16 |
| CD141 | 7.92E-19 | 0.270252363 | 0.779 | 0.677 | 2.46E-14 | Macro_SPP1 | CD14 |
| CPM | 8.16E-19 | 0.310125916 | 0.297 | 0.203 | 2.54E-14 | Macro_SPP1 | CPM |
| FRMD4B1 | 1.14E-18 | 0.282490629 | 0.238 | 0.154 | 3.55E-14 | Macro_SPP1 | FRMD4B |
| RASGRP3 | 1.15E-18 | 0.328284709 | 0.312 | 0.22 | 3.59E-14 | Macro_SPP1 | RASGRP3 |
| C19orf70 | 1.65E-18 | 0.308644225 | 0.476 | 0.376 | 5.15E-14 | Macro_SPP1 | C19orf70 |
| FIS1 | 1.91E-18 | 0.286269314 | 0.518 | 0.414 | 5.93E-14 | Macro_SPP1 | FIS1 |
| CLTA1 | 2.25E-18 | 0.309657793 | 0.63 | 0.542 | 7.02E-14 | Macro_SPP1 | CLTA |
| TPST2 | 2.28E-18 | 0.256217086 | 0.231 | 0.148 | 7.10E-14 | Macro_SPP1 | TPST2 |
| TMEM1471 | 2.33E-18 | 0.280015719 | 0.512 | 0.419 | 7.24E-14 | Macro_SPP1 | TMEM147 |
| SIRPA | 3.18E-18 | 0.294235291 | 0.429 | 0.336 | 9.90E-14 | Macro_SPP1 | SIRPA |
| RTN3 | 3.82E-18 | 0.268817831 | 0.408 | 0.306 | 1.19E-13 | Macro_SPP1 | RTN3 |
| IGFBP4 | 3.86E-18 | 0.275787249 | 0.163 | 0.092 | 1.20E-13 | Macro_SPP1 | IGFBP4 |
| ABL2 | 4.16E-18 | 0.372054667 | 0.566 | 0.478 | 1.29E-13 | Macro_SPP1 | ABL2 |
| CTDNEP1 | 4.22E-18 | 0.284321002 | 0.301 | 0.209 | 1.31E-13 | Macro_SPP1 | CTDNEP1 |
| PPT11 | 4.31E-18 | 0.27504079 | 0.645 | 0.546 | 1.34E-13 | Macro_SPP1 | PPT1 |
| ZFP36L21 | 4.82E-18 | 0.258193691 | 0.485 | 0.37 | 1.50E-13 | Macro_SPP1 | ZFP36L2 |
| NR4A21 | 4.83E-18 | 0.461913048 | 0.579 | 0.508 | 1.50E-13 | Macro_SPP1 | NR4A2 |
| GNB41 | 5.20E-18 | 0.27586103 | 0.33 | 0.237 | 1.62E-13 | Macro_SPP1 | GNB4 |
| CALU | 8.34E-18 | 0.306407931 | 0.305 | 0.217 | 2.60E-13 | Macro_SPP1 | CALU |
| DUSP3 | 1.39E-17 | 0.28549149 | 0.3 | 0.212 | 4.33E-13 | Macro_SPP1 | DUSP3 |
| ENG | 1.52E-17 | 0.275109085 | 0.368 | 0.273 | 4.73E-13 | Macro_SPP1 | ENG |
| ME2 | 1.99E-17 | 0.278878173 | 0.288 | 0.201 | 6.18E-13 | Macro_SPP1 | ME2 |
| SERPING11 | 6.40E-17 | 0.259131317 | 0.309 | 0.215 | 1.99E-12 | Macro_SPP1 | SERPING1 |
| IQGAP21 | 8.95E-17 | 0.266520454 | 0.311 | 0.222 | 2.79E-12 | Macro_SPP1 | IQGAP2 |
| ITGB1 | 9.20E-17 | 0.287534402 | 0.551 | 0.464 | 2.86E-12 | Macro_SPP1 | ITGB1 |
| TMEM14C1 | 1.41E-16 | 0.275485706 | 0.511 | 0.411 | 4.40E-12 | Macro_SPP1 | TMEM14C |
| TCEAL4 | 2.02E-16 | 0.264683564 | 0.295 | 0.209 | 6.27E-12 | Macro_SPP1 | TCEAL4 |
| RHOC | 2.19E-16 | 0.295062055 | 0.457 | 0.368 | 6.81E-12 | Macro_SPP1 | RHOC |
| C3AR11 | 2.92E-16 | 0.252623242 | 0.388 | 0.292 | 9.09E-12 | Macro_SPP1 | C3AR1 |
| NDUFA3 | 4.15E-16 | 0.317432546 | 0.557 | 0.472 | 1.29E-11 | Macro_SPP1 | NDUFA3 |
| NDUFA11 | 6.87E-16 | 0.275331349 | 0.645 | 0.571 | 2.14E-11 | Macro_SPP1 | NDUFA11 |
| RB11 | 7.10E-16 | 0.385929792 | 0.347 | 0.266 | 2.21E-11 | Macro_SPP1 | RB1 |
| SCPEP11 | 7.55E-16 | 0.26122396 | 0.447 | 0.349 | 2.35E-11 | Macro_SPP1 | SCPEP1 |
| GTF2H5 | 7.67E-16 | 0.316012931 | 0.375 | 0.285 | 2.39E-11 | Macro_SPP1 | GTF2H5 |
| CISD3 | 8.34E-16 | 0.284583611 | 0.375 | 0.287 | 2.59E-11 | Macro_SPP1 | CISD3 |
| USMG5 | 1.02E-15 | 0.29649659 | 0.706 | 0.664 | 3.17E-11 | Macro_SPP1 | USMG5 |
| ATP6V1A | 1.17E-15 | 0.274777576 | 0.341 | 0.255 | 3.64E-11 | Macro_SPP1 | ATP6V1A |
| CRTAP | 1.18E-15 | 0.282034402 | 0.314 | 0.23 | 3.66E-11 | Macro_SPP1 | CRTAP |
| MRPL571 | 1.32E-15 | 0.266123604 | 0.4 | 0.31 | 4.11E-11 | Macro_SPP1 | MRPL57 |
| COX5B | 1.43E-15 | 0.264469313 | 0.794 | 0.746 | 4.46E-11 | Macro_SPP1 | COX5B |
| ITGB21 | 1.49E-15 | 0.265330382 | 0.756 | 0.675 | 4.62E-11 | Macro_SPP1 | ITGB2 |
| BLVRB1 | 1.54E-15 | 0.311983262 | 0.565 | 0.479 | 4.78E-11 | Macro_SPP1 | BLVRB |
| ANKH | 1.63E-15 | 0.320709166 | 0.207 | 0.136 | 5.07E-11 | Macro_SPP1 | ANKH |
| ARL21 | 1.79E-15 | 0.285754806 | 0.304 | 0.226 | 5.56E-11 | Macro_SPP1 | ARL2 |
| RNF13 | 2.23E-15 | 0.259064063 | 0.508 | 0.421 | 6.93E-11 | Macro_SPP1 | RNF13 |
| LAIR11 | 2.29E-15 | 0.256486916 | 0.492 | 0.394 | 7.13E-11 | Macro_SPP1 | LAIR1 |
| CALM3 | 2.68E-15 | 0.291632042 | 0.63 | 0.56 | 8.34E-11 | Macro_SPP1 | CALM3 |
| DUSP23 | 3.64E-15 | 0.259399273 | 0.39 | 0.302 | 1.13E-10 | Macro_SPP1 | DUSP23 |
| G3BP1 | 3.83E-15 | 0.265183179 | 0.304 | 0.22 | 1.19E-10 | Macro_SPP1 | G3BP1 |
| MT1X | 4.13E-15 | 0.421665332 | 0.391 | 0.3 | 1.28E-10 | Macro_SPP1 | MT1X |
| RWDD1 | 6.20E-15 | 0.263268471 | 0.421 | 0.332 | 1.93E-10 | Macro_SPP1 | RWDD1 |
| VTI1B | 8.82E-15 | 0.25247215 | 0.329 | 0.248 | 2.75E-10 | Macro_SPP1 | VTI1B |
| CAPZA2 | 9.91E-15 | 0.251204162 | 0.587 | 0.507 | 3.08E-10 | Macro_SPP1 | CAPZA2 |
| HIF1A | 1.05E-14 | 0.311992202 | 0.718 | 0.666 | 3.25E-10 | Macro_SPP1 | HIF1A |
| CANX1 | 1.29E-14 | 0.265147731 | 0.609 | 0.529 | 4.01E-10 | Macro_SPP1 | CANX |
| QKI | 1.37E-14 | 0.278638856 | 0.498 | 0.411 | 4.26E-10 | Macro_SPP1 | QKI |
| JUNB1 | 1.58E-14 | 0.306402995 | 0.748 | 0.69 | 4.91E-10 | Macro_SPP1 | JUNB |
| PLXDC2 | 1.70E-14 | 0.287619998 | 0.406 | 0.323 | 5.30E-10 | Macro_SPP1 | PLXDC2 |
| CORO1C | 3.12E-14 | 0.304207951 | 0.434 | 0.352 | 9.70E-10 | Macro_SPP1 | CORO1C |
| SNX21 | 3.50E-14 | 0.285641782 | 0.39 | 0.305 | 1.09E-09 | Macro_SPP1 | SNX2 |
| RBX1 | 4.39E-14 | 0.278392882 | 0.658 | 0.577 | 1.36E-09 | Macro_SPP1 | RBX1 |
| PPCS | 5.72E-14 | 0.254355952 | 0.298 | 0.22 | 1.78E-09 | Macro_SPP1 | PPCS |
| LASP1 | 6.39E-14 | 0.265932467 | 0.306 | 0.229 | 1.99E-09 | Macro_SPP1 | LASP1 |
| CD151 | 6.68E-14 | 0.262106417 | 0.382 | 0.302 | 2.08E-09 | Macro_SPP1 | CD151 |
| CCL7 | 7.47E-14 | 0.381105371 | 0.113 | 0.059 | 2.32E-09 | Macro_SPP1 | CCL7 |
| CLEC11A | 1.29E-13 | 0.255711686 | 0.258 | 0.183 | 4.00E-09 | Macro_SPP1 | CLEC11A |
| TGOLN2 | 1.29E-13 | 0.327044449 | 0.459 | 0.384 | 4.02E-09 | Macro_SPP1 | TGOLN2 |
| GNPDA11 | 5.25E-13 | 0.270239742 | 0.235 | 0.166 | 1.63E-08 | Macro_SPP1 | GNPDA1 |
| WNK1 | 5.29E-13 | 0.251131963 | 0.305 | 0.228 | 1.64E-08 | Macro_SPP1 | WNK1 |
| LEPROT | 7.72E-13 | 0.255798699 | 0.368 | 0.289 | 2.40E-08 | Macro_SPP1 | LEPROT |
| STX7 | 1.10E-12 | 0.26296584 | 0.317 | 0.244 | 3.41E-08 | Macro_SPP1 | STX7 |
| MT1G | 1.11E-12 | 0.346855258 | 0.18 | 0.115 | 3.45E-08 | Macro_SPP1 | MT1G |
| RPS19BP11 | 1.41E-12 | 0.273931782 | 0.442 | 0.363 | 4.40E-08 | Macro_SPP1 | RPS19BP1 |
| NDUFC21 | 2.03E-12 | 0.255691562 | 0.549 | 0.472 | 6.31E-08 | Macro_SPP1 | NDUFC2 |
| IFI6 | 2.44E-12 | 0.512922251 | 0.606 | 0.556 | 7.58E-08 | Macro_SPP1 | IFI6 |
| NOP10 | 2.95E-12 | 0.256327218 | 0.753 | 0.742 | 9.18E-08 | Macro_SPP1 | NOP10 |
| CCDC85B1 | 3.32E-12 | 0.281345656 | 0.373 | 0.295 | 1.03E-07 | Macro_SPP1 | CCDC85B |
| M6PR1 | 4.82E-12 | 0.259288413 | 0.544 | 0.467 | 1.50E-07 | Macro_SPP1 | M6PR |
| TUBA1C | 6.11E-12 | 0.263155043 | 0.455 | 0.383 | 1.90E-07 | Macro_SPP1 | TUBA1C |
| SDS1 | 6.85E-12 | 0.304631423 | 0.371 | 0.286 | 2.13E-07 | Macro_SPP1 | SDS |
| SLC15A3 | 9.79E-12 | 0.256332969 | 0.383 | 0.307 | 3.04E-07 | Macro_SPP1 | SLC15A3 |
| BEX4 | 1.14E-11 | 0.256833108 | 0.293 | 0.223 | 3.55E-07 | Macro_SPP1 | BEX4 |
| CREBL21 | 1.33E-11 | 0.267931772 | 0.297 | 0.228 | 4.14E-07 | Macro_SPP1 | CREBL2 |
| GEM | 1.53E-11 | 0.267617933 | 0.161 | 0.104 | 4.76E-07 | Macro_SPP1 | GEM |
| TMED101 | 1.97E-11 | 0.250538051 | 0.553 | 0.483 | 6.13E-07 | Macro_SPP1 | TMED10 |
| SPG21 | 5.86E-11 | 0.263268338 | 0.521 | 0.455 | 1.82E-06 | Macro_SPP1 | SPG21 |
| XIST | 1.41E-10 | 0.266608027 | 0.393 | 0.321 | 4.40E-06 | Macro_SPP1 | XIST |
| AP3S1 | 1.99E-10 | 0.256360977 | 0.42 | 0.356 | 6.18E-06 | Macro_SPP1 | AP3S1 |
| RPN1 | 2.14E-10 | 0.261606695 | 0.417 | 0.348 | 6.66E-06 | Macro_SPP1 | RPN1 |
| LRPAP11 | 5.57E-10 | 0.270093939 | 0.453 | 0.389 | 1.73E-05 | Macro_SPP1 | LRPAP1 |
| NDFIP11 | 1.55E-09 | 0.25623199 | 0.46 | 0.395 | 4.82E-05 | Macro_SPP1 | NDFIP1 |
| FOSB | 2.08E-09 | 0.376873329 | 0.708 | 0.699 | 6.49E-05 | Macro_SPP1 | FOSB |
| CNDP2 | 8.08E-09 | 0.261941173 | 0.376 | 0.316 | 0.000251533 | Macro_SPP1 | CNDP2 |
| KLF6 | 1.08E-08 | 0.281439556 | 0.851 | 0.856 | 0.000334541 | Macro_SPP1 | KLF6 |
| TNF | 1.74E-08 | 0.43537004 | 0.35 | 0.294 | 0.000540672 | Macro_SPP1 | TNF |
| CCL5 | 2.71E-08 | 0.278269636 | 0.262 | 0.207 | 0.000843177 | Macro_SPP1 | CCL5 |
| CFD | 3.00E-07 | 0.315508078 | 0.493 | 0.43 | 0.009344979 | Macro_SPP1 | CFD |
| FOS | 5.06E-07 | 0.27725196 | 0.726 | 0.694 | 0.015740033 | Macro_SPP1 | FOS |
| FDX1 | 6.38E-07 | 0.264254687 | 0.317 | 0.269 | 0.019849516 | Macro_SPP1 | FDX1 |
| FCER1A | 0 | 1.945545142 | 0.48 | 0.013 | 0 | cDC | FCER1A |
| CD1C | 0 | 1.749041446 | 0.582 | 0.025 | 0 | cDC | CD1C |
| CST7 | 3.07E-296 | 2.318650874 | 0.759 | 0.099 | 9.57E-292 | cDC | CST7 |
| PPP1R14A | 1.18E-264 | 0.967539433 | 0.355 | 0.014 | 3.67E-260 | cDC | PPP1R14A |
| CLEC10A | 1.72E-251 | 2.106752981 | 0.744 | 0.115 | 5.37E-247 | cDC | CLEC10A |
| CD1E | 7.16E-237 | 1.244657302 | 0.366 | 0.019 | 2.23E-232 | cDC | CD1E |
| CCR7 | 1.30E-186 | 1.895938435 | 0.497 | 0.06 | 4.04E-182 | cDC | CCR7 |
| JAML | 1.30E-173 | 1.518272726 | 0.784 | 0.186 | 4.04E-169 | cDC | JAML |
| HLA-DPB11 | 1.87E-161 | 1.750945241 | 1 | 0.875 | 5.81E-157 | cDC | HLA-DPB1 |
| PKIB | 4.42E-159 | 1.384998814 | 0.565 | 0.095 | 1.37E-154 | cDC | PKIB |
| SPIB | 1.51E-153 | 0.50517716 | 0.207 | 0.008 | 4.69E-149 | cDC | SPIB |
| ADAM191 | 2.25E-152 | 1.099341886 | 0.571 | 0.094 | 7.00E-148 | cDC | ADAM19 |
| CD207 | 3.11E-152 | 0.558142091 | 0.139 | 0.001 | 9.67E-148 | cDC | CD207 |
| PPA1 | 1.20E-145 | 2.011330224 | 0.932 | 0.41 | 3.74E-141 | cDC | PPA1 |
| HLA-DPA11 | 3.84E-136 | 1.585752822 | 1 | 0.868 | 1.19E-131 | cDC | HLA-DPA1 |
| HLA-DQB11 | 4.41E-134 | 1.671574112 | 0.997 | 0.767 | 1.37E-129 | cDC | HLA-DQB1 |
| CCL17 | 3.27E-133 | 2.177638509 | 0.173 | 0.006 | 1.02E-128 | cDC | CCL17 |
| GPR157 | 4.28E-125 | 0.769414001 | 0.378 | 0.05 | 1.33E-120 | cDC | GPR157 |
| HLA-DQA11 | 9.19E-124 | 1.691016543 | 0.994 | 0.714 | 2.86E-119 | cDC | HLA-DQA1 |
| HLA-DRA1 | 2.43E-122 | 1.210810894 | 1 | 0.984 | 7.55E-118 | cDC | HLA-DRA |
| CD741 | 9.87E-121 | 1.249629634 | 1 | 0.962 | 3.07E-116 | cDC | CD74 |
| NDRG21 | 8.02E-117 | 0.712588933 | 0.457 | 0.08 | 2.49E-112 | cDC | NDRG2 |
| DAPP1 | 1.98E-115 | 0.809906821 | 0.449 | 0.079 | 6.15E-111 | cDC | DAPP1 |
| LAMP3 | 1.20E-113 | 0.804694026 | 0.301 | 0.034 | 3.72E-109 | cDC | LAMP3 |
| PLD4 | 6.23E-113 | 0.679491559 | 0.344 | 0.046 | 1.94E-108 | cDC | PLD4 |
| RPL3 | 7.63E-112 | 0.969166064 | 1 | 0.957 | 2.38E-107 | cDC | RPL3 |
| CCL22 | 6.16E-111 | 1.635880677 | 0.233 | 0.019 | 1.92E-106 | cDC | CCL22 |
| RPL4 | 1.53E-110 | 1.135033585 | 0.997 | 0.805 | 4.76E-106 | cDC | RPL4 |
| SLC38A1 | 3.61E-110 | 0.41150515 | 0.247 | 0.022 | 1.12E-105 | cDC | SLC38A1 |
| RPS18 | 2.78E-106 | 0.881404645 | 0.997 | 0.99 | 8.66E-102 | cDC | RPS18 |
| RPL5 | 1.86E-105 | 1.0834891 | 0.997 | 0.836 | 5.78E-101 | cDC | RPL5 |
| RPL10A | 3.91E-101 | 0.915589713 | 0.997 | 0.911 | 1.22E-96 | cDC | RPL10A |
| CSF2RA | 9.50E-99 | 1.04468144 | 0.778 | 0.278 | 2.95E-94 | cDC | CSF2RA |
| RHOF | 5.57E-97 | 0.777321439 | 0.511 | 0.118 | 1.73E-92 | cDC | RHOF |
| STK4 | 5.43E-96 | 0.990917427 | 0.753 | 0.262 | 1.69E-91 | cDC | STK4 |
| RPL15 | 1.62E-94 | 0.784930617 | 0.997 | 0.966 | 5.05E-90 | cDC | RPL15 |
| RPS2 | 1.93E-94 | 0.814232212 | 1 | 0.987 | 6.01E-90 | cDC | RPS2 |
| RPS6 | 2.56E-94 | 0.795543226 | 1 | 0.979 | 7.96E-90 | cDC | RPS6 |
| HLA-DRB11 | 3.49E-94 | 1.345467155 | 0.997 | 0.912 | 1.09E-89 | cDC | HLA-DRB1 |
| RPS23 | 4.54E-94 | 0.852217206 | 0.997 | 0.97 | 1.41E-89 | cDC | RPS23 |
| RPS4X | 1.11E-92 | 0.914501505 | 0.997 | 0.961 | 3.44E-88 | cDC | RPS4X |
| IRF4 | 5.53E-91 | 0.436775061 | 0.247 | 0.028 | 1.72E-86 | cDC | IRF4 |
| CFP1 | 3.37E-90 | 0.773115057 | 0.631 | 0.166 | 1.05E-85 | cDC | CFP |
| RUNX3 | 3.71E-90 | 0.853487188 | 0.591 | 0.164 | 1.16E-85 | cDC | RUNX3 |
| RPS11 | 4.53E-89 | 0.826136496 | 0.997 | 0.956 | 1.41E-84 | cDC | RPS11 |
| HLA-DOB | 5.08E-89 | 0.27597486 | 0.134 | 0.006 | 1.58E-84 | cDC | HLA-DOB |
| EEF1A1 | 6.57E-89 | 0.757922424 | 1 | 0.997 | 2.04E-84 | cDC | EEF1A1 |
| RPS19 | 2.36E-88 | 0.661595198 | 1 | 0.993 | 7.36E-84 | cDC | RPS19 |
| SEPT6 | 8.94E-87 | 0.671917419 | 0.577 | 0.158 | 2.78E-82 | cDC | SEPT6 |
| RPL101 | 1.84E-85 | 0.655040303 | 1 | 0.997 | 5.71E-81 | cDC | RPL10 |
| RPS3 | 2.63E-85 | 0.741415569 | 1 | 0.968 | 8.19E-81 | cDC | RPS3 |
| RPS7 | 8.94E-85 | 0.797549092 | 0.997 | 0.944 | 2.78E-80 | cDC | RPS7 |
| CBX6 | 1.82E-84 | 0.679424524 | 0.426 | 0.091 | 5.67E-80 | cDC | CBX6 |
| RPL23A | 5.56E-84 | 0.798918392 | 0.997 | 0.958 | 1.73E-79 | cDC | RPL23A |
| LSP1 | 2.10E-83 | 1.163963199 | 0.912 | 0.465 | 6.53E-79 | cDC | LSP1 |
| RPL7A | 1.32E-81 | 0.81849042 | 0.994 | 0.914 | 4.11E-77 | cDC | RPL7A |
| RPL18A1 | 4.63E-81 | 0.728986908 | 1 | 0.984 | 1.44E-76 | cDC | RPL18A |
| RPL6 | 1.43E-80 | 0.823181152 | 0.994 | 0.922 | 4.44E-76 | cDC | RPL6 |
| CCND2 | 1.53E-80 | 0.64116966 | 0.472 | 0.113 | 4.76E-76 | cDC | CCND2 |
| SPINT21 | 3.49E-79 | 1.045471011 | 0.864 | 0.454 | 1.09E-74 | cDC | SPINT2 |
| PTGIR | 3.69E-79 | 0.386872529 | 0.216 | 0.024 | 1.15E-74 | cDC | PTGIR |
| RPS3A | 9.84E-79 | 0.80604222 | 0.997 | 0.967 | 3.06E-74 | cDC | RPS3A |
| RPL111 | 3.54E-78 | 0.681688682 | 1 | 0.983 | 1.10E-73 | cDC | RPL11 |
| ALDH2 | 1.23E-76 | 0.900775474 | 0.798 | 0.325 | 3.83E-72 | cDC | ALDH2 |
| RPS17 | 1.75E-76 | 0.725086855 | 1 | 0.956 | 5.45E-72 | cDC | RPS17 |
| RPL19 | 1.84E-76 | 0.665917062 | 1 | 0.979 | 5.73E-72 | cDC | RPL19 |
| CD1A | 9.58E-75 | 0.46225295 | 0.145 | 0.011 | 2.98E-70 | cDC | CD1A |
| RPS14 | 9.93E-75 | 0.639083844 | 0.997 | 0.987 | 3.09E-70 | cDC | RPS14 |
| RPSA1 | 3.18E-74 | 0.869332335 | 0.983 | 0.821 | 9.88E-70 | cDC | RPSA |
| RPS5 | 4.90E-74 | 0.741955063 | 0.994 | 0.922 | 1.53E-69 | cDC | RPS5 |
| RPL81 | 3.20E-72 | 0.666585948 | 1 | 0.975 | 9.94E-68 | cDC | RPL8 |
| RAMP1 | 6.94E-72 | 0.818456498 | 0.222 | 0.029 | 2.16E-67 | cDC | RAMP1 |
| PLAC81 | 1.23E-71 | 1.094914851 | 0.543 | 0.162 | 3.83E-67 | cDC | PLAC8 |
| REL | 6.73E-71 | 1.139684186 | 0.94 | 0.603 | 2.09E-66 | cDC | REL |
| GPAT3 | 6.98E-71 | 0.514157955 | 0.361 | 0.075 | 2.17E-66 | cDC | GPAT3 |
| CD1B | 1.10E-70 | 0.268746954 | 0.148 | 0.012 | 3.42E-66 | cDC | CD1B |
| LTB | 2.47E-70 | 1.289765124 | 0.344 | 0.073 | 7.68E-66 | cDC | LTB |
| RPS161 | 1.92E-69 | 0.646075168 | 1 | 0.982 | 5.96E-65 | cDC | RPS16 |
| LGALS21 | 6.79E-69 | 1.115323727 | 0.682 | 0.261 | 2.11E-64 | cDC | LGALS2 |
| EIF3L | 1.51E-68 | 0.875977258 | 0.818 | 0.398 | 4.69E-64 | cDC | EIF3L |
| RPS81 | 3.93E-68 | 0.73043055 | 0.994 | 0.95 | 1.22E-63 | cDC | RPS8 |
| GNB2L1 | 4.45E-68 | 0.712670942 | 0.997 | 0.906 | 1.38E-63 | cDC | GNB2L1 |
| RASSF5 | 1.56E-67 | 0.778452503 | 0.634 | 0.229 | 4.86E-63 | cDC | RASSF5 |
| RPL27A | 2.23E-66 | 0.619185761 | 0.997 | 0.979 | 6.95E-62 | cDC | RPL27A |
| HNRNPA1 | 3.15E-66 | 0.814147134 | 0.98 | 0.778 | 9.79E-62 | cDC | HNRNPA1 |
| RPS91 | 4.07E-65 | 0.545547033 | 0.997 | 0.985 | 1.27E-60 | cDC | RPS9 |
| TCTN3 | 5.89E-65 | 0.504012025 | 0.27 | 0.049 | 1.83E-60 | cDC | TCTN3 |
| RPL91 | 6.15E-65 | 0.585599804 | 0.997 | 0.977 | 1.91E-60 | cDC | RPL9 |
| MALT1 | 1.16E-64 | 0.596574704 | 0.551 | 0.172 | 3.61E-60 | cDC | MALT1 |
| LDLRAD4 | 1.21E-64 | 0.638781376 | 0.557 | 0.175 | 3.76E-60 | cDC | LDLRAD4 |
| CNN2 | 2.50E-64 | 0.665177172 | 0.645 | 0.237 | 7.79E-60 | cDC | CNN2 |
| MCOLN2 | 3.37E-63 | 0.484837744 | 0.33 | 0.071 | 1.05E-58 | cDC | MCOLN2 |
| RPL13A | 1.07E-62 | 0.547105041 | 0.997 | 0.994 | 3.34E-58 | cDC | RPL13A |
| RPL261 | 2.69E-62 | 0.648949915 | 0.997 | 0.974 | 8.38E-58 | cDC | RPL26 |
| HIC1 | 4.24E-62 | 0.389100586 | 0.253 | 0.044 | 1.32E-57 | cDC | HIC1 |
| RPL71 | 8.15E-62 | 0.625936236 | 0.997 | 0.973 | 2.53E-57 | cDC | RPL7 |
| COTL1 | 3.76E-61 | 1.026947472 | 0.972 | 0.786 | 1.17E-56 | cDC | COTL1 |
| RPL14 | 3.90E-61 | 0.64361449 | 0.997 | 0.93 | 1.21E-56 | cDC | RPL14 |
| GDI2 | 1.71E-60 | 0.852069262 | 0.884 | 0.489 | 5.33E-56 | cDC | GDI2 |
| RPL131 | 2.64E-60 | 0.530235921 | 0.997 | 0.996 | 8.21E-56 | cDC | RPL13 |
| RPL321 | 2.85E-60 | 0.564374353 | 1 | 0.989 | 8.86E-56 | cDC | RPL32 |
| SNHG15 | 3.20E-60 | 0.738197388 | 0.588 | 0.21 | 9.95E-56 | cDC | SNHG15 |
| CORO1A | 3.91E-60 | 0.98762485 | 0.855 | 0.505 | 1.22E-55 | cDC | CORO1A |
| CALCRL | 5.77E-60 | 0.29041381 | 0.136 | 0.012 | 1.79E-55 | cDC | CALCRL |
| RPS15A1 | 8.84E-60 | 0.590412852 | 0.997 | 0.981 | 2.75E-55 | cDC | RPS15A |
| EEF1B2 | 1.01E-59 | 0.791914583 | 0.963 | 0.764 | 3.15E-55 | cDC | EEF1B2 |
| AREG1 | 5.79E-59 | 1.462995469 | 0.795 | 0.415 | 1.80E-54 | cDC | AREG |
| RPS27A | 6.82E-59 | 0.523044179 | 0.994 | 0.988 | 2.12E-54 | cDC | RPS27A |
| RPL291 | 7.26E-59 | 0.575129728 | 1 | 0.951 | 2.26E-54 | cDC | RPL29 |
| RPL371 | 1.14E-57 | 0.701419912 | 0.997 | 0.957 | 3.56E-53 | cDC | RPL37 |
| GPR1831 | 1.53E-57 | 1.060709429 | 0.96 | 0.65 | 4.75E-53 | cDC | GPR183 |
| RPL18 | 2.24E-57 | 0.579363494 | 0.991 | 0.95 | 6.96E-53 | cDC | RPL18 |
| TCOF1 | 3.25E-57 | 0.58827457 | 0.438 | 0.127 | 1.01E-52 | cDC | TCOF1 |
| AIM1 | 4.07E-57 | 0.388601487 | 0.355 | 0.087 | 1.27E-52 | cDC | AIM1 |
| RPL31 | 4.70E-57 | 0.682306237 | 0.997 | 0.924 | 1.46E-52 | cDC | RPL31 |
| ACTB | 8.73E-57 | 0.699907678 | 1 | 0.997 | 2.72E-52 | cDC | ACTB |
| PABPC1 | 1.71E-56 | 0.750512145 | 0.983 | 0.854 | 5.33E-52 | cDC | PABPC1 |
| DUSP41 | 1.72E-56 | 0.936756672 | 0.616 | 0.242 | 5.35E-52 | cDC | DUSP4 |
| SERPINB11 | 2.84E-56 | 1.161058481 | 0.895 | 0.604 | 8.85E-52 | cDC | SERPINB1 |
| RPS241 | 6.54E-56 | 0.551307205 | 0.997 | 0.985 | 2.03E-51 | cDC | RPS24 |
| RPS10 | 2.14E-55 | 0.761813167 | 0.983 | 0.844 | 6.66E-51 | cDC | RPS10 |
| SULF2 | 2.77E-55 | 0.345142611 | 0.349 | 0.084 | 8.63E-51 | cDC | SULF2 |
| RPL27 | 2.82E-55 | 0.592896237 | 0.997 | 0.948 | 8.76E-51 | cDC | RPL27 |
| SLC25A6 | 9.33E-55 | 0.741132873 | 0.983 | 0.769 | 2.90E-50 | cDC | SLC25A6 |
| EEF2 | 6.93E-54 | 0.753086509 | 0.966 | 0.718 | 2.16E-49 | cDC | EEF2 |
| HLA-DQB2 | 1.37E-53 | 0.412993499 | 0.216 | 0.037 | 4.25E-49 | cDC | HLA-DQB2 |
| CYTIP | 3.30E-52 | 0.740866674 | 0.574 | 0.223 | 1.03E-47 | cDC | CYTIP |
| FSCN1 | 4.62E-52 | 0.713093723 | 0.315 | 0.078 | 1.44E-47 | cDC | FSCN1 |
| RPL211 | 5.44E-52 | 0.487207497 | 1 | 0.992 | 1.69E-47 | cDC | RPL21 |
| RPS29 | 8.58E-52 | 0.6709153 | 1 | 0.965 | 2.67E-47 | cDC | RPS29 |
| IL1R2 | 1.06E-51 | 0.645417193 | 0.318 | 0.077 | 3.28E-47 | cDC | IL1R2 |
| TMSB10 | 1.06E-51 | 0.569243107 | 1 | 0.999 | 3.30E-47 | cDC | TMSB10 |
| PPP1R16A | 1.20E-51 | 0.316428906 | 0.21 | 0.036 | 3.75E-47 | cDC | PPP1R16A |
| PLEKHA5 | 1.24E-51 | 0.51492039 | 0.173 | 0.025 | 3.86E-47 | cDC | PLEKHA5 |
| RPL35A1 | 1.65E-51 | 0.539198286 | 0.997 | 0.967 | 5.14E-47 | cDC | RPL35A |
| RPL341 | 4.64E-51 | 0.553522043 | 1 | 0.991 | 1.45E-46 | cDC | RPL34 |
| CST31 | 1.09E-50 | 1.00565665 | 0.98 | 0.92 | 3.40E-46 | cDC | CST3 |
| FAM118A | 1.33E-50 | 0.343917107 | 0.222 | 0.041 | 4.15E-46 | cDC | FAM118A |
| PAK1 | 2.16E-50 | 0.551061035 | 0.52 | 0.186 | 6.73E-46 | cDC | PAK1 |
| BID | 2.47E-50 | 0.831408175 | 0.815 | 0.453 | 7.68E-46 | cDC | BID |
| KCNK6 | 7.80E-50 | 0.543035302 | 0.372 | 0.108 | 2.43E-45 | cDC | KCNK6 |
| HLA-DMA1 | 1.68E-49 | 0.713836368 | 0.972 | 0.699 | 5.22E-45 | cDC | HLA-DMA |
| RPL22 | 4.03E-49 | 0.546871268 | 0.997 | 0.911 | 1.26E-44 | cDC | RPL22 |
| INSIG11 | 4.36E-49 | 1.087730158 | 0.798 | 0.455 | 1.36E-44 | cDC | INSIG1 |
| FAU1 | 1.32E-48 | 0.4505055 | 1 | 0.974 | 4.12E-44 | cDC | FAU |
| RFTN1 | 1.45E-48 | 0.404650896 | 0.347 | 0.094 | 4.50E-44 | cDC | RFTN1 |
| RPS131 | 1.91E-48 | 0.523425342 | 0.997 | 0.969 | 5.95E-44 | cDC | RPS13 |
| RPL37A | 1.90E-47 | 0.634219089 | 1 | 0.961 | 5.91E-43 | cDC | RPL37A |
| MARCKSL11 | 3.12E-47 | 0.8361543 | 0.773 | 0.399 | 9.70E-43 | cDC | MARCKSL1 |
| CPVL | 4.65E-47 | 1.000615396 | 0.773 | 0.41 | 1.45E-42 | cDC | CPVL |
| RPS121 | 5.45E-47 | 0.490560853 | 0.997 | 0.988 | 1.69E-42 | cDC | RPS12 |
| HLA-DRB51 | 5.67E-47 | 1.417459158 | 0.884 | 0.673 | 1.76E-42 | cDC | HLA-DRB5 |
| RPS15 | 6.25E-47 | 0.45179161 | 0.997 | 0.99 | 1.95E-42 | cDC | RPS15 |
| RPL301 | 7.01E-47 | 0.514420126 | 1 | 0.958 | 2.18E-42 | cDC | RPL30 |
| FAM110A1 | 7.73E-47 | 0.537798208 | 0.384 | 0.115 | 2.41E-42 | cDC | FAM110A |
| RBM3 | 1.22E-46 | 0.680075489 | 0.923 | 0.6 | 3.81E-42 | cDC | RBM3 |
| CEBPD1 | 3.00E-46 | 0.912179128 | 0.685 | 0.341 | 9.33E-42 | cDC | CEBPD |
| ENTPD11 | 9.51E-46 | 0.609732231 | 0.469 | 0.169 | 2.96E-41 | cDC | ENTPD1 |
| HLA-DQA21 | 1.03E-45 | 1.587583433 | 0.679 | 0.363 | 3.20E-41 | cDC | HLA-DQA2 |
| LIMD2 | 1.83E-45 | 0.599586375 | 0.483 | 0.179 | 5.68E-41 | cDC | LIMD2 |
| ETV3 | 3.62E-45 | 0.531879993 | 0.514 | 0.191 | 1.13E-40 | cDC | ETV3 |
| GLTSCR2 | 4.15E-45 | 0.635733833 | 0.872 | 0.485 | 1.29E-40 | cDC | GLTSCR2 |
| RPL23 | 5.55E-45 | 0.549379165 | 0.994 | 0.934 | 1.73E-40 | cDC | RPL23 |
| FAM60A | 8.56E-45 | 0.38762155 | 0.307 | 0.081 | 2.66E-40 | cDC | FAM60A |
| HMGN1 | 2.04E-44 | 0.635009977 | 0.918 | 0.557 | 6.36E-40 | cDC | HMGN1 |
| LMNB1 | 3.49E-44 | 0.272969864 | 0.264 | 0.061 | 1.08E-39 | cDC | LMNB1 |
| RP11-347P5.1 | 7.43E-44 | 0.337191762 | 0.259 | 0.059 | 2.31E-39 | cDC | RP11-347P5.1 |
| C12orf75 | 1.07E-43 | 0.321963774 | 0.276 | 0.068 | 3.32E-39 | cDC | C12orf75 |
| NR4A3 | 1.33E-43 | 0.740120095 | 0.773 | 0.422 | 4.15E-39 | cDC | NR4A3 |
| RPLP0 | 1.67E-43 | 0.582175004 | 0.986 | 0.878 | 5.21E-39 | cDC | RPLP0 |
| RPL35 | 1.71E-43 | 0.479026032 | 1 | 0.964 | 5.31E-39 | cDC | RPL35 |
| RPS25 | 4.08E-43 | 0.465067333 | 0.997 | 0.964 | 1.27E-38 | cDC | RPS25 |
| EIF3F | 4.95E-43 | 0.623798267 | 0.838 | 0.465 | 1.54E-38 | cDC | EIF3F |
| RPS271 | 5.57E-43 | 0.552272396 | 0.997 | 0.992 | 1.73E-38 | cDC | RPS27 |
| CFL1 | 1.89E-42 | 0.532488985 | 0.994 | 0.935 | 5.90E-38 | cDC | CFL1 |
| MIR181A1HG | 2.19E-42 | 0.286297734 | 0.253 | 0.059 | 6.81E-38 | cDC | MIR181A1HG |
| CACNA2D3 | 3.17E-42 | 0.276111659 | 0.188 | 0.034 | 9.86E-38 | cDC | CACNA2D3 |
| WHAMM | 8.75E-42 | 0.268319346 | 0.244 | 0.056 | 2.72E-37 | cDC | WHAMM |
| SERPINB91 | 4.29E-41 | 0.763377646 | 0.869 | 0.547 | 1.34E-36 | cDC | SERPINB9 |
| RPL281 | 7.28E-41 | 0.387463462 | 1 | 0.995 | 2.27E-36 | cDC | RPL28 |
| CD86 | 7.40E-41 | 0.807393189 | 0.844 | 0.495 | 2.30E-36 | cDC | CD86 |
| TMEM120B | 1.19E-40 | 0.266843831 | 0.199 | 0.04 | 3.72E-36 | cDC | TMEM120B |
| PPHLN1 | 2.05E-40 | 0.368897875 | 0.432 | 0.151 | 6.38E-36 | cDC | PPHLN1 |
| EZR | 2.12E-40 | 0.73852512 | 0.773 | 0.417 | 6.61E-36 | cDC | EZR |
| RPL241 | 2.42E-40 | 0.440265071 | 0.991 | 0.931 | 7.54E-36 | cDC | RPL24 |
| MOB3A | 3.01E-40 | 0.360838088 | 0.452 | 0.16 | 9.36E-36 | cDC | MOB3A |
| TNFRSF4 | 8.54E-40 | 0.535415399 | 0.247 | 0.061 | 2.66E-35 | cDC | TNFRSF4 |
| CD521 | 9.90E-40 | 0.636631542 | 0.77 | 0.405 | 3.08E-35 | cDC | CD52 |
| NFKBID | 1.18E-39 | 0.545096621 | 0.497 | 0.198 | 3.67E-35 | cDC | NFKBID |
| BTF3 | 1.35E-39 | 0.530047313 | 0.98 | 0.843 | 4.20E-35 | cDC | BTF3 |
| AKAP13 | 1.51E-39 | 0.610176827 | 0.764 | 0.408 | 4.69E-35 | cDC | AKAP13 |
| RPLP21 | 1.61E-39 | 0.413184494 | 1 | 0.991 | 5.00E-35 | cDC | RPLP2 |
| TTYH2 | 2.14E-39 | 0.276214625 | 0.267 | 0.068 | 6.66E-35 | cDC | TTYH2 |
| RALA | 4.71E-39 | 0.700200918 | 0.781 | 0.433 | 1.47E-34 | cDC | RALA |
| HNRNPA0 | 1.40E-38 | 0.532943789 | 0.79 | 0.431 | 4.36E-34 | cDC | HNRNPA0 |
| RP5-1171I10.5 | 3.01E-38 | 0.343959375 | 0.361 | 0.114 | 9.36E-34 | cDC | RP5-1171I10.5 |
| PFN1 | 7.01E-38 | 0.481158763 | 1 | 0.968 | 2.18E-33 | cDC | PFN1 |
| SYAP1 | 9.64E-38 | 0.580013158 | 0.668 | 0.319 | 3.00E-33 | cDC | SYAP1 |
| BCL3 | 1.27E-37 | 0.363774251 | 0.403 | 0.139 | 3.95E-33 | cDC | BCL3 |
| CDC37 | 1.91E-37 | 0.541749865 | 0.815 | 0.453 | 5.94E-33 | cDC | CDC37 |
| TNFRSF18 | 2.19E-37 | 0.422421657 | 0.227 | 0.054 | 6.81E-33 | cDC | TNFRSF18 |
| STK17B | 2.30E-37 | 0.500477606 | 0.605 | 0.274 | 7.16E-33 | cDC | STK17B |
| IL2RG | 3.76E-37 | 0.458842668 | 0.608 | 0.264 | 1.17E-32 | cDC | IL2RG |
| N4BP2L1 | 5.02E-37 | 0.459196976 | 0.426 | 0.161 | 1.56E-32 | cDC | N4BP2L1 |
| CMTM6 | 5.44E-37 | 0.607817701 | 0.889 | 0.566 | 1.69E-32 | cDC | CMTM6 |
| RPL41 | 6.25E-37 | 0.481682777 | 0.997 | 0.994 | 1.95E-32 | cDC | RPL41 |
| FNBP1 | 6.37E-37 | 0.547388862 | 0.702 | 0.351 | 1.98E-32 | cDC | FNBP1 |
| CYP2S1 | 8.17E-37 | 0.325792289 | 0.276 | 0.077 | 2.54E-32 | cDC | CYP2S1 |
| PTPN1 | 8.81E-37 | 0.488608052 | 0.688 | 0.329 | 2.74E-32 | cDC | PTPN1 |
| EIF3E | 1.21E-36 | 0.579542406 | 0.869 | 0.541 | 3.77E-32 | cDC | EIF3E |
| DENND1B | 1.44E-36 | 0.321107472 | 0.321 | 0.099 | 4.47E-32 | cDC | DENND1B |
| CLIC2 | 1.69E-36 | 0.343724912 | 0.327 | 0.103 | 5.26E-32 | cDC | CLIC2 |
| ARF5 | 1.78E-36 | 0.498232277 | 0.588 | 0.269 | 5.52E-32 | cDC | ARF5 |
| CUL1 | 3.86E-36 | 0.365375516 | 0.327 | 0.103 | 1.20E-31 | cDC | CUL1 |
| ADAM8 | 1.65E-35 | 0.375326494 | 0.628 | 0.276 | 5.12E-31 | cDC | ADAM8 |
| SLC25A33 | 5.33E-35 | 0.319512656 | 0.403 | 0.145 | 1.66E-30 | cDC | SLC25A33 |
| SRSF2 | 6.82E-35 | 0.590265201 | 0.878 | 0.575 | 2.12E-30 | cDC | SRSF2 |
| FMNL1 | 7.20E-35 | 0.392135998 | 0.58 | 0.255 | 2.24E-30 | cDC | FMNL1 |
| MBOAT7 | 8.07E-35 | 0.336785491 | 0.48 | 0.188 | 2.51E-30 | cDC | MBOAT7 |
| TRAF4 | 1.17E-34 | 0.25761352 | 0.207 | 0.049 | 3.63E-30 | cDC | TRAF4 |
| POGLUT1 | 1.93E-34 | 0.298991632 | 0.179 | 0.038 | 6.01E-30 | cDC | POGLUT1 |
| PDE4A | 3.79E-34 | 0.361172908 | 0.5 | 0.203 | 1.18E-29 | cDC | PDE4A |
| TES | 1.32E-33 | 0.323040335 | 0.44 | 0.168 | 4.10E-29 | cDC | TES |
| RAB11FIP1 | 1.38E-33 | 0.501786779 | 0.381 | 0.14 | 4.30E-29 | cDC | RAB11FIP1 |
| VDR | 1.48E-33 | 0.280316091 | 0.298 | 0.09 | 4.62E-29 | cDC | VDR |
| FBL | 1.53E-33 | 0.412405148 | 0.565 | 0.257 | 4.77E-29 | cDC | FBL |
| ARL4C2 | 1.65E-33 | 0.616071706 | 0.716 | 0.381 | 5.12E-29 | cDC | ARL4C |
| PIM2 | 1.75E-33 | 0.273857138 | 0.261 | 0.073 | 5.43E-29 | cDC | PIM2 |
| ACAA1 | 1.91E-33 | 0.410865857 | 0.543 | 0.243 | 5.94E-29 | cDC | ACAA1 |
| UBA521 | 3.81E-33 | 0.349127793 | 0.997 | 0.98 | 1.19E-28 | cDC | UBA52 |
| EIF3H | 7.17E-33 | 0.481335084 | 0.841 | 0.525 | 2.23E-28 | cDC | EIF3H |
| SUB11 | 8.65E-33 | 0.495853877 | 0.983 | 0.807 | 2.69E-28 | cDC | SUB1 |
| GSN2 | 1.12E-32 | 0.668515159 | 0.798 | 0.49 | 3.49E-28 | cDC | GSN |
| RPL17 | 1.13E-32 | 0.511146077 | 0.801 | 0.474 | 3.51E-28 | cDC | RPL17 |
| IDO11 | 1.15E-32 | 1.068024984 | 0.281 | 0.087 | 3.57E-28 | cDC | IDO1 |
| RPS21 | 1.19E-32 | 0.585495293 | 0.98 | 0.888 | 3.71E-28 | cDC | RPS21 |
| VASP | 2.97E-32 | 0.517507775 | 0.798 | 0.472 | 9.24E-28 | cDC | VASP |
| YPEL5 | 3.26E-32 | 0.718688118 | 0.705 | 0.394 | 1.01E-27 | cDC | YPEL5 |
| HMGA1 | 5.86E-32 | 0.471804064 | 0.719 | 0.395 | 1.82E-27 | cDC | HMGA1 |
| NACA | 1.06E-31 | 0.459186467 | 0.989 | 0.889 | 3.29E-27 | cDC | NACA |
| TRABD | 1.09E-31 | 0.411622573 | 0.548 | 0.255 | 3.39E-27 | cDC | TRABD |
| RPL391 | 1.97E-31 | 0.398020765 | 1 | 0.993 | 6.12E-27 | cDC | RPL39 |
| EDEM1 | 2.57E-31 | 0.430220774 | 0.494 | 0.212 | 8.01E-27 | cDC | EDEM1 |
| NAP1L11 | 5.02E-31 | 0.587782032 | 0.929 | 0.643 | 1.56E-26 | cDC | NAP1L1 |
| TMSB4X2 | 5.60E-31 | 0.435522131 | 1 | 1 | 1.74E-26 | cDC | TMSB4X |
| SPN | 5.97E-31 | 0.270334985 | 0.29 | 0.092 | 1.86E-26 | cDC | SPN |
| CRIP1 | 6.71E-31 | 0.445338671 | 0.548 | 0.243 | 2.09E-26 | cDC | CRIP1 |
| SLC25A3 | 8.51E-31 | 0.544391294 | 0.915 | 0.65 | 2.65E-26 | cDC | SLC25A3 |
| IDH21 | 1.41E-30 | 0.43366046 | 0.605 | 0.292 | 4.37E-26 | cDC | IDH2 |
| SATB1 | 2.07E-30 | 0.425522382 | 0.472 | 0.199 | 6.45E-26 | cDC | SATB1 |
| CIITA | 2.08E-30 | 0.267826575 | 0.298 | 0.097 | 6.46E-26 | cDC | CIITA |
| BCL2L11 | 2.74E-30 | 0.345704439 | 0.304 | 0.102 | 8.52E-26 | cDC | BCL2L11 |
| LST1 | 2.98E-30 | 0.500176666 | 0.974 | 0.806 | 9.27E-26 | cDC | LST1 |
| RGS13 | 9.77E-30 | 0.546862775 | 0.926 | 0.595 | 3.04E-25 | cDC | RGS1 |
| PIP4K2A | 1.01E-29 | 0.284433387 | 0.415 | 0.164 | 3.13E-25 | cDC | PIP4K2A |
| ARPC1B1 | 1.80E-29 | 0.545985696 | 0.955 | 0.751 | 5.60E-25 | cDC | ARPC1B |
| PRMT1 | 1.96E-29 | 0.348956816 | 0.44 | 0.184 | 6.10E-25 | cDC | PRMT1 |
| HNRNPUL1 | 2.86E-29 | 0.349491397 | 0.486 | 0.215 | 8.89E-25 | cDC | HNRNPUL1 |
| GPBP1 | 4.62E-29 | 0.409315332 | 0.673 | 0.343 | 1.44E-24 | cDC | GPBP1 |
| AXL1 | 4.73E-29 | 0.658890838 | 0.438 | 0.194 | 1.47E-24 | cDC | AXL |
| PIM3 | 7.48E-29 | 0.584497314 | 0.79 | 0.494 | 2.33E-24 | cDC | PIM3 |
| RPL36 | 8.97E-29 | 0.439709677 | 0.997 | 0.964 | 2.79E-24 | cDC | RPL36 |
| GABARAPL2 | 9.52E-29 | 0.554798283 | 0.832 | 0.529 | 2.96E-24 | cDC | GABARAPL2 |
| PTP4A2 | 1.10E-28 | 0.444059584 | 0.787 | 0.459 | 3.44E-24 | cDC | PTP4A2 |
| HLA-DMB1 | 1.48E-28 | 0.506829023 | 0.881 | 0.546 | 4.61E-24 | cDC | HLA-DMB |
| PSME1 | 1.73E-28 | 0.515492658 | 0.909 | 0.623 | 5.37E-24 | cDC | PSME1 |
| GPR35 | 1.98E-28 | 0.29718279 | 0.312 | 0.109 | 6.16E-24 | cDC | GPR35 |
| PPA2 | 2.04E-28 | 0.335724041 | 0.503 | 0.227 | 6.36E-24 | cDC | PPA2 |
| BBX | 2.25E-28 | 0.301633903 | 0.406 | 0.165 | 7.01E-24 | cDC | BBX |
| CDC42SE2 | 2.68E-28 | 0.392742167 | 0.517 | 0.242 | 8.35E-24 | cDC | CDC42SE2 |
| ICAM31 | 8.16E-28 | 0.256693459 | 0.293 | 0.098 | 2.54E-23 | cDC | ICAM3 |
| BIRC3 | 1.08E-27 | 0.87633079 | 0.634 | 0.338 | 3.35E-23 | cDC | BIRC3 |
| CCND3 | 1.11E-27 | 0.374186779 | 0.412 | 0.173 | 3.46E-23 | cDC | CCND3 |
| POLR2E | 1.38E-27 | 0.409276617 | 0.679 | 0.361 | 4.29E-23 | cDC | POLR2E |
| BAZ1A | 1.47E-27 | 0.517870472 | 0.73 | 0.414 | 4.56E-23 | cDC | BAZ1A |
| ENSA | 2.63E-27 | 0.407630134 | 0.699 | 0.387 | 8.19E-23 | cDC | ENSA |
| HAPLN3 | 3.37E-27 | 0.354759194 | 0.27 | 0.091 | 1.05E-22 | cDC | HAPLN3 |
| EIF3I | 5.19E-27 | 0.360225704 | 0.702 | 0.376 | 1.62E-22 | cDC | EIF3I |
| RBM39 | 5.62E-27 | 0.430225352 | 0.807 | 0.499 | 1.75E-22 | cDC | RBM39 |
| EBI3 | 7.01E-27 | 0.537989697 | 0.213 | 0.063 | 2.18E-22 | cDC | EBI3 |
| IFI30 | 8.94E-27 | 0.420149901 | 0.724 | 0.413 | 2.78E-22 | cDC | IFI30 |
| RBMX | 1.15E-26 | 0.326133941 | 0.577 | 0.283 | 3.56E-22 | cDC | RBMX |
| ID2 | 1.59E-26 | 0.92819124 | 0.864 | 0.69 | 4.95E-22 | cDC | ID2 |
| SMDT1 | 1.67E-26 | 0.415900639 | 0.764 | 0.428 | 5.21E-22 | cDC | SMDT1 |
| ATF5 | 2.26E-26 | 0.547740473 | 0.597 | 0.315 | 7.04E-22 | cDC | ATF5 |
| MTHFD2 | 2.68E-26 | 0.525153152 | 0.676 | 0.388 | 8.35E-22 | cDC | MTHFD2 |
| CXCR4 | 4.29E-26 | 0.857607349 | 0.699 | 0.431 | 1.33E-21 | cDC | CXCR4 |
| SMAP2 | 6.48E-26 | 0.298606137 | 0.506 | 0.236 | 2.02E-21 | cDC | SMAP2 |
| HINT11 | 7.05E-26 | 0.443967467 | 0.966 | 0.716 | 2.19E-21 | cDC | HINT1 |
| ARF6 | 8.87E-26 | 0.428219938 | 0.713 | 0.41 | 2.76E-21 | cDC | ARF6 |
| TOR3A1 | 9.42E-26 | 0.266604293 | 0.361 | 0.144 | 2.93E-21 | cDC | TOR3A |
| CREM1 | 1.62E-25 | 0.753394532 | 0.847 | 0.704 | 5.03E-21 | cDC | CREM |
| PTRHD1 | 4.84E-25 | 0.297780531 | 0.486 | 0.226 | 1.50E-20 | cDC | PTRHD1 |
| PNISR | 7.29E-25 | 0.317551108 | 0.551 | 0.269 | 2.27E-20 | cDC | PNISR |
| DNAJC4 | 8.79E-25 | 0.254486633 | 0.392 | 0.165 | 2.74E-20 | cDC | DNAJC4 |
| EIF3D | 1.14E-24 | 0.345812628 | 0.602 | 0.314 | 3.54E-20 | cDC | EIF3D |
| PRELID11 | 1.43E-24 | 0.567540086 | 0.889 | 0.669 | 4.45E-20 | cDC | PRELID1 |
| CD80 | 1.53E-24 | 0.353864699 | 0.327 | 0.128 | 4.75E-20 | cDC | CD80 |
| TNIP2 | 2.11E-24 | 0.292455741 | 0.423 | 0.186 | 6.56E-20 | cDC | TNIP2 |
| WBSCR22 | 2.15E-24 | 0.273641083 | 0.44 | 0.198 | 6.68E-20 | cDC | WBSCR22 |
| GNA151 | 2.56E-24 | 0.455869776 | 0.639 | 0.356 | 7.97E-20 | cDC | GNA15 |
| IFITM21 | 3.14E-24 | 0.537393415 | 0.744 | 0.458 | 9.78E-20 | cDC | IFITM2 |
| RP11-138A9.2 | 5.22E-24 | 0.301048882 | 0.259 | 0.089 | 1.63E-19 | cDC | RP11-138A9.2 |
| QPCT1 | 5.30E-24 | 0.309257062 | 0.335 | 0.133 | 1.65E-19 | cDC | QPCT |
| SLC25A19 | 6.17E-24 | 0.452559243 | 0.446 | 0.216 | 1.92E-19 | cDC | SLC25A19 |
| ARID5A | 7.53E-24 | 0.433707706 | 0.599 | 0.322 | 2.34E-19 | cDC | ARID5A |
| FAM49A | 9.32E-24 | 0.377342335 | 0.631 | 0.338 | 2.90E-19 | cDC | FAM49A |
| PTTG1 | 1.28E-23 | 0.261326305 | 0.298 | 0.112 | 3.98E-19 | cDC | PTTG1 |
| NQO2 | 1.85E-23 | 0.422722462 | 0.54 | 0.278 | 5.74E-19 | cDC | NQO2 |
| EIF3M | 1.95E-23 | 0.348056871 | 0.696 | 0.388 | 6.07E-19 | cDC | EIF3M |
| REV3L | 3.82E-23 | 0.252631011 | 0.318 | 0.122 | 1.19E-18 | cDC | REV3L |
| NPM1 | 3.90E-23 | 0.471557058 | 0.949 | 0.735 | 1.21E-18 | cDC | NPM1 |
| RPL38 | 4.09E-23 | 0.413060772 | 0.994 | 0.933 | 1.27E-18 | cDC | RPL38 |
| CIB1 | 5.95E-23 | 0.462790991 | 0.835 | 0.602 | 1.85E-18 | cDC | CIB1 |
| OSTC | 6.17E-23 | 0.387568228 | 0.767 | 0.455 | 1.92E-18 | cDC | OSTC |
| RSL24D1 | 9.65E-23 | 0.327882552 | 0.622 | 0.344 | 3.00E-18 | cDC | RSL24D1 |
| EID11 | 1.19E-22 | 0.367975599 | 0.724 | 0.417 | 3.70E-18 | cDC | EID1 |
| PTMA | 1.20E-22 | 0.336257445 | 0.997 | 0.979 | 3.72E-18 | cDC | PTMA |
| RPL36A | 1.31E-22 | 0.530973735 | 0.912 | 0.701 | 4.07E-18 | cDC | RPL36A |
| WAS | 1.66E-22 | 0.30206377 | 0.509 | 0.252 | 5.15E-18 | cDC | WAS |
| ARID4B | 1.86E-22 | 0.256984496 | 0.497 | 0.236 | 5.78E-18 | cDC | ARID4B |
| RAB4B | 1.93E-22 | 0.261999281 | 0.332 | 0.137 | 6.00E-18 | cDC | RAB4B |
| CKLF | 2.02E-22 | 0.552943386 | 0.79 | 0.54 | 6.27E-18 | cDC | CKLF |
| EIF2A | 2.03E-22 | 0.254057152 | 0.42 | 0.19 | 6.30E-18 | cDC | EIF2A |
| ACTG1 | 2.12E-22 | 0.54985261 | 0.986 | 0.931 | 6.60E-18 | cDC | ACTG1 |
| MAD2L2 | 2.20E-22 | 0.255607543 | 0.401 | 0.178 | 6.85E-18 | cDC | MAD2L2 |
| MYADM | 2.59E-22 | 0.35652657 | 0.545 | 0.279 | 8.06E-18 | cDC | MYADM |
| DDOST1 | 3.02E-22 | 0.290342738 | 0.491 | 0.244 | 9.39E-18 | cDC | DDOST |
| THEMIS21 | 3.21E-22 | 0.348102572 | 0.622 | 0.328 | 9.99E-18 | cDC | THEMIS2 |
| PEA15 | 3.40E-22 | 0.385731986 | 0.699 | 0.408 | 1.06E-17 | cDC | PEA15 |
| ANKRD11 | 4.99E-22 | 0.325851593 | 0.415 | 0.191 | 1.55E-17 | cDC | ANKRD11 |
| BZW2 | 5.06E-22 | 0.262050814 | 0.304 | 0.122 | 1.57E-17 | cDC | BZW2 |
| SNRPB | 5.34E-22 | 0.38211831 | 0.804 | 0.503 | 1.66E-17 | cDC | SNRPB |
| FAM129A | 6.15E-22 | 0.273038789 | 0.332 | 0.136 | 1.91E-17 | cDC | FAM129A |
| PGLS | 6.72E-22 | 0.358598065 | 0.716 | 0.417 | 2.09E-17 | cDC | PGLS |
| UBE2F | 6.95E-22 | 0.3769812 | 0.554 | 0.299 | 2.16E-17 | cDC | UBE2F |
| CLNS1A | 7.31E-22 | 0.321213495 | 0.474 | 0.233 | 2.28E-17 | cDC | CLNS1A |
| ZNF90 | 7.83E-22 | 0.263075833 | 0.426 | 0.197 | 2.44E-17 | cDC | ZNF90 |
| JARID21 | 1.06E-21 | 0.31849008 | 0.503 | 0.248 | 3.30E-17 | cDC | JARID2 |
| CDKN1A1 | 1.56E-21 | 0.442065391 | 0.81 | 0.54 | 4.84E-17 | cDC | CDKN1A |
| PSMA2 | 1.60E-21 | 0.369698098 | 0.707 | 0.414 | 4.97E-17 | cDC | PSMA2 |
| PRMT91 | 1.68E-21 | 0.288022945 | 0.335 | 0.142 | 5.21E-17 | cDC | PRMT9 |
| CHST2 | 1.70E-21 | 0.2884937 | 0.267 | 0.101 | 5.30E-17 | cDC | CHST2 |
| HMHA1 | 1.97E-21 | 0.268464672 | 0.352 | 0.152 | 6.12E-17 | cDC | HMHA1 |
| C10orf128 | 2.23E-21 | 0.271395232 | 0.352 | 0.152 | 6.94E-17 | cDC | C10orf128 |
| DBNL1 | 3.95E-21 | 0.343840585 | 0.653 | 0.369 | 1.23E-16 | cDC | DBNL |
| CHD1 | 4.17E-21 | 0.280608106 | 0.474 | 0.233 | 1.30E-16 | cDC | CHD1 |
| RPL12 | 4.38E-21 | 0.311295099 | 0.997 | 0.981 | 1.36E-16 | cDC | RPL12 |
| IL4I11 | 4.55E-21 | 0.71100905 | 0.554 | 0.316 | 1.41E-16 | cDC | IL4I1 |
| DDX21 | 6.07E-21 | 0.338589238 | 0.696 | 0.409 | 1.89E-16 | cDC | DDX21 |
| PPM1G | 6.45E-21 | 0.259575745 | 0.42 | 0.197 | 2.01E-16 | cDC | PPM1G |
| CD401 | 7.33E-21 | 0.545439169 | 0.554 | 0.308 | 2.28E-16 | cDC | CD40 |
| ILF2 | 8.99E-21 | 0.256353871 | 0.491 | 0.25 | 2.80E-16 | cDC | ILF2 |
| C1QBP1 | 1.22E-20 | 0.334878142 | 0.602 | 0.335 | 3.80E-16 | cDC | C1QBP |
| EIF6 | 1.28E-20 | 0.286042617 | 0.71 | 0.408 | 3.99E-16 | cDC | EIF6 |
| DNASE1L31 | 1.28E-20 | 0.503752727 | 0.136 | 0.035 | 3.99E-16 | cDC | DNASE1L3 |
| DENND4A | 1.56E-20 | 0.261961319 | 0.344 | 0.147 | 4.87E-16 | cDC | DENND4A |
| MZT2A | 1.67E-20 | 0.275414518 | 0.528 | 0.265 | 5.20E-16 | cDC | MZT2A |
| NFE2L2 | 1.83E-20 | 0.416350682 | 0.804 | 0.526 | 5.68E-16 | cDC | NFE2L2 |
| CSRNP1 | 1.83E-20 | 0.353490687 | 0.616 | 0.344 | 5.70E-16 | cDC | CSRNP1 |
| FCGR2B | 1.84E-20 | 0.370034132 | 0.565 | 0.308 | 5.71E-16 | cDC | FCGR2B |
| APRT | 1.86E-20 | 0.385669618 | 0.849 | 0.562 | 5.78E-16 | cDC | APRT |
| EIF11 | 2.03E-20 | 0.290250217 | 1 | 0.981 | 6.31E-16 | cDC | EIF1 |
| RTFDC1 | 2.07E-20 | 0.310895042 | 0.531 | 0.282 | 6.44E-16 | cDC | RTFDC1 |
| CAT | 2.41E-20 | 0.263683883 | 0.426 | 0.206 | 7.49E-16 | cDC | CAT |
| NFKB2 | 2.68E-20 | 0.261057457 | 0.497 | 0.247 | 8.34E-16 | cDC | NFKB2 |
| CLECL1 | 3.18E-20 | 0.253435255 | 0.222 | 0.078 | 9.89E-16 | cDC | CLECL1 |
| MAP3K13 | 3.74E-20 | 0.334458588 | 0.455 | 0.223 | 1.16E-15 | cDC | MAP3K13 |
| CDK2AP2 | 5.14E-20 | 0.277233314 | 0.398 | 0.188 | 1.60E-15 | cDC | CDK2AP2 |
| GSTP11 | 5.71E-20 | 0.427448856 | 0.98 | 0.789 | 1.78E-15 | cDC | GSTP1 |
| ANXA11 | 5.77E-20 | 0.351581439 | 0.673 | 0.387 | 1.79E-15 | cDC | ANXA11 |
| FKBP1A | 7.60E-20 | 0.382691221 | 0.935 | 0.729 | 2.37E-15 | cDC | FKBP1A |
| OPN3 | 8.08E-20 | 0.313724766 | 0.347 | 0.156 | 2.51E-15 | cDC | OPN3 |
| BRD2 | 8.19E-20 | 0.251504396 | 0.645 | 0.35 | 2.55E-15 | cDC | BRD2 |
| PHB2 | 1.07E-19 | 0.269607872 | 0.48 | 0.247 | 3.33E-15 | cDC | PHB2 |
| ARPC2 | 1.13E-19 | 0.403955131 | 0.986 | 0.874 | 3.52E-15 | cDC | ARPC2 |
| CTSH1 | 1.29E-19 | 0.412917723 | 0.901 | 0.65 | 4.02E-15 | cDC | CTSH |
| SUPT4H1 | 2.12E-19 | 0.339643279 | 0.682 | 0.407 | 6.58E-15 | cDC | SUPT4H1 |
| IDI1 | 2.29E-19 | 0.350317415 | 0.398 | 0.19 | 7.13E-15 | cDC | IDI1 |
| TUBA1A | 2.93E-19 | 0.496106447 | 0.574 | 0.332 | 9.12E-15 | cDC | TUBA1A |
| TNFAIP81 | 3.21E-19 | 0.492597357 | 0.719 | 0.472 | 9.97E-15 | cDC | TNFAIP8 |
| PTPRC | 3.37E-19 | 0.331379418 | 0.83 | 0.531 | 1.05E-14 | cDC | PTPRC |
| HGSNAT | 4.41E-19 | 0.280106909 | 0.361 | 0.167 | 1.37E-14 | cDC | HGSNAT |
| STX111 | 8.42E-19 | 0.429419116 | 0.764 | 0.511 | 2.62E-14 | cDC | STX11 |
| RALY | 8.69E-19 | 0.279307784 | 0.599 | 0.339 | 2.70E-14 | cDC | RALY |
| EIF3K | 9.27E-19 | 0.366643112 | 0.926 | 0.687 | 2.88E-14 | cDC | EIF3K |
| RIPK21 | 1.12E-18 | 0.322136745 | 0.594 | 0.339 | 3.50E-14 | cDC | RIPK2 |
| SYNGR21 | 1.41E-18 | 0.38468823 | 0.759 | 0.456 | 4.38E-14 | cDC | SYNGR2 |
| ATP5G1 | 1.43E-18 | 0.257046663 | 0.645 | 0.363 | 4.46E-14 | cDC | ATP5G1 |
| THOC7 | 1.47E-18 | 0.25142258 | 0.528 | 0.283 | 4.58E-14 | cDC | THOC7 |
| DDX46 | 2.14E-18 | 0.263414972 | 0.489 | 0.255 | 6.65E-14 | cDC | DDX46 |
| PDCL3 | 2.42E-18 | 0.277823945 | 0.406 | 0.201 | 7.53E-14 | cDC | PDCL3 |
| RAB9A | 2.82E-18 | 0.459337894 | 0.401 | 0.206 | 8.78E-14 | cDC | RAB9A |
| TRAF11 | 3.82E-18 | 0.328095258 | 0.44 | 0.223 | 1.19E-13 | cDC | TRAF1 |
| RPS281 | 4.31E-18 | 0.315462012 | 1 | 0.982 | 1.34E-13 | cDC | RPS28 |
| ATP5B1 | 4.51E-18 | 0.320237423 | 0.847 | 0.564 | 1.40E-13 | cDC | ATP5B |
| ELF1 | 4.75E-18 | 0.288232162 | 0.631 | 0.352 | 1.48E-13 | cDC | ELF1 |
| C12orf45 | 5.77E-18 | 0.286424961 | 0.247 | 0.101 | 1.80E-13 | cDC | C12orf45 |
| SKIL | 5.86E-18 | 0.295650649 | 0.531 | 0.282 | 1.82E-13 | cDC | SKIL |
| GLIPR1 | 5.88E-18 | 0.326942713 | 0.71 | 0.436 | 1.83E-13 | cDC | GLIPR1 |
| EIF5A | 6.03E-18 | 0.313929006 | 0.693 | 0.417 | 1.88E-13 | cDC | EIF5A |
| FAM49B | 6.24E-18 | 0.324219087 | 0.901 | 0.627 | 1.94E-13 | cDC | FAM49B |
| PIM1 | 7.70E-18 | 0.371989084 | 0.423 | 0.218 | 2.40E-13 | cDC | PIM1 |
| PSME21 | 7.77E-18 | 0.449519339 | 0.884 | 0.644 | 2.42E-13 | cDC | PSME2 |
| DNAJB11 | 2.01E-17 | 0.283804254 | 0.602 | 0.34 | 6.26E-13 | cDC | DNAJB11 |
| TAP1 | 2.24E-17 | 0.458387793 | 0.503 | 0.282 | 6.97E-13 | cDC | TAP1 |
| CLTB | 2.41E-17 | 0.273879549 | 0.614 | 0.356 | 7.49E-13 | cDC | CLTB |
| UCP21 | 2.49E-17 | 0.394669775 | 0.756 | 0.49 | 7.73E-13 | cDC | UCP2 |
| SLC25A51 | 2.82E-17 | 0.353415567 | 0.912 | 0.646 | 8.76E-13 | cDC | SLC25A5 |
| NDUFA121 | 2.88E-17 | 0.261377167 | 0.614 | 0.356 | 8.95E-13 | cDC | NDUFA12 |
| AC090498.1 | 4.09E-17 | 0.361314133 | 0.693 | 0.441 | 1.27E-12 | cDC | AC090498.1 |
| ARPC4 | 4.44E-17 | 0.333059248 | 0.852 | 0.586 | 1.38E-12 | cDC | ARPC4 |
| CCNI | 4.45E-17 | 0.374915441 | 0.906 | 0.688 | 1.38E-12 | cDC | CCNI |
| NAAA1 | 4.68E-17 | 0.270610234 | 0.406 | 0.204 | 1.46E-12 | cDC | NAAA |
| MAP2K11 | 5.14E-17 | 0.301502036 | 0.599 | 0.344 | 1.60E-12 | cDC | MAP2K1 |
| ACADVL | 5.88E-17 | 0.2773432 | 0.619 | 0.357 | 1.83E-12 | cDC | ACADVL |
| TPM3 | 5.96E-17 | 0.343386576 | 0.946 | 0.743 | 1.86E-12 | cDC | TPM3 |
| H3F3A1 | 6.67E-17 | 0.269731804 | 0.994 | 0.972 | 2.08E-12 | cDC | H3F3A |
| RSL1D1 | 8.72E-17 | 0.27771163 | 0.511 | 0.285 | 2.71E-12 | cDC | RSL1D1 |
| HMGN3 | 8.72E-17 | 0.270593617 | 0.67 | 0.399 | 2.71E-12 | cDC | HMGN3 |
| MDH2 | 8.81E-17 | 0.270542927 | 0.619 | 0.362 | 2.74E-12 | cDC | MDH2 |
| TUFM1 | 8.94E-17 | 0.269745842 | 0.636 | 0.37 | 2.78E-12 | cDC | TUFM |
| PARK71 | 1.07E-16 | 0.330391627 | 0.827 | 0.551 | 3.32E-12 | cDC | PARK7 |
| XRCC6 | 1.25E-16 | 0.254964454 | 0.577 | 0.33 | 3.89E-12 | cDC | XRCC6 |
| LDHB1 | 1.46E-16 | 0.284503207 | 0.645 | 0.377 | 4.54E-12 | cDC | LDHB |
| SELK1 | 1.58E-16 | 0.555025296 | 0.824 | 0.62 | 4.92E-12 | cDC | SELK |
| ATG3 | 1.61E-16 | 0.301331721 | 0.665 | 0.412 | 5.01E-12 | cDC | ATG3 |
| ATP5A11 | 1.64E-16 | 0.302150973 | 0.645 | 0.385 | 5.12E-12 | cDC | ATP5A1 |
| PSMB6 | 2.17E-16 | 0.277921738 | 0.773 | 0.473 | 6.76E-12 | cDC | PSMB6 |
| YBX1 | 2.23E-16 | 0.309020396 | 0.989 | 0.895 | 6.93E-12 | cDC | YBX1 |
| AOAH1 | 2.36E-16 | 0.262208567 | 0.364 | 0.178 | 7.33E-12 | cDC | AOAH |
| S100B1 | 3.44E-16 | 0.909450863 | 0.278 | 0.126 | 1.07E-11 | cDC | S100B |
| SAMHD11 | 3.52E-16 | 0.320917341 | 0.747 | 0.478 | 1.10E-11 | cDC | SAMHD1 |
| H2AFV | 3.81E-16 | 0.268305658 | 0.733 | 0.461 | 1.19E-11 | cDC | H2AFV |
| SARAF | 4.20E-16 | 0.350487579 | 0.884 | 0.647 | 1.31E-11 | cDC | SARAF |
| SEC61B | 4.24E-16 | 0.380305315 | 0.943 | 0.779 | 1.32E-11 | cDC | SEC61B |
| NCL | 4.25E-16 | 0.303376096 | 0.705 | 0.442 | 1.32E-11 | cDC | NCL |
| TOB1 | 4.82E-16 | 0.261252319 | 0.318 | 0.15 | 1.50E-11 | cDC | TOB1 |
| HADHA | 6.04E-16 | 0.253027105 | 0.5 | 0.28 | 1.88E-11 | cDC | HADHA |
| TAGLN2 | 7.23E-16 | 0.367599784 | 0.901 | 0.693 | 2.25E-11 | cDC | TAGLN2 |
| LMNA2 | 9.53E-16 | 0.411487059 | 0.776 | 0.53 | 2.97E-11 | cDC | LMNA |
| AVPI1 | 1.30E-15 | 0.302823263 | 0.426 | 0.228 | 4.05E-11 | cDC | AVPI1 |
| CD83 | 1.33E-15 | 0.675089609 | 0.875 | 0.733 | 4.14E-11 | cDC | CD83 |
| SERBP1 | 1.42E-15 | 0.283496463 | 0.716 | 0.446 | 4.40E-11 | cDC | SERBP1 |
| RPS20 | 1.51E-15 | 0.254882514 | 1 | 0.976 | 4.70E-11 | cDC | RPS20 |
| YWHAQ | 1.58E-15 | 0.259874858 | 0.625 | 0.367 | 4.92E-11 | cDC | YWHAQ |
| CLEC4A | 1.71E-15 | 0.275513786 | 0.554 | 0.317 | 5.32E-11 | cDC | CLEC4A |
| SIAH2 | 2.61E-15 | 0.265187113 | 0.338 | 0.165 | 8.13E-11 | cDC | SIAH2 |
| TAPBP | 2.85E-15 | 0.287068985 | 0.75 | 0.474 | 8.87E-11 | cDC | TAPBP |
| PRRC2C | 3.05E-15 | 0.260618218 | 0.668 | 0.414 | 9.49E-11 | cDC | PRRC2C |
| CD58 | 3.20E-15 | 0.283050789 | 0.534 | 0.309 | 9.97E-11 | cDC | CD58 |
| PSMA1 | 3.24E-15 | 0.271356929 | 0.784 | 0.517 | 1.01E-10 | cDC | PSMA1 |
| UBE2D3 | 5.20E-15 | 0.338564834 | 0.955 | 0.757 | 1.62E-10 | cDC | UBE2D3 |
| CCT8 | 5.67E-15 | 0.270390004 | 0.565 | 0.331 | 1.76E-10 | cDC | CCT8 |
| COMMD6 | 6.49E-15 | 0.299652752 | 0.895 | 0.691 | 2.02E-10 | cDC | COMMD6 |
| HCLS1 | 6.97E-15 | 0.296812838 | 0.693 | 0.437 | 2.17E-10 | cDC | HCLS1 |
| PSMB10 | 7.48E-15 | 0.279935125 | 0.446 | 0.249 | 2.33E-10 | cDC | PSMB10 |
| NBEAL1 | 8.01E-15 | 0.266275464 | 0.696 | 0.439 | 2.49E-10 | cDC | NBEAL1 |
| HNRNPA2B1 | 9.15E-15 | 0.304034255 | 0.932 | 0.759 | 2.85E-10 | cDC | HNRNPA2B1 |
| SRSF51 | 1.02E-14 | 0.269892387 | 0.872 | 0.592 | 3.19E-10 | cDC | SRSF5 |
| SLC31A2 | 1.62E-14 | 0.268387139 | 0.597 | 0.353 | 5.06E-10 | cDC | SLC31A2 |
| SET | 1.82E-14 | 0.272298682 | 0.716 | 0.457 | 5.66E-10 | cDC | SET |
| TWF21 | 1.97E-14 | 0.266179693 | 0.574 | 0.342 | 6.14E-10 | cDC | TWF2 |
| RILPL21 | 2.01E-14 | 0.320591314 | 0.756 | 0.505 | 6.24E-10 | cDC | RILPL2 |
| ISG201 | 2.17E-14 | 0.334359413 | 0.551 | 0.33 | 6.76E-10 | cDC | ISG20 |
| RHOG1 | 2.53E-14 | 0.272362179 | 0.812 | 0.554 | 7.88E-10 | cDC | RHOG |
| GADD45A | 3.37E-14 | 0.334425328 | 0.304 | 0.147 | 1.05E-09 | cDC | GADD45A |
| GPR137B1 | 6.75E-14 | 0.447358943 | 0.545 | 0.341 | 2.10E-09 | cDC | GPR137B |
| CIRBP | 7.72E-14 | 0.303791983 | 0.898 | 0.63 | 2.40E-09 | cDC | CIRBP |
| ATP1B1 | 8.99E-14 | 0.299425281 | 0.455 | 0.26 | 2.80E-09 | cDC | ATP1B1 |
| PLSCR11 | 1.07E-13 | 0.278174079 | 0.818 | 0.568 | 3.34E-09 | cDC | PLSCR1 |
| CLIC1 | 1.27E-13 | 0.284941822 | 0.957 | 0.817 | 3.94E-09 | cDC | CLIC1 |
| GPR65 | 1.55E-13 | 0.267277682 | 0.31 | 0.153 | 4.84E-09 | cDC | GPR65 |
| ATP5G2 | 2.23E-13 | 0.27541475 | 0.972 | 0.787 | 6.94E-09 | cDC | ATP5G2 |
| MAFF | 2.53E-13 | 0.271817387 | 0.611 | 0.374 | 7.87E-09 | cDC | MAFF |
| SRSF3 | 4.10E-13 | 0.308029253 | 0.844 | 0.601 | 1.28E-08 | cDC | SRSF3 |
| RNH11 | 4.39E-13 | 0.257470797 | 0.75 | 0.487 | 1.37E-08 | cDC | RNH1 |
| IRF1 | 5.04E-13 | 0.367156514 | 0.557 | 0.345 | 1.57E-08 | cDC | IRF1 |
| TMA7 | 5.96E-13 | 0.275935927 | 0.935 | 0.74 | 1.85E-08 | cDC | TMA7 |
| COPE | 6.04E-13 | 0.256712083 | 0.866 | 0.601 | 1.88E-08 | cDC | COPE |
| PDIA3 | 7.12E-13 | 0.309223757 | 0.847 | 0.623 | 2.22E-08 | cDC | PDIA3 |
| ARPC5 | 1.26E-12 | 0.271251015 | 0.946 | 0.741 | 3.93E-08 | cDC | ARPC5 |
| ARPC3 | 1.55E-12 | 0.278612 | 0.986 | 0.87 | 4.83E-08 | cDC | ARPC3 |
| RGS101 | 3.95E-12 | 0.297491029 | 0.872 | 0.64 | 1.23E-07 | cDC | RGS10 |
| SAMSN11 | 4.48E-12 | 0.383806558 | 0.759 | 0.564 | 1.40E-07 | cDC | SAMSN1 |
| AP1S21 | 7.61E-12 | 0.255403357 | 0.67 | 0.434 | 2.37E-07 | cDC | AP1S2 |
| WARS1 | 9.73E-12 | 0.278971506 | 0.543 | 0.337 | 3.03E-07 | cDC | WARS |
| EDF1 | 9.89E-12 | 0.250295624 | 0.932 | 0.713 | 3.08E-07 | cDC | EDF1 |
| CHMP4B | 1.14E-11 | 0.253096374 | 0.75 | 0.516 | 3.54E-07 | cDC | CHMP4B |
| ZFP36 | 1.54E-11 | 0.331247274 | 0.901 | 0.727 | 4.80E-07 | cDC | ZFP36 |
| LYSMD2 | 1.88E-11 | 0.275388877 | 0.432 | 0.262 | 5.84E-07 | cDC | LYSMD2 |
| GADD45B | 2.18E-11 | 0.342297834 | 0.866 | 0.637 | 6.79E-07 | cDC | GADD45B |
| PPP1CB | 3.47E-11 | 0.25811388 | 0.727 | 0.513 | 1.08E-06 | cDC | PPP1CB |
| TSC22D3 | 4.55E-11 | 0.293692853 | 0.688 | 0.461 | 1.42E-06 | cDC | TSC22D3 |
| CYCS | 6.55E-11 | 0.257816984 | 0.909 | 0.694 | 2.04E-06 | cDC | CYCS |
| MTDH1 | 1.10E-10 | 0.257375336 | 0.759 | 0.53 | 3.42E-06 | cDC | MTDH |
| CFLAR1 | 1.11E-10 | 0.276928205 | 0.869 | 0.678 | 3.44E-06 | cDC | CFLAR |
| ARHGDIB1 | 1.74E-10 | 0.291266267 | 0.886 | 0.696 | 5.41E-06 | cDC | ARHGDIB |
| RGS21 | 2.56E-10 | 0.262641681 | 0.798 | 0.582 | 7.96E-06 | cDC | RGS2 |
| ITGB22 | 2.56E-10 | 0.289158987 | 0.872 | 0.692 | 7.97E-06 | cDC | ITGB2 |
| ACTR3 | 3.16E-10 | 0.261218196 | 0.847 | 0.629 | 9.84E-06 | cDC | ACTR3 |
| SNX3 | 5.90E-10 | 0.298198792 | 0.915 | 0.715 | 1.83E-05 | cDC | SNX3 |
| MIR155HG | 6.21E-10 | 0.363212655 | 0.44 | 0.276 | 1.93E-05 | cDC | MIR155HG |
| ATP1B31 | 7.63E-10 | 0.409817868 | 0.903 | 0.739 | 2.37E-05 | cDC | ATP1B3 |
| PSMB81 | 7.68E-10 | 0.256549254 | 0.608 | 0.405 | 2.39E-05 | cDC | PSMB8 |
| IFITM31 | 1.20E-08 | 0.369851572 | 0.886 | 0.748 | 0.000372188 | cDC | IFITM3 |
| RGCC | 1.33E-08 | 0.324323951 | 0.682 | 0.51 | 0.000412817 | cDC | RGCC |
| HSPA81 | 1.37E-08 | 0.286606641 | 0.864 | 0.659 | 0.000426083 | cDC | HSPA8 |
| PHLDA21 | 2.96E-08 | 0.338120833 | 0.636 | 0.451 | 0.000921124 | cDC | PHLDA2 |
| IL7R | 4.10E-08 | 0.379783681 | 0.506 | 0.356 | 0.001274369 | cDC | IL7R |
| ACOT7 | 1.35E-07 | 0.362939745 | 0.17 | 0.085 | 0.004197593 | cDC | ACOT7 |
| IGHG11 | 4.94E-07 | 0.559811359 | 0.395 | 0.245 | 0.015383777 | cDC | IGHG1 |
| KIAA0101 | 0 | 2.541877001 | 0.879 | 0.033 | 0 | Proliferating | KIAA0101 |
| TYMS | 0 | 1.725043336 | 0.782 | 0.017 | 0 | Proliferating | TYMS |
| TK1 | 0 | 1.676096726 | 0.727 | 0.036 | 0 | Proliferating | TK1 |
| BIRC5 | 0 | 1.487619898 | 0.691 | 0.017 | 0 | Proliferating | BIRC5 |
| RRM2 | 0 | 1.474945195 | 0.624 | 0.011 | 0 | Proliferating | RRM2 |
| TOP2A | 0 | 1.428150339 | 0.527 | 0.01 | 0 | Proliferating | TOP2A |
| CENPM | 0 | 1.200709075 | 0.636 | 0.014 | 0 | Proliferating | CENPM |
| MKI67 | 0 | 1.177457776 | 0.582 | 0.011 | 0 | Proliferating | MKI67 |
| HMMR | 0 | 0.831277148 | 0.376 | 0.005 | 0 | Proliferating | HMMR |
| TPX2 | 3.44E-304 | 0.734733558 | 0.358 | 0.005 | 1.07E-299 | Proliferating | TPX2 |
| MYBL2 | 6.25E-301 | 0.538459225 | 0.376 | 0.006 | 1.95E-296 | Proliferating | MYBL2 |
| ZWINT | 1.16E-300 | 1.114119848 | 0.618 | 0.027 | 3.60E-296 | Proliferating | ZWINT |
| CEP55 | 1.87E-300 | 0.630216721 | 0.364 | 0.006 | 5.82E-296 | Proliferating | CEP55 |
| SPC25 | 2.24E-294 | 0.530315338 | 0.291 | 0.002 | 6.96E-290 | Proliferating | SPC25 |
| UBE2C | 3.24E-284 | 1.634015151 | 0.6 | 0.028 | 1.01E-279 | Proliferating | UBE2C |
| CDKN3 | 3.78E-272 | 1.043501437 | 0.485 | 0.017 | 1.18E-267 | Proliferating | CDKN3 |
| ANLN | 1.09E-271 | 0.575640612 | 0.327 | 0.005 | 3.39E-267 | Proliferating | ANLN |
| GTSE1 | 7.79E-257 | 0.532150712 | 0.279 | 0.003 | 2.42E-252 | Proliferating | GTSE1 |
| CDK1 | 9.44E-254 | 1.385197752 | 0.642 | 0.038 | 2.94E-249 | Proliferating | CDK1 |
| AURKB | 1.15E-253 | 0.527586553 | 0.376 | 0.009 | 3.58E-249 | Proliferating | AURKB |
| PLK1 | 2.01E-249 | 0.522529369 | 0.273 | 0.003 | 6.25E-245 | Proliferating | PLK1 |
| NUSAP1 | 3.93E-248 | 1.038204658 | 0.485 | 0.02 | 1.22E-243 | Proliferating | NUSAP1 |
| FOXM1 | 5.35E-248 | 0.614064998 | 0.333 | 0.007 | 1.66E-243 | Proliferating | FOXM1 |
| MAD2L1 | 7.16E-239 | 0.731476704 | 0.515 | 0.024 | 2.23E-234 | Proliferating | MAD2L1 |
| CDCA3 | 9.04E-230 | 0.449011232 | 0.285 | 0.005 | 2.81E-225 | Proliferating | CDCA3 |
| SPC24 | 2.44E-224 | 0.354330298 | 0.248 | 0.003 | 7.59E-220 | Proliferating | SPC24 |
| CENPK | 3.10E-220 | 0.644440815 | 0.376 | 0.012 | 9.66E-216 | Proliferating | CENPK |
| DLGAP5 | 8.90E-219 | 0.400251633 | 0.206 | 0.001 | 2.77E-214 | Proliferating | DLGAP5 |
| SHCBP1 | 2.90E-213 | 0.581671742 | 0.364 | 0.011 | 9.03E-209 | Proliferating | SHCBP1 |
| ASPM | 4.46E-212 | 0.373975558 | 0.224 | 0.002 | 1.39E-207 | Proliferating | ASPM |
| PKMYT1 | 3.88E-209 | 0.415469756 | 0.248 | 0.004 | 1.21E-204 | Proliferating | PKMYT1 |
| CENPF | 1.08E-208 | 0.82898869 | 0.388 | 0.014 | 3.37E-204 | Proliferating | CENPF |
| DEPDC1 | 4.15E-208 | 0.358432714 | 0.206 | 0.002 | 1.29E-203 | Proliferating | DEPDC1 |
| CCNA2 | 4.94E-206 | 0.507011444 | 0.255 | 0.004 | 1.54E-201 | Proliferating | CCNA2 |
| NUF2 | 2.21E-199 | 0.429769423 | 0.248 | 0.004 | 6.88E-195 | Proliferating | NUF2 |
| CENPA | 4.13E-199 | 0.49380929 | 0.236 | 0.003 | 1.29E-194 | Proliferating | CENPA |
| ESCO2 | 3.82E-196 | 0.320595624 | 0.218 | 0.003 | 1.19E-191 | Proliferating | ESCO2 |
| KIF11 | 3.18E-194 | 0.413134144 | 0.261 | 0.005 | 9.88E-190 | Proliferating | KIF11 |
| CDC20 | 2.16E-191 | 0.675838354 | 0.333 | 0.011 | 6.73E-187 | Proliferating | CDC20 |
| PRC1 | 1.51E-190 | 0.617325297 | 0.352 | 0.013 | 4.69E-186 | Proliferating | PRC1 |
| CCNB2 | 2.30E-176 | 0.629477703 | 0.333 | 0.012 | 7.15E-172 | Proliferating | CCNB2 |
| TCF19 | 3.65E-175 | 0.445987421 | 0.327 | 0.012 | 1.14E-170 | Proliferating | TCF19 |
| ASF1B | 5.15E-175 | 0.466214788 | 0.303 | 0.01 | 1.60E-170 | Proliferating | ASF1B |
| MELK | 1.59E-172 | 0.425841864 | 0.279 | 0.008 | 4.94E-168 | Proliferating | MELK |
| CKS1B | 5.52E-171 | 2.037565027 | 0.842 | 0.131 | 1.72E-166 | Proliferating | CKS1B |
| TMEM106C | 1.54E-169 | 1.00120528 | 0.636 | 0.06 | 4.78E-165 | Proliferating | TMEM106C |
| DHFR | 2.77E-168 | 0.770016395 | 0.479 | 0.032 | 8.62E-164 | Proliferating | DHFR |
| NCAPG | 2.27E-167 | 0.28092504 | 0.194 | 0.003 | 7.05E-163 | Proliferating | NCAPG |
| RAD51AP1 | 6.07E-166 | 0.739229569 | 0.479 | 0.032 | 1.89E-161 | Proliferating | RAD51AP1 |
| TROAP | 7.23E-163 | 0.443703925 | 0.242 | 0.006 | 2.25E-158 | Proliferating | TROAP |
| NEK2 | 8.34E-162 | 0.28975933 | 0.176 | 0.002 | 2.60E-157 | Proliferating | NEK2 |
| CENPN | 5.41E-160 | 0.687503195 | 0.485 | 0.035 | 1.68E-155 | Proliferating | CENPN |
| UBE2T | 3.26E-159 | 0.535878864 | 0.352 | 0.017 | 1.01E-154 | Proliferating | UBE2T |
| NCAPH | 9.43E-159 | 0.278773946 | 0.212 | 0.004 | 2.94E-154 | Proliferating | NCAPH |
| UHRF1 | 1.08E-158 | 0.579050747 | 0.309 | 0.012 | 3.37E-154 | Proliferating | UHRF1 |
| STMN11 | 2.83E-158 | 2.832287111 | 0.964 | 0.218 | 8.81E-154 | Proliferating | STMN1 |
| MCM7 | 2.54E-156 | 0.940682671 | 0.515 | 0.042 | 7.89E-152 | Proliferating | MCM7 |
| BUB1 | 1.26E-150 | 0.272188106 | 0.17 | 0.002 | 3.91E-146 | Proliferating | BUB1 |
| SGOL1 | 6.18E-148 | 0.389971605 | 0.242 | 0.007 | 1.92E-143 | Proliferating | SGOL1 |
| CLSPN | 7.78E-147 | 0.447367841 | 0.279 | 0.01 | 2.42E-142 | Proliferating | CLSPN |
| KIF23 | 2.40E-143 | 0.288296532 | 0.176 | 0.003 | 7.45E-139 | Proliferating | KIF23 |
| KIF2C | 3.22E-143 | 0.291540139 | 0.17 | 0.002 | 1.00E-138 | Proliferating | KIF2C |
| CENPU | 9.56E-142 | 0.774118552 | 0.406 | 0.027 | 2.98E-137 | Proliferating | CENPU |
| CCDC34 | 6.44E-141 | 0.654636147 | 0.394 | 0.026 | 2.00E-136 | Proliferating | CCDC34 |
| CIT | 2.09E-140 | 0.279381712 | 0.158 | 0.002 | 6.52E-136 | Proliferating | CIT |
| FAM64A | 4.56E-140 | 0.265788606 | 0.139 | 0.001 | 1.42E-135 | Proliferating | FAM64A |
| GGH | 8.99E-139 | 0.974462644 | 0.624 | 0.072 | 2.80E-134 | Proliferating | GGH |
| BUB1B | 9.07E-137 | 0.252155499 | 0.152 | 0.002 | 2.82E-132 | Proliferating | BUB1B |
| CDT1 | 1.95E-136 | 0.404173806 | 0.267 | 0.01 | 6.06E-132 | Proliferating | CDT1 |
| SMC2 | 5.74E-134 | 0.764998123 | 0.473 | 0.041 | 1.79E-129 | Proliferating | SMC2 |
| FANCI | 1.63E-131 | 0.355700634 | 0.242 | 0.009 | 5.06E-127 | Proliferating | FANCI |
| HELLS | 1.28E-126 | 0.524641558 | 0.303 | 0.016 | 3.99E-122 | Proliferating | HELLS |
| OIP5 | 1.21E-122 | 0.342454697 | 0.206 | 0.006 | 3.75E-118 | Proliferating | OIP5 |
| APITD1 | 8.83E-120 | 0.389991453 | 0.279 | 0.014 | 2.75E-115 | Proliferating | APITD1 |
| HIST1H4C | 4.89E-119 | 1.75634536 | 0.661 | 0.102 | 1.52E-114 | Proliferating | HIST1H4C |
| WDR34 | 5.30E-117 | 0.533677045 | 0.333 | 0.022 | 1.65E-112 | Proliferating | WDR34 |
| PTTG11 | 3.40E-116 | 1.661850928 | 0.673 | 0.108 | 1.06E-111 | Proliferating | PTTG1 |
| ARHGAP11A | 3.47E-115 | 0.264873681 | 0.2 | 0.006 | 1.08E-110 | Proliferating | ARHGAP11A |
| FEN1 | 6.75E-114 | 0.450748129 | 0.345 | 0.024 | 2.10E-109 | Proliferating | FEN1 |
| CENPE | 2.51E-113 | 0.350344322 | 0.212 | 0.008 | 7.80E-109 | Proliferating | CENPE |
| DTYMK | 4.32E-113 | 1.044114724 | 0.612 | 0.088 | 1.34E-108 | Proliferating | DTYMK |
| SKA3 | 8.82E-113 | 0.268442566 | 0.164 | 0.004 | 2.74E-108 | Proliferating | SKA3 |
| ATAD2 | 2.42E-110 | 0.42964801 | 0.261 | 0.013 | 7.54E-106 | Proliferating | ATAD2 |
| E2F1 | 2.47E-110 | 0.364141356 | 0.2 | 0.007 | 7.69E-106 | Proliferating | E2F1 |
| CENPW | 5.32E-110 | 0.877035847 | 0.606 | 0.085 | 1.65E-105 | Proliferating | CENPW |
| POC1A | 7.16E-110 | 0.282624517 | 0.212 | 0.008 | 2.23E-105 | Proliferating | POC1A |
| RAD51 | 2.41E-106 | 0.266000011 | 0.164 | 0.004 | 7.49E-102 | Proliferating | RAD51 |
| RRM1 | 1.34E-104 | 0.668406378 | 0.412 | 0.04 | 4.17E-100 | Proliferating | RRM1 |
| CASC5 | 1.38E-104 | 0.283085708 | 0.158 | 0.004 | 4.30E-100 | Proliferating | CASC5 |
| CENPH | 1.41E-104 | 0.500719722 | 0.327 | 0.024 | 4.39E-100 | Proliferating | CENPH |
| GINS2 | 2.04E-104 | 0.450698836 | 0.261 | 0.014 | 6.36E-100 | Proliferating | GINS2 |
| H2AFZ | 4.43E-99 | 2.599316136 | 0.994 | 0.709 | 1.38E-94 | Proliferating | H2AFZ |
| KIFC1 | 4.73E-97 | 0.303264038 | 0.218 | 0.01 | 1.47E-92 | Proliferating | KIFC1 |
| GMNN | 8.79E-97 | 0.609143731 | 0.43 | 0.047 | 2.74E-92 | Proliferating | GMNN |
| MCM4 | 7.12E-96 | 0.579182358 | 0.321 | 0.026 | 2.21E-91 | Proliferating | MCM4 |
| CCNB1 | 8.54E-96 | 0.580966921 | 0.279 | 0.019 | 2.66E-91 | Proliferating | CCNB1 |
| CENPP | 6.42E-95 | 0.331539483 | 0.248 | 0.015 | 2.00E-90 | Proliferating | CENPP |
| ORC6 | 2.98E-94 | 0.366519348 | 0.248 | 0.015 | 9.27E-90 | Proliferating | ORC6 |
| SMC4 | 2.04E-91 | 0.935123757 | 0.57 | 0.089 | 6.34E-87 | Proliferating | SMC4 |
| IQGAP3 | 7.95E-91 | 0.38747283 | 0.206 | 0.01 | 2.47E-86 | Proliferating | IQGAP3 |
| DNAJC9 | 3.64E-89 | 0.60991044 | 0.436 | 0.053 | 1.13E-84 | Proliferating | DNAJC9 |
| KIF20B | 2.21E-87 | 0.397890099 | 0.273 | 0.02 | 6.87E-83 | Proliferating | KIF20B |
| TUBB | 7.07E-85 | 2.123623676 | 0.958 | 0.529 | 2.20E-80 | Proliferating | TUBB |
| SAC3D1 | 1.07E-84 | 0.484428485 | 0.345 | 0.034 | 3.33E-80 | Proliferating | SAC3D1 |
| SKA2 | 3.98E-84 | 0.827630519 | 0.552 | 0.091 | 1.24E-79 | Proliferating | SKA2 |
| HMGB3 | 6.15E-84 | 0.85547496 | 0.558 | 0.093 | 1.91E-79 | Proliferating | HMGB3 |
| SGOL2 | 9.28E-83 | 0.311742973 | 0.218 | 0.013 | 2.89E-78 | Proliferating | SGOL2 |
| RPL39L | 1.06E-82 | 0.654737764 | 0.376 | 0.042 | 3.30E-78 | Proliferating | RPL39L |
| NCAPD2 | 4.13E-81 | 0.322483473 | 0.218 | 0.013 | 1.29E-76 | Proliferating | NCAPD2 |
| TUBA1B1 | 1.33E-80 | 2.162879304 | 0.988 | 0.62 | 4.13E-76 | Proliferating | TUBA1B |
| HIRIP3 | 7.77E-80 | 0.396155493 | 0.315 | 0.03 | 2.42E-75 | Proliferating | HIRIP3 |
| TMPO | 8.58E-79 | 0.816966948 | 0.576 | 0.102 | 2.67E-74 | Proliferating | TMPO |
| HMGB1 | 1.20E-78 | 1.762276656 | 0.982 | 0.689 | 3.72E-74 | Proliferating | HMGB1 |
| DUT | 2.05E-78 | 1.717941884 | 0.8 | 0.252 | 6.37E-74 | Proliferating | DUT |
| RNASEH2A | 2.07E-78 | 0.675596186 | 0.442 | 0.062 | 6.44E-74 | Proliferating | RNASEH2A |
| RACGAP1 | 2.89E-78 | 0.521417919 | 0.297 | 0.027 | 8.99E-74 | Proliferating | RACGAP1 |
| ITGB3BP | 3.79E-77 | 0.441021649 | 0.376 | 0.044 | 1.18E-72 | Proliferating | ITGB3BP |
| FAM111A | 1.36E-76 | 0.565296438 | 0.442 | 0.062 | 4.24E-72 | Proliferating | FAM111A |
| PCNA | 1.88E-76 | 1.49218101 | 0.57 | 0.115 | 5.85E-72 | Proliferating | PCNA |
| CDKN2C | 1.64E-74 | 0.49918667 | 0.279 | 0.025 | 5.09E-70 | Proliferating | CDKN2C |
| CBX5 | 4.40E-72 | 0.57737249 | 0.461 | 0.07 | 1.37E-67 | Proliferating | CBX5 |
| NCAPD3 | 5.80E-72 | 0.374628442 | 0.285 | 0.027 | 1.80E-67 | Proliferating | NCAPD3 |
| WHSC1 | 6.39E-72 | 0.443190887 | 0.291 | 0.028 | 1.99E-67 | Proliferating | WHSC1 |
| HMGN2 | 8.93E-72 | 2.0341905 | 0.964 | 0.585 | 2.78E-67 | Proliferating | HMGN2 |
| NUCKS1 | 2.59E-71 | 1.315043449 | 0.903 | 0.35 | 8.07E-67 | Proliferating | NUCKS1 |
| CARHSP1 | 3.52E-69 | 0.956457755 | 0.661 | 0.155 | 1.10E-64 | Proliferating | CARHSP1 |
| PRIM1 | 7.07E-68 | 0.361952631 | 0.218 | 0.017 | 2.20E-63 | Proliferating | PRIM1 |
| RPA3 | 1.40E-66 | 1.154180059 | 0.782 | 0.232 | 4.34E-62 | Proliferating | RPA3 |
| PHF19 | 3.33E-66 | 0.644482456 | 0.448 | 0.073 | 1.04E-61 | Proliferating | PHF19 |
| PARP11 | 8.74E-66 | 1.037154812 | 0.655 | 0.162 | 2.72E-61 | Proliferating | PARP1 |
| STRA13 | 9.72E-64 | 1.17102044 | 0.782 | 0.246 | 3.03E-59 | Proliferating | STRA13 |
| HMGB2 | 1.46E-63 | 1.730160946 | 0.842 | 0.344 | 4.54E-59 | Proliferating | HMGB2 |
| DEK | 4.97E-63 | 1.359457889 | 0.927 | 0.438 | 1.55E-58 | Proliferating | DEK |
| ANP32E | 1.73E-62 | 0.90857542 | 0.618 | 0.15 | 5.38E-58 | Proliferating | ANP32E |
| PSIP1 | 2.87E-62 | 0.557998437 | 0.442 | 0.074 | 8.92E-58 | Proliferating | PSIP1 |
| KPNA2 | 6.40E-62 | 0.9795139 | 0.661 | 0.168 | 1.99E-57 | Proliferating | KPNA2 |
| KIF22 | 1.04E-61 | 0.539600599 | 0.376 | 0.055 | 3.25E-57 | Proliferating | KIF22 |
| TMEM97 | 3.32E-61 | 0.298265133 | 0.23 | 0.021 | 1.03E-56 | Proliferating | TMEM97 |
| FANCD2 | 1.88E-60 | 0.250796236 | 0.17 | 0.011 | 5.86E-56 | Proliferating | FANCD2 |
| H2AFV1 | 3.81E-59 | 1.367045161 | 0.927 | 0.464 | 1.19E-54 | Proliferating | H2AFV |
| PXMP2 | 2.02E-58 | 0.285052603 | 0.236 | 0.023 | 6.29E-54 | Proliferating | PXMP2 |
| C14orf80 | 4.14E-58 | 0.388822004 | 0.285 | 0.034 | 1.29E-53 | Proliferating | C14orf80 |
| MCM3 | 1.68E-57 | 0.428439354 | 0.321 | 0.043 | 5.24E-53 | Proliferating | MCM3 |
| CDC25B | 5.85E-57 | 0.34511078 | 0.321 | 0.043 | 1.82E-52 | Proliferating | CDC25B |
| MCM6 | 7.57E-57 | 0.404751986 | 0.248 | 0.027 | 2.36E-52 | Proliferating | MCM6 |
| USP1 | 6.15E-56 | 0.570120599 | 0.394 | 0.066 | 1.91E-51 | Proliferating | USP1 |
| SVIP | 2.81E-55 | 0.606546153 | 0.418 | 0.075 | 8.75E-51 | Proliferating | SVIP |
| MCM2 | 4.13E-55 | 0.319934057 | 0.188 | 0.015 | 1.29E-50 | Proliferating | MCM2 |
| RANBP1 | 1.02E-54 | 1.363705207 | 0.891 | 0.424 | 3.17E-50 | Proliferating | RANBP1 |
| SMC1A | 1.96E-54 | 0.673732475 | 0.539 | 0.124 | 6.11E-50 | Proliferating | SMC1A |
| TACC3 | 8.03E-54 | 0.408163454 | 0.321 | 0.046 | 2.50E-49 | Proliferating | TACC3 |
| CKAP2 | 9.32E-54 | 0.412176083 | 0.248 | 0.028 | 2.90E-49 | Proliferating | CKAP2 |
| ANP32B | 8.44E-53 | 1.189983791 | 0.867 | 0.394 | 2.63E-48 | Proliferating | ANP32B |
| FADS1 | 4.80E-52 | 0.568749291 | 0.436 | 0.082 | 1.49E-47 | Proliferating | FADS1 |
| SNRNP25 | 7.26E-51 | 0.912133948 | 0.612 | 0.176 | 2.26E-46 | Proliferating | SNRNP25 |
| PTMA1 | 1.46E-50 | 0.939642124 | 1 | 0.98 | 4.54E-46 | Proliferating | PTMA |
| SIVA1 | 6.01E-50 | 0.967178184 | 0.818 | 0.321 | 1.87E-45 | Proliferating | SIVA1 |
| BRI3BP | 6.95E-49 | 0.422798429 | 0.388 | 0.07 | 2.16E-44 | Proliferating | BRI3BP |
| MZT2A1 | 7.89E-49 | 0.961960535 | 0.739 | 0.267 | 2.46E-44 | Proliferating | MZT2A |
| RPA1 | 7.46E-48 | 0.549715045 | 0.406 | 0.08 | 2.32E-43 | Proliferating | RPA1 |
| LSM5 | 8.31E-48 | 1.026053316 | 0.842 | 0.374 | 2.59E-43 | Proliferating | LSM5 |
| CKAP5 | 2.51E-47 | 0.432124248 | 0.315 | 0.049 | 7.80E-43 | Proliferating | CKAP5 |
| NRM | 5.01E-47 | 0.41139036 | 0.309 | 0.048 | 1.56E-42 | Proliferating | NRM |
| NASP | 1.66E-46 | 0.753179205 | 0.612 | 0.175 | 5.16E-42 | Proliferating | NASP |
| TMEM1601 | 7.13E-46 | 0.904286724 | 0.873 | 0.37 | 2.22E-41 | Proliferating | TMEM160 |
| FANCA | 1.77E-45 | 0.292819167 | 0.194 | 0.02 | 5.49E-41 | Proliferating | FANCA |
| HAUS1 | 2.27E-45 | 0.544229004 | 0.442 | 0.098 | 7.06E-41 | Proliferating | HAUS1 |
| MCM5 | 9.76E-45 | 0.559760153 | 0.418 | 0.09 | 3.04E-40 | Proliferating | MCM5 |
| IDH22 | 2.28E-44 | 1.016526845 | 0.752 | 0.298 | 7.11E-40 | Proliferating | IDH2 |
| LIG1 | 2.92E-44 | 0.289762231 | 0.218 | 0.026 | 9.09E-40 | Proliferating | LIG1 |
| NUDT1 | 4.39E-44 | 0.769918182 | 0.624 | 0.194 | 1.37E-39 | Proliferating | NUDT1 |
| NUP210 | 3.07E-43 | 0.29222464 | 0.248 | 0.034 | 9.55E-39 | Proliferating | NUP210 |
| LSM4 | 3.37E-43 | 0.98183401 | 0.818 | 0.386 | 1.05E-38 | Proliferating | LSM4 |
| MZT2B | 4.14E-43 | 1.026911534 | 0.83 | 0.386 | 1.29E-38 | Proliferating | MZT2B |
| BCL2L12 | 1.08E-42 | 0.379985424 | 0.352 | 0.066 | 3.36E-38 | Proliferating | BCL2L12 |
| RAN | 1.23E-42 | 1.053206842 | 0.933 | 0.59 | 3.84E-38 | Proliferating | RAN |
| DCK | 2.98E-42 | 0.555272227 | 0.515 | 0.132 | 9.26E-38 | Proliferating | DCK |
| DNMT11 | 1.04E-41 | 0.730346728 | 0.606 | 0.185 | 3.25E-37 | Proliferating | DNMT1 |
| LMNB2 | 1.09E-41 | 0.333018507 | 0.285 | 0.046 | 3.40E-37 | Proliferating | LMNB2 |
| CDK4 | 2.16E-41 | 0.632327356 | 0.545 | 0.152 | 6.73E-37 | Proliferating | CDK4 |
| UNG | 4.50E-41 | 0.326891173 | 0.164 | 0.016 | 1.40E-36 | Proliferating | UNG |
| SAE1 | 1.24E-40 | 0.440727911 | 0.406 | 0.089 | 3.87E-36 | Proliferating | SAE1 |
| UBE2S | 4.39E-40 | 0.907668475 | 0.588 | 0.189 | 1.37E-35 | Proliferating | UBE2S |
| ASRGL1 | 5.65E-40 | 0.361071118 | 0.297 | 0.052 | 1.76E-35 | Proliferating | ASRGL1 |
| SSRP1 | 8.38E-40 | 0.51043517 | 0.485 | 0.126 | 2.61E-35 | Proliferating | SSRP1 |
| RFC4 | 1.24E-39 | 0.272779754 | 0.2 | 0.024 | 3.87E-35 | Proliferating | RFC4 |
| NR2C2AP | 1.75E-39 | 0.288688673 | 0.206 | 0.026 | 5.45E-35 | Proliferating | NR2C2AP |
| POLR3K | 2.26E-39 | 0.512384998 | 0.388 | 0.085 | 7.04E-35 | Proliferating | POLR3K |
| EXOSC8 | 2.50E-39 | 0.437778575 | 0.358 | 0.073 | 7.79E-35 | Proliferating | EXOSC8 |
| LDHB2 | 2.97E-39 | 1.068227872 | 0.8 | 0.381 | 9.24E-35 | Proliferating | LDHB |
| C19orf48 | 3.85E-39 | 0.37181279 | 0.303 | 0.054 | 1.20E-34 | Proliferating | C19orf48 |
| ICMT | 5.59E-39 | 0.390769412 | 0.309 | 0.057 | 1.74E-34 | Proliferating | ICMT |
| TUBA1C1 | 7.92E-38 | 1.182854746 | 0.855 | 0.395 | 2.46E-33 | Proliferating | TUBA1C |
| HINT12 | 1.24E-37 | 0.901225654 | 0.97 | 0.724 | 3.87E-33 | Proliferating | HINT1 |
| HNRNPAB | 1.34E-37 | 0.899514357 | 0.691 | 0.277 | 4.18E-33 | Proliferating | HNRNPAB |
| MZT1 | 2.02E-37 | 0.524931653 | 0.467 | 0.122 | 6.28E-33 | Proliferating | MZT1 |
| ZDHHC121 | 2.05E-37 | 0.655017477 | 0.63 | 0.214 | 6.38E-33 | Proliferating | ZDHHC12 |
| LMNB11 | 2.51E-37 | 0.350482691 | 0.333 | 0.065 | 7.80E-33 | Proliferating | LMNB1 |
| RNASEH2B | 1.30E-36 | 0.587663381 | 0.576 | 0.178 | 4.04E-32 | Proliferating | RNASEH2B |
| ACOT71 | 5.40E-36 | 0.360614817 | 0.37 | 0.083 | 1.68E-31 | Proliferating | ACOT7 |
| EMC9 | 1.17E-35 | 0.335258286 | 0.261 | 0.044 | 3.63E-31 | Proliferating | EMC9 |
| PMM1 | 1.34E-35 | 0.311023284 | 0.303 | 0.058 | 4.16E-31 | Proliferating | PMM1 |
| UQCC21 | 1.96E-35 | 0.676724477 | 0.691 | 0.258 | 6.09E-31 | Proliferating | UQCC2 |
| TUBG1 | 2.14E-35 | 0.314452646 | 0.255 | 0.043 | 6.67E-31 | Proliferating | TUBG1 |
| BRCA2 | 2.80E-35 | 0.25478387 | 0.194 | 0.026 | 8.73E-31 | Proliferating | BRCA2 |
| ANKRD39 | 5.51E-35 | 0.363553785 | 0.327 | 0.069 | 1.72E-30 | Proliferating | ANKRD39 |
| RBBP7 | 6.16E-35 | 0.519789535 | 0.485 | 0.138 | 1.92E-30 | Proliferating | RBBP7 |
| HACD3 | 6.73E-35 | 0.422573638 | 0.358 | 0.08 | 2.09E-30 | Proliferating | HACD3 |
| COQ2 | 6.97E-35 | 0.544212912 | 0.485 | 0.14 | 2.17E-30 | Proliferating | COQ2 |
| SNRPD1 | 7.65E-35 | 0.796809659 | 0.806 | 0.359 | 2.38E-30 | Proliferating | SNRPD1 |
| POLD1 | 1.05E-34 | 0.299329159 | 0.224 | 0.035 | 3.26E-30 | Proliferating | POLD1 |
| DCTPP1 | 1.57E-34 | 0.547111808 | 0.491 | 0.144 | 4.88E-30 | Proliferating | DCTPP1 |
| TPGS2 | 1.77E-34 | 0.413317521 | 0.406 | 0.1 | 5.52E-30 | Proliferating | TPGS2 |
| H2AFX | 2.03E-34 | 0.583420631 | 0.461 | 0.13 | 6.31E-30 | Proliferating | H2AFX |
| LBR | 2.03E-34 | 0.388799981 | 0.442 | 0.115 | 6.33E-30 | Proliferating | LBR |
| C1QB2 | 2.09E-34 | 1.020539348 | 0.976 | 0.505 | 6.51E-30 | Proliferating | C1QB |
| PPIH | 2.28E-34 | 0.398204452 | 0.418 | 0.104 | 7.10E-30 | Proliferating | PPIH |
| TMEM14C2 | 2.61E-34 | 0.87349507 | 0.867 | 0.433 | 8.11E-30 | Proliferating | TMEM14C |
| NDC1 | 2.99E-34 | 0.257899557 | 0.182 | 0.023 | 9.29E-30 | Proliferating | NDC1 |
| POLE3 | 3.39E-34 | 0.486878229 | 0.455 | 0.122 | 1.06E-29 | Proliferating | POLE3 |
| PLAU2 | 9.01E-34 | 0.78708741 | 0.782 | 0.314 | 2.80E-29 | Proliferating | PLAU |
| TIMM10 | 9.69E-34 | 0.610721147 | 0.63 | 0.22 | 3.02E-29 | Proliferating | TIMM10 |
| ACTL6A | 1.15E-33 | 0.38665165 | 0.285 | 0.055 | 3.59E-29 | Proliferating | ACTL6A |
| PGP | 1.53E-33 | 0.458717141 | 0.448 | 0.123 | 4.77E-29 | Proliferating | PGP |
| POLD2 | 2.38E-33 | 0.370525619 | 0.388 | 0.093 | 7.42E-29 | Proliferating | POLD2 |
| PPIA1 | 2.99E-33 | 0.804204702 | 0.988 | 0.859 | 9.31E-29 | Proliferating | PPIA |
| GMPS | 1.55E-32 | 0.364865515 | 0.321 | 0.069 | 4.82E-28 | Proliferating | GMPS |
| RB12 | 1.64E-32 | 0.664048386 | 0.727 | 0.282 | 5.10E-28 | Proliferating | RB1 |
| VRK1 | 2.60E-32 | 0.307087532 | 0.224 | 0.037 | 8.08E-28 | Proliferating | VRK1 |
| CHAF1A | 4.88E-32 | 0.302655764 | 0.248 | 0.045 | 1.52E-27 | Proliferating | CHAF1A |
| MMD | 6.03E-32 | 0.488463613 | 0.455 | 0.129 | 1.88E-27 | Proliferating | MMD |
| ANAPC11 | 7.76E-32 | 0.834788875 | 0.861 | 0.52 | 2.41E-27 | Proliferating | ANAPC11 |
| PRPS2 | 1.05E-31 | 0.401654724 | 0.394 | 0.1 | 3.27E-27 | Proliferating | PRPS2 |
| SLBP | 1.27E-31 | 0.495568437 | 0.533 | 0.171 | 3.97E-27 | Proliferating | SLBP |
| EBP | 1.79E-31 | 0.529104334 | 0.461 | 0.135 | 5.57E-27 | Proliferating | EBP |
| TEX30 | 2.65E-31 | 0.359902697 | 0.297 | 0.062 | 8.24E-27 | Proliferating | TEX30 |
| YEATS4 | 3.41E-31 | 0.303338522 | 0.303 | 0.065 | 1.06E-26 | Proliferating | YEATS4 |
| SAP30 | 5.83E-31 | 0.405956436 | 0.321 | 0.072 | 1.81E-26 | Proliferating | SAP30 |
| CSE1L | 1.21E-30 | 0.295672466 | 0.279 | 0.056 | 3.76E-26 | Proliferating | CSE1L |
| LSM2 | 2.16E-30 | 0.599627091 | 0.606 | 0.223 | 6.71E-26 | Proliferating | LSM2 |
| HN11 | 2.38E-30 | 0.892947694 | 0.891 | 0.493 | 7.40E-26 | Proliferating | HN1 |
| EMP21 | 2.42E-30 | 0.466781188 | 0.333 | 0.078 | 7.54E-26 | Proliferating | EMP2 |
| GALM1 | 2.82E-30 | 0.583559475 | 0.509 | 0.17 | 8.78E-26 | Proliferating | GALM |
| MRPL37 | 3.24E-30 | 0.419350674 | 0.43 | 0.122 | 1.01E-25 | Proliferating | MRPL37 |
| VAMP82 | 3.79E-30 | 0.748682667 | 0.988 | 0.723 | 1.18E-25 | Proliferating | VAMP8 |
| SLC25A52 | 4.38E-30 | 0.964353301 | 0.915 | 0.654 | 1.36E-25 | Proliferating | SLC25A5 |
| COMMD4 | 6.12E-30 | 0.531552512 | 0.564 | 0.196 | 1.90E-25 | Proliferating | COMMD4 |
| GLRX5 | 6.33E-30 | 0.41221554 | 0.418 | 0.117 | 1.97E-25 | Proliferating | GLRX5 |
| NENF2 | 6.64E-30 | 0.635754776 | 0.806 | 0.358 | 2.07E-25 | Proliferating | NENF |
| MRPL12 | 9.35E-30 | 0.589327144 | 0.648 | 0.248 | 2.91E-25 | Proliferating | MRPL12 |
| C1QC2 | 1.35E-29 | 0.80694973 | 0.964 | 0.477 | 4.20E-25 | Proliferating | C1QC |
| MRPL17 | 3.32E-29 | 0.41006573 | 0.442 | 0.13 | 1.03E-24 | Proliferating | MRPL17 |
| CKS21 | 4.08E-29 | 0.806021294 | 0.776 | 0.374 | 1.27E-24 | Proliferating | CKS2 |
| CBX1 | 6.57E-29 | 0.3784834 | 0.345 | 0.085 | 2.05E-24 | Proliferating | CBX1 |
| CCDC14 | 6.99E-29 | 0.278389973 | 0.248 | 0.048 | 2.18E-24 | Proliferating | CCDC14 |
| SNRPE | 7.62E-29 | 0.759166896 | 0.836 | 0.444 | 2.37E-24 | Proliferating | SNRPE |
| PAFAH1B3 | 9.59E-29 | 0.383143213 | 0.388 | 0.105 | 2.99E-24 | Proliferating | PAFAH1B3 |
| NME4 | 1.04E-28 | 0.523789186 | 0.527 | 0.175 | 3.23E-24 | Proliferating | NME4 |
| ALYREF | 1.16E-28 | 0.419480779 | 0.333 | 0.081 | 3.62E-24 | Proliferating | ALYREF |
| LRRC45 | 1.33E-28 | 0.253652347 | 0.164 | 0.023 | 4.15E-24 | Proliferating | LRRC45 |
| C1QA2 | 1.69E-28 | 0.851932235 | 0.994 | 0.533 | 5.25E-24 | Proliferating | C1QA |
| PRPS1 | 1.79E-28 | 0.349368836 | 0.327 | 0.078 | 5.56E-24 | Proliferating | PRPS1 |
| S100B2 | 4.53E-28 | 0.707045093 | 0.424 | 0.126 | 1.41E-23 | Proliferating | S100B |
| COX5A1 | 7.58E-28 | 0.786850295 | 0.891 | 0.585 | 2.36E-23 | Proliferating | COX5A |
| SMC3 | 3.54E-27 | 0.396321561 | 0.406 | 0.117 | 1.10E-22 | Proliferating | SMC3 |
| LSM3 | 8.29E-27 | 0.70596921 | 0.812 | 0.433 | 2.58E-22 | Proliferating | LSM3 |
| NAP1L4 | 1.37E-26 | 0.330577468 | 0.352 | 0.093 | 4.26E-22 | Proliferating | NAP1L4 |
| AURKA | 1.43E-26 | 0.309829969 | 0.164 | 0.024 | 4.45E-22 | Proliferating | AURKA |
| YWHAH2 | 1.51E-26 | 0.727462683 | 0.915 | 0.522 | 4.70E-22 | Proliferating | YWHAH |
| NFATC2IP | 1.66E-26 | 0.284682886 | 0.352 | 0.091 | 5.18E-22 | Proliferating | NFATC2IP |
| HSPB111 | 1.73E-26 | 0.568752713 | 0.588 | 0.229 | 5.38E-22 | Proliferating | HSPB11 |
| RAD21 | 2.17E-26 | 0.52839981 | 0.533 | 0.193 | 6.74E-22 | Proliferating | RAD21 |
| EPB41L21 | 2.45E-26 | 0.421311912 | 0.406 | 0.121 | 7.62E-22 | Proliferating | EPB41L2 |
| KPNB1 | 2.47E-26 | 0.576833696 | 0.685 | 0.29 | 7.69E-22 | Proliferating | KPNB1 |
| CCDC85B2 | 3.00E-26 | 0.605199389 | 0.715 | 0.311 | 9.33E-22 | Proliferating | CCDC85B |
| HMGN11 | 3.16E-26 | 0.766958092 | 0.897 | 0.569 | 9.84E-22 | Proliferating | HMGN1 |
| NUCB2 | 3.62E-26 | 0.356196738 | 0.412 | 0.121 | 1.13E-21 | Proliferating | NUCB2 |
| YWHAQ1 | 4.39E-26 | 0.620938446 | 0.77 | 0.371 | 1.37E-21 | Proliferating | YWHAQ |
| C4orf27 | 4.65E-26 | 0.271152159 | 0.285 | 0.065 | 1.45E-21 | Proliferating | C4orf27 |
| PPM1G1 | 4.94E-26 | 0.540211208 | 0.539 | 0.201 | 1.54E-21 | Proliferating | PPM1G |
| HYI | 5.11E-26 | 0.299112646 | 0.255 | 0.055 | 1.59E-21 | Proliferating | HYI |
| MMP122 | 5.84E-26 | 0.550464816 | 0.648 | 0.253 | 1.82E-21 | Proliferating | MMP12 |
| VPS29 | 6.24E-26 | 0.743120555 | 0.867 | 0.489 | 1.94E-21 | Proliferating | VPS29 |
| DCAF12 | 6.66E-26 | 0.2745284 | 0.309 | 0.075 | 2.07E-21 | Proliferating | DCAF12 |
| TRIM28 | 6.90E-26 | 0.438434454 | 0.473 | 0.153 | 2.15E-21 | Proliferating | TRIM28 |
| PTMS2 | 7.20E-26 | 0.69454262 | 0.83 | 0.397 | 2.24E-21 | Proliferating | PTMS |
| STAB12 | 7.36E-26 | 0.666087609 | 0.685 | 0.296 | 2.29E-21 | Proliferating | STAB1 |
| TMEM109 | 7.38E-26 | 0.553270658 | 0.509 | 0.184 | 2.29E-21 | Proliferating | TMEM109 |
| RHNO1 | 7.81E-26 | 0.268324397 | 0.285 | 0.067 | 2.43E-21 | Proliferating | RHNO1 |
| PMVK1 | 9.44E-26 | 0.467094791 | 0.576 | 0.218 | 2.94E-21 | Proliferating | PMVK |
| HAT1 | 9.52E-26 | 0.368197904 | 0.406 | 0.121 | 2.96E-21 | Proliferating | HAT1 |
| HINT2 | 1.01E-25 | 0.468934313 | 0.533 | 0.193 | 3.13E-21 | Proliferating | HINT2 |
| ERP291 | 1.08E-25 | 0.656663721 | 0.848 | 0.45 | 3.37E-21 | Proliferating | ERP29 |
| POP7 | 1.28E-25 | 0.31327893 | 0.358 | 0.099 | 3.98E-21 | Proliferating | POP7 |
| MPC22 | 1.53E-25 | 0.634540919 | 0.673 | 0.293 | 4.76E-21 | Proliferating | MPC2 |
| FCGBP | 1.56E-25 | 0.447782546 | 0.176 | 0.029 | 4.85E-21 | Proliferating | FCGBP |
| HTRA11 | 2.23E-25 | 0.56584749 | 0.376 | 0.11 | 6.94E-21 | Proliferating | HTRA1 |
| DDX39A | 2.54E-25 | 0.53158132 | 0.552 | 0.207 | 7.91E-21 | Proliferating | DDX39A |
| RPA2 | 3.17E-25 | 0.363687154 | 0.37 | 0.105 | 9.87E-21 | Proliferating | RPA2 |
| MRPL51 | 4.03E-25 | 0.60783965 | 0.824 | 0.419 | 1.25E-20 | Proliferating | MRPL51 |
| AP1M1 | 4.42E-25 | 0.2708785 | 0.345 | 0.093 | 1.38E-20 | Proliferating | AP1M1 |
| PA2G4 | 4.46E-25 | 0.705760623 | 0.661 | 0.297 | 1.39E-20 | Proliferating | PA2G4 |
| COA1 | 5.79E-25 | 0.333788414 | 0.43 | 0.134 | 1.80E-20 | Proliferating | COA1 |
| RPP25 | 6.39E-25 | 0.260344951 | 0.248 | 0.054 | 1.99E-20 | Proliferating | RPP25 |
| HADH | 7.43E-25 | 0.323459819 | 0.309 | 0.08 | 2.31E-20 | Proliferating | HADH |
| DPM2 | 7.64E-25 | 0.316819479 | 0.43 | 0.132 | 2.38E-20 | Proliferating | DPM2 |
| HMGN31 | 9.51E-25 | 0.603784515 | 0.8 | 0.404 | 2.96E-20 | Proliferating | HMGN3 |
| MRPS16 | 1.18E-24 | 0.490630952 | 0.612 | 0.245 | 3.66E-20 | Proliferating | MRPS16 |
| BOLA3 | 1.68E-24 | 0.418464943 | 0.509 | 0.177 | 5.22E-20 | Proliferating | BOLA3 |
| UQCC3 | 1.72E-24 | 0.380496335 | 0.418 | 0.13 | 5.36E-20 | Proliferating | UQCC3 |
| TPRKB | 1.77E-24 | 0.469624169 | 0.539 | 0.204 | 5.51E-20 | Proliferating | TPRKB |
| ALOX5AP1 | 2.09E-24 | 0.942793753 | 0.879 | 0.556 | 6.51E-20 | Proliferating | ALOX5AP |
| SUPT16H | 2.17E-24 | 0.278894467 | 0.352 | 0.097 | 6.77E-20 | Proliferating | SUPT16H |
| TMEM14B | 2.33E-24 | 0.606235512 | 0.758 | 0.382 | 7.25E-20 | Proliferating | TMEM14B |
| XPO1 | 2.77E-24 | 0.389331582 | 0.448 | 0.147 | 8.61E-20 | Proliferating | XPO1 |
| ZCRB1 | 3.01E-24 | 0.484438579 | 0.63 | 0.254 | 9.37E-20 | Proliferating | ZCRB1 |
| ETV51 | 3.06E-24 | 0.490705308 | 0.467 | 0.157 | 9.54E-20 | Proliferating | ETV5 |
| BANF11 | 3.63E-24 | 0.683169915 | 0.788 | 0.41 | 1.13E-19 | Proliferating | BANF1 |
| PIN1 | 4.42E-24 | 0.507452753 | 0.636 | 0.271 | 1.37E-19 | Proliferating | PIN1 |
| COPRS | 4.83E-24 | 0.315342515 | 0.418 | 0.129 | 1.50E-19 | Proliferating | COPRS |
| NUDT21 | 5.84E-24 | 0.404327034 | 0.515 | 0.184 | 1.82E-19 | Proliferating | NUDT21 |
| ILF21 | 7.45E-24 | 0.427587727 | 0.624 | 0.253 | 2.32E-19 | Proliferating | ILF2 |
| NAA38 | 1.06E-23 | 0.546891613 | 0.782 | 0.364 | 3.30E-19 | Proliferating | NAA38 |
| C9orf142 | 1.10E-23 | 0.524730011 | 0.582 | 0.234 | 3.43E-19 | Proliferating | C9orf142 |
| RPSA2 | 1.12E-23 | 0.694473594 | 0.982 | 0.827 | 3.47E-19 | Proliferating | RPSA |
| C14orf1 | 1.78E-23 | 0.268505958 | 0.267 | 0.064 | 5.53E-19 | Proliferating | C14orf1 |
| RNASEH2C | 1.92E-23 | 0.53763381 | 0.733 | 0.351 | 5.99E-19 | Proliferating | RNASEH2C |
| NOP56 | 1.94E-23 | 0.436549098 | 0.521 | 0.193 | 6.04E-19 | Proliferating | NOP56 |
| CLN6 | 3.10E-23 | 0.298037983 | 0.364 | 0.107 | 9.65E-19 | Proliferating | CLN6 |
| FAF1 | 3.34E-23 | 0.261862935 | 0.242 | 0.055 | 1.04E-18 | Proliferating | FAF1 |
| ASH2L | 3.73E-23 | 0.261743194 | 0.218 | 0.046 | 1.16E-18 | Proliferating | ASH2L |
| NUDCD2 | 4.54E-23 | 0.363634162 | 0.473 | 0.165 | 1.41E-18 | Proliferating | NUDCD2 |
| CKLF1 | 4.61E-23 | 0.783683183 | 0.885 | 0.545 | 1.43E-18 | Proliferating | CKLF |
| NFIC | 4.64E-23 | 0.427403278 | 0.448 | 0.152 | 1.44E-18 | Proliferating | NFIC |
| SEPT11 | 5.02E-23 | 0.331697373 | 0.382 | 0.117 | 1.56E-18 | Proliferating | SEPT11 |
| MIS18BP1 | 5.73E-23 | 0.262587792 | 0.297 | 0.077 | 1.78E-18 | Proliferating | MIS18BP1 |
| PSMC3 | 8.88E-23 | 0.601858984 | 0.63 | 0.28 | 2.76E-18 | Proliferating | PSMC3 |
| SEC22C | 1.18E-22 | 0.252864192 | 0.333 | 0.093 | 3.66E-18 | Proliferating | SEC22C |
| CHCHD2 | 1.20E-22 | 0.628541104 | 0.97 | 0.844 | 3.73E-18 | Proliferating | CHCHD2 |
| BUB3 | 1.22E-22 | 0.483639999 | 0.436 | 0.15 | 3.80E-18 | Proliferating | BUB3 |
| TXNL4A | 1.52E-22 | 0.410342087 | 0.564 | 0.221 | 4.72E-18 | Proliferating | TXNL4A |
| GPN3 | 1.55E-22 | 0.394751065 | 0.376 | 0.116 | 4.81E-18 | Proliferating | GPN3 |
| SNRPF | 2.17E-22 | 0.557492786 | 0.776 | 0.392 | 6.74E-18 | Proliferating | SNRPF |
| MRPS341 | 2.39E-22 | 0.506996913 | 0.588 | 0.247 | 7.45E-18 | Proliferating | MRPS34 |
| GGCT | 3.01E-22 | 0.508696034 | 0.503 | 0.199 | 9.36E-18 | Proliferating | GGCT |
| SNRPA1 | 4.38E-22 | 0.434189384 | 0.412 | 0.139 | 1.36E-17 | Proliferating | SNRPA1 |
| NDUFS81 | 7.68E-22 | 0.561809224 | 0.824 | 0.436 | 2.39E-17 | Proliferating | NDUFS8 |
| PCBD11 | 9.46E-22 | 0.53158243 | 0.661 | 0.308 | 2.94E-17 | Proliferating | PCBD1 |
| MRPL27 | 1.07E-21 | 0.477776089 | 0.606 | 0.248 | 3.33E-17 | Proliferating | MRPL27 |
| MAGOHB | 1.08E-21 | 0.324304846 | 0.315 | 0.09 | 3.35E-17 | Proliferating | MAGOHB |
| COPS3 | 1.10E-21 | 0.413317804 | 0.43 | 0.148 | 3.42E-17 | Proliferating | COPS3 |
| H1FX | 1.15E-21 | 0.39550951 | 0.521 | 0.195 | 3.57E-17 | Proliferating | H1FX |
| RFC2 | 1.21E-21 | 0.269165344 | 0.279 | 0.073 | 3.75E-17 | Proliferating | RFC2 |
| MRPS26 | 1.33E-21 | 0.268657701 | 0.364 | 0.111 | 4.15E-17 | Proliferating | MRPS26 |
| PAICS | 1.38E-21 | 0.306442666 | 0.309 | 0.086 | 4.28E-17 | Proliferating | PAICS |
| ATL3 | 1.49E-21 | 0.316957058 | 0.406 | 0.133 | 4.65E-17 | Proliferating | ATL3 |
| EAF2 | 1.64E-21 | 0.346464302 | 0.291 | 0.078 | 5.11E-17 | Proliferating | EAF2 |
| AP2S11 | 2.08E-21 | 0.614789522 | 0.976 | 0.781 | 6.48E-17 | Proliferating | AP2S1 |
| SNRPD3 | 2.43E-21 | 0.524473811 | 0.709 | 0.344 | 7.57E-17 | Proliferating | SNRPD3 |
| COX8A | 2.46E-21 | 0.599122808 | 0.958 | 0.797 | 7.66E-17 | Proliferating | COX8A |
| FUCA21 | 3.26E-21 | 0.340283363 | 0.57 | 0.223 | 1.01E-16 | Proliferating | FUCA2 |
| SUZ12 | 3.46E-21 | 0.356119323 | 0.315 | 0.091 | 1.08E-16 | Proliferating | SUZ12 |
| SET1 | 3.53E-21 | 0.667507259 | 0.83 | 0.462 | 1.10E-16 | Proliferating | SET |
| TSPAN15 | 3.81E-21 | 0.388759569 | 0.333 | 0.101 | 1.19E-16 | Proliferating | TSPAN15 |
| CMC2 | 3.82E-21 | 0.42094336 | 0.497 | 0.188 | 1.19E-16 | Proliferating | CMC2 |
| RBBP8 | 4.26E-21 | 0.274057776 | 0.303 | 0.084 | 1.33E-16 | Proliferating | RBBP8 |
| VDAC3 | 4.69E-21 | 0.482732944 | 0.521 | 0.207 | 1.46E-16 | Proliferating | VDAC3 |
| ANAPC5 | 4.94E-21 | 0.326704456 | 0.448 | 0.155 | 1.54E-16 | Proliferating | ANAPC5 |
| AKR7A2 | 5.25E-21 | 0.505284537 | 0.479 | 0.183 | 1.63E-16 | Proliferating | AKR7A2 |
| IGFBP41 | 5.53E-21 | 0.444145584 | 0.352 | 0.11 | 1.72E-16 | Proliferating | IGFBP4 |
| CALM22 | 6.07E-21 | 0.682539246 | 0.97 | 0.76 | 1.89E-16 | Proliferating | CALM2 |
| SUMO3 | 6.12E-21 | 0.618668469 | 0.812 | 0.452 | 1.90E-16 | Proliferating | SUMO3 |
| MAZ | 6.30E-21 | 0.362150899 | 0.382 | 0.126 | 1.96E-16 | Proliferating | MAZ |
| TMED91 | 6.71E-21 | 0.599096918 | 0.794 | 0.418 | 2.09E-16 | Proliferating | TMED9 |
| C1orf542 | 1.12E-20 | 0.575486616 | 0.655 | 0.294 | 3.47E-16 | Proliferating | C1orf54 |
| AHCY | 1.13E-20 | 0.387852521 | 0.461 | 0.166 | 3.53E-16 | Proliferating | AHCY |
| TMSB4X3 | 1.33E-20 | 0.453318977 | 1 | 1 | 4.13E-16 | Proliferating | TMSB4X |
| RGS14 | 1.39E-20 | 0.703143056 | 0.952 | 0.605 | 4.31E-16 | Proliferating | RGS1 |
| POP5 | 1.54E-20 | 0.278319307 | 0.333 | 0.101 | 4.79E-16 | Proliferating | POP5 |
| NRGN | 1.64E-20 | 0.253694504 | 0.164 | 0.03 | 5.09E-16 | Proliferating | NRGN |
| TP53I13 | 1.66E-20 | 0.325773884 | 0.406 | 0.138 | 5.16E-16 | Proliferating | TP53I13 |
| TECR1 | 1.90E-20 | 0.432401305 | 0.57 | 0.238 | 5.93E-16 | Proliferating | TECR |
| NUBP2 | 2.04E-20 | 0.4148911 | 0.424 | 0.152 | 6.34E-16 | Proliferating | NUBP2 |
| MLEC1 | 2.27E-20 | 0.489789148 | 0.642 | 0.288 | 7.07E-16 | Proliferating | MLEC |
| PSMB51 | 2.40E-20 | 0.516213088 | 0.564 | 0.24 | 7.47E-16 | Proliferating | PSMB5 |
| TFDP1 | 2.61E-20 | 0.414129827 | 0.533 | 0.213 | 8.11E-16 | Proliferating | TFDP1 |
| MRPL11 | 2.67E-20 | 0.488878015 | 0.509 | 0.207 | 8.31E-16 | Proliferating | MRPL11 |
| FDPS | 3.90E-20 | 0.3262758 | 0.467 | 0.171 | 1.21E-15 | Proliferating | FDPS |
| GPAA1 | 3.90E-20 | 0.403711242 | 0.515 | 0.203 | 1.21E-15 | Proliferating | GPAA1 |
| EIF4EBP1 | 4.05E-20 | 0.511641126 | 0.758 | 0.387 | 1.26E-15 | Proliferating | EIF4EBP1 |
| MASTL | 4.52E-20 | 0.250844938 | 0.188 | 0.039 | 1.41E-15 | Proliferating | MASTL |
| ILVBL | 6.40E-20 | 0.296023592 | 0.273 | 0.074 | 1.99E-15 | Proliferating | ILVBL |
| BAZ1B | 7.59E-20 | 0.297008702 | 0.291 | 0.083 | 2.36E-15 | Proliferating | BAZ1B |
| RRP7A | 9.28E-20 | 0.370021142 | 0.479 | 0.183 | 2.89E-15 | Proliferating | RRP7A |
| SNX4 | 1.04E-19 | 0.250312603 | 0.327 | 0.099 | 3.22E-15 | Proliferating | SNX4 |
| DDX461 | 1.06E-19 | 0.374430122 | 0.612 | 0.259 | 3.29E-15 | Proliferating | DDX46 |
| CTSZ2 | 1.08E-19 | 0.645320594 | 0.97 | 0.685 | 3.35E-15 | Proliferating | CTSZ |
| HDGF | 1.36E-19 | 0.377976775 | 0.515 | 0.2 | 4.24E-15 | Proliferating | HDGF |
| CCDC167 | 1.40E-19 | 0.337379464 | 0.442 | 0.16 | 4.37E-15 | Proliferating | CCDC167 |
| TP53 | 1.54E-19 | 0.33303535 | 0.285 | 0.082 | 4.80E-15 | Proliferating | TP53 |
| TUBA1A1 | 1.88E-19 | 0.522501324 | 0.697 | 0.336 | 5.85E-15 | Proliferating | TUBA1A |
| SHMT2 | 2.02E-19 | 0.303450341 | 0.327 | 0.101 | 6.28E-15 | Proliferating | SHMT2 |
| VMA21 | 2.28E-19 | 0.439893937 | 0.703 | 0.344 | 7.09E-15 | Proliferating | VMA21 |
| COA3 | 2.31E-19 | 0.365216095 | 0.576 | 0.24 | 7.19E-15 | Proliferating | COA3 |
| MINOS1 | 2.57E-19 | 0.678323488 | 0.909 | 0.613 | 8.00E-15 | Proliferating | MINOS1 |
| GTF2A2 | 2.89E-19 | 0.525501089 | 0.691 | 0.335 | 8.99E-15 | Proliferating | GTF2A2 |
| NCL1 | 3.28E-19 | 0.587888016 | 0.812 | 0.447 | 1.02E-14 | Proliferating | NCL |
| REXO2 | 3.89E-19 | 0.311385084 | 0.424 | 0.152 | 1.21E-14 | Proliferating | REXO2 |
| PNN | 3.96E-19 | 0.328865631 | 0.442 | 0.162 | 1.23E-14 | Proliferating | PNN |
| ARL6IP11 | 4.04E-19 | 0.907444093 | 0.836 | 0.546 | 1.26E-14 | Proliferating | ARL6IP1 |
| SPATS2L | 4.09E-19 | 0.45204759 | 0.527 | 0.215 | 1.27E-14 | Proliferating | SPATS2L |
| SNRNP40 | 4.15E-19 | 0.32015648 | 0.327 | 0.103 | 1.29E-14 | Proliferating | SNRNP40 |
| GCSH | 4.52E-19 | 0.312308272 | 0.297 | 0.088 | 1.41E-14 | Proliferating | GCSH |
| ITGB71 | 4.70E-19 | 0.385121771 | 0.352 | 0.116 | 1.46E-14 | Proliferating | ITGB7 |
| ARL6IP4 | 5.04E-19 | 0.531675285 | 0.903 | 0.582 | 1.57E-14 | Proliferating | ARL6IP4 |
| HNRNPR | 5.26E-19 | 0.429518351 | 0.564 | 0.244 | 1.64E-14 | Proliferating | HNRNPR |
| GLOD4 | 5.65E-19 | 0.335993448 | 0.388 | 0.134 | 1.76E-14 | Proliferating | GLOD4 |
| FKBP3 | 7.03E-19 | 0.38110228 | 0.527 | 0.214 | 2.19E-14 | Proliferating | FKBP3 |
| IPO5 | 7.70E-19 | 0.279527695 | 0.297 | 0.087 | 2.40E-14 | Proliferating | IPO5 |
| QDPR | 8.97E-19 | 0.312492629 | 0.382 | 0.13 | 2.79E-14 | Proliferating | QDPR |
| RAP2A | 9.62E-19 | 0.314300482 | 0.37 | 0.125 | 2.99E-14 | Proliferating | RAP2A |
| NONO | 1.07E-18 | 0.44351968 | 0.63 | 0.29 | 3.32E-14 | Proliferating | NONO |
| SNRPB1 | 1.09E-18 | 0.580576989 | 0.818 | 0.512 | 3.39E-14 | Proliferating | SNRPB |
| CLPP | 1.12E-18 | 0.354069277 | 0.467 | 0.179 | 3.49E-14 | Proliferating | CLPP |
| ERH | 1.15E-18 | 0.577333885 | 0.806 | 0.498 | 3.58E-14 | Proliferating | ERH |
| ETFB | 1.24E-18 | 0.50345801 | 0.685 | 0.349 | 3.87E-14 | Proliferating | ETFB |
| CYBRD1 | 1.25E-18 | 0.292262552 | 0.285 | 0.082 | 3.88E-14 | Proliferating | CYBRD1 |
| PSMD14 | 1.37E-18 | 0.439639119 | 0.533 | 0.23 | 4.27E-14 | Proliferating | PSMD14 |
| HP1BP3 | 1.57E-18 | 0.46016547 | 0.491 | 0.205 | 4.90E-14 | Proliferating | HP1BP3 |
| CMSS1 | 1.58E-18 | 0.267754933 | 0.242 | 0.065 | 4.93E-14 | Proliferating | CMSS1 |
| RCC2 | 1.60E-18 | 0.35207586 | 0.388 | 0.137 | 4.99E-14 | Proliferating | RCC2 |
| PRDX4 | 1.82E-18 | 0.498357315 | 0.612 | 0.285 | 5.66E-14 | Proliferating | PRDX4 |
| TMED31 | 1.83E-18 | 0.35446264 | 0.552 | 0.236 | 5.70E-14 | Proliferating | TMED3 |
| MEA1 | 1.93E-18 | 0.343424816 | 0.533 | 0.214 | 5.99E-14 | Proliferating | MEA1 |
| PAK11 | 2.11E-18 | 0.377092229 | 0.497 | 0.197 | 6.57E-14 | Proliferating | PAK1 |
| MT-CO21 | 2.11E-18 | 0.486261719 | 1 | 0.996 | 6.58E-14 | Proliferating | MT-CO2 |
| GUSB1 | 2.35E-18 | 0.413526945 | 0.552 | 0.234 | 7.32E-14 | Proliferating | GUSB |
| IMPDH2 | 2.84E-18 | 0.317350021 | 0.442 | 0.166 | 8.84E-14 | Proliferating | IMPDH2 |
| LMO4 | 2.93E-18 | 0.352257933 | 0.485 | 0.191 | 9.10E-14 | Proliferating | LMO4 |
| GLO1 | 2.98E-18 | 0.400899471 | 0.461 | 0.177 | 9.27E-14 | Proliferating | GLO1 |
| RHEB | 3.10E-18 | 0.632605841 | 0.873 | 0.588 | 9.65E-14 | Proliferating | RHEB |
| RASSF1 | 3.25E-18 | 0.282163529 | 0.297 | 0.09 | 1.01E-13 | Proliferating | RASSF1 |
| NUP50 | 3.52E-18 | 0.310444079 | 0.285 | 0.084 | 1.10E-13 | Proliferating | NUP50 |
| ADAM15 | 3.72E-18 | 0.315144078 | 0.321 | 0.102 | 1.16E-13 | Proliferating | ADAM15 |
| RNPEP | 3.73E-18 | 0.388481981 | 0.539 | 0.233 | 1.16E-13 | Proliferating | RNPEP |
| LMNA3 | 3.74E-18 | 0.699269333 | 0.873 | 0.535 | 1.16E-13 | Proliferating | LMNA |
| SF3A3 | 4.07E-18 | 0.324624646 | 0.309 | 0.098 | 1.27E-13 | Proliferating | SF3A3 |
| MRPS12 | 4.37E-18 | 0.412729338 | 0.503 | 0.21 | 1.36E-13 | Proliferating | MRPS12 |
| SNRPC1 | 4.58E-18 | 0.498765381 | 0.6 | 0.286 | 1.42E-13 | Proliferating | SNRPC |
| ST141 | 4.59E-18 | 0.400372536 | 0.503 | 0.205 | 1.43E-13 | Proliferating | ST14 |
| NHP2 | 4.67E-18 | 0.493388316 | 0.661 | 0.324 | 1.45E-13 | Proliferating | NHP2 |
| MBD3 | 5.36E-18 | 0.2607113 | 0.248 | 0.068 | 1.67E-13 | Proliferating | MBD3 |
| AKIP1 | 5.41E-18 | 0.350725138 | 0.327 | 0.108 | 1.68E-13 | Proliferating | AKIP1 |
| EIF2S2 | 6.66E-18 | 0.488840808 | 0.721 | 0.383 | 2.07E-13 | Proliferating | EIF2S2 |
| ICT1 | 6.98E-18 | 0.270888907 | 0.279 | 0.082 | 2.17E-13 | Proliferating | ICT1 |
| MTCH2 | 7.14E-18 | 0.474930348 | 0.618 | 0.286 | 2.22E-13 | Proliferating | MTCH2 |
| ARPC5L | 7.58E-18 | 0.349876005 | 0.442 | 0.174 | 2.36E-13 | Proliferating | ARPC5L |
| SLC39A3 | 8.03E-18 | 0.285755125 | 0.388 | 0.138 | 2.50E-13 | Proliferating | SLC39A3 |
| HNRNPD | 8.48E-18 | 0.45478692 | 0.648 | 0.313 | 2.64E-13 | Proliferating | HNRNPD |
| STOML2 | 8.90E-18 | 0.41957923 | 0.545 | 0.238 | 2.77E-13 | Proliferating | STOML2 |
| SMIM20 | 8.93E-18 | 0.350966316 | 0.473 | 0.187 | 2.78E-13 | Proliferating | SMIM20 |
| PRDX3 | 1.15E-17 | 0.529088503 | 0.764 | 0.425 | 3.59E-13 | Proliferating | PRDX3 |
| ECM1 | 1.19E-17 | 0.279873262 | 0.248 | 0.068 | 3.69E-13 | Proliferating | ECM1 |
| ARL22 | 1.24E-17 | 0.43559085 | 0.545 | 0.245 | 3.84E-13 | Proliferating | ARL2 |
| CISD31 | 1.38E-17 | 0.416597857 | 0.648 | 0.307 | 4.30E-13 | Proliferating | CISD3 |
| ELAVL1 | 1.41E-17 | 0.394746421 | 0.491 | 0.201 | 4.40E-13 | Proliferating | ELAVL1 |
| ARL6IP6 | 1.45E-17 | 0.306926349 | 0.327 | 0.108 | 4.50E-13 | Proliferating | ARL6IP6 |
| HNRNPUL2 | 1.46E-17 | 0.268860005 | 0.388 | 0.14 | 4.55E-13 | Proliferating | HNRNPUL2 |
| CEP57 | 1.50E-17 | 0.259407388 | 0.255 | 0.073 | 4.67E-13 | Proliferating | CEP57 |
| APEH | 1.51E-17 | 0.286792312 | 0.309 | 0.1 | 4.70E-13 | Proliferating | APEH |
| CACYBP1 | 1.59E-17 | 0.328733759 | 0.752 | 0.377 | 4.94E-13 | Proliferating | CACYBP |
| EBNA1BP2 | 1.65E-17 | 0.310821836 | 0.376 | 0.136 | 5.14E-13 | Proliferating | EBNA1BP2 |
| TM7SF3 | 1.70E-17 | 0.294553126 | 0.321 | 0.104 | 5.29E-13 | Proliferating | TM7SF3 |
| MRPL572 | 1.72E-17 | 0.558682152 | 0.642 | 0.332 | 5.36E-13 | Proliferating | MRPL57 |
| TOMM40 | 1.74E-17 | 0.297560264 | 0.382 | 0.137 | 5.42E-13 | Proliferating | TOMM40 |
| MAPRE2 | 1.89E-17 | 0.273313611 | 0.37 | 0.128 | 5.89E-13 | Proliferating | MAPRE2 |
| CDC27 | 2.18E-17 | 0.351876108 | 0.279 | 0.086 | 6.78E-13 | Proliferating | CDC27 |
| RPN21 | 2.41E-17 | 0.523820581 | 0.733 | 0.396 | 7.49E-13 | Proliferating | RPN2 |
| EIF2S1 | 2.50E-17 | 0.3391921 | 0.424 | 0.161 | 7.78E-13 | Proliferating | EIF2S1 |
| GMPPB | 2.52E-17 | 0.289231416 | 0.297 | 0.094 | 7.84E-13 | Proliferating | GMPPB |
| RBBP4 | 3.22E-17 | 0.359012323 | 0.461 | 0.183 | 1.00E-12 | Proliferating | RBBP4 |
| CLECL11 | 3.65E-17 | 0.256691696 | 0.273 | 0.082 | 1.14E-12 | Proliferating | CLECL1 |
| NUDT5 | 4.29E-17 | 0.416040492 | 0.442 | 0.18 | 1.33E-12 | Proliferating | NUDT5 |
| PDCD5 | 4.54E-17 | 0.427733263 | 0.588 | 0.269 | 1.41E-12 | Proliferating | PDCD5 |
| UQCRH | 5.54E-17 | 0.59669101 | 0.958 | 0.752 | 1.72E-12 | Proliferating | UQCRH |
| HAUS2 | 6.42E-17 | 0.281350805 | 0.242 | 0.067 | 2.00E-12 | Proliferating | HAUS2 |
| LAGE3 | 7.96E-17 | 0.389281367 | 0.588 | 0.27 | 2.48E-12 | Proliferating | LAGE3 |
| MT-ND6 | 8.25E-17 | 0.320045599 | 0.461 | 0.181 | 2.57E-12 | Proliferating | MT-ND6 |
| MGMT | 8.36E-17 | 0.377406026 | 0.479 | 0.2 | 2.60E-12 | Proliferating | MGMT |
| HNRNPA2B11 | 9.00E-17 | 0.519795301 | 0.945 | 0.764 | 2.80E-12 | Proliferating | HNRNPA2B1 |
| CBX3 | 9.08E-17 | 0.473352078 | 0.745 | 0.402 | 2.82E-12 | Proliferating | CBX3 |
| CAMK11 | 9.67E-17 | 0.33259636 | 0.509 | 0.213 | 3.01E-12 | Proliferating | CAMK1 |
| DAZAP1 | 1.01E-16 | 0.337508667 | 0.467 | 0.186 | 3.15E-12 | Proliferating | DAZAP1 |
| ARHGDIB2 | 1.08E-16 | 0.588836802 | 0.921 | 0.701 | 3.35E-12 | Proliferating | ARHGDIB |
| EIF5B | 1.08E-16 | 0.338830322 | 0.576 | 0.257 | 3.37E-12 | Proliferating | EIF5B |
| PSMD1 | 1.12E-16 | 0.289328529 | 0.436 | 0.17 | 3.49E-12 | Proliferating | PSMD1 |
| KHSRP | 1.13E-16 | 0.2521576 | 0.339 | 0.115 | 3.51E-12 | Proliferating | KHSRP |
| CBFB | 1.14E-16 | 0.292139962 | 0.4 | 0.15 | 3.54E-12 | Proliferating | CBFB |
| ENOPH1 | 1.19E-16 | 0.290629136 | 0.352 | 0.123 | 3.70E-12 | Proliferating | ENOPH1 |
| KIAA2013 | 1.31E-16 | 0.262191844 | 0.315 | 0.105 | 4.08E-12 | Proliferating | KIAA2013 |
| MGST21 | 1.48E-16 | 0.523065727 | 0.624 | 0.309 | 4.60E-12 | Proliferating | MGST2 |
| SLC43A3 | 1.67E-16 | 0.455489788 | 0.6 | 0.279 | 5.20E-12 | Proliferating | SLC43A3 |
| REEP4 | 1.67E-16 | 0.293118105 | 0.327 | 0.113 | 5.20E-12 | Proliferating | REEP4 |
| CCDC53 | 1.72E-16 | 0.307981036 | 0.364 | 0.134 | 5.36E-12 | Proliferating | CCDC53 |
| HDAC2 | 1.77E-16 | 0.330472731 | 0.521 | 0.226 | 5.50E-12 | Proliferating | HDAC2 |
| IKBIP | 1.83E-16 | 0.320767516 | 0.352 | 0.125 | 5.70E-12 | Proliferating | IKBIP |
| MRPL55 | 2.12E-16 | 0.335827864 | 0.594 | 0.275 | 6.61E-12 | Proliferating | MRPL55 |
| MRPS25 | 2.24E-16 | 0.302763533 | 0.382 | 0.143 | 6.97E-12 | Proliferating | MRPS25 |
| AKR1B12 | 2.34E-16 | 0.471292205 | 0.685 | 0.352 | 7.28E-12 | Proliferating | AKR1B1 |
| SSNA1 | 2.46E-16 | 0.403664293 | 0.612 | 0.286 | 7.64E-12 | Proliferating | SSNA1 |
| HNRNPA3 | 2.69E-16 | 0.543474046 | 0.891 | 0.597 | 8.37E-12 | Proliferating | HNRNPA3 |
| RTF1 | 2.70E-16 | 0.363513655 | 0.467 | 0.195 | 8.40E-12 | Proliferating | RTF1 |
| SMIM19 | 2.74E-16 | 0.352295724 | 0.473 | 0.2 | 8.52E-12 | Proliferating | SMIM19 |
| BCL7C | 2.74E-16 | 0.3006909 | 0.315 | 0.107 | 8.54E-12 | Proliferating | BCL7C |
| NME1 | 2.83E-16 | 0.484734754 | 0.612 | 0.3 | 8.81E-12 | Proliferating | NME1 |
| ATPIF12 | 2.90E-16 | 0.56095447 | 0.758 | 0.416 | 9.01E-12 | Proliferating | ATPIF1 |
| ZDHHC24 | 3.03E-16 | 0.285163903 | 0.394 | 0.148 | 9.42E-12 | Proliferating | ZDHHC24 |
| ACAT1 | 3.07E-16 | 0.276660773 | 0.339 | 0.119 | 9.54E-12 | Proliferating | ACAT1 |
| UQCRC1 | 3.70E-16 | 0.464233071 | 0.733 | 0.399 | 1.15E-11 | Proliferating | UQCRC1 |
| SRSF1 | 3.77E-16 | 0.267430847 | 0.455 | 0.182 | 1.17E-11 | Proliferating | SRSF1 |
| APOBEC3C | 4.28E-16 | 0.281499782 | 0.382 | 0.14 | 1.33E-11 | Proliferating | APOBEC3C |
| CALU1 | 4.30E-16 | 0.294764829 | 0.552 | 0.238 | 1.34E-11 | Proliferating | CALU |
| PIEZO1 | 4.56E-16 | 0.263903244 | 0.242 | 0.07 | 1.42E-11 | Proliferating | PIEZO1 |
| CLTA2 | 4.60E-16 | 0.613997116 | 0.867 | 0.564 | 1.43E-11 | Proliferating | CLTA |
| SCCPDH1 | 4.70E-16 | 0.379346513 | 0.37 | 0.139 | 1.46E-11 | Proliferating | SCCPDH |
| G3BP11 | 4.81E-16 | 0.418765915 | 0.539 | 0.241 | 1.50E-11 | Proliferating | G3BP1 |
| YBX11 | 5.11E-16 | 0.500665269 | 0.994 | 0.898 | 1.59E-11 | Proliferating | YBX1 |
| TXN21 | 5.16E-16 | 0.38092107 | 0.594 | 0.281 | 1.61E-11 | Proliferating | TXN2 |
| MRPL15 | 5.19E-16 | 0.271306424 | 0.388 | 0.144 | 1.61E-11 | Proliferating | MRPL15 |
| HADHA1 | 5.60E-16 | 0.346750635 | 0.606 | 0.284 | 1.74E-11 | Proliferating | HADHA |
| GAL3ST42 | 5.76E-16 | 0.283640193 | 0.345 | 0.122 | 1.79E-11 | Proliferating | GAL3ST4 |
| TAF15 | 6.32E-16 | 0.363460387 | 0.364 | 0.132 | 1.97E-11 | Proliferating | TAF15 |
| PITHD1 | 6.35E-16 | 0.254097252 | 0.43 | 0.167 | 1.98E-11 | Proliferating | PITHD1 |
| ME21 | 6.76E-16 | 0.438872535 | 0.503 | 0.223 | 2.10E-11 | Proliferating | ME2 |
| MMP92 | 8.31E-16 | 0.65412545 | 0.703 | 0.39 | 2.59E-11 | Proliferating | MMP9 |
| DGCR6L | 8.34E-16 | 0.364618162 | 0.509 | 0.228 | 2.59E-11 | Proliferating | DGCR6L |
| CWC15 | 9.52E-16 | 0.266502471 | 0.509 | 0.212 | 2.96E-11 | Proliferating | CWC15 |
| NDUFAF3 | 1.01E-15 | 0.451568793 | 0.709 | 0.376 | 3.14E-11 | Proliferating | NDUFAF3 |
| POLR2F | 1.08E-15 | 0.346317217 | 0.618 | 0.298 | 3.36E-11 | Proliferating | POLR2F |
| PDAP1 | 1.09E-15 | 0.307677956 | 0.545 | 0.244 | 3.39E-11 | Proliferating | PDAP1 |
| PFKL | 1.25E-15 | 0.42013505 | 0.582 | 0.275 | 3.90E-11 | Proliferating | PFKL |
| FARSB | 1.42E-15 | 0.254050048 | 0.206 | 0.055 | 4.41E-11 | Proliferating | FARSB |
| ARHGAP4 | 1.61E-15 | 0.346973663 | 0.485 | 0.208 | 5.02E-11 | Proliferating | ARHGAP4 |
| CALM31 | 1.67E-15 | 0.56229424 | 0.867 | 0.576 | 5.20E-11 | Proliferating | CALM3 |
| PRDX2 | 1.68E-15 | 0.409521552 | 0.515 | 0.235 | 5.21E-11 | Proliferating | PRDX2 |
| PSMG1 | 1.74E-15 | 0.303766636 | 0.303 | 0.105 | 5.41E-11 | Proliferating | PSMG1 |
| AP1B12 | 1.77E-15 | 0.39411668 | 0.527 | 0.241 | 5.50E-11 | Proliferating | AP1B1 |
| HSP90B11 | 1.78E-15 | 0.505637154 | 0.933 | 0.697 | 5.53E-11 | Proliferating | HSP90B1 |
| RANGAP1 | 1.90E-15 | 0.297381113 | 0.236 | 0.07 | 5.91E-11 | Proliferating | RANGAP1 |
| GNAI21 | 1.97E-15 | 0.547582635 | 0.891 | 0.561 | 6.14E-11 | Proliferating | GNAI2 |
| PDIA41 | 2.22E-15 | 0.370007957 | 0.703 | 0.349 | 6.92E-11 | Proliferating | PDIA4 |
| LGALS3BP2 | 2.26E-15 | 0.438722862 | 0.6 | 0.28 | 7.05E-11 | Proliferating | LGALS3BP |
| CCAR1 | 2.29E-15 | 0.302446268 | 0.382 | 0.146 | 7.13E-11 | Proliferating | CCAR1 |
| TTYH32 | 2.37E-15 | 0.388205604 | 0.648 | 0.316 | 7.36E-11 | Proliferating | TTYH3 |
| CCND31 | 2.46E-15 | 0.334600002 | 0.436 | 0.18 | 7.66E-11 | Proliferating | CCND3 |
| FIBP | 2.47E-15 | 0.336601473 | 0.648 | 0.304 | 7.69E-11 | Proliferating | FIBP |
| IMMT | 2.54E-15 | 0.295724803 | 0.376 | 0.144 | 7.90E-11 | Proliferating | IMMT |
| MRPL3 | 2.55E-15 | 0.309232883 | 0.412 | 0.165 | 7.92E-11 | Proliferating | MRPL3 |
| TUBB4B | 3.02E-15 | 0.490512545 | 0.812 | 0.468 | 9.41E-11 | Proliferating | TUBB4B |
| YWHAE | 3.04E-15 | 0.496985982 | 0.933 | 0.618 | 9.46E-11 | Proliferating | YWHAE |
| COLGALT1 | 3.16E-15 | 0.34657861 | 0.497 | 0.217 | 9.84E-11 | Proliferating | COLGALT1 |
| PPT12 | 3.46E-15 | 0.501824586 | 0.879 | 0.571 | 1.08E-10 | Proliferating | PPT1 |
| TPR | 3.52E-15 | 0.279940472 | 0.4 | 0.159 | 1.09E-10 | Proliferating | TPR |
| SRP9 | 3.71E-15 | 0.441246868 | 0.727 | 0.406 | 1.16E-10 | Proliferating | SRP9 |
| SNX51 | 3.75E-15 | 0.307168907 | 0.545 | 0.249 | 1.17E-10 | Proliferating | SNX5 |
| MT-ND41 | 4.40E-15 | 0.391054164 | 0.994 | 0.996 | 1.37E-10 | Proliferating | MT-ND4 |
| PLGRKT | 4.85E-15 | 0.37704197 | 0.564 | 0.26 | 1.51E-10 | Proliferating | PLGRKT |
| RPL26L1 | 4.88E-15 | 0.37554018 | 0.491 | 0.221 | 1.52E-10 | Proliferating | RPL26L1 |
| ACP1 | 4.90E-15 | 0.376534534 | 0.57 | 0.267 | 1.52E-10 | Proliferating | ACP1 |
| PSMC5 | 5.00E-15 | 0.429649476 | 0.642 | 0.337 | 1.55E-10 | Proliferating | PSMC5 |
| GPX12 | 5.45E-15 | 0.45856547 | 1 | 0.932 | 1.69E-10 | Proliferating | GPX1 |
| CMC1 | 5.88E-15 | 0.325117592 | 0.4 | 0.162 | 1.83E-10 | Proliferating | CMC1 |
| MRPS33 | 6.70E-15 | 0.29755132 | 0.467 | 0.198 | 2.09E-10 | Proliferating | MRPS33 |
| TUBB6 | 6.80E-15 | 0.280255798 | 0.394 | 0.155 | 2.12E-10 | Proliferating | TUBB6 |
| NTAN11 | 7.00E-15 | 0.271329794 | 0.43 | 0.176 | 2.18E-10 | Proliferating | NTAN1 |
| XRCC61 | 7.12E-15 | 0.383788048 | 0.661 | 0.336 | 2.21E-10 | Proliferating | XRCC6 |
| CAPRIN1 | 7.31E-15 | 0.293882957 | 0.424 | 0.172 | 2.28E-10 | Proliferating | CAPRIN1 |
| TIAL1 | 7.83E-15 | 0.297243113 | 0.436 | 0.181 | 2.44E-10 | Proliferating | TIAL1 |
| NDUFAB1 | 8.66E-15 | 0.423816777 | 0.739 | 0.412 | 2.70E-10 | Proliferating | NDUFAB1 |
| PTGES31 | 8.99E-15 | 0.498732105 | 0.836 | 0.525 | 2.80E-10 | Proliferating | PTGES3 |
| CCDC25 | 9.79E-15 | 0.352506364 | 0.315 | 0.113 | 3.05E-10 | Proliferating | CCDC25 |
| CYC11 | 9.84E-15 | 0.411874078 | 0.691 | 0.373 | 3.06E-10 | Proliferating | CYC1 |
| CFL11 | 1.02E-14 | 0.428034564 | 0.988 | 0.937 | 3.16E-10 | Proliferating | CFL1 |
| VSIG42 | 1.04E-14 | 0.488206914 | 0.57 | 0.278 | 3.22E-10 | Proliferating | VSIG4 |
| PARK72 | 1.06E-14 | 0.544764865 | 0.848 | 0.559 | 3.28E-10 | Proliferating | PARK7 |
| RBX11 | 1.18E-14 | 0.524004304 | 0.897 | 0.596 | 3.66E-10 | Proliferating | RBX1 |
| RALY1 | 1.34E-14 | 0.423806014 | 0.667 | 0.345 | 4.17E-10 | Proliferating | RALY |
| MS4A6A1 | 1.40E-14 | 0.495583249 | 0.927 | 0.612 | 4.36E-10 | Proliferating | MS4A6A |
| DEGS1 | 1.45E-14 | 0.321745507 | 0.533 | 0.24 | 4.50E-10 | Proliferating | DEGS1 |
| PSMC6 | 1.55E-14 | 0.263060553 | 0.497 | 0.217 | 4.81E-10 | Proliferating | PSMC6 |
| MT-ND51 | 1.66E-14 | 0.620728645 | 0.97 | 0.92 | 5.17E-10 | Proliferating | MT-ND5 |
| HNRNPA11 | 1.78E-14 | 0.522317688 | 0.939 | 0.786 | 5.53E-10 | Proliferating | HNRNPA1 |
| SF3B2 | 1.78E-14 | 0.339225874 | 0.588 | 0.283 | 5.54E-10 | Proliferating | SF3B2 |
| ATP5A12 | 1.94E-14 | 0.453936337 | 0.727 | 0.391 | 6.02E-10 | Proliferating | ATP5A1 |
| NGFRAP11 | 1.98E-14 | 0.383522995 | 0.533 | 0.246 | 6.15E-10 | Proliferating | NGFRAP1 |
| NDUFC22 | 2.00E-14 | 0.448957015 | 0.812 | 0.49 | 6.23E-10 | Proliferating | NDUFC2 |
| PKIB1 | 2.13E-14 | 0.405596789 | 0.321 | 0.117 | 6.64E-10 | Proliferating | PKIB |
| AAMDC | 2.29E-14 | 0.283684243 | 0.297 | 0.103 | 7.12E-10 | Proliferating | AAMDC |
| SUMO2 | 2.34E-14 | 0.43190202 | 0.939 | 0.77 | 7.29E-10 | Proliferating | SUMO2 |
| TGFBI1 | 2.36E-14 | 0.508689184 | 0.927 | 0.65 | 7.33E-10 | Proliferating | TGFBI |
| NDUFB71 | 2.40E-14 | 0.422713748 | 0.855 | 0.538 | 7.48E-10 | Proliferating | NDUFB7 |
| TARDBP | 2.46E-14 | 0.289686986 | 0.358 | 0.137 | 7.66E-10 | Proliferating | TARDBP |
| CCT6A | 2.64E-14 | 0.381150203 | 0.6 | 0.3 | 8.20E-10 | Proliferating | CCT6A |
| SDC32 | 2.65E-14 | 0.350256469 | 0.376 | 0.148 | 8.25E-10 | Proliferating | SDC3 |
| TMEM261 | 2.71E-14 | 0.315779754 | 0.43 | 0.183 | 8.44E-10 | Proliferating | TMEM261 |
| NAA50 | 2.83E-14 | 0.273358091 | 0.491 | 0.213 | 8.80E-10 | Proliferating | NAA50 |
| TTC1 | 3.11E-14 | 0.263004759 | 0.4 | 0.163 | 9.66E-10 | Proliferating | TTC1 |
| POLE4 | 3.26E-14 | 0.390534341 | 0.679 | 0.363 | 1.01E-09 | Proliferating | POLE4 |
| ATP2A2 | 3.30E-14 | 0.325714045 | 0.57 | 0.27 | 1.03E-09 | Proliferating | ATP2A2 |
| RAB32 | 3.73E-14 | 0.339936599 | 0.661 | 0.327 | 1.16E-09 | Proliferating | RAB32 |
| CISD1 | 3.78E-14 | 0.278375983 | 0.376 | 0.148 | 1.18E-09 | Proliferating | CISD1 |
| AUP1 | 3.95E-14 | 0.374832656 | 0.691 | 0.356 | 1.23E-09 | Proliferating | AUP1 |
| XRN2 | 3.97E-14 | 0.288343771 | 0.618 | 0.3 | 1.23E-09 | Proliferating | XRN2 |
| AP2A22 | 3.98E-14 | 0.34821396 | 0.388 | 0.157 | 1.24E-09 | Proliferating | AP2A2 |
| NPM3 | 3.99E-14 | 0.301501147 | 0.345 | 0.134 | 1.24E-09 | Proliferating | NPM3 |
| RBMX1 | 4.88E-14 | 0.405994113 | 0.594 | 0.292 | 1.52E-09 | Proliferating | RBMX |
| UCHL3 | 4.99E-14 | 0.250392092 | 0.436 | 0.183 | 1.55E-09 | Proliferating | UCHL3 |
| NARS1 | 5.26E-14 | 0.266070731 | 0.491 | 0.217 | 1.64E-09 | Proliferating | NARS |
| C20orf24 | 5.51E-14 | 0.323875439 | 0.752 | 0.403 | 1.72E-09 | Proliferating | C20orf24 |
| UBE2M | 5.88E-14 | 0.270227754 | 0.327 | 0.12 | 1.83E-09 | Proliferating | UBE2M |
| MESDC2 | 5.89E-14 | 0.367888784 | 0.509 | 0.235 | 1.83E-09 | Proliferating | MESDC2 |
| PPP2R4 | 6.38E-14 | 0.253224122 | 0.321 | 0.12 | 1.98E-09 | Proliferating | PPP2R4 |
| TSPAN3 | 6.44E-14 | 0.295108509 | 0.388 | 0.159 | 2.00E-09 | Proliferating | TSPAN3 |
| CHCHD1 | 6.69E-14 | 0.301720327 | 0.467 | 0.206 | 2.08E-09 | Proliferating | CHCHD1 |
| HPRT1 | 6.78E-14 | 0.267171 | 0.552 | 0.255 | 2.11E-09 | Proliferating | HPRT1 |
| TUFM2 | 7.76E-14 | 0.407586804 | 0.673 | 0.378 | 2.41E-09 | Proliferating | TUFM |
| POLR2K | 7.96E-14 | 0.394386809 | 0.618 | 0.327 | 2.48E-09 | Proliferating | POLR2K |
| RPLP01 | 8.02E-14 | 0.455319309 | 0.97 | 0.882 | 2.50E-09 | Proliferating | RPLP0 |
| TMEM141 | 8.64E-14 | 0.355469891 | 0.509 | 0.238 | 2.69E-09 | Proliferating | TMEM141 |
| NDUFB101 | 8.93E-14 | 0.433022226 | 0.752 | 0.441 | 2.78E-09 | Proliferating | NDUFB10 |
| CCDC124 | 8.96E-14 | 0.283048797 | 0.545 | 0.246 | 2.79E-09 | Proliferating | CCDC124 |
| LARP1 | 9.31E-14 | 0.290213391 | 0.394 | 0.164 | 2.90E-09 | Proliferating | LARP1 |
| ATP5F1 | 9.79E-14 | 0.435516029 | 0.794 | 0.493 | 3.05E-09 | Proliferating | ATP5F1 |
| DDOST2 | 1.07E-13 | 0.304744115 | 0.539 | 0.25 | 3.33E-09 | Proliferating | DDOST |
| DYNC1I2 | 1.09E-13 | 0.317562306 | 0.521 | 0.243 | 3.40E-09 | Proliferating | DYNC1I2 |
| VOPP11 | 1.12E-13 | 0.385122328 | 0.636 | 0.331 | 3.49E-09 | Proliferating | VOPP1 |
| ATP5L | 1.18E-13 | 0.445089159 | 0.97 | 0.853 | 3.68E-09 | Proliferating | ATP5L |
| ILF3 | 1.34E-13 | 0.302463229 | 0.515 | 0.241 | 4.18E-09 | Proliferating | ILF3 |
| ATP5J | 1.35E-13 | 0.413138645 | 0.921 | 0.589 | 4.20E-09 | Proliferating | ATP5J |
| DHRS4 | 1.41E-13 | 0.300034606 | 0.358 | 0.142 | 4.39E-09 | Proliferating | DHRS4 |
| NIT2 | 1.44E-13 | 0.276467928 | 0.345 | 0.137 | 4.49E-09 | Proliferating | NIT2 |
| CHCHD5 | 1.46E-13 | 0.300464196 | 0.515 | 0.237 | 4.53E-09 | Proliferating | CHCHD5 |
| ATP5O | 1.55E-13 | 0.470389178 | 0.897 | 0.62 | 4.83E-09 | Proliferating | ATP5O |
| COX20 | 1.68E-13 | 0.306772601 | 0.503 | 0.236 | 5.22E-09 | Proliferating | COX20 |
| GSTP12 | 1.77E-13 | 0.479248985 | 0.958 | 0.795 | 5.52E-09 | Proliferating | GSTP1 |
| NUTF2 | 1.86E-13 | 0.300354326 | 0.588 | 0.286 | 5.80E-09 | Proliferating | NUTF2 |
| COA4 | 1.90E-13 | 0.262140966 | 0.43 | 0.184 | 5.91E-09 | Proliferating | COA4 |
| AKR1A11 | 1.92E-13 | 0.42287603 | 0.776 | 0.455 | 5.96E-09 | Proliferating | AKR1A1 |
| GSN3 | 2.00E-13 | 0.434433822 | 0.836 | 0.498 | 6.22E-09 | Proliferating | GSN |
| SPRED12 | 2.01E-13 | 0.276195961 | 0.461 | 0.202 | 6.26E-09 | Proliferating | SPRED1 |
| TMEM14A | 2.05E-13 | 0.265592352 | 0.255 | 0.086 | 6.39E-09 | Proliferating | TMEM14A |
| VKORC1 | 2.07E-13 | 0.361838033 | 0.733 | 0.402 | 6.44E-09 | Proliferating | VKORC1 |
| PPP1R14B | 2.10E-13 | 0.317560968 | 0.37 | 0.15 | 6.53E-09 | Proliferating | PPP1R14B |
| CDK2AP21 | 2.21E-13 | 0.346256915 | 0.436 | 0.194 | 6.89E-09 | Proliferating | CDK2AP2 |
| CCT2 | 2.37E-13 | 0.328149482 | 0.479 | 0.222 | 7.38E-09 | Proliferating | CCT2 |
| ATP5G31 | 2.38E-13 | 0.570456872 | 0.903 | 0.66 | 7.40E-09 | Proliferating | ATP5G3 |
| NDUFA41 | 2.60E-13 | 0.565462247 | 0.952 | 0.751 | 8.09E-09 | Proliferating | NDUFA4 |
| NCF41 | 2.61E-13 | 0.286587292 | 0.503 | 0.229 | 8.13E-09 | Proliferating | NCF4 |
| STARD7 | 2.63E-13 | 0.257865959 | 0.43 | 0.182 | 8.18E-09 | Proliferating | STARD7 |
| ASPH1 | 2.80E-13 | 0.253866196 | 0.485 | 0.217 | 8.70E-09 | Proliferating | ASPH |
| POLR2I | 2.90E-13 | 0.347653021 | 0.515 | 0.244 | 9.02E-09 | Proliferating | POLR2I |
| C1QBP2 | 3.08E-13 | 0.468724967 | 0.63 | 0.343 | 9.60E-09 | Proliferating | C1QBP |
| MGST32 | 3.23E-13 | 0.45376523 | 0.855 | 0.514 | 1.00E-08 | Proliferating | MGST3 |
| C19orf43 | 3.24E-13 | 0.425156988 | 0.855 | 0.602 | 1.01E-08 | Proliferating | C19orf43 |
| RUVBL2 | 3.54E-13 | 0.258592369 | 0.279 | 0.099 | 1.10E-08 | Proliferating | RUVBL2 |
| DNAJC1 | 3.66E-13 | 0.315225197 | 0.412 | 0.179 | 1.14E-08 | Proliferating | DNAJC1 |
| RASSF41 | 3.74E-13 | 0.362643863 | 0.618 | 0.314 | 1.16E-08 | Proliferating | RASSF4 |
| HNRNPUL11 | 3.93E-13 | 0.354448298 | 0.491 | 0.223 | 1.22E-08 | Proliferating | HNRNPUL1 |
| SNX62 | 4.21E-13 | 0.313985185 | 0.661 | 0.334 | 1.31E-08 | Proliferating | SNX6 |
| CD42 | 4.72E-13 | 0.428076926 | 0.733 | 0.419 | 1.47E-08 | Proliferating | CD4 |
| SNCA | 4.83E-13 | 0.271602049 | 0.376 | 0.152 | 1.50E-08 | Proliferating | SNCA |
| SRM | 5.00E-13 | 0.412845337 | 0.4 | 0.179 | 1.56E-08 | Proliferating | SRM |
| DPP72 | 5.07E-13 | 0.455821317 | 0.752 | 0.46 | 1.58E-08 | Proliferating | DPP7 |
| RBM17 | 5.22E-13 | 0.355576381 | 0.618 | 0.321 | 1.62E-08 | Proliferating | RBM17 |
| XRCC5 | 5.57E-13 | 0.374758674 | 0.57 | 0.294 | 1.73E-08 | Proliferating | XRCC5 |
| AURKAIP1 | 5.60E-13 | 0.424455191 | 0.8 | 0.495 | 1.74E-08 | Proliferating | AURKAIP1 |
| MRPL13 | 5.82E-13 | 0.27406759 | 0.442 | 0.195 | 1.81E-08 | Proliferating | MRPL13 |
| PFDN4 | 6.09E-13 | 0.271698608 | 0.424 | 0.184 | 1.90E-08 | Proliferating | PFDN4 |
| MRPS71 | 6.15E-13 | 0.275407384 | 0.497 | 0.23 | 1.91E-08 | Proliferating | MRPS7 |
| LSM8 | 6.16E-13 | 0.331719106 | 0.576 | 0.292 | 1.92E-08 | Proliferating | LSM8 |
| GANAB | 6.50E-13 | 0.281156977 | 0.43 | 0.189 | 2.02E-08 | Proliferating | GANAB |
| MRPL4 | 6.50E-13 | 0.289108749 | 0.394 | 0.168 | 2.02E-08 | Proliferating | MRPL4 |
| CAPG2 | 6.70E-13 | 0.530924279 | 0.933 | 0.735 | 2.09E-08 | Proliferating | CAPG |
| TMEM371 | 7.03E-13 | 0.293512685 | 0.285 | 0.105 | 2.19E-08 | Proliferating | TMEM37 |
| GTF3A | 7.15E-13 | 0.376517707 | 0.703 | 0.388 | 2.22E-08 | Proliferating | GTF3A |
| LRRC59 | 7.26E-13 | 0.341290496 | 0.558 | 0.273 | 2.26E-08 | Proliferating | LRRC59 |
| EIF1AX | 8.53E-13 | 0.455739626 | 0.715 | 0.43 | 2.65E-08 | Proliferating | EIF1AX |
| ABI31 | 8.64E-13 | 0.342813077 | 0.497 | 0.238 | 2.69E-08 | Proliferating | ABI3 |
| ATP5D | 8.73E-13 | 0.452704904 | 0.909 | 0.639 | 2.72E-08 | Proliferating | ATP5D |
| C22 | 8.83E-13 | 0.360562894 | 0.461 | 0.21 | 2.75E-08 | Proliferating | C2 |
| MDH11 | 8.98E-13 | 0.426008305 | 0.552 | 0.285 | 2.79E-08 | Proliferating | MDH1 |
| COX6C2 | 1.08E-12 | 0.513721986 | 0.952 | 0.715 | 3.37E-08 | Proliferating | COX6C |
| TIMM9 | 1.18E-12 | 0.252740037 | 0.291 | 0.109 | 3.66E-08 | Proliferating | TIMM9 |
| CLEC11A1 | 1.19E-12 | 0.550066553 | 0.43 | 0.203 | 3.69E-08 | Proliferating | CLEC11A |
| CD1511 | 1.20E-12 | 0.354600885 | 0.606 | 0.321 | 3.74E-08 | Proliferating | CD151 |
| UCP22 | 1.21E-12 | 0.468112194 | 0.806 | 0.497 | 3.76E-08 | Proliferating | UCP2 |
| SRSF9 | 1.26E-12 | 0.40187623 | 0.758 | 0.444 | 3.91E-08 | Proliferating | SRSF9 |
| DUSP231 | 1.27E-12 | 0.304308044 | 0.618 | 0.324 | 3.94E-08 | Proliferating | DUSP23 |
| ATG31 | 1.59E-12 | 0.413908098 | 0.721 | 0.418 | 4.95E-08 | Proliferating | ATG3 |
| RPS201 | 1.60E-12 | 0.344042773 | 0.994 | 0.977 | 4.98E-08 | Proliferating | RPS20 |
| ATRAID1 | 1.61E-12 | 0.366941048 | 0.655 | 0.347 | 5.01E-08 | Proliferating | ATRAID |
| SHISA5 | 1.75E-12 | 0.312852737 | 0.442 | 0.196 | 5.43E-08 | Proliferating | SHISA5 |
| SRD5A3 | 1.90E-12 | 0.345567562 | 0.345 | 0.145 | 5.91E-08 | Proliferating | SRD5A3 |
| SLC25A11 | 1.91E-12 | 0.311294262 | 0.491 | 0.235 | 5.94E-08 | Proliferating | SLC25A11 |
| C20orf271 | 1.91E-12 | 0.294462917 | 0.509 | 0.242 | 5.95E-08 | Proliferating | C20orf27 |
| ENY2 | 2.10E-12 | 0.448195159 | 0.867 | 0.601 | 6.53E-08 | Proliferating | ENY2 |
| MAD2L21 | 2.23E-12 | 0.312935568 | 0.412 | 0.184 | 6.94E-08 | Proliferating | MAD2L2 |
| MMADHC | 2.40E-12 | 0.260047633 | 0.552 | 0.266 | 7.48E-08 | Proliferating | MMADHC |
| PSMD7 | 2.47E-12 | 0.299433461 | 0.648 | 0.341 | 7.68E-08 | Proliferating | PSMD7 |
| RAD23A | 2.49E-12 | 0.354173039 | 0.691 | 0.388 | 7.74E-08 | Proliferating | RAD23A |
| EIF3B | 2.61E-12 | 0.259344379 | 0.352 | 0.142 | 8.11E-08 | Proliferating | EIF3B |
| DNPH11 | 2.67E-12 | 0.391793188 | 0.594 | 0.314 | 8.29E-08 | Proliferating | DNPH1 |
| HNRNPH3 | 2.67E-12 | 0.262563597 | 0.57 | 0.28 | 8.31E-08 | Proliferating | HNRNPH3 |
| SEPT7 | 2.70E-12 | 0.303616018 | 0.703 | 0.388 | 8.41E-08 | Proliferating | SEPT7 |
| ANKRD13A | 2.81E-12 | 0.262588496 | 0.242 | 0.083 | 8.74E-08 | Proliferating | ANKRD13A |
| UQCRQ | 2.97E-12 | 0.501421159 | 0.903 | 0.666 | 9.23E-08 | Proliferating | UQCRQ |
| NPC22 | 3.10E-12 | 0.388412965 | 0.982 | 0.935 | 9.65E-08 | Proliferating | NPC2 |
| HS3ST11 | 3.52E-12 | 0.388492449 | 0.345 | 0.145 | 1.09E-07 | Proliferating | HS3ST1 |
| NDUFB11 | 3.53E-12 | 0.406727776 | 0.903 | 0.62 | 1.10E-07 | Proliferating | NDUFB11 |
| ENSA1 | 3.63E-12 | 0.369474345 | 0.703 | 0.397 | 1.13E-07 | Proliferating | ENSA |
| GLRX3 | 3.65E-12 | 0.290614328 | 0.545 | 0.271 | 1.13E-07 | Proliferating | GLRX3 |
| SRRM1 | 3.65E-12 | 0.360575978 | 0.673 | 0.371 | 1.14E-07 | Proliferating | SRRM1 |
| TMEM19 | 3.70E-12 | 0.276642854 | 0.267 | 0.098 | 1.15E-07 | Proliferating | TMEM19 |
| RNPS1 | 3.72E-12 | 0.348193322 | 0.509 | 0.245 | 1.16E-07 | Proliferating | RNPS1 |
| PNKD | 3.73E-12 | 0.251000529 | 0.485 | 0.227 | 1.16E-07 | Proliferating | PNKD |
| CSK | 3.83E-12 | 0.290244187 | 0.436 | 0.2 | 1.19E-07 | Proliferating | CSK |
| PEBP12 | 4.09E-12 | 0.353003681 | 0.709 | 0.391 | 1.27E-07 | Proliferating | PEBP1 |
| MRPL54 | 4.14E-12 | 0.343133695 | 0.691 | 0.382 | 1.29E-07 | Proliferating | MRPL54 |
| RAC11 | 4.22E-12 | 0.414360295 | 0.988 | 0.849 | 1.31E-07 | Proliferating | RAC1 |
| TIMP22 | 4.73E-12 | 0.327811627 | 0.752 | 0.421 | 1.47E-07 | Proliferating | TIMP2 |
| ECHS11 | 4.74E-12 | 0.437111174 | 0.533 | 0.287 | 1.48E-07 | Proliferating | ECHS1 |
| CLNS1A1 | 4.87E-12 | 0.266709949 | 0.497 | 0.24 | 1.52E-07 | Proliferating | CLNS1A |
| SNRPG | 5.57E-12 | 0.528852947 | 0.782 | 0.531 | 1.73E-07 | Proliferating | SNRPG |
| SNX17 | 5.91E-12 | 0.278095537 | 0.564 | 0.28 | 1.84E-07 | Proliferating | SNX17 |
| RPS19BP12 | 6.16E-12 | 0.318666072 | 0.691 | 0.381 | 1.92E-07 | Proliferating | RPS19BP1 |
| PSMC21 | 6.57E-12 | 0.288099734 | 0.455 | 0.211 | 2.04E-07 | Proliferating | PSMC2 |
| SLC25A391 | 7.11E-12 | 0.380760319 | 0.594 | 0.314 | 2.21E-07 | Proliferating | SLC25A39 |
| DDT | 7.28E-12 | 0.353454834 | 0.788 | 0.478 | 2.27E-07 | Proliferating | DDT |
| TCEAL41 | 7.34E-12 | 0.318357647 | 0.485 | 0.231 | 2.28E-07 | Proliferating | TCEAL4 |
| TIMM13 | 7.51E-12 | 0.352024766 | 0.745 | 0.427 | 2.34E-07 | Proliferating | TIMM13 |
| MT-CO31 | 8.10E-12 | 0.338633 | 0.994 | 0.994 | 2.52E-07 | Proliferating | MT-CO3 |
| AXL2 | 8.16E-12 | 0.451719354 | 0.424 | 0.202 | 2.54E-07 | Proliferating | AXL |
| PLD32 | 8.31E-12 | 0.356993593 | 0.782 | 0.453 | 2.58E-07 | Proliferating | PLD3 |
| PSMD2 | 8.51E-12 | 0.291854209 | 0.461 | 0.22 | 2.65E-07 | Proliferating | PSMD2 |
| COMMD7 | 8.80E-12 | 0.293274704 | 0.491 | 0.233 | 2.74E-07 | Proliferating | COMMD7 |
| SERBP11 | 9.72E-12 | 0.428770103 | 0.739 | 0.454 | 3.03E-07 | Proliferating | SERBP1 |
| MRPL14 | 9.95E-12 | 0.288228008 | 0.594 | 0.302 | 3.09E-07 | Proliferating | MRPL14 |
| RWDD11 | 1.03E-11 | 0.339770977 | 0.655 | 0.354 | 3.21E-07 | Proliferating | RWDD1 |
| HNMT2 | 1.08E-11 | 0.383966284 | 0.739 | 0.432 | 3.35E-07 | Proliferating | HNMT |
| PPP1CC | 1.10E-11 | 0.336642374 | 0.527 | 0.265 | 3.42E-07 | Proliferating | PPP1CC |
| C1orf35 | 1.12E-11 | 0.25603231 | 0.255 | 0.092 | 3.49E-07 | Proliferating | C1orf35 |
| C17orf89 | 1.19E-11 | 0.299617447 | 0.473 | 0.224 | 3.69E-07 | Proliferating | C17orf89 |
| NRP22 | 1.20E-11 | 0.348275243 | 0.612 | 0.32 | 3.75E-07 | Proliferating | NRP2 |
| TXNDC17 | 1.31E-11 | 0.345952744 | 0.703 | 0.387 | 4.06E-07 | Proliferating | TXNDC17 |
| IFIT31 | 1.32E-11 | 0.65866942 | 0.37 | 0.169 | 4.10E-07 | Proliferating | IFIT3 |
| ANP32A | 1.40E-11 | 0.3163599 | 0.57 | 0.299 | 4.37E-07 | Proliferating | ANP32A |
| PGAM1 | 1.60E-11 | 0.422791328 | 0.836 | 0.568 | 4.98E-07 | Proliferating | PGAM1 |
| NPM11 | 1.72E-11 | 0.482115617 | 0.939 | 0.742 | 5.35E-07 | Proliferating | NPM1 |
| C3orf14 | 1.72E-11 | 0.253421483 | 0.218 | 0.074 | 5.36E-07 | Proliferating | C3orf14 |
| NDUFV1 | 1.79E-11 | 0.315883628 | 0.509 | 0.256 | 5.58E-07 | Proliferating | NDUFV1 |
| CAPZB1 | 1.95E-11 | 0.407559167 | 0.897 | 0.642 | 6.07E-07 | Proliferating | CAPZB |
| UQCR102 | 1.97E-11 | 0.413017666 | 0.891 | 0.623 | 6.14E-07 | Proliferating | UQCR10 |
| UBE2A | 1.98E-11 | 0.319621765 | 0.655 | 0.359 | 6.15E-07 | Proliferating | UBE2A |
| BAX | 2.03E-11 | 0.353605023 | 0.824 | 0.511 | 6.30E-07 | Proliferating | BAX |
| SRSF31 | 2.04E-11 | 0.376052657 | 0.873 | 0.607 | 6.34E-07 | Proliferating | SRSF3 |
| VTI1B1 | 2.08E-11 | 0.277951399 | 0.539 | 0.268 | 6.46E-07 | Proliferating | VTI1B |
| FAM96A1 | 2.24E-11 | 0.305922816 | 0.661 | 0.358 | 6.98E-07 | Proliferating | FAM96A |
| RPN11 | 2.30E-11 | 0.280032867 | 0.667 | 0.363 | 7.17E-07 | Proliferating | RPN1 |
| ARHGAP182 | 2.42E-11 | 0.386796872 | 0.624 | 0.343 | 7.52E-07 | Proliferating | ARHGAP18 |
| SUN2 | 2.54E-11 | 0.282063938 | 0.321 | 0.133 | 7.90E-07 | Proliferating | SUN2 |
| CD2761 | 2.54E-11 | 0.257162043 | 0.321 | 0.133 | 7.91E-07 | Proliferating | CD276 |
| MRPS21 | 2.62E-11 | 0.311595325 | 0.648 | 0.36 | 8.16E-07 | Proliferating | MRPS21 |
| PTPN18 | 2.64E-11 | 0.305214822 | 0.394 | 0.179 | 8.21E-07 | Proliferating | PTPN18 |
| WSB2 | 3.01E-11 | 0.260842516 | 0.327 | 0.135 | 9.38E-07 | Proliferating | WSB2 |
| MAGOH | 3.03E-11 | 0.293071763 | 0.564 | 0.293 | 9.41E-07 | Proliferating | MAGOH |
| CRELD2 | 3.05E-11 | 0.273929549 | 0.448 | 0.211 | 9.50E-07 | Proliferating | CRELD2 |
| PSMB61 | 3.06E-11 | 0.419008136 | 0.752 | 0.483 | 9.53E-07 | Proliferating | PSMB6 |
| NDUFA6 | 3.34E-11 | 0.369222752 | 0.703 | 0.398 | 1.04E-06 | Proliferating | NDUFA6 |
| SRSF71 | 3.68E-11 | 0.324450214 | 0.661 | 0.353 | 1.15E-06 | Proliferating | SRSF7 |
| GTF2H51 | 3.91E-11 | 0.275139125 | 0.594 | 0.308 | 1.22E-06 | Proliferating | GTF2H5 |
| SELPLG | 3.93E-11 | 0.26226152 | 0.394 | 0.174 | 1.22E-06 | Proliferating | SELPLG |
| BTF3L4 | 3.94E-11 | 0.399238365 | 0.576 | 0.304 | 1.23E-06 | Proliferating | BTF3L4 |
| TCEA1 | 4.07E-11 | 0.257843051 | 0.509 | 0.248 | 1.27E-06 | Proliferating | TCEA1 |
| PPP2R3C | 4.32E-11 | 0.341168669 | 0.394 | 0.175 | 1.34E-06 | Proliferating | PPP2R3C |
| KHDRBS1 | 4.57E-11 | 0.375537567 | 0.642 | 0.363 | 1.42E-06 | Proliferating | KHDRBS1 |
| PFN11 | 4.97E-11 | 0.347191182 | 1 | 0.969 | 1.55E-06 | Proliferating | PFN1 |
| GNAS1 | 5.04E-11 | 0.363280855 | 0.806 | 0.525 | 1.57E-06 | Proliferating | GNAS |
| CTSB2 | 5.35E-11 | 0.449654411 | 0.994 | 0.918 | 1.67E-06 | Proliferating | CTSB |
| NDUFB6 | 5.53E-11 | 0.287892128 | 0.576 | 0.305 | 1.72E-06 | Proliferating | NDUFB6 |
| SNRNP70 | 5.55E-11 | 0.307642115 | 0.497 | 0.249 | 1.73E-06 | Proliferating | SNRNP70 |
| PIGT1 | 5.94E-11 | 0.26705515 | 0.406 | 0.187 | 1.85E-06 | Proliferating | PIGT |
| CCT4 | 5.98E-11 | 0.345643603 | 0.63 | 0.347 | 1.86E-06 | Proliferating | CCT4 |
| ANAPC15 | 6.14E-11 | 0.254741337 | 0.37 | 0.163 | 1.91E-06 | Proliferating | ANAPC15 |
| PSMB2 | 6.55E-11 | 0.383452933 | 0.679 | 0.399 | 2.04E-06 | Proliferating | PSMB2 |
| OLA1 | 7.08E-11 | 0.265992744 | 0.455 | 0.223 | 2.20E-06 | Proliferating | OLA1 |
| ALG5 | 7.52E-11 | 0.25361344 | 0.467 | 0.228 | 2.34E-06 | Proliferating | ALG5 |
| MYEOV2 | 7.53E-11 | 0.407807784 | 0.739 | 0.451 | 2.34E-06 | Proliferating | MYEOV2 |
| HNRNPF | 7.62E-11 | 0.317846292 | 0.77 | 0.463 | 2.37E-06 | Proliferating | HNRNPF |
| IFI27L21 | 7.66E-11 | 0.348827616 | 0.812 | 0.479 | 2.38E-06 | Proliferating | IFI27L2 |
| SSR3 | 7.72E-11 | 0.345615894 | 0.83 | 0.517 | 2.40E-06 | Proliferating | SSR3 |
| PSMA21 | 7.88E-11 | 0.362909472 | 0.721 | 0.423 | 2.45E-06 | Proliferating | PSMA2 |
| FAM26F1 | 8.18E-11 | 0.301565771 | 0.752 | 0.455 | 2.55E-06 | Proliferating | FAM26F |
| NTMT1 | 8.24E-11 | 0.250910053 | 0.364 | 0.164 | 2.57E-06 | Proliferating | NTMT1 |
| NEDD8 | 8.33E-11 | 0.404175534 | 0.909 | 0.663 | 2.59E-06 | Proliferating | NEDD8 |
| CCT81 | 8.85E-11 | 0.329638293 | 0.606 | 0.337 | 2.75E-06 | Proliferating | CCT8 |
| APMAP1 | 9.13E-11 | 0.255785437 | 0.448 | 0.216 | 2.84E-06 | Proliferating | APMAP |
| ACOT131 | 9.37E-11 | 0.311695386 | 0.345 | 0.155 | 2.91E-06 | Proliferating | ACOT13 |
| RBM8A | 1.03E-10 | 0.307385564 | 0.8 | 0.478 | 3.20E-06 | Proliferating | RBM8A |
| GNPDA12 | 1.04E-10 | 0.265663448 | 0.4 | 0.183 | 3.25E-06 | Proliferating | GNPDA1 |
| LAMTOR22 | 1.12E-10 | 0.353070705 | 0.83 | 0.538 | 3.50E-06 | Proliferating | LAMTOR2 |
| NDUFB1 | 1.14E-10 | 0.427737009 | 0.836 | 0.57 | 3.53E-06 | Proliferating | NDUFB1 |
| MRPS18C | 1.19E-10 | 0.252925638 | 0.418 | 0.2 | 3.71E-06 | Proliferating | MRPS18C |
| UNC93B11 | 1.20E-10 | 0.252249669 | 0.485 | 0.237 | 3.72E-06 | Proliferating | UNC93B1 |
| CTDNEP11 | 1.35E-10 | 0.29450708 | 0.467 | 0.234 | 4.19E-06 | Proliferating | CTDNEP1 |
| MS4A4A2 | 1.35E-10 | 0.503157349 | 0.709 | 0.429 | 4.19E-06 | Proliferating | MS4A4A |
| BMP2K2 | 1.47E-10 | 0.271604822 | 0.43 | 0.207 | 4.58E-06 | Proliferating | BMP2K |
| P4HB1 | 1.51E-10 | 0.414297999 | 0.861 | 0.596 | 4.70E-06 | Proliferating | P4HB |
| IFI161 | 1.61E-10 | 0.28654572 | 0.564 | 0.296 | 5.00E-06 | Proliferating | IFI16 |
| AAMP | 1.62E-10 | 0.262228266 | 0.382 | 0.177 | 5.03E-06 | Proliferating | AAMP |
| PSMD13 | 1.70E-10 | 0.294487956 | 0.497 | 0.26 | 5.28E-06 | Proliferating | PSMD13 |
| POLR2E1 | 1.93E-10 | 0.377902737 | 0.655 | 0.371 | 6.01E-06 | Proliferating | POLR2E |
| PFDN2 | 2.09E-10 | 0.304242975 | 0.691 | 0.399 | 6.52E-06 | Proliferating | PFDN2 |
| SAMHD12 | 2.12E-10 | 0.363498994 | 0.788 | 0.485 | 6.61E-06 | Proliferating | SAMHD1 |
| APEX11 | 2.17E-10 | 0.284356305 | 0.473 | 0.243 | 6.77E-06 | Proliferating | APEX1 |
| POLR3GL | 2.40E-10 | 0.251972031 | 0.388 | 0.182 | 7.47E-06 | Proliferating | POLR3GL |
| SLC25A31 | 2.41E-10 | 0.43364121 | 0.879 | 0.659 | 7.51E-06 | Proliferating | SLC25A3 |
| PLP2 | 2.65E-10 | 0.344415723 | 0.703 | 0.413 | 8.25E-06 | Proliferating | PLP2 |
| PPP1CA | 2.65E-10 | 0.393009516 | 0.824 | 0.54 | 8.26E-06 | Proliferating | PPP1CA |
| SMIM71 | 2.65E-10 | 0.279276034 | 0.442 | 0.221 | 8.26E-06 | Proliferating | SMIM7 |
| SCAMP21 | 2.69E-10 | 0.294872131 | 0.479 | 0.243 | 8.37E-06 | Proliferating | SCAMP2 |
| C11orf73 | 2.71E-10 | 0.300242921 | 0.461 | 0.232 | 8.42E-06 | Proliferating | C11orf73 |
| RTN31 | 2.72E-10 | 0.327861888 | 0.606 | 0.333 | 8.46E-06 | Proliferating | RTN3 |
| NAGA1 | 2.72E-10 | 0.276647989 | 0.479 | 0.239 | 8.47E-06 | Proliferating | NAGA |
| KIAA09301 | 3.03E-10 | 0.25064716 | 0.406 | 0.19 | 9.42E-06 | Proliferating | KIAA0930 |
| ARPC21 | 3.42E-10 | 0.355485089 | 0.982 | 0.878 | 1.06E-05 | Proliferating | ARPC2 |
| RNASE61 | 4.42E-10 | 0.329447829 | 0.745 | 0.446 | 1.37E-05 | Proliferating | RNASE6 |
| NDUFS6 | 4.56E-10 | 0.297997793 | 0.842 | 0.527 | 1.42E-05 | Proliferating | NDUFS6 |
| DAPK11 | 4.76E-10 | 0.252227724 | 0.418 | 0.202 | 1.48E-05 | Proliferating | DAPK1 |
| EEF1B21 | 5.29E-10 | 0.447742365 | 0.939 | 0.771 | 1.65E-05 | Proliferating | EEF1B2 |
| LMAN21 | 5.31E-10 | 0.288542322 | 0.752 | 0.43 | 1.65E-05 | Proliferating | LMAN2 |
| HSBP1 | 5.61E-10 | 0.324420537 | 0.903 | 0.59 | 1.75E-05 | Proliferating | HSBP1 |
| TMEM1472 | 5.94E-10 | 0.321076557 | 0.745 | 0.443 | 1.85E-05 | Proliferating | TMEM147 |
| COMMD91 | 5.96E-10 | 0.271068303 | 0.424 | 0.211 | 1.85E-05 | Proliferating | COMMD9 |
| CTSD2 | 6.10E-10 | 0.405160772 | 0.964 | 0.787 | 1.90E-05 | Proliferating | CTSD |
| SYPL1 | 6.26E-10 | 0.278336885 | 0.473 | 0.242 | 1.95E-05 | Proliferating | SYPL1 |
| PSMA51 | 6.30E-10 | 0.277728675 | 0.594 | 0.324 | 1.96E-05 | Proliferating | PSMA5 |
| PHB1 | 7.12E-10 | 0.273399416 | 0.539 | 0.288 | 2.22E-05 | Proliferating | PHB |
| CTSC2 | 7.28E-10 | 0.297610666 | 0.921 | 0.656 | 2.26E-05 | Proliferating | CTSC |
| MTDH2 | 7.31E-10 | 0.413394416 | 0.788 | 0.537 | 2.27E-05 | Proliferating | MTDH |
| LAIR12 | 7.43E-10 | 0.287383766 | 0.721 | 0.419 | 2.31E-05 | Proliferating | LAIR1 |
| C19orf53 | 7.99E-10 | 0.346550333 | 0.812 | 0.562 | 2.49E-05 | Proliferating | C19orf53 |
| ISOC21 | 8.01E-10 | 0.270530188 | 0.406 | 0.199 | 2.49E-05 | Proliferating | ISOC2 |
| MRPL41 | 8.57E-10 | 0.392161566 | 0.667 | 0.408 | 2.67E-05 | Proliferating | MRPL41 |
| CD592 | 8.79E-10 | 0.315775348 | 0.739 | 0.449 | 2.73E-05 | Proliferating | CD59 |
| GDI21 | 9.39E-10 | 0.384936717 | 0.77 | 0.505 | 2.92E-05 | Proliferating | GDI2 |
| GADD45GIP1 | 9.71E-10 | 0.286948929 | 0.745 | 0.453 | 3.02E-05 | Proliferating | GADD45GIP1 |
| PSMG2 | 9.78E-10 | 0.337282957 | 0.497 | 0.264 | 3.04E-05 | Proliferating | PSMG2 |
| SLIRP | 9.83E-10 | 0.298581629 | 0.685 | 0.408 | 3.06E-05 | Proliferating | SLIRP |
| WDR61 | 1.00E-09 | 0.261242104 | 0.327 | 0.146 | 3.11E-05 | Proliferating | WDR61 |
| CALR1 | 1.04E-09 | 0.385397452 | 0.903 | 0.718 | 3.25E-05 | Proliferating | CALR |
| NAP1L12 | 1.08E-09 | 0.333847223 | 0.885 | 0.653 | 3.37E-05 | Proliferating | NAP1L1 |
| JTB | 1.18E-09 | 0.336836529 | 0.812 | 0.528 | 3.66E-05 | Proliferating | JTB |
| ARPP19 | 1.18E-09 | 0.306718928 | 0.497 | 0.259 | 3.67E-05 | Proliferating | ARPP19 |
| CNIH4 | 1.18E-09 | 0.301737734 | 0.582 | 0.312 | 3.68E-05 | Proliferating | CNIH4 |
| DBNL2 | 1.22E-09 | 0.338705515 | 0.673 | 0.377 | 3.81E-05 | Proliferating | DBNL |
| CSF1R2 | 1.31E-09 | 0.411572134 | 0.709 | 0.428 | 4.06E-05 | Proliferating | CSF1R |
| KIF5B | 1.39E-09 | 0.283444674 | 0.691 | 0.406 | 4.33E-05 | Proliferating | KIF5B |
| DYNLL11 | 1.39E-09 | 0.400513096 | 0.927 | 0.738 | 4.34E-05 | Proliferating | DYNLL1 |
| DCTN3 | 1.42E-09 | 0.304979495 | 0.485 | 0.257 | 4.41E-05 | Proliferating | DCTN3 |
| PEA151 | 1.53E-09 | 0.337689566 | 0.721 | 0.417 | 4.75E-05 | Proliferating | PEA15 |
| MRPL20 | 1.55E-09 | 0.375957827 | 0.685 | 0.417 | 4.81E-05 | Proliferating | MRPL20 |
| BLOC1S1 | 1.55E-09 | 0.353905534 | 0.891 | 0.649 | 4.84E-05 | Proliferating | BLOC1S1 |
| SOD11 | 1.60E-09 | 0.343716782 | 0.842 | 0.554 | 4.97E-05 | Proliferating | SOD1 |
| NQO21 | 1.66E-09 | 0.293738409 | 0.527 | 0.287 | 5.18E-05 | Proliferating | NQO2 |
| MT-CYB1 | 1.79E-09 | 0.369293359 | 0.994 | 0.987 | 5.56E-05 | Proliferating | MT-CYB |
| LINC01420 | 1.83E-09 | 0.332485664 | 0.6 | 0.338 | 5.70E-05 | Proliferating | LINC01420 |
| CNDP21 | 1.88E-09 | 0.255575534 | 0.6 | 0.329 | 5.84E-05 | Proliferating | CNDP2 |
| RER1 | 1.96E-09 | 0.400940116 | 0.642 | 0.407 | 6.10E-05 | Proliferating | RER1 |
| C11orf31 | 2.04E-09 | 0.317417121 | 0.848 | 0.553 | 6.36E-05 | Proliferating | C11orf31 |
| MT-ATP61 | 2.18E-09 | 0.3231042 | 1 | 0.993 | 6.77E-05 | Proliferating | MT-ATP6 |
| C19orf601 | 2.29E-09 | 0.308780453 | 0.764 | 0.463 | 7.13E-05 | Proliferating | C19orf60 |
| SGPL11 | 2.42E-09 | 0.268699171 | 0.515 | 0.272 | 7.53E-05 | Proliferating | SGPL1 |
| SNU13 | 2.45E-09 | 0.335528936 | 0.715 | 0.449 | 7.62E-05 | Proliferating | SNU13 |
| CCDC109B | 2.49E-09 | 0.269019343 | 0.594 | 0.325 | 7.74E-05 | Proliferating | CCDC109B |
| AES | 2.51E-09 | 0.25194773 | 0.461 | 0.236 | 7.80E-05 | Proliferating | AES |
| YIF1B | 2.51E-09 | 0.251819711 | 0.436 | 0.225 | 7.82E-05 | Proliferating | YIF1B |
| UBE2I | 2.53E-09 | 0.273240229 | 0.642 | 0.377 | 7.87E-05 | Proliferating | UBE2I |
| NDUFS71 | 2.60E-09 | 0.328322466 | 0.745 | 0.481 | 8.07E-05 | Proliferating | NDUFS7 |
| CD812 | 2.72E-09 | 0.355218085 | 0.794 | 0.494 | 8.47E-05 | Proliferating | CD81 |
| SLC16A101 | 2.84E-09 | 0.276457576 | 0.618 | 0.352 | 8.85E-05 | Proliferating | SLC16A10 |
| FABP51 | 2.90E-09 | 0.28641973 | 0.891 | 0.585 | 9.02E-05 | Proliferating | FABP5 |
| DUSP22 | 2.98E-09 | 0.389718386 | 0.782 | 0.514 | 9.28E-05 | Proliferating | DUSP2 |
| PDIA61 | 3.31E-09 | 0.373131997 | 0.788 | 0.53 | 0.000102869 | Proliferating | PDIA6 |
| WASF21 | 3.37E-09 | 0.25165989 | 0.545 | 0.299 | 0.000104731 | Proliferating | WASF2 |
| ASNA1 | 3.60E-09 | 0.278766039 | 0.485 | 0.258 | 0.000112009 | Proliferating | ASNA1 |
| MRFAP1 | 3.63E-09 | 0.302037324 | 0.679 | 0.405 | 0.000112999 | Proliferating | MRFAP1 |
| DNAJC8 | 3.86E-09 | 0.289761239 | 0.594 | 0.34 | 0.000119996 | Proliferating | DNAJC8 |
| NDUFB3 | 3.95E-09 | 0.306539527 | 0.721 | 0.438 | 0.000122913 | Proliferating | NDUFB3 |
| PTTG1IP | 3.96E-09 | 0.274647116 | 0.612 | 0.348 | 0.000123176 | Proliferating | PTTG1IP |
| ATP5H | 4.24E-09 | 0.355748871 | 0.824 | 0.557 | 0.000131945 | Proliferating | ATP5H |
| CCL81 | 4.32E-09 | 0.526393017 | 0.212 | 0.08 | 0.000134273 | Proliferating | CCL8 |
| ATP5C11 | 4.42E-09 | 0.323046856 | 0.782 | 0.504 | 0.000137572 | Proliferating | ATP5C1 |
| USF2 | 4.46E-09 | 0.253342222 | 0.442 | 0.226 | 0.00013865 | Proliferating | USF2 |
| SPCS3 | 4.69E-09 | 0.279048744 | 0.782 | 0.498 | 0.000145819 | Proliferating | SPCS3 |
| CLPTM1 | 4.72E-09 | 0.269657544 | 0.345 | 0.163 | 0.000146889 | Proliferating | CLPTM1 |
| PSMD8 | 5.87E-09 | 0.312888776 | 0.697 | 0.443 | 0.000182646 | Proliferating | PSMD8 |
| LINC009982 | 5.87E-09 | 0.274507515 | 0.648 | 0.376 | 0.000182705 | Proliferating | LINC00998 |
| PSMA4 | 7.23E-09 | 0.382352966 | 0.794 | 0.511 | 0.000224845 | Proliferating | PSMA4 |
| SF3B5 | 7.96E-09 | 0.302470832 | 0.818 | 0.539 | 0.000247588 | Proliferating | SF3B5 |
| SNRPD2 | 8.32E-09 | 0.346195305 | 0.861 | 0.602 | 0.000258945 | Proliferating | SNRPD2 |
| MTCH1 | 8.82E-09 | 0.255438867 | 0.606 | 0.352 | 0.000274529 | Proliferating | MTCH1 |
| M6PR2 | 8.92E-09 | 0.312090111 | 0.739 | 0.487 | 0.000277568 | Proliferating | M6PR |
| ATP5B2 | 8.98E-09 | 0.328069966 | 0.848 | 0.573 | 0.000279575 | Proliferating | ATP5B |
| PSMA7 | 9.34E-09 | 0.342975218 | 0.933 | 0.76 | 0.000290593 | Proliferating | PSMA7 |
| SEPW12 | 9.89E-09 | 0.301011849 | 0.691 | 0.409 | 0.000307636 | Proliferating | SEPW1 |
| HSD17B11 | 1.10E-08 | 0.300812079 | 0.6 | 0.342 | 0.000340795 | Proliferating | HSD17B11 |
| ADAP22 | 1.12E-08 | 0.290529218 | 0.461 | 0.252 | 0.00034884 | Proliferating | ADAP2 |
| WDR18 | 1.42E-08 | 0.266633615 | 0.248 | 0.107 | 0.000441373 | Proliferating | WDR18 |
| SDHC1 | 1.44E-08 | 0.265682096 | 0.57 | 0.324 | 0.00044702 | Proliferating | SDHC |
| CLTB1 | 1.54E-08 | 0.254278936 | 0.624 | 0.363 | 0.000479996 | Proliferating | CLTB |
| GSDMD | 1.62E-08 | 0.326351664 | 0.448 | 0.242 | 0.000504211 | Proliferating | GSDMD |
| NUDC | 1.77E-08 | 0.284144521 | 0.521 | 0.298 | 0.000549642 | Proliferating | NUDC |
| SEC11A | 1.78E-08 | 0.330255572 | 0.8 | 0.549 | 0.000553575 | Proliferating | SEC11A |
| PSMA3 | 1.79E-08 | 0.28556716 | 0.57 | 0.329 | 0.000557295 | Proliferating | PSMA3 |
| COX7A2 | 1.84E-08 | 0.33467527 | 0.97 | 0.83 | 0.000571331 | Proliferating | COX7A2 |
| HMGA11 | 1.95E-08 | 0.317432008 | 0.655 | 0.407 | 0.000605625 | Proliferating | HMGA1 |
| NDUFB51 | 1.96E-08 | 0.252239104 | 0.715 | 0.425 | 0.00060842 | Proliferating | NDUFB5 |
| ACADVL1 | 2.10E-08 | 0.250897736 | 0.624 | 0.365 | 0.000654773 | Proliferating | ACADVL |
| H2AFJ1 | 2.13E-08 | 0.348957342 | 0.758 | 0.476 | 0.000661692 | Proliferating | H2AFJ |
| RPL351 | 2.14E-08 | 0.316137053 | 1 | 0.965 | 0.000666985 | Proliferating | RPL35 |
| TPM31 | 2.22E-08 | 0.353896266 | 0.927 | 0.75 | 0.000691588 | Proliferating | TPM3 |
| TMX2 | 2.41E-08 | 0.253237623 | 0.345 | 0.169 | 0.000748384 | Proliferating | TMX2 |
| SDSL1 | 2.47E-08 | 0.260763346 | 0.479 | 0.26 | 0.00076926 | Proliferating | SDSL |
| NDUFB81 | 2.52E-08 | 0.289996038 | 0.848 | 0.547 | 0.000782683 | Proliferating | NDUFB8 |
| VPS35 | 2.98E-08 | 0.277581928 | 0.558 | 0.327 | 0.000926213 | Proliferating | VPS35 |
| CAMTA1 | 3.08E-08 | 0.281583796 | 0.624 | 0.391 | 0.000957591 | Proliferating | CAMTA1 |
| SLC25A44 | 3.14E-08 | 0.306090604 | 0.212 | 0.087 | 0.000978555 | Proliferating | SLC25A44 |
| HEXA2 | 3.17E-08 | 0.345366377 | 0.606 | 0.356 | 0.000986253 | Proliferating | HEXA |
| ATP5G11 | 3.45E-08 | 0.270005009 | 0.618 | 0.373 | 0.001074378 | Proliferating | ATP5G1 |
| LAP31 | 3.62E-08 | 0.263032016 | 0.758 | 0.453 | 0.001125893 | Proliferating | LAP3 |
| SPG211 | 3.87E-08 | 0.256675244 | 0.758 | 0.469 | 0.001203229 | Proliferating | SPG21 |
| NDUFA2 | 4.27E-08 | 0.294274767 | 0.788 | 0.51 | 0.00132942 | Proliferating | NDUFA2 |
| ITM2C1 | 4.35E-08 | 0.264556936 | 0.23 | 0.095 | 0.001352607 | Proliferating | ITM2C |
| DBI1 | 4.54E-08 | 0.361809041 | 0.945 | 0.771 | 0.001411114 | Proliferating | DBI |
| PCBP2 | 4.67E-08 | 0.294851474 | 0.867 | 0.614 | 0.001454141 | Proliferating | PCBP2 |
| NDUFA122 | 4.69E-08 | 0.274906539 | 0.606 | 0.364 | 0.001458595 | Proliferating | NDUFA12 |
| ARHGDIA | 5.03E-08 | 0.272032934 | 0.794 | 0.519 | 0.001564526 | Proliferating | ARHGDIA |
| FOLR22 | 5.42E-08 | 0.319305807 | 0.339 | 0.167 | 0.001685897 | Proliferating | FOLR2 |
| ATP2C11 | 5.68E-08 | 0.250837459 | 0.473 | 0.262 | 0.001766035 | Proliferating | ATP2C1 |
| TMEM230 | 6.60E-08 | 0.274743294 | 0.679 | 0.418 | 0.002052277 | Proliferating | TMEM230 |
| NDUFB21 | 6.96E-08 | 0.33048657 | 0.891 | 0.681 | 0.002165948 | Proliferating | NDUFB2 |
| UBE2L3 | 7.22E-08 | 0.286444781 | 0.739 | 0.488 | 0.002245366 | Proliferating | UBE2L3 |
| EMC7 | 7.22E-08 | 0.281384743 | 0.515 | 0.305 | 0.002248081 | Proliferating | EMC7 |
| FAM49B1 | 9.33E-08 | 0.357789886 | 0.903 | 0.636 | 0.002901767 | Proliferating | FAM49B |
| GLIPR11 | 9.38E-08 | 0.278886722 | 0.679 | 0.446 | 0.002918579 | Proliferating | GLIPR1 |
| PTGS11 | 1.07E-07 | 0.259410975 | 0.376 | 0.195 | 0.003314353 | Proliferating | PTGS1 |
| FCGRT2 | 1.10E-07 | 0.342688368 | 0.915 | 0.699 | 0.003426125 | Proliferating | FCGRT |
| LGALS91 | 1.16E-07 | 0.259591378 | 0.721 | 0.442 | 0.00360867 | Proliferating | LGALS9 |
| EIF5A1 | 1.61E-07 | 0.380352675 | 0.636 | 0.427 | 0.005011988 | Proliferating | EIF5A |
| RPL22L1 | 1.65E-07 | 0.315575521 | 0.661 | 0.429 | 0.005123291 | Proliferating | RPL22L1 |
| PRDM1 | 1.69E-07 | 0.265186315 | 0.491 | 0.276 | 0.00526581 | Proliferating | PRDM1 |
| CCL51 | 1.77E-07 | 0.29408985 | 0.412 | 0.221 | 0.005509371 | Proliferating | CCL5 |
| LAPTM4A2 | 1.92E-07 | 0.279375761 | 0.891 | 0.592 | 0.00595896 | Proliferating | LAPTM4A |
| HAMP1 | 1.96E-07 | 0.317076013 | 0.158 | 0.058 | 0.006101964 | Proliferating | HAMP |
| EFHD2 | 2.75E-07 | 0.292324508 | 0.77 | 0.504 | 0.008542166 | Proliferating | EFHD2 |
| GNB2 | 2.84E-07 | 0.284754613 | 0.745 | 0.487 | 0.008830413 | Proliferating | GNB2 |
| BST21 | 2.84E-07 | 0.313027896 | 0.842 | 0.57 | 0.008832688 | Proliferating | BST2 |
| LAMTOR11 | 3.33E-07 | 0.278937483 | 0.764 | 0.511 | 0.010356965 | Proliferating | LAMTOR1 |
| ZNF3311 | 3.36E-07 | 0.275024889 | 0.515 | 0.314 | 0.010446421 | Proliferating | ZNF331 |
| FUS | 3.41E-07 | 0.265133957 | 0.776 | 0.514 | 0.010603123 | Proliferating | FUS |
| TMED2 | 3.67E-07 | 0.277641507 | 0.782 | 0.504 | 0.011418841 | Proliferating | TMED2 |
| TAGLN21 | 3.94E-07 | 0.257678688 | 0.891 | 0.699 | 0.012269955 | Proliferating | TAGLN2 |
| SAP18 | 4.20E-07 | 0.251556885 | 0.873 | 0.625 | 0.013056635 | Proliferating | SAP18 |
| RGS102 | 4.23E-07 | 0.280650445 | 0.897 | 0.647 | 0.013156603 | Proliferating | RGS10 |
| HNRNPH1 | 4.35E-07 | 0.350469585 | 0.642 | 0.413 | 0.013534279 | Proliferating | HNRNPH1 |
| ITGB23 | 4.65E-07 | 0.256626446 | 0.921 | 0.696 | 0.014471787 | Proliferating | ITGB2 |
| PSMB1 | 4.79E-07 | 0.293097386 | 0.812 | 0.581 | 0.014897431 | Proliferating | PSMB1 |
| SPCS1 | 5.33E-07 | 0.289288124 | 0.758 | 0.526 | 0.016597796 | Proliferating | SPCS1 |
| RPS51 | 6.01E-07 | 0.260465287 | 0.994 | 0.924 | 0.018708881 | Proliferating | RPS5 |
| PYCARD1 | 6.23E-07 | 0.277963003 | 0.842 | 0.588 | 0.019391819 | Proliferating | PYCARD |
| NR4A1 | 6.48E-07 | 0.250726012 | 0.661 | 0.415 | 0.020169115 | Proliferating | NR4A1 |
| TRAPPC1 | 6.49E-07 | 0.255633715 | 0.794 | 0.536 | 0.020209247 | Proliferating | TRAPPC1 |
| MRPL23 | 6.63E-07 | 0.261424778 | 0.57 | 0.354 | 0.020620203 | Proliferating | MRPL23 |
| ATP5I1 | 9.38E-07 | 0.289377578 | 0.897 | 0.646 | 0.029187856 | Proliferating | ATP5I |
| COX6A1 | 1.12E-06 | 0.298399338 | 0.915 | 0.763 | 0.034804442 | Proliferating | COX6A1 |
| NDUFS5 | 1.21E-06 | 0.317245929 | 0.855 | 0.663 | 0.037656783 | Proliferating | NDUFS5 |

Table S5. Characteristic genes of Endothelial cell subtypes.

|  | p_val | avg_log2FC | pct.1 | pct.2 | p_val_adj | cluster | gene |
| --- | --- | --- | --- | --- | --- | --- | --- |
| ACKR1 | 1.13E-147 | 3.517687274 | 0.799 | 0.066 | 3.50E-143 | Vein | ACKR1 |
| SELP | 6.96E-109 | 1.799983858 | 0.627 | 0.028 | 2.17E-104 | Vein | SELP |
| SELE | 1.24E-78 | 2.598580629 | 0.607 | 0.112 | 3.86E-74 | Vein | SELE |
| POSTN | 2.70E-56 | 2.158995038 | 0.462 | 0.074 | 8.39E-52 | Vein | POSTN |
| PRCP | 5.49E-55 | 1.107134155 | 0.833 | 0.504 | 1.71E-50 | Vein | PRCP |
| DUSP23 | 5.52E-52 | 1.214781252 | 0.742 | 0.433 | 1.72E-47 | Vein | DUSP23 |
| ADIRF | 5.22E-51 | 1.578054339 | 0.662 | 0.293 | 1.62E-46 | Vein | ADIRF |
| RPL32 | 3.20E-46 | 0.499814542 | 1 | 0.991 | 9.96E-42 | Vein | RPL32 |
| IL1R1 | 2.46E-44 | 0.835595375 | 0.344 | 0.036 | 7.67E-40 | Vein | IL1R1 |
| CD74 | 1.07E-42 | 1.099426422 | 0.898 | 0.657 | 3.32E-38 | Vein | CD74 |
| RPL12 | 1.56E-42 | 0.544699092 | 0.992 | 0.972 | 4.84E-38 | Vein | RPL12 |
| ZNF385D | 2.72E-42 | 0.900427425 | 0.552 | 0.197 | 8.47E-38 | Vein | ZNF385D |
| NPC2 | 4.02E-42 | 1.20928423 | 0.828 | 0.647 | 1.25E-37 | Vein | NPC2 |
| NCOA7 | 8.45E-42 | 1.270726066 | 0.681 | 0.389 | 2.63E-37 | Vein | NCOA7 |
| SLCO2A1 | 1.84E-41 | 0.86155922 | 0.711 | 0.326 | 5.72E-37 | Vein | SLCO2A1 |
| ZFP36L1 | 2.14E-38 | 0.987518331 | 0.826 | 0.625 | 6.66E-34 | Vein | ZFP36L1 |
| RPL34 | 4.98E-38 | 0.500025684 | 0.995 | 0.995 | 1.55E-33 | Vein | RPL34 |
| NR2F2 | 6.36E-38 | 0.947905224 | 0.552 | 0.216 | 1.98E-33 | Vein | NR2F2 |
| RPS19 | 6.47E-38 | 0.447592169 | 0.997 | 0.994 | 2.01E-33 | Vein | RPS19 |
| RAB3C | 9.41E-38 | 0.627269604 | 0.236 | 0.002 | 2.93E-33 | Vein | RAB3C |
| RPS14 | 1.13E-37 | 0.446124408 | 0.997 | 0.98 | 3.52E-33 | Vein | RPS14 |
| RPS15A | 2.44E-37 | 0.459273774 | 0.987 | 0.964 | 7.59E-33 | Vein | RPS15A |
| RPS18 | 3.55E-37 | 0.468564142 | 1 | 0.991 | 1.10E-32 | Vein | RPS18 |
| NNMT | 1.03E-36 | 0.755272022 | 0.816 | 0.521 | 3.21E-32 | Vein | NNMT |
| CST3 | 1.78E-36 | 0.766611114 | 0.938 | 0.835 | 5.54E-32 | Vein | CST3 |
| RAMP3 | 2.88E-36 | 0.741488932 | 0.893 | 0.614 | 8.96E-32 | Vein | RAMP3 |
| TPT1 | 5.89E-36 | 0.489349473 | 0.997 | 0.978 | 1.83E-31 | Vein | TPT1 |
| HLA-DRA | 7.83E-35 | 1.187454603 | 0.717 | 0.447 | 2.44E-30 | Vein | HLA-DRA |
| HLA-DPB1 | 2.08E-33 | 0.973232884 | 0.651 | 0.38 | 6.46E-29 | Vein | HLA-DPB1 |
| RPS23 | 3.52E-33 | 0.445508693 | 0.988 | 0.989 | 1.10E-28 | Vein | RPS23 |
| RPLP1 | 5.53E-33 | 0.360538908 | 1 | 0.994 | 1.72E-28 | Vein | RPLP1 |
| MEOX1 | 5.67E-33 | 0.591218626 | 0.386 | 0.098 | 1.76E-28 | Vein | MEOX1 |
| RPL13 | 2.08E-32 | 0.402572981 | 0.998 | 0.995 | 6.46E-28 | Vein | RPL13 |
| VCAM1 | 1.12E-31 | 1.017608612 | 0.386 | 0.109 | 3.48E-27 | Vein | VCAM1 |
| RPS27A | 2.57E-31 | 0.386430753 | 0.997 | 0.989 | 7.99E-27 | Vein | RPS27A |
| PTGDS | 1.38E-30 | 1.103735688 | 0.294 | 0.052 | 4.29E-26 | Vein | PTGDS |
| HLA-DRB5 | 2.97E-30 | 0.915402631 | 0.395 | 0.12 | 9.23E-26 | Vein | HLA-DRB5 |
| RBP5 | 3.00E-30 | 0.650304358 | 0.413 | 0.128 | 9.35E-26 | Vein | RBP5 |
| ARL4A | 3.80E-30 | 0.732794077 | 0.783 | 0.542 | 1.18E-25 | Vein | ARL4A |
| SDCBP | 6.99E-30 | 0.643729481 | 0.801 | 0.602 | 2.18E-25 | Vein | SDCBP |
| HLA-DPA1 | 7.08E-30 | 0.984979463 | 0.634 | 0.365 | 2.20E-25 | Vein | HLA-DPA1 |
| RPS6 | 3.09E-29 | 0.458422305 | 0.998 | 0.986 | 9.60E-25 | Vein | RPS6 |
| RPL13A | 6.82E-29 | 0.360877298 | 0.993 | 0.995 | 2.12E-24 | Vein | RPL13A |
| RPS12 | 8.68E-29 | 0.398349296 | 0.997 | 0.984 | 2.70E-24 | Vein | RPS12 |
| RPL10 | 3.35E-28 | 0.396998698 | 0.997 | 0.991 | 1.04E-23 | Vein | RPL10 |
| HAPLN3 | 3.52E-28 | 0.668784328 | 0.475 | 0.195 | 1.09E-23 | Vein | HAPLN3 |
| RPS9 | 3.68E-28 | 0.394463066 | 0.993 | 0.973 | 1.15E-23 | Vein | RPS9 |
| RPL39 | 5.38E-28 | 0.448692169 | 0.993 | 0.978 | 1.68E-23 | Vein | RPL39 |
| TSPAN7 | 6.39E-28 | 0.880504652 | 0.542 | 0.269 | 1.99E-23 | Vein | TSPAN7 |
| PTAFR | 9.17E-28 | 0.454599797 | 0.196 | 0.009 | 2.85E-23 | Vein | PTAFR |
| RPS15 | 1.73E-27 | 0.332882534 | 0.998 | 0.994 | 5.39E-23 | Vein | RPS15 |
| NFKBIZ | 2.01E-27 | 0.833494646 | 0.607 | 0.34 | 6.25E-23 | Vein | NFKBIZ |
| MATN2 | 8.61E-27 | 0.601604473 | 0.426 | 0.153 | 2.68E-22 | Vein | MATN2 |
| OLFM1 | 2.18E-26 | 0.586564231 | 0.326 | 0.091 | 6.78E-22 | Vein | OLFM1 |
| CCDC69 | 4.26E-26 | 0.582726789 | 0.51 | 0.241 | 1.32E-21 | Vein | CCDC69 |
| FOXP1 | 1.91E-25 | 0.807966208 | 0.661 | 0.413 | 5.93E-21 | Vein | FOXP1 |
| CYP1B1 | 2.02E-25 | 0.74102813 | 0.264 | 0.05 | 6.29E-21 | Vein | CYP1B1 |
| RPS8 | 4.87E-25 | 0.408682335 | 0.988 | 0.987 | 1.52E-20 | Vein | RPS8 |
| RPS3A | 4.97E-25 | 0.426264541 | 0.988 | 0.975 | 1.55E-20 | Vein | RPS3A |
| IL33 | 5.81E-25 | 0.620702999 | 0.413 | 0.142 | 1.81E-20 | Vein | IL33 |
| RPL26 | 7.00E-25 | 0.401105104 | 0.993 | 0.976 | 2.18E-20 | Vein | RPL26 |
| ADGRG6 | 1.02E-24 | 0.574377045 | 0.207 | 0.024 | 3.17E-20 | Vein | ADGRG6 |
| RPL7 | 1.48E-24 | 0.402119517 | 0.988 | 0.975 | 4.60E-20 | Vein | RPL7 |
| RPS2 | 2.63E-24 | 0.418463239 | 0.997 | 0.98 | 8.18E-20 | Vein | RPS2 |
| EPB41L3 | 7.68E-24 | 0.566479468 | 0.283 | 0.069 | 2.39E-19 | Vein | EPB41L3 |
| RPL36 | 1.39E-23 | 0.375923 | 0.997 | 0.967 | 4.31E-19 | Vein | RPL36 |
| RPLP0 | 1.39E-23 | 0.431908733 | 0.977 | 0.928 | 4.31E-19 | Vein | RPLP0 |
| CPE | 1.61E-23 | 0.995453372 | 0.207 | 0.028 | 5.02E-19 | Vein | CPE |
| RPS28 | 2.41E-23 | 0.387427769 | 0.992 | 0.989 | 7.49E-19 | Vein | RPS28 |
| RPS13 | 5.87E-23 | 0.37839172 | 0.978 | 0.959 | 1.83E-18 | Vein | RPS13 |
| RPS24 | 9.58E-23 | 0.352917936 | 0.988 | 0.986 | 2.98E-18 | Vein | RPS24 |
| RPL35 | 1.05E-22 | 0.320112135 | 0.993 | 0.98 | 3.27E-18 | Vein | RPL35 |
| RPL37 | 3.20E-22 | 0.402388594 | 0.987 | 0.972 | 9.95E-18 | Vein | RPL37 |
| PLA1A | 3.33E-22 | 0.556761295 | 0.214 | 0.035 | 1.04E-17 | Vein | PLA1A |
| CLU | 3.09E-21 | 1.002874551 | 0.368 | 0.145 | 9.62E-17 | Vein | CLU |
| RPL18A | 4.43E-21 | 0.323665434 | 0.993 | 0.992 | 1.38E-16 | Vein | RPL18A |
| JUND | 4.87E-21 | 0.666947427 | 0.829 | 0.666 | 1.51E-16 | Vein | JUND |
| RPS17 | 1.77E-20 | 0.325886645 | 0.993 | 0.967 | 5.52E-16 | Vein | RPS17 |
| FAM84B | 2.05E-20 | 0.472036455 | 0.274 | 0.08 | 6.37E-16 | Vein | FAM84B |
| HLA-DRB1 | 2.29E-20 | 0.687970204 | 0.742 | 0.526 | 7.14E-16 | Vein | HLA-DRB1 |
| CEBPD | 2.49E-20 | 0.728607819 | 0.391 | 0.17 | 7.74E-16 | Vein | CEBPD |
| LRRC1 | 2.63E-20 | 0.467443486 | 0.236 | 0.055 | 8.18E-16 | Vein | LRRC1 |
| SELL | 4.85E-20 | 0.372173947 | 0.156 | 0.013 | 1.51E-15 | Vein | SELL |
| VWF | 6.29E-20 | 0.635440812 | 0.955 | 0.805 | 1.96E-15 | Vein | VWF |
| ADAMTS18 | 6.51E-20 | 0.410963886 | 0.14 | 0.006 | 2.03E-15 | Vein | ADAMTS18 |
| CADM3-AS1 | 6.64E-20 | 0.34090088 | 0.124 | 0 | 2.07E-15 | Vein | CADM3-AS1 |
| MEOX2 | 8.60E-20 | 0.425645563 | 0.196 | 0.033 | 2.68E-15 | Vein | MEOX2 |
| ITM2A | 1.70E-19 | 0.554500971 | 0.605 | 0.356 | 5.28E-15 | Vein | ITM2A |
| IER3 | 1.94E-19 | 1.008614102 | 0.686 | 0.496 | 6.03E-15 | Vein | IER3 |
| PDLIM1 | 2.18E-19 | 0.561093758 | 0.871 | 0.781 | 6.79E-15 | Vein | PDLIM1 |
| TGFBR3 | 2.42E-19 | 0.604981312 | 0.326 | 0.124 | 7.54E-15 | Vein | TGFBR3 |
| CSF3 | 3.08E-19 | 1.052272455 | 0.192 | 0.035 | 9.60E-15 | Vein | CSF3 |
| RPS5 | 4.55E-19 | 0.376193643 | 0.977 | 0.945 | 1.41E-14 | Vein | RPS5 |
| CNKSR3 | 8.87E-19 | 0.764135681 | 0.436 | 0.225 | 2.76E-14 | Vein | CNKSR3 |
| RPL11 | 1.22E-18 | 0.29340522 | 0.997 | 0.984 | 3.81E-14 | Vein | RPL11 |
| BIRC3 | 1.34E-18 | 0.586545855 | 0.314 | 0.117 | 4.17E-14 | Vein | BIRC3 |
| UGCG | 3.05E-18 | 0.582866275 | 0.493 | 0.283 | 9.48E-14 | Vein | UGCG |
| HLA-DQA1 | 3.31E-18 | 0.557323689 | 0.221 | 0.057 | 1.03E-13 | Vein | HLA-DQA1 |
| RASA4 | 5.57E-18 | 0.452416579 | 0.405 | 0.186 | 1.73E-13 | Vein | RASA4 |
| CSF2RB | 6.30E-18 | 0.585068517 | 0.361 | 0.165 | 1.96E-13 | Vein | CSF2RB |
| NFKBIA | 1.66E-17 | 0.60276025 | 0.906 | 0.813 | 5.17E-13 | Vein | NFKBIA |
| RPL19 | 2.03E-17 | 0.294515977 | 0.99 | 0.973 | 6.33E-13 | Vein | RPL19 |
| FOS | 2.73E-17 | 0.735624097 | 0.871 | 0.723 | 8.49E-13 | Vein | FOS |
| RPL41 | 5.03E-17 | 0.32680205 | 0.998 | 0.992 | 1.57E-12 | Vein | RPL41 |
| RPL35A | 8.65E-17 | 0.288461389 | 0.99 | 0.969 | 2.69E-12 | Vein | RPL35A |
| RPL10A | 9.29E-17 | 0.340408921 | 0.968 | 0.945 | 2.89E-12 | Vein | RPL10A |
| CMIP | 2.02E-16 | 0.513128783 | 0.62 | 0.433 | 6.30E-12 | Vein | CMIP |
| RPS25 | 2.16E-16 | 0.258471001 | 0.99 | 0.965 | 6.71E-12 | Vein | RPS25 |
| VCAN | 3.07E-16 | 0.487440837 | 0.147 | 0.02 | 9.56E-12 | Vein | VCAN |
| TINAGL1 | 3.10E-16 | 0.432488269 | 0.62 | 0.419 | 9.66E-12 | Vein | TINAGL1 |
| LHX6 | 3.30E-16 | 0.435127702 | 0.283 | 0.109 | 1.03E-11 | Vein | LHX6 |
| KLF6 | 3.40E-16 | 0.543940851 | 0.881 | 0.78 | 1.06E-11 | Vein | KLF6 |
| RPL18 | 3.41E-16 | 0.28561891 | 0.993 | 0.967 | 1.06E-11 | Vein | RPL18 |
| HLA-DQB1 | 4.52E-16 | 0.530519512 | 0.328 | 0.145 | 1.41E-11 | Vein | HLA-DQB1 |
| SYT15 | 7.64E-16 | 0.359448959 | 0.167 | 0.031 | 2.38E-11 | Vein | SYT15 |
| RPL6 | 1.05E-15 | 0.321182987 | 0.963 | 0.939 | 3.28E-11 | Vein | RPL6 |
| RPS3 | 1.52E-15 | 0.305280503 | 0.99 | 0.97 | 4.74E-11 | Vein | RPS3 |
| SNCG | 1.89E-15 | 0.461440791 | 0.584 | 0.405 | 5.87E-11 | Vein | SNCG |
| DAAM1 | 1.89E-15 | 0.560138841 | 0.478 | 0.28 | 5.89E-11 | Vein | DAAM1 |
| HIF1A | 2.16E-15 | 0.477459838 | 0.722 | 0.55 | 6.72E-11 | Vein | HIF1A |
| LITAF | 2.18E-15 | 0.665541677 | 0.515 | 0.332 | 6.80E-11 | Vein | LITAF |
| HLA-DMA | 2.25E-15 | 0.465631411 | 0.435 | 0.238 | 7.01E-11 | Vein | HLA-DMA |
| NDRG1 | 2.77E-15 | 0.534513863 | 0.724 | 0.583 | 8.62E-11 | Vein | NDRG1 |
| SLC2A3 | 3.03E-15 | 0.475952947 | 0.689 | 0.483 | 9.44E-11 | Vein | SLC2A3 |
| ICAM1 | 6.91E-15 | 0.838381897 | 0.645 | 0.49 | 2.15E-10 | Vein | ICAM1 |
| CCL14 | 1.06E-14 | 0.518662715 | 0.236 | 0.079 | 3.31E-10 | Vein | CCL14 |
| LCN6 | 1.10E-14 | 0.443739971 | 0.242 | 0.08 | 3.42E-10 | Vein | LCN6 |
| SESN3 | 1.12E-14 | 0.44439682 | 0.227 | 0.076 | 3.49E-10 | Vein | SESN3 |
| JUN | 1.15E-14 | 0.627988427 | 0.855 | 0.698 | 3.59E-10 | Vein | JUN |
| CRTAC1 | 1.20E-14 | 0.382678532 | 0.129 | 0.016 | 3.74E-10 | Vein | CRTAC1 |
| RPS4X | 1.67E-14 | 0.325762505 | 0.992 | 0.962 | 5.21E-10 | Vein | RPS4X |
| TLL1 | 2.34E-14 | 0.316145392 | 0.164 | 0.036 | 7.29E-10 | Vein | TLL1 |
| SLC8A1 | 2.61E-14 | 0.294182947 | 0.125 | 0.016 | 8.13E-10 | Vein | SLC8A1 |
| ARRDC3 | 2.66E-14 | 0.569405277 | 0.505 | 0.309 | 8.29E-10 | Vein | ARRDC3 |
| S100A10 | 2.70E-14 | 0.463146932 | 0.952 | 0.894 | 8.41E-10 | Vein | S100A10 |
| RPS27 | 3.31E-14 | 0.284504712 | 0.993 | 0.992 | 1.03E-09 | Vein | RPS27 |
| PLAC9 | 3.46E-14 | 0.373529466 | 0.212 | 0.061 | 1.08E-09 | Vein | PLAC9 |
| YBX3 | 3.52E-14 | 0.45707727 | 0.826 | 0.699 | 1.10E-09 | Vein | YBX3 |
| RPL24 | 4.90E-14 | 0.254120963 | 0.978 | 0.953 | 1.53E-09 | Vein | RPL24 |
| ACTN1 | 8.40E-14 | 0.436742501 | 0.722 | 0.554 | 2.61E-09 | Vein | ACTN1 |
| SOD2 | 8.46E-14 | 0.942598667 | 0.605 | 0.479 | 2.63E-09 | Vein | SOD2 |
| COTL1 | 1.15E-13 | 0.56569866 | 0.607 | 0.413 | 3.59E-09 | Vein | COTL1 |
| ENTPD1 | 1.52E-13 | 0.455410232 | 0.667 | 0.485 | 4.73E-09 | Vein | ENTPD1 |
| RPS21 | 1.61E-13 | 0.307245303 | 0.982 | 0.934 | 5.01E-09 | Vein | RPS21 |
| MADCAM1 | 1.80E-13 | 0.75863759 | 0.109 | 0.011 | 5.59E-09 | Vein | MADCAM1 |
| TGM2 | 2.77E-13 | 0.595111212 | 0.671 | 0.535 | 8.63E-09 | Vein | TGM2 |
| RPL29 | 3.35E-13 | 0.260218157 | 0.985 | 0.97 | 1.04E-08 | Vein | RPL29 |
| PMP22 | 4.24E-13 | 0.451292243 | 0.532 | 0.357 | 1.32E-08 | Vein | PMP22 |
| RAI14 | 4.61E-13 | 0.453885302 | 0.651 | 0.509 | 1.44E-08 | Vein | RAI14 |
| KLF4 | 5.98E-13 | 0.485446434 | 0.625 | 0.428 | 1.86E-08 | Vein | KLF4 |
| PABPC1 | 7.03E-13 | 0.367158804 | 0.881 | 0.776 | 2.19E-08 | Vein | PABPC1 |
| ST6GAL1 | 8.03E-13 | 0.303177536 | 0.274 | 0.117 | 2.50E-08 | Vein | ST6GAL1 |
| EEF1A1 | 1.63E-12 | 0.25305035 | 1 | 0.994 | 5.06E-08 | Vein | EEF1A1 |
| SERPINE1 | 1.99E-12 | 0.644996746 | 0.657 | 0.509 | 6.21E-08 | Vein | SERPINE1 |
| LPCAT4 | 2.05E-12 | 0.388237563 | 0.264 | 0.115 | 6.38E-08 | Vein | LPCAT4 |
| RPL30 | 2.07E-12 | 0.252786415 | 0.977 | 0.964 | 6.45E-08 | Vein | RPL30 |
| SOCS3 | 2.15E-12 | 0.573216982 | 0.749 | 0.583 | 6.68E-08 | Vein | SOCS3 |
| FOXC1 | 2.39E-12 | 0.467757705 | 0.259 | 0.113 | 7.42E-08 | Vein | FOXC1 |
| PKP4 | 2.74E-12 | 0.324989138 | 0.472 | 0.296 | 8.51E-08 | Vein | PKP4 |
| NET1 | 2.82E-12 | 0.384780847 | 0.266 | 0.117 | 8.78E-08 | Vein | NET1 |
| EEF1B2 | 3.06E-12 | 0.349439272 | 0.893 | 0.809 | 9.52E-08 | Vein | EEF1B2 |
| DOC2B | 3.55E-12 | 0.408779126 | 0.331 | 0.173 | 1.11E-07 | Vein | DOC2B |
| RND1 | 3.70E-12 | 0.471412823 | 0.455 | 0.279 | 1.15E-07 | Vein | RND1 |
| FOSB | 4.99E-12 | 0.503618582 | 0.761 | 0.606 | 1.55E-07 | Vein | FOSB |
| LINC-PINT | 5.62E-12 | 0.299377852 | 0.237 | 0.094 | 1.75E-07 | Vein | LINC-PINT |
| SNTB2 | 1.05E-11 | 0.401429583 | 0.505 | 0.343 | 3.26E-07 | Vein | SNTB2 |
| TPD52L1 | 1.85E-11 | 0.355245104 | 0.284 | 0.135 | 5.76E-07 | Vein | TPD52L1 |
| RPL7A | 2.54E-11 | 0.270323091 | 0.983 | 0.954 | 7.92E-07 | Vein | RPL7A |
| MT-ND1 | 2.59E-11 | 0.280907753 | 0.993 | 0.972 | 8.07E-07 | Vein | MT-ND1 |
| ZFAND5 | 2.63E-11 | 0.487397472 | 0.689 | 0.584 | 8.19E-07 | Vein | ZFAND5 |
| EEF1D | 3.35E-11 | 0.265363797 | 0.953 | 0.902 | 1.04E-06 | Vein | EEF1D |
| CYTH1 | 3.40E-11 | 0.329874474 | 0.433 | 0.265 | 1.06E-06 | Vein | CYTH1 |
| NAMPT | 3.73E-11 | 0.461174554 | 0.689 | 0.551 | 1.16E-06 | Vein | NAMPT |
| APLNR | 5.49E-11 | 0.482307926 | 0.652 | 0.465 | 1.71E-06 | Vein | APLNR |
| LIFR | 1.38E-10 | 0.439504688 | 0.226 | 0.096 | 4.31E-06 | Vein | LIFR |
| RPL3 | 2.60E-10 | 0.262683559 | 0.993 | 0.976 | 8.08E-06 | Vein | RPL3 |
| MALL | 2.75E-10 | 0.342052921 | 0.642 | 0.49 | 8.56E-06 | Vein | MALL |
| IL15 | 2.86E-10 | 0.298852033 | 0.197 | 0.079 | 8.91E-06 | Vein | IL15 |
| C1QTNF1 | 5.01E-10 | 0.319310571 | 0.11 | 0.024 | 1.56E-05 | Vein | C1QTNF1 |
| CPD | 5.04E-10 | 0.393477478 | 0.396 | 0.255 | 1.57E-05 | Vein | CPD |
| ICAM4 | 6.15E-10 | 0.347351129 | 0.224 | 0.098 | 1.91E-05 | Vein | ICAM4 |
| AHR | 6.57E-10 | 0.340015178 | 0.446 | 0.29 | 2.04E-05 | Vein | AHR |
| DLC1 | 6.92E-10 | 0.377192712 | 0.572 | 0.425 | 2.15E-05 | Vein | DLC1 |
| CFLAR | 7.09E-10 | 0.345969055 | 0.756 | 0.624 | 2.21E-05 | Vein | CFLAR |
| TSHZ2 | 1.01E-09 | 0.462102782 | 0.575 | 0.419 | 3.13E-05 | Vein | TSHZ2 |
| RALGAPA2 | 1.07E-09 | 0.420851302 | 0.395 | 0.255 | 3.34E-05 | Vein | RALGAPA2 |
| FKBP11 | 1.23E-09 | 0.346562193 | 0.339 | 0.203 | 3.82E-05 | Vein | FKBP11 |
| C4orf32 | 1.41E-09 | 0.404418443 | 0.436 | 0.302 | 4.37E-05 | Vein | C4orf32 |
| BMP2 | 1.50E-09 | 0.353857992 | 0.186 | 0.074 | 4.67E-05 | Vein | BMP2 |
| CSRP2 | 1.51E-09 | 0.424998577 | 0.39 | 0.247 | 4.70E-05 | Vein | CSRP2 |
| LY6E | 1.53E-09 | 0.362371967 | 0.652 | 0.491 | 4.77E-05 | Vein | LY6E |
| MAP3K8 | 1.64E-09 | 0.474620385 | 0.355 | 0.222 | 5.12E-05 | Vein | MAP3K8 |
| CTNNAL1 | 2.12E-09 | 0.377720589 | 0.395 | 0.25 | 6.58E-05 | Vein | CTNNAL1 |
| DIXDC1 | 2.44E-09 | 0.275242478 | 0.274 | 0.143 | 7.59E-05 | Vein | DIXDC1 |
| NEAT1 | 2.45E-09 | 0.427747611 | 0.978 | 0.912 | 7.62E-05 | Vein | NEAT1 |
| FAM171A1 | 4.01E-09 | 0.321885668 | 0.324 | 0.187 | 0.000124654 | Vein | FAM171A1 |
| RP11-138A9.1 | 4.54E-09 | 0.296990348 | 0.278 | 0.151 | 0.000141168 | Vein | RP11-138A9.1 |
| EVA1C | 5.35E-09 | 0.274888522 | 0.343 | 0.198 | 0.000166357 | Vein | EVA1C |
| HIPK3 | 5.74E-09 | 0.354377666 | 0.39 | 0.257 | 0.000178496 | Vein | HIPK3 |
| MCL1 | 6.36E-09 | 0.290638639 | 0.776 | 0.657 | 0.000197962 | Vein | MCL1 |
| NFATC2 | 8.35E-09 | 0.304749113 | 0.256 | 0.132 | 0.000259827 | Vein | NFATC2 |
| ETS2 | 9.47E-09 | 0.274198277 | 0.769 | 0.622 | 0.000294805 | Vein | ETS2 |
| CTSC | 1.02E-08 | 0.445524931 | 0.376 | 0.247 | 0.000317748 | Vein | CTSC |
| MYOF | 1.88E-08 | 0.281533539 | 0.349 | 0.216 | 0.000585933 | Vein | MYOF |
| PNRC1 | 2.14E-08 | 0.335360327 | 0.764 | 0.665 | 0.000665639 | Vein | PNRC1 |
| DUSP1 | 3.29E-08 | 0.285406854 | 0.871 | 0.762 | 0.001024682 | Vein | DUSP1 |
| CDKN3 | 3.54E-08 | 0.264102713 | 0.161 | 0.065 | 0.001101714 | Vein | CDKN3 |
| MBNL1 | 3.97E-08 | 0.392588881 | 0.569 | 0.455 | 0.001235252 | Vein | MBNL1 |
| HMGN3 | 4.13E-08 | 0.279831822 | 0.734 | 0.631 | 0.001284525 | Vein | HMGN3 |
| CLSTN3 | 4.35E-08 | 0.26364591 | 0.279 | 0.157 | 0.001354842 | Vein | CLSTN3 |
| CDC42EP3 | 5.79E-08 | 0.282861146 | 0.53 | 0.394 | 0.001801014 | Vein | CDC42EP3 |
| SPNS2 | 6.29E-08 | 0.304773198 | 0.383 | 0.26 | 0.00195723 | Vein | SPNS2 |
| CALCRL | 7.24E-08 | 0.297926803 | 0.798 | 0.688 | 0.002253686 | Vein | CALCRL |
| ARL4C | 1.40E-07 | 0.261262298 | 0.283 | 0.165 | 0.004359389 | Vein | ARL4C |
| GLTSCR2 | 1.61E-07 | 0.300476639 | 0.808 | 0.759 | 0.005008474 | Vein | GLTSCR2 |
| TGFBR2 | 1.75E-07 | 0.28613883 | 0.771 | 0.661 | 0.005445119 | Vein | TGFBR2 |
| BNC2 | 1.84E-07 | 0.250754548 | 0.182 | 0.085 | 0.005711635 | Vein | BNC2 |
| LDHA | 1.89E-07 | 0.268001932 | 0.849 | 0.773 | 0.005893148 | Vein | LDHA |
| ARID5B | 2.25E-07 | 0.427911275 | 0.532 | 0.433 | 0.00700907 | Vein | ARID5B |
| RPL17 | 2.69E-07 | 0.283391281 | 0.784 | 0.694 | 0.008369816 | Vein | RPL17 |
| NBEAL1 | 2.88E-07 | 0.283912184 | 0.647 | 0.535 | 0.008966561 | Vein | NBEAL1 |
| SNHG8 | 3.58E-07 | 0.273423486 | 0.632 | 0.524 | 0.011145611 | Vein | SNHG8 |
| RPL36A | 3.96E-07 | 0.292737175 | 0.921 | 0.868 | 0.012326263 | Vein | RPL36A |
| EIF3E | 4.28E-07 | 0.302197608 | 0.803 | 0.706 | 0.013327483 | Vein | EIF3E |
| G0S2 | 4.85E-07 | 0.324572639 | 0.164 | 0.076 | 0.015100897 | Vein | G0S2 |
| RGS16 | 5.29E-07 | 0.496270665 | 0.326 | 0.211 | 0.016474114 | Vein | RGS16 |
| CCNL1 | 5.95E-07 | 0.351860322 | 0.731 | 0.647 | 0.018517021 | Vein | CCNL1 |
| TNFAIP3 | 7.04E-07 | 0.432976008 | 0.457 | 0.34 | 0.02190328 | Vein | TNFAIP3 |
| ZC3H12A | 7.57E-07 | 0.255012281 | 0.273 | 0.162 | 0.023540953 | Vein | ZC3H12A |
| PDK4 | 8.96E-07 | 0.50063711 | 0.271 | 0.167 | 0.027865884 | Vein | PDK4 |
| PPP1R15A | 9.25E-07 | 0.348821469 | 0.749 | 0.627 | 0.028767282 | Vein | PPP1R15A |
| PDLIM4 | 9.83E-07 | 0.283914682 | 0.525 | 0.414 | 0.030580652 | Vein | PDLIM4 |
| PBX1 | 1.19E-06 | 0.255829555 | 0.227 | 0.129 | 0.036900589 | Vein | PBX1 |
| MCTP1 | 1.34E-06 | 0.305046688 | 0.473 | 0.359 | 0.041713051 | Vein | MCTP1 |
| CTSH | 1.45E-06 | 0.308824524 | 0.433 | 0.321 | 0.0451854 | Vein | CTSH |
| ITGA10 | 1.46E-06 | 0.301318332 | 0.206 | 0.11 | 0.045524483 | Vein | ITGA10 |
| SPAG9 | 1.60E-06 | 0.298540436 | 0.498 | 0.4 | 0.049762694 | Vein | SPAG9 |
| COL4A1 | 2.85E-86 | 1.530026195 | 0.993 | 0.824 | 8.88E-82 | TIP | COL4A1 |
| SPARC | 8.22E-85 | 1.281360021 | 1 | 0.915 | 2.56E-80 | TIP | SPARC |
| TP53I11 | 7.42E-81 | 1.340607355 | 0.723 | 0.16 | 2.31E-76 | TIP | TP53I11 |
| ESM1 | 9.88E-81 | 2.070021627 | 0.633 | 0.101 | 3.07E-76 | TIP | ESM1 |
| COL4A2 | 6.24E-68 | 1.268547922 | 0.978 | 0.792 | 1.94E-63 | TIP | COL4A2 |
| NID2 | 2.17E-62 | 0.913077702 | 0.489 | 0.07 | 6.76E-58 | TIP | NID2 |
| RGCC | 4.80E-62 | 1.393043134 | 0.953 | 0.554 | 1.49E-57 | TIP | RGCC |
| CA2 | 7.02E-60 | 1.111611751 | 0.529 | 0.092 | 2.18E-55 | TIP | CA2 |
| ANGPTL2 | 1.38E-54 | 1.031221023 | 0.698 | 0.222 | 4.31E-50 | TIP | ANGPTL2 |
| GABRD | 4.52E-53 | 0.751500181 | 0.363 | 0.036 | 1.41E-48 | TIP | GABRD |
| PDGFB | 5.70E-50 | 0.906051066 | 0.68 | 0.218 | 1.77E-45 | TIP | PDGFB |
| TSPAN15 | 1.17E-49 | 0.854466783 | 0.651 | 0.201 | 3.63E-45 | TIP | TSPAN15 |
| MCAM | 8.44E-49 | 1.083478372 | 0.932 | 0.547 | 2.63E-44 | TIP | MCAM |
| LBH | 4.18E-47 | 1.019643569 | 0.694 | 0.259 | 1.30E-42 | TIP | LBH |
| RGS3 | 1.70E-45 | 1.104681535 | 0.871 | 0.453 | 5.30E-41 | TIP | RGS3 |
| GNG2 | 1.50E-44 | 0.564693644 | 0.439 | 0.082 | 4.68E-40 | TIP | GNG2 |
| APLN | 2.65E-44 | 1.308521075 | 0.432 | 0.084 | 8.25E-40 | TIP | APLN |
| PXDN | 2.98E-44 | 1.041942899 | 0.791 | 0.365 | 9.28E-40 | TIP | PXDN |
| CALM1 | 7.19E-44 | 0.969893621 | 0.968 | 0.873 | 2.24E-39 | TIP | CALM1 |
| VWA1 | 7.35E-44 | 0.923603904 | 0.964 | 0.668 | 2.29E-39 | TIP | VWA1 |
| NOTCH4 | 6.23E-43 | 0.803898156 | 0.899 | 0.432 | 1.94E-38 | TIP | NOTCH4 |
| FSCN1 | 1.29E-42 | 0.89874688 | 0.871 | 0.477 | 4.00E-38 | TIP | FSCN1 |
| PLAU | 1.65E-42 | 0.68369185 | 0.558 | 0.149 | 5.14E-38 | TIP | PLAU |
| PLVAP | 2.02E-41 | 0.987589465 | 0.993 | 0.821 | 6.27E-37 | TIP | PLVAP |
| COL12A1 | 7.91E-41 | 0.681035931 | 0.424 | 0.087 | 2.46E-36 | TIP | COL12A1 |
| UNC5B | 1.73E-40 | 0.711905311 | 0.54 | 0.143 | 5.38E-36 | TIP | UNC5B |
| MYO1B | 8.89E-40 | 0.73737512 | 0.68 | 0.252 | 2.77E-35 | TIP | MYO1B |
| PCDH12 | 1.40E-38 | 0.740610944 | 0.644 | 0.225 | 4.37E-34 | TIP | PCDH12 |
| TNFRSF4 | 2.04E-38 | 1.035185749 | 0.705 | 0.285 | 6.34E-34 | TIP | TNFRSF4 |
| GMFG | 2.44E-38 | 0.802265807 | 0.881 | 0.494 | 7.60E-34 | TIP | GMFG |
| ROBO4 | 2.56E-38 | 0.673739538 | 0.856 | 0.437 | 7.98E-34 | TIP | ROBO4 |
| TNFAIP8L1 | 2.92E-38 | 0.734533113 | 0.687 | 0.269 | 9.08E-34 | TIP | TNFAIP8L1 |
| ACTB | 8.26E-38 | 0.814403029 | 1 | 0.988 | 2.57E-33 | TIP | ACTB |
| PGF | 6.50E-37 | 1.576871783 | 0.662 | 0.291 | 2.02E-32 | TIP | PGF |
| KCNQ1 | 1.83E-36 | 0.334257098 | 0.255 | 0.025 | 5.70E-32 | TIP | KCNQ1 |
| JUP | 3.73E-36 | 0.642731895 | 0.716 | 0.291 | 1.16E-31 | TIP | JUP |
| LAMA4 | 4.04E-36 | 0.833733454 | 0.885 | 0.545 | 1.26E-31 | TIP | LAMA4 |
| ANGPT2 | 4.10E-36 | 0.956841851 | 0.669 | 0.25 | 1.28E-31 | TIP | ANGPT2 |
| KDR | 6.17E-36 | 0.74598453 | 0.784 | 0.352 | 1.92E-31 | TIP | KDR |
| TMEM233 | 2.89E-35 | 0.57489031 | 0.424 | 0.102 | 8.99E-31 | TIP | TMEM233 |
| LGALS1 | 5.30E-35 | 0.899318887 | 0.975 | 0.872 | 1.65E-30 | TIP | LGALS1 |
| SPRY4 | 5.81E-35 | 0.586999215 | 0.586 | 0.198 | 1.81E-30 | TIP | SPRY4 |
| FAM101B | 6.58E-35 | 0.64831349 | 0.705 | 0.298 | 2.05E-30 | TIP | FAM101B |
| TTYH2 | 1.56E-34 | 0.305017405 | 0.201 | 0.012 | 4.85E-30 | TIP | TTYH2 |
| DYSF | 3.24E-33 | 0.643614646 | 0.737 | 0.329 | 1.01E-28 | TIP | DYSF |
| COL18A1 | 7.53E-33 | 0.84812679 | 0.906 | 0.619 | 2.34E-28 | TIP | COL18A1 |
| LOXL2 | 1.01E-32 | 0.611441712 | 0.54 | 0.177 | 3.14E-28 | TIP | LOXL2 |
| HSPG2 | 1.26E-32 | 0.706873659 | 0.996 | 0.896 | 3.91E-28 | TIP | HSPG2 |
| INSR | 1.98E-32 | 0.864585391 | 0.914 | 0.563 | 6.17E-28 | TIP | INSR |
| ACTG1 | 2.56E-32 | 0.676290807 | 0.993 | 0.955 | 7.98E-28 | TIP | ACTG1 |
| ARHGAP18 | 3.01E-32 | 0.64466266 | 0.576 | 0.215 | 9.37E-28 | TIP | ARHGAP18 |
| ADGRF5 | 3.94E-32 | 0.635823931 | 0.888 | 0.488 | 1.23E-27 | TIP | ADGRF5 |
| TM4SF18 | 3.13E-31 | 0.667411397 | 0.799 | 0.412 | 9.75E-27 | TIP | TM4SF18 |
| MYL9 | 5.02E-31 | 1.026002538 | 0.734 | 0.39 | 1.56E-26 | TIP | MYL9 |
| LXN | 1.04E-30 | 0.684049257 | 0.568 | 0.219 | 3.24E-26 | TIP | LXN |
| CHST1 | 1.12E-30 | 0.526947489 | 0.435 | 0.119 | 3.49E-26 | TIP | CHST1 |
| SOX11 | 1.28E-30 | 0.295969798 | 0.173 | 0.008 | 3.98E-26 | TIP | SOX11 |
| COL27A1 | 1.41E-30 | 0.370267 | 0.299 | 0.051 | 4.40E-26 | TIP | COL27A1 |
| EIF4EBP1 | 1.90E-30 | 0.661301667 | 0.68 | 0.315 | 5.91E-26 | TIP | EIF4EBP1 |
| NES | 2.46E-30 | 0.577334059 | 0.694 | 0.294 | 7.65E-26 | TIP | NES |
| ADAMTS7 | 3.71E-30 | 0.582613263 | 0.421 | 0.115 | 1.15E-25 | TIP | ADAMTS7 |
| PLXNA2 | 6.15E-30 | 0.540447397 | 0.705 | 0.313 | 1.91E-25 | TIP | PLXNA2 |
| PFN1 | 1.93E-29 | 0.645554556 | 0.993 | 0.918 | 6.00E-25 | TIP | PFN1 |
| DLL4 | 2.54E-29 | 0.518879577 | 0.594 | 0.216 | 7.89E-25 | TIP | DLL4 |
| MMP14 | 5.79E-29 | 0.726981845 | 0.694 | 0.326 | 1.80E-24 | TIP | MMP14 |
| EFNA1 | 1.16E-28 | 0.648602732 | 0.845 | 0.461 | 3.60E-24 | TIP | EFNA1 |
| KLHDC8A | 1.83E-28 | 0.259429217 | 0.176 | 0.013 | 5.70E-24 | TIP | KLHDC8A |
| COLGALT1 | 2.09E-28 | 0.473630524 | 0.665 | 0.282 | 6.51E-24 | TIP | COLGALT1 |
| COL15A1 | 2.25E-28 | 0.720297351 | 0.95 | 0.709 | 6.99E-24 | TIP | COL15A1 |
| LINC00152 | 7.86E-28 | 0.775511872 | 0.896 | 0.581 | 2.45E-23 | TIP | LINC00152 |
| APLP2 | 1.44E-27 | 0.691941027 | 0.928 | 0.653 | 4.47E-23 | TIP | APLP2 |
| CCDC85B | 2.49E-27 | 0.518890411 | 0.971 | 0.865 | 7.74E-23 | TIP | CCDC85B |
| CDH13 | 2.63E-27 | 0.626383851 | 0.719 | 0.352 | 8.18E-23 | TIP | CDH13 |
| FLT1 | 4.07E-27 | 0.872428402 | 0.871 | 0.532 | 1.27E-22 | TIP | FLT1 |
| RCAN2 | 5.60E-27 | 0.394651719 | 0.414 | 0.121 | 1.74E-22 | TIP | RCAN2 |
| SLC27A3 | 1.48E-26 | 0.414295216 | 0.442 | 0.136 | 4.59E-22 | TIP | SLC27A3 |
| CFL1 | 1.50E-26 | 0.527574325 | 0.975 | 0.934 | 4.68E-22 | TIP | CFL1 |
| THY1 | 2.03E-26 | 0.630440076 | 0.507 | 0.184 | 6.31E-22 | TIP | THY1 |
| DGKD | 2.38E-26 | 0.387110754 | 0.388 | 0.11 | 7.40E-22 | TIP | DGKD |
| ESAM | 2.44E-26 | 0.600451982 | 0.935 | 0.682 | 7.58E-22 | TIP | ESAM |
| SERPINH1 | 3.12E-26 | 0.647491185 | 0.942 | 0.69 | 9.70E-22 | TIP | SERPINH1 |
| IVNS1ABP | 3.96E-26 | 0.733185794 | 0.701 | 0.338 | 1.23E-21 | TIP | IVNS1ABP |
| TMEM204 | 4.05E-26 | 0.644989764 | 0.734 | 0.379 | 1.26E-21 | TIP | TMEM204 |
| EFNB1 | 4.96E-26 | 0.411224577 | 0.547 | 0.203 | 1.54E-21 | TIP | EFNB1 |
| MIR4435-2HG | 1.09E-25 | 0.60427101 | 0.853 | 0.492 | 3.39E-21 | TIP | MIR4435-2HG |
| IL32 | 1.38E-25 | 0.862990919 | 0.892 | 0.63 | 4.30E-21 | TIP | IL32 |
| FCN3 | 1.67E-25 | 0.717763072 | 0.392 | 0.114 | 5.20E-21 | TIP | FCN3 |
| LAMB1 | 1.95E-25 | 0.636343438 | 0.723 | 0.378 | 6.06E-21 | TIP | LAMB1 |
| CXCR4 | 2.78E-25 | 0.697971239 | 0.403 | 0.13 | 8.64E-21 | TIP | CXCR4 |
| CD34 | 2.83E-25 | 0.59332477 | 0.928 | 0.642 | 8.80E-21 | TIP | CD34 |
| CYTL1 | 4.55E-25 | 0.736353641 | 0.36 | 0.105 | 1.41E-20 | TIP | CYTL1 |
| PASK | 6.33E-25 | 0.38872652 | 0.194 | 0.024 | 1.97E-20 | TIP | PASK |
| PMEPA1 | 1.02E-24 | 0.677507789 | 0.676 | 0.324 | 3.17E-20 | TIP | PMEPA1 |
| IGFBP7 | 1.23E-24 | 0.647395068 | 1 | 0.979 | 3.83E-20 | TIP | IGFBP7 |
| GYPC | 1.65E-24 | 0.639311939 | 0.698 | 0.352 | 5.15E-20 | TIP | GYPC |
| MAGED2 | 1.70E-24 | 0.598501118 | 0.73 | 0.38 | 5.29E-20 | TIP | MAGED2 |
| MICALL2 | 2.24E-24 | 0.318462987 | 0.353 | 0.095 | 6.97E-20 | TIP | MICALL2 |
| IGFBP3 | 2.86E-24 | 0.637946887 | 0.77 | 0.396 | 8.90E-20 | TIP | IGFBP3 |
| GRPEL2 | 3.17E-24 | 0.455106832 | 0.45 | 0.155 | 9.86E-20 | TIP | GRPEL2 |
| GPR4 | 3.27E-24 | 0.471051689 | 0.532 | 0.212 | 1.02E-19 | TIP | GPR4 |
| IDH2 | 4.62E-24 | 0.51679987 | 0.543 | 0.23 | 1.44E-19 | TIP | IDH2 |
| CLIC1 | 4.96E-24 | 0.517923018 | 0.957 | 0.816 | 1.54E-19 | TIP | CLIC1 |
| GRB10 | 5.23E-24 | 0.560663046 | 0.673 | 0.327 | 1.63E-19 | TIP | GRB10 |
| GSN | 5.88E-24 | 0.746481173 | 0.939 | 0.705 | 1.83E-19 | TIP | GSN |
| VASH1 | 7.20E-24 | 0.485107595 | 0.55 | 0.221 | 2.24E-19 | TIP | VASH1 |
| CD276 | 7.22E-24 | 0.410479903 | 0.536 | 0.209 | 2.25E-19 | TIP | CD276 |
| AC133644.2 | 1.37E-23 | 0.284705294 | 0.263 | 0.054 | 4.27E-19 | TIP | AC133644.2 |
| YWHAH | 2.31E-23 | 0.586976842 | 0.878 | 0.622 | 7.20E-19 | TIP | YWHAH |
| CSTB | 2.53E-23 | 0.604143867 | 0.953 | 0.748 | 7.88E-19 | TIP | CSTB |
| NID1 | 2.69E-23 | 0.618867398 | 0.709 | 0.364 | 8.36E-19 | TIP | NID1 |
| IKBIP | 3.04E-23 | 0.383821752 | 0.583 | 0.246 | 9.47E-19 | TIP | IKBIP |
| FHL3 | 4.12E-23 | 0.341324618 | 0.446 | 0.149 | 1.28E-18 | TIP | FHL3 |
| AFAP1L1 | 5.45E-23 | 0.50591459 | 0.608 | 0.285 | 1.70E-18 | TIP | AFAP1L1 |
| C16orf74 | 6.97E-23 | 0.298036009 | 0.295 | 0.07 | 2.17E-18 | TIP | C16orf74 |
| PLOD1 | 9.68E-23 | 0.451094297 | 0.612 | 0.268 | 3.01E-18 | TIP | PLOD1 |
| ARHGAP6 | 9.80E-23 | 0.330023697 | 0.183 | 0.024 | 3.05E-18 | TIP | ARHGAP6 |
| LDHB | 1.01E-22 | 0.577469169 | 0.853 | 0.614 | 3.15E-18 | TIP | LDHB |
| CDC42EP1 | 1.11E-22 | 0.430555321 | 0.629 | 0.287 | 3.46E-18 | TIP | CDC42EP1 |
| F2RL3 | 1.23E-22 | 0.590677329 | 0.683 | 0.338 | 3.82E-18 | TIP | F2RL3 |
| PODXL | 1.47E-22 | 0.568023415 | 0.917 | 0.57 | 4.58E-18 | TIP | PODXL |
| LAMC1 | 2.14E-22 | 0.609239403 | 0.795 | 0.479 | 6.65E-18 | TIP | LAMC1 |
| PDGFA | 2.29E-22 | 0.430802728 | 0.313 | 0.084 | 7.12E-18 | TIP | PDGFA |
| CMTM3 | 2.76E-22 | 0.405396001 | 0.482 | 0.186 | 8.60E-18 | TIP | CMTM3 |
| BCAT1 | 3.35E-22 | 0.333964066 | 0.331 | 0.093 | 1.04E-17 | TIP | BCAT1 |
| KIT | 3.73E-22 | 0.319758497 | 0.165 | 0.019 | 1.16E-17 | TIP | KIT |
| PTP4A3 | 4.85E-22 | 0.461804901 | 0.665 | 0.311 | 1.51E-17 | TIP | PTP4A3 |
| COL22A1 | 6.21E-22 | 0.260813001 | 0.14 | 0.012 | 1.93E-17 | TIP | COL22A1 |
| CCND1 | 6.45E-22 | 0.585854157 | 0.615 | 0.296 | 2.01E-17 | TIP | CCND1 |
| FOLH1 | 7.37E-22 | 0.282728672 | 0.223 | 0.041 | 2.29E-17 | TIP | FOLH1 |
| STC2 | 8.06E-22 | 0.367348572 | 0.421 | 0.145 | 2.51E-17 | TIP | STC2 |
| ARHGDIB | 9.64E-22 | 0.538512161 | 0.881 | 0.632 | 3.00E-17 | TIP | ARHGDIB |
| NRP2 | 9.89E-22 | 0.48098997 | 0.712 | 0.362 | 3.08E-17 | TIP | NRP2 |
| SNTB1 | 1.01E-21 | 0.252285982 | 0.155 | 0.016 | 3.14E-17 | TIP | SNTB1 |
| NDST1 | 1.92E-21 | 0.436954454 | 0.615 | 0.291 | 5.97E-17 | TIP | NDST1 |
| MRPL17 | 2.01E-21 | 0.41700373 | 0.737 | 0.407 | 6.25E-17 | TIP | MRPL17 |
| PLXND1 | 2.31E-21 | 0.584866457 | 0.86 | 0.535 | 7.20E-17 | TIP | PLXND1 |
| KCNJ2 | 2.61E-21 | 0.332604637 | 0.273 | 0.065 | 8.11E-17 | TIP | KCNJ2 |
| CLIC4 | 3.80E-21 | 0.522705335 | 0.853 | 0.536 | 1.18E-16 | TIP | CLIC4 |
| MMP15 | 4.67E-21 | 0.364886876 | 0.435 | 0.16 | 1.45E-16 | TIP | MMP15 |
| RASSF2 | 5.62E-21 | 0.348645392 | 0.273 | 0.068 | 1.75E-16 | TIP | RASSF2 |
| DBN1 | 6.03E-21 | 0.40613781 | 0.561 | 0.246 | 1.88E-16 | TIP | DBN1 |
| ITGB1 | 6.49E-21 | 0.510312848 | 0.957 | 0.793 | 2.02E-16 | TIP | ITGB1 |
| N4BP3 | 6.67E-21 | 0.274847746 | 0.273 | 0.067 | 2.08E-16 | TIP | N4BP3 |
| CHST15 | 6.87E-21 | 0.349945816 | 0.284 | 0.074 | 2.14E-16 | TIP | CHST15 |
| CD99 | 9.01E-21 | 0.528343822 | 0.957 | 0.799 | 2.80E-16 | TIP | CD99 |
| ITIH5 | 9.03E-21 | 0.615778807 | 0.421 | 0.169 | 2.81E-16 | TIP | ITIH5 |
| AGRN | 1.01E-20 | 0.496611312 | 0.615 | 0.292 | 3.16E-16 | TIP | AGRN |
| MLEC | 1.33E-20 | 0.498241352 | 0.77 | 0.442 | 4.14E-16 | TIP | MLEC |
| NAV1 | 1.55E-20 | 0.464109728 | 0.475 | 0.198 | 4.82E-16 | TIP | NAV1 |
| PREX1 | 1.74E-20 | 0.355038643 | 0.428 | 0.151 | 5.43E-16 | TIP | PREX1 |
| MMP2 | 1.76E-20 | 0.50809316 | 0.806 | 0.462 | 5.47E-16 | TIP | MMP2 |
| CTHRC1 | 2.03E-20 | 0.536937017 | 0.752 | 0.428 | 6.30E-16 | TIP | CTHRC1 |
| GNG11 | 2.37E-20 | 0.459555987 | 0.982 | 0.914 | 7.37E-16 | TIP | GNG11 |
| FLNA | 5.30E-20 | 0.490171305 | 0.878 | 0.563 | 1.65E-15 | TIP | FLNA |
| HOMER3 | 6.50E-20 | 0.384096489 | 0.406 | 0.149 | 2.02E-15 | TIP | HOMER3 |
| FHOD1 | 7.88E-20 | 0.398836212 | 0.396 | 0.138 | 2.45E-15 | TIP | FHOD1 |
| TMSB4X | 9.02E-20 | 0.54424654 | 1 | 1 | 2.81E-15 | TIP | TMSB4X |
| RHBDF2 | 1.13E-19 | 0.259242252 | 0.295 | 0.081 | 3.51E-15 | TIP | RHBDF2 |
| UACA | 1.20E-19 | 0.409459436 | 0.716 | 0.373 | 3.73E-15 | TIP | UACA |
| BGN | 2.29E-19 | 0.515453052 | 0.673 | 0.347 | 7.12E-15 | TIP | BGN |
| CFAP20 | 2.97E-19 | 0.426465639 | 0.647 | 0.335 | 9.25E-15 | TIP | CFAP20 |
| FLT4 | 3.27E-19 | 0.30829274 | 0.403 | 0.145 | 1.02E-14 | TIP | FLT4 |
| EIF2B2 | 3.72E-19 | 0.40481117 | 0.385 | 0.143 | 1.16E-14 | TIP | EIF2B2 |
| TMOD2 | 3.73E-19 | 0.277954502 | 0.317 | 0.093 | 1.16E-14 | TIP | TMOD2 |
| HMOX2 | 4.40E-19 | 0.314500777 | 0.658 | 0.329 | 1.37E-14 | TIP | HMOX2 |
| FMNL3 | 5.89E-19 | 0.369087096 | 0.558 | 0.253 | 1.83E-14 | TIP | FMNL3 |
| PLEKHO1 | 7.91E-19 | 0.395677531 | 0.579 | 0.274 | 2.46E-14 | TIP | PLEKHO1 |
| VASP | 8.87E-19 | 0.450825284 | 0.727 | 0.415 | 2.76E-14 | TIP | VASP |
| TMED9 | 9.28E-19 | 0.468835852 | 0.853 | 0.556 | 2.89E-14 | TIP | TMED9 |
| TGFB1I1 | 1.16E-18 | 0.420090874 | 0.568 | 0.277 | 3.62E-14 | TIP | TGFB1I1 |
| ITPKB | 1.31E-18 | 0.301878504 | 0.363 | 0.125 | 4.09E-14 | TIP | ITPKB |
| ST3GAL6 | 1.41E-18 | 0.312927535 | 0.237 | 0.057 | 4.38E-14 | TIP | ST3GAL6 |
| OAF | 1.85E-18 | 0.453023578 | 0.629 | 0.326 | 5.76E-14 | TIP | OAF |
| LOX | 1.96E-18 | 0.426670559 | 0.277 | 0.08 | 6.10E-14 | TIP | LOX |
| MARCKSL1 | 2.58E-18 | 0.527849021 | 0.932 | 0.763 | 8.04E-14 | TIP | MARCKSL1 |
| DOCK6 | 2.73E-18 | 0.490288278 | 0.68 | 0.373 | 8.50E-14 | TIP | DOCK6 |
| PALD1 | 2.89E-18 | 0.271454274 | 0.356 | 0.124 | 8.98E-14 | TIP | PALD1 |
| KCNE3 | 3.13E-18 | 0.379426788 | 0.18 | 0.032 | 9.75E-14 | TIP | KCNE3 |
| ADAMTSL2 | 3.25E-18 | 0.336473624 | 0.263 | 0.07 | 1.01E-13 | TIP | ADAMTSL2 |
| ISG20 | 3.44E-18 | 0.423459125 | 0.511 | 0.225 | 1.07E-13 | TIP | ISG20 |
| MYL6 | 4.15E-18 | 0.400581279 | 0.993 | 0.973 | 1.29E-13 | TIP | MYL6 |
| HTRA1 | 4.70E-18 | 0.642249045 | 0.878 | 0.659 | 1.46E-13 | TIP | HTRA1 |
| BDKRB2 | 5.96E-18 | 0.405138485 | 0.41 | 0.16 | 1.86E-13 | TIP | BDKRB2 |
| PLP2 | 6.12E-18 | 0.539703198 | 0.601 | 0.333 | 1.91E-13 | TIP | PLP2 |
| FAM129A | 6.28E-18 | 0.295266886 | 0.212 | 0.047 | 1.95E-13 | TIP | FAM129A |
| CETP | 6.90E-18 | 0.279268196 | 0.237 | 0.06 | 2.15E-13 | TIP | CETP |
| SMTN | 8.69E-18 | 0.369974476 | 0.468 | 0.198 | 2.71E-13 | TIP | SMTN |
| MYDGF | 8.75E-18 | 0.428614078 | 0.867 | 0.575 | 2.72E-13 | TIP | MYDGF |
| ICAM2 | 1.17E-17 | 0.409844656 | 0.914 | 0.608 | 3.64E-13 | TIP | ICAM2 |
| DGKH | 1.17E-17 | 0.32915046 | 0.496 | 0.216 | 3.64E-13 | TIP | DGKH |
| HECW2 | 1.21E-17 | 0.468641058 | 0.532 | 0.247 | 3.78E-13 | TIP | HECW2 |
| MYO6 | 1.21E-17 | 0.376748159 | 0.579 | 0.274 | 3.78E-13 | TIP | MYO6 |
| NT5DC2 | 1.50E-17 | 0.264094897 | 0.248 | 0.065 | 4.67E-13 | TIP | NT5DC2 |
| ACE | 1.69E-17 | 0.351101345 | 0.439 | 0.177 | 5.27E-13 | TIP | ACE |
| BCL6B | 1.73E-17 | 0.383279946 | 0.601 | 0.307 | 5.38E-13 | TIP | BCL6B |
| BID | 2.06E-17 | 0.340302663 | 0.536 | 0.245 | 6.40E-13 | TIP | BID |
| MSN | 2.52E-17 | 0.487697917 | 0.917 | 0.748 | 7.84E-13 | TIP | MSN |
| LY6H | 2.54E-17 | 0.738592385 | 0.122 | 0.013 | 7.91E-13 | TIP | LY6H |
| KITLG | 2.55E-17 | 0.268695329 | 0.313 | 0.101 | 7.94E-13 | TIP | KITLG |
| TAX1BP3 | 2.88E-17 | 0.475155323 | 0.827 | 0.571 | 8.96E-13 | TIP | TAX1BP3 |
| GPX8 | 3.59E-17 | 0.294798967 | 0.374 | 0.136 | 1.12E-12 | TIP | GPX8 |
| SLC16A3 | 4.46E-17 | 0.308544507 | 0.579 | 0.27 | 1.39E-12 | TIP | SLC16A3 |
| MYO10 | 4.85E-17 | 0.308631303 | 0.612 | 0.304 | 1.51E-12 | TIP | MYO10 |
| RALA | 5.72E-17 | 0.469380823 | 0.647 | 0.358 | 1.78E-12 | TIP | RALA |
| ADGRL2 | 7.64E-17 | 0.385035642 | 0.496 | 0.23 | 2.38E-12 | TIP | ADGRL2 |
| SGCB | 8.79E-17 | 0.256126197 | 0.406 | 0.161 | 2.74E-12 | TIP | SGCB |
| HAGLROS | 8.85E-17 | 0.320385018 | 0.385 | 0.148 | 2.75E-12 | TIP | HAGLROS |
| SIPA1 | 9.05E-17 | 0.257855731 | 0.421 | 0.171 | 2.82E-12 | TIP | SIPA1 |
| DOCK8 | 1.12E-16 | 0.257579226 | 0.201 | 0.046 | 3.48E-12 | TIP | DOCK8 |
| EXOC3L1 | 1.18E-16 | 0.308407821 | 0.371 | 0.14 | 3.67E-12 | TIP | EXOC3L1 |
| MYL12A | 1.21E-16 | 0.435368657 | 0.982 | 0.917 | 3.75E-12 | TIP | MYL12A |
| KIAA1462 | 1.49E-16 | 0.372821142 | 0.518 | 0.252 | 4.64E-12 | TIP | KIAA1462 |
| TMEM132A | 1.49E-16 | 0.260874621 | 0.281 | 0.088 | 4.65E-12 | TIP | TMEM132A |
| GPX1 | 1.50E-16 | 0.466339746 | 0.928 | 0.787 | 4.67E-12 | TIP | GPX1 |
| MPZL1 | 1.55E-16 | 0.316999518 | 0.572 | 0.282 | 4.82E-12 | TIP | MPZL1 |
| CD63 | 1.98E-16 | 0.418111404 | 0.975 | 0.918 | 6.16E-12 | TIP | CD63 |
| C11orf96 | 2.05E-16 | 0.289902073 | 0.45 | 0.19 | 6.37E-12 | TIP | C11orf96 |
| CD40 | 2.85E-16 | 0.392211485 | 0.583 | 0.295 | 8.87E-12 | TIP | CD40 |
| TAGLN | 3.70E-16 | 1.171096597 | 0.424 | 0.187 | 1.15E-11 | TIP | TAGLN |
| ADRM1 | 3.94E-16 | 0.387109994 | 0.734 | 0.452 | 1.23E-11 | TIP | ADRM1 |
| TWF2 | 4.55E-16 | 0.309541587 | 0.597 | 0.308 | 1.42E-11 | TIP | TWF2 |
| SEC14L1 | 4.87E-16 | 0.416118555 | 0.809 | 0.507 | 1.51E-11 | TIP | SEC14L1 |
| REG4 | 5.54E-16 | 0.293689685 | 0.216 | 0.055 | 1.72E-11 | TIP | REG4 |
| C1orf54 | 6.26E-16 | 0.454716322 | 0.906 | 0.666 | 1.95E-11 | TIP | C1orf54 |
| DAPK3 | 6.61E-16 | 0.363213361 | 0.612 | 0.329 | 2.06E-11 | TIP | DAPK3 |
| SEC13 | 6.69E-16 | 0.284249916 | 0.576 | 0.28 | 2.08E-11 | TIP | SEC13 |
| COL13A1 | 7.00E-16 | 0.296412067 | 0.273 | 0.086 | 2.18E-11 | TIP | COL13A1 |
| MMP1 | 7.64E-16 | 0.406610907 | 0.263 | 0.08 | 2.38E-11 | TIP | MMP1 |
| ADM | 1.03E-15 | 0.694593173 | 0.478 | 0.232 | 3.22E-11 | TIP | ADM |
| MCF2L | 1.15E-15 | 0.307107416 | 0.518 | 0.244 | 3.57E-11 | TIP | MCF2L |
| GRAP | 2.10E-15 | 0.276901456 | 0.331 | 0.12 | 6.55E-11 | TIP | GRAP |
| CD93 | 2.31E-15 | 0.449987065 | 0.957 | 0.769 | 7.19E-11 | TIP | CD93 |
| CTNNBIP1 | 2.61E-15 | 0.337591618 | 0.439 | 0.19 | 8.11E-11 | TIP | CTNNBIP1 |
| SPG7 | 3.51E-15 | 0.385692311 | 0.543 | 0.269 | 1.09E-10 | TIP | SPG7 |
| SH2B3 | 3.89E-15 | 0.329182716 | 0.64 | 0.338 | 1.21E-10 | TIP | SH2B3 |
| PFKP | 4.17E-15 | 0.331512879 | 0.432 | 0.193 | 1.30E-10 | TIP | PFKP |
| TTYH3 | 5.10E-15 | 0.395188946 | 0.507 | 0.251 | 1.59E-10 | TIP | TTYH3 |
| RAPGEF1 | 6.32E-15 | 0.312272655 | 0.457 | 0.208 | 1.97E-10 | TIP | RAPGEF1 |
| GADD45G | 8.23E-15 | 0.286818555 | 0.353 | 0.138 | 2.56E-10 | TIP | GADD45G |
| PLS3 | 8.79E-15 | 0.36305363 | 0.723 | 0.421 | 2.74E-10 | TIP | PLS3 |
| EDIL3 | 9.26E-15 | 0.260643177 | 0.288 | 0.097 | 2.88E-10 | TIP | EDIL3 |
| ACOT7 | 1.01E-14 | 0.296667549 | 0.475 | 0.226 | 3.14E-10 | TIP | ACOT7 |
| P3H4 | 1.14E-14 | 0.253155423 | 0.32 | 0.116 | 3.54E-10 | TIP | P3H4 |
| COL6A2 | 1.25E-14 | 0.373570111 | 0.799 | 0.486 | 3.88E-10 | TIP | COL6A2 |
| SEMA6B | 1.34E-14 | 0.268217546 | 0.568 | 0.286 | 4.18E-10 | TIP | SEMA6B |
| SLC12A2 | 1.79E-14 | 0.292094253 | 0.457 | 0.213 | 5.58E-10 | TIP | SLC12A2 |
| MRPL43 | 1.87E-14 | 0.267511014 | 0.579 | 0.294 | 5.82E-10 | TIP | MRPL43 |
| DCHS1 | 1.95E-14 | 0.310517103 | 0.5 | 0.242 | 6.06E-10 | TIP | DCHS1 |
| F2R | 1.99E-14 | 0.46965669 | 0.68 | 0.4 | 6.18E-10 | TIP | F2R |
| SIPA1L2 | 2.65E-14 | 0.268057091 | 0.381 | 0.156 | 8.23E-10 | TIP | SIPA1L2 |
| CXCL12 | 3.09E-14 | 0.492961991 | 0.496 | 0.238 | 9.61E-10 | TIP | CXCL12 |
| LAPTM4B | 3.12E-14 | 0.273418027 | 0.701 | 0.388 | 9.70E-10 | TIP | LAPTM4B |
| PSMB5 | 3.20E-14 | 0.366231052 | 0.845 | 0.586 | 9.97E-10 | TIP | PSMB5 |
| FYN | 3.34E-14 | 0.278711278 | 0.626 | 0.338 | 1.04E-09 | TIP | FYN |
| MAP4K4 | 3.56E-14 | 0.42308579 | 0.698 | 0.419 | 1.11E-09 | TIP | MAP4K4 |
| PNP | 3.56E-14 | 0.421232905 | 0.784 | 0.504 | 1.11E-09 | TIP | PNP |
| PSMD13 | 3.85E-14 | 0.280789038 | 0.655 | 0.371 | 1.20E-09 | TIP | PSMD13 |
| DTNBP1 | 4.58E-14 | 0.261277563 | 0.335 | 0.133 | 1.42E-09 | TIP | DTNBP1 |
| NGFRAP1 | 4.71E-14 | 0.375817577 | 0.888 | 0.668 | 1.47E-09 | TIP | NGFRAP1 |
| SOX18 | 4.95E-14 | 0.319994839 | 0.73 | 0.432 | 1.54E-09 | TIP | SOX18 |
| GNAS | 5.10E-14 | 0.37234929 | 0.989 | 0.783 | 1.59E-09 | TIP | GNAS |
| RASGRP3 | 5.55E-14 | 0.375262092 | 0.658 | 0.373 | 1.73E-09 | TIP | RASGRP3 |
| FDPS | 6.91E-14 | 0.320994015 | 0.813 | 0.53 | 2.15E-09 | TIP | FDPS |
| TMEM184B | 7.03E-14 | 0.29136804 | 0.529 | 0.266 | 2.19E-09 | TIP | TMEM184B |
| CREG1 | 7.75E-14 | 0.285490875 | 0.363 | 0.154 | 2.41E-09 | TIP | CREG1 |
| ITGA1 | 8.00E-14 | 0.423040597 | 0.522 | 0.266 | 2.49E-09 | TIP | ITGA1 |
| TEX264 | 8.24E-14 | 0.309914982 | 0.633 | 0.355 | 2.56E-09 | TIP | TEX264 |
| FRMD8 | 1.06E-13 | 0.266024148 | 0.414 | 0.183 | 3.29E-09 | TIP | FRMD8 |
| BCAP31 | 1.20E-13 | 0.374233657 | 0.856 | 0.598 | 3.72E-09 | TIP | BCAP31 |
| RNF19A | 1.83E-13 | 0.333840006 | 0.536 | 0.292 | 5.68E-09 | TIP | RNF19A |
| WDR1 | 2.11E-13 | 0.381825984 | 0.755 | 0.526 | 6.57E-09 | TIP | WDR1 |
| TM2D2 | 2.66E-13 | 0.25270849 | 0.489 | 0.234 | 8.29E-09 | TIP | TM2D2 |
| PRDM1 | 2.71E-13 | 0.305552961 | 0.5 | 0.241 | 8.44E-09 | TIP | PRDM1 |
| GNAI1 | 2.92E-13 | 0.272733497 | 0.335 | 0.136 | 9.08E-09 | TIP | GNAI1 |
| LINC01235 | 3.39E-13 | 0.438032993 | 0.482 | 0.251 | 1.05E-08 | TIP | LINC01235 |
| IGF2 | 4.31E-13 | 0.872290029 | 0.532 | 0.292 | 1.34E-08 | TIP | IGF2 |
| ETS1 | 4.71E-13 | 0.393068535 | 0.827 | 0.583 | 1.47E-08 | TIP | ETS1 |
| UBE2J1 | 4.76E-13 | 0.407709721 | 0.712 | 0.462 | 1.48E-08 | TIP | UBE2J1 |
| UBALD2 | 4.86E-13 | 0.382124373 | 0.576 | 0.319 | 1.51E-08 | TIP | UBALD2 |
| HERC1 | 5.20E-13 | 0.253898488 | 0.446 | 0.208 | 1.62E-08 | TIP | HERC1 |
| PPM1F | 5.56E-13 | 0.337796521 | 0.604 | 0.331 | 1.73E-08 | TIP | PPM1F |
| GALNT18 | 5.66E-13 | 0.341895361 | 0.496 | 0.254 | 1.76E-08 | TIP | GALNT18 |
| NDUFA6 | 7.09E-13 | 0.317449508 | 0.719 | 0.446 | 2.21E-08 | TIP | NDUFA6 |
| C7orf73 | 8.57E-13 | 0.259658474 | 0.716 | 0.426 | 2.67E-08 | TIP | C7orf73 |
| GJA1 | 1.27E-12 | 0.363345066 | 0.777 | 0.482 | 3.95E-08 | TIP | GJA1 |
| C10orf54 | 1.45E-12 | 0.394261131 | 0.629 | 0.364 | 4.50E-08 | TIP | C10orf54 |
| APLNR1 | 1.50E-12 | 0.392515493 | 0.773 | 0.492 | 4.66E-08 | TIP | APLNR |
| ALDOA | 1.57E-12 | 0.363405911 | 0.939 | 0.763 | 4.87E-08 | TIP | ALDOA |
| CYBA | 1.71E-12 | 0.385010268 | 0.784 | 0.53 | 5.31E-08 | TIP | CYBA |
| DPYSL3 | 1.94E-12 | 0.317632163 | 0.676 | 0.391 | 6.04E-08 | TIP | DPYSL3 |
| ADD3 | 2.03E-12 | 0.266499112 | 0.446 | 0.209 | 6.32E-08 | TIP | ADD3 |
| UBTD1 | 2.14E-12 | 0.284895304 | 0.504 | 0.262 | 6.65E-08 | TIP | UBTD1 |
| SPRY4-IT1 | 2.16E-12 | 0.25363789 | 0.241 | 0.084 | 6.73E-08 | TIP | SPRY4-IT1 |
| ODC1 | 2.37E-12 | 0.458575314 | 0.457 | 0.234 | 7.37E-08 | TIP | ODC1 |
| EHD4 | 2.37E-12 | 0.291136019 | 0.813 | 0.532 | 7.38E-08 | TIP | EHD4 |
| JAM3 | 2.52E-12 | 0.252903097 | 0.378 | 0.17 | 7.84E-08 | TIP | JAM3 |
| RHOC | 3.02E-12 | 0.31254451 | 0.942 | 0.822 | 9.38E-08 | TIP | RHOC |
| SEC61A1 | 3.02E-12 | 0.323851624 | 0.601 | 0.348 | 9.39E-08 | TIP | SEC61A1 |
| NME4 | 3.13E-12 | 0.331509673 | 0.622 | 0.368 | 9.74E-08 | TIP | NME4 |
| GAPDH | 3.20E-12 | 0.36203206 | 1 | 0.957 | 9.96E-08 | TIP | GAPDH |
| SSR2 | 3.26E-12 | 0.337221809 | 0.871 | 0.627 | 1.01E-07 | TIP | SSR2 |
| P3H1 | 3.37E-12 | 0.259975604 | 0.388 | 0.176 | 1.05E-07 | TIP | P3H1 |
| ATP6V0B | 4.04E-12 | 0.28050934 | 0.788 | 0.526 | 1.26E-07 | TIP | ATP6V0B |
| INHBB | 4.76E-12 | 0.251431999 | 0.338 | 0.145 | 1.48E-07 | TIP | INHBB |
| NOTCH1 | 5.11E-12 | 0.265817623 | 0.532 | 0.287 | 1.59E-07 | TIP | NOTCH1 |
| PLAUR | 5.22E-12 | 0.329085979 | 0.446 | 0.222 | 1.62E-07 | TIP | PLAUR |
| PDCL | 5.38E-12 | 0.286115973 | 0.432 | 0.209 | 1.67E-07 | TIP | PDCL |
| SDF4 | 6.55E-12 | 0.325517407 | 0.745 | 0.487 | 2.04E-07 | TIP | SDF4 |
| VCP | 8.45E-12 | 0.353983878 | 0.719 | 0.462 | 2.63E-07 | TIP | VCP |
| PKIG | 8.53E-12 | 0.316170222 | 0.809 | 0.541 | 2.65E-07 | TIP | PKIG |
| NPTX2 | 8.61E-12 | 0.260155677 | 0.119 | 0.023 | 2.68E-07 | TIP | NPTX2 |
| ATP6AP1 | 8.84E-12 | 0.308046086 | 0.622 | 0.37 | 2.75E-07 | TIP | ATP6AP1 |
| PPP1CA | 1.07E-11 | 0.294213881 | 0.766 | 0.493 | 3.32E-07 | TIP | PPP1CA |
| CSGALNACT1 | 1.16E-11 | 0.262284494 | 0.507 | 0.262 | 3.61E-07 | TIP | CSGALNACT1 |
| C11orf31 | 1.26E-11 | 0.323506875 | 0.849 | 0.637 | 3.92E-07 | TIP | C11orf31 |
| ATP5A1 | 1.42E-11 | 0.319772079 | 0.784 | 0.525 | 4.42E-07 | TIP | ATP5A1 |
| MAP1B | 1.60E-11 | 0.260209261 | 0.73 | 0.461 | 4.97E-07 | TIP | MAP1B |
| SPRED1 | 1.64E-11 | 0.267047785 | 0.295 | 0.123 | 5.10E-07 | TIP | SPRED1 |
| RRBP1 | 1.65E-11 | 0.345912724 | 0.827 | 0.555 | 5.15E-07 | TIP | RRBP1 |
| PRKD2 | 1.76E-11 | 0.264996141 | 0.518 | 0.279 | 5.49E-07 | TIP | PRKD2 |
| FJX1 | 1.80E-11 | 0.314393399 | 0.234 | 0.085 | 5.60E-07 | TIP | FJX1 |
| GRAMD1A | 2.38E-11 | 0.321872497 | 0.716 | 0.444 | 7.41E-07 | TIP | GRAMD1A |
| IGF2R | 2.65E-11 | 0.276859354 | 0.367 | 0.171 | 8.23E-07 | TIP | IGF2R |
| MPG | 2.81E-11 | 0.296825802 | 0.655 | 0.393 | 8.75E-07 | TIP | MPG |
| KIAA1147 | 2.98E-11 | 0.336426886 | 0.428 | 0.214 | 9.28E-07 | TIP | KIAA1147 |
| HLA-A | 3.17E-11 | 0.389826222 | 0.996 | 0.969 | 9.86E-07 | TIP | HLA-A |
| P4HB | 3.42E-11 | 0.400981954 | 0.824 | 0.623 | 1.07E-06 | TIP | P4HB |
| TMEM255B | 3.43E-11 | 0.28218101 | 0.863 | 0.598 | 1.07E-06 | TIP | TMEM255B |
| SLC4A7 | 4.16E-11 | 0.259981923 | 0.453 | 0.229 | 1.30E-06 | TIP | SLC4A7 |
| CD200 | 4.32E-11 | 0.297532521 | 0.568 | 0.326 | 1.35E-06 | TIP | CD200 |
| KRT8 | 5.05E-11 | 0.346962009 | 0.637 | 0.365 | 1.57E-06 | TIP | KRT8 |
| ARHGAP29 | 5.13E-11 | 0.272157142 | 0.928 | 0.665 | 1.60E-06 | TIP | ARHGAP29 |
| PLPP2 | 5.29E-11 | 0.291502785 | 0.291 | 0.125 | 1.65E-06 | TIP | PLPP2 |
| MEF2D | 5.70E-11 | 0.262064943 | 0.374 | 0.175 | 1.77E-06 | TIP | MEF2D |
| MRPL13 | 5.83E-11 | 0.253018988 | 0.464 | 0.242 | 1.81E-06 | TIP | MRPL13 |
| PINK1 | 6.12E-11 | 0.408075981 | 0.504 | 0.282 | 1.90E-06 | TIP | PINK1 |
| ATPIF1 | 6.62E-11 | 0.328711707 | 0.867 | 0.623 | 2.06E-06 | TIP | ATPIF1 |
| MYL12B | 7.91E-11 | 0.277200715 | 0.978 | 0.908 | 2.46E-06 | TIP | MYL12B |
| SHANK3 | 8.38E-11 | 0.283498166 | 0.439 | 0.224 | 2.61E-06 | TIP | SHANK3 |
| FADS3 | 9.42E-11 | 0.256098151 | 0.41 | 0.201 | 2.93E-06 | TIP | FADS3 |
| PRKCDBP | 9.77E-11 | 0.320461502 | 0.917 | 0.728 | 3.04E-06 | TIP | PRKCDBP |
| PON2 | 1.01E-10 | 0.273327713 | 0.719 | 0.444 | 3.15E-06 | TIP | PON2 |
| COMT | 1.05E-10 | 0.269883087 | 0.716 | 0.463 | 3.27E-06 | TIP | COMT |
| YWHAZ | 1.22E-10 | 0.292385453 | 0.881 | 0.634 | 3.80E-06 | TIP | YWHAZ |
| MARCKS | 1.25E-10 | 0.319968194 | 0.863 | 0.616 | 3.88E-06 | TIP | MARCKS |
| BSG | 1.27E-10 | 0.298194849 | 0.903 | 0.685 | 3.95E-06 | TIP | BSG |
| ENO1 | 1.28E-10 | 0.329214857 | 0.921 | 0.741 | 3.99E-06 | TIP | ENO1 |
| SCARF1 | 1.45E-10 | 0.310027578 | 0.54 | 0.312 | 4.52E-06 | TIP | SCARF1 |
| BACE2 | 1.58E-10 | 0.351973294 | 0.662 | 0.432 | 4.91E-06 | TIP | BACE2 |
| YIF1A | 1.58E-10 | 0.28383586 | 0.59 | 0.353 | 4.91E-06 | TIP | YIF1A |
| MEF2C | 1.86E-10 | 0.265784002 | 0.827 | 0.568 | 5.78E-06 | TIP | MEF2C |
| MTSS1 | 2.31E-10 | 0.279704862 | 0.306 | 0.141 | 7.18E-06 | TIP | MTSS1 |
| ARPC3 | 2.31E-10 | 0.323767861 | 0.885 | 0.725 | 7.19E-06 | TIP | ARPC3 |
| ITGA5 | 2.51E-10 | 0.316607786 | 0.885 | 0.664 | 7.82E-06 | TIP | ITGA5 |
| HDAC1 | 2.68E-10 | 0.250279112 | 0.647 | 0.394 | 8.34E-06 | TIP | HDAC1 |
| APCDD1 | 2.79E-10 | 0.285718663 | 0.212 | 0.077 | 8.69E-06 | TIP | APCDD1 |
| ADAMTS5 | 3.22E-10 | 0.533562003 | 0.277 | 0.12 | 1.00E-05 | TIP | ADAMTS5 |
| GANAB | 3.27E-10 | 0.281306536 | 0.565 | 0.32 | 1.02E-05 | TIP | GANAB |
| JAK1 | 3.76E-10 | 0.308329067 | 0.799 | 0.555 | 1.17E-05 | TIP | JAK1 |
| ADAMTS4 | 3.86E-10 | 0.277328029 | 0.486 | 0.27 | 1.20E-05 | TIP | ADAMTS4 |
| TBXA2R | 5.20E-10 | 0.260028393 | 0.306 | 0.135 | 1.62E-05 | TIP | TBXA2R |
| MGST3 | 7.39E-10 | 0.414253581 | 0.856 | 0.693 | 2.30E-05 | TIP | MGST3 |
| TMEM2 | 7.75E-10 | 0.318552685 | 0.64 | 0.408 | 2.41E-05 | TIP | TMEM2 |
| YWHAQ | 8.46E-10 | 0.306489148 | 0.827 | 0.626 | 2.63E-05 | TIP | YWHAQ |
| COX5A | 8.84E-10 | 0.285824703 | 0.817 | 0.613 | 2.75E-05 | TIP | COX5A |
| HLA-C | 9.47E-10 | 0.333286093 | 0.993 | 0.938 | 2.95E-05 | TIP | HLA-C |
| ARPC5 | 1.08E-09 | 0.291947669 | 0.827 | 0.619 | 3.36E-05 | TIP | ARPC5 |
| SHISA5 | 1.17E-09 | 0.275761295 | 0.655 | 0.41 | 3.65E-05 | TIP | SHISA5 |
| POMP | 1.29E-09 | 0.284758259 | 0.957 | 0.835 | 4.01E-05 | TIP | POMP |
| GNAI2 | 1.52E-09 | 0.288448457 | 0.917 | 0.766 | 4.73E-05 | TIP | GNAI2 |
| ARF1 | 1.54E-09 | 0.280435206 | 0.888 | 0.683 | 4.80E-05 | TIP | ARF1 |
| MANF | 1.61E-09 | 0.27807114 | 0.698 | 0.464 | 5.01E-05 | TIP | MANF |
| SSR3 | 1.65E-09 | 0.322216774 | 0.734 | 0.529 | 5.12E-05 | TIP | SSR3 |
| IER3IP1 | 1.73E-09 | 0.250785283 | 0.626 | 0.392 | 5.39E-05 | TIP | IER3IP1 |
| DYNLT1 | 1.87E-09 | 0.32617958 | 0.784 | 0.583 | 5.82E-05 | TIP | DYNLT1 |
| CTSB | 2.44E-09 | 0.293007019 | 0.813 | 0.585 | 7.59E-05 | TIP | CTSB |
| PGAM1 | 2.54E-09 | 0.250215238 | 0.824 | 0.576 | 7.92E-05 | TIP | PGAM1 |
| MYADM | 2.65E-09 | 0.401257006 | 0.698 | 0.484 | 8.25E-05 | TIP | MYADM |
| PPIB | 2.68E-09 | 0.354351404 | 0.928 | 0.761 | 8.33E-05 | TIP | PPIB |
| FILIP1 | 3.26E-09 | 0.250518958 | 0.518 | 0.295 | 0.000101589 | TIP | FILIP1 |
| PRELID1 | 3.52E-09 | 0.285894314 | 0.827 | 0.631 | 0.000109494 | TIP | PRELID1 |
| ATP5C1 | 3.53E-09 | 0.26111322 | 0.777 | 0.583 | 0.00010989 | TIP | ATP5C1 |
| PLK2 | 4.28E-09 | 0.390155337 | 0.637 | 0.415 | 0.000133027 | TIP | PLK2 |
| TPPP3 | 4.40E-09 | 0.285233004 | 0.194 | 0.072 | 0.00013703 | TIP | TPPP3 |
| TUFM | 4.49E-09 | 0.277700149 | 0.737 | 0.505 | 0.000139625 | TIP | TUFM |
| SCAND1 | 4.51E-09 | 0.255086524 | 0.842 | 0.614 | 0.000140266 | TIP | SCAND1 |
| RNF7 | 4.64E-09 | 0.287808196 | 0.773 | 0.578 | 0.000144522 | TIP | RNF7 |
| HSPA8 | 5.03E-09 | 0.283868691 | 0.928 | 0.792 | 0.000156406 | TIP | HSPA8 |
| AP2M1 | 5.44E-09 | 0.303731649 | 0.827 | 0.63 | 0.00016929 | TIP | AP2M1 |
| ITPRIP | 5.81E-09 | 0.278540861 | 0.54 | 0.325 | 0.000180767 | TIP | ITPRIP |
| RABAC1 | 5.90E-09 | 0.265314459 | 0.917 | 0.757 | 0.00018354 | TIP | RABAC1 |
| RAB5C | 8.08E-09 | 0.251499629 | 0.806 | 0.582 | 0.000251308 | TIP | RAB5C |
| ACTR3 | 9.09E-09 | 0.264027501 | 0.741 | 0.526 | 0.000282864 | TIP | ACTR3 |
| CD81 | 1.08E-08 | 0.33686438 | 0.914 | 0.738 | 0.000336522 | TIP | CD81 |
| SEMA3F | 1.44E-08 | 0.360135921 | 0.597 | 0.369 | 0.000448827 | TIP | SEMA3F |
| H2AFZ | 1.68E-08 | 0.289459051 | 0.903 | 0.77 | 0.000521874 | TIP | H2AFZ |
| PSMA7 | 1.97E-08 | 0.278727859 | 0.942 | 0.807 | 0.000611639 | TIP | PSMA7 |
| DAD1 | 2.48E-08 | 0.295576724 | 0.878 | 0.71 | 0.000770665 | TIP | DAD1 |
| MTDH | 2.86E-08 | 0.2676582 | 0.849 | 0.642 | 0.000890597 | TIP | MTDH |
| TGFB1 | 3.06E-08 | 0.291812964 | 0.658 | 0.434 | 0.000951424 | TIP | TGFB1 |
| HSBP1 | 3.07E-08 | 0.303679143 | 0.856 | 0.659 | 0.000955618 | TIP | HSBP1 |
| TPM4 | 3.34E-08 | 0.287459443 | 0.935 | 0.82 | 0.00103997 | TIP | TPM4 |
| PRSS23 | 3.85E-08 | 0.33789461 | 0.892 | 0.72 | 0.001199048 | TIP | PRSS23 |
| ARPC2 | 3.85E-08 | 0.261344102 | 0.924 | 0.785 | 0.001199507 | TIP | ARPC2 |
| MGLL | 4.54E-08 | 0.296450862 | 0.691 | 0.474 | 0.001411169 | TIP | MGLL |
| APOD | 4.68E-08 | 0.607253493 | 0.162 | 0.06 | 0.00145544 | TIP | APOD |
| LTBP1 | 5.05E-08 | 0.252562875 | 0.338 | 0.178 | 0.001570213 | TIP | LTBP1 |
| CDC37 | 5.50E-08 | 0.280047874 | 0.903 | 0.716 | 0.001712467 | TIP | CDC37 |
| CTNNB1 | 5.79E-08 | 0.310547991 | 0.867 | 0.658 | 0.001802161 | TIP | CTNNB1 |
| APP | 7.49E-08 | 0.312363156 | 0.975 | 0.826 | 0.002331204 | TIP | APP |
| FSTL1 | 7.79E-08 | 0.303478782 | 0.809 | 0.571 | 0.002425053 | TIP | FSTL1 |
| MYH9 | 7.92E-08 | 0.274176524 | 0.896 | 0.741 | 0.002463765 | TIP | MYH9 |
| SEC61G | 9.45E-08 | 0.253497499 | 0.935 | 0.801 | 0.002939169 | TIP | SEC61G |
| SNRPB | 1.03E-07 | 0.269355957 | 0.845 | 0.66 | 0.003216128 | TIP | SNRPB |
| CLEC14A | 1.09E-07 | 0.346650022 | 0.906 | 0.695 | 0.003379802 | TIP | CLEC14A |
| PDGFD | 1.14E-07 | 0.253918306 | 0.219 | 0.095 | 0.003549539 | TIP | PDGFD |
| ATP6V1G1 | 1.31E-07 | 0.25163844 | 0.853 | 0.674 | 0.004070838 | TIP | ATP6V1G1 |
| GPIHBP1 | 1.69E-07 | 0.256038389 | 0.144 | 0.05 | 0.005273067 | TIP | GPIHBP1 |
| PECAM1 | 1.93E-07 | 0.271967538 | 0.986 | 0.896 | 0.005996042 | TIP | PECAM1 |
| CALR | 2.02E-07 | 0.287100782 | 0.939 | 0.785 | 0.006290619 | TIP | CALR |
| ID3 | 2.42E-07 | 0.284992745 | 0.903 | 0.69 | 0.007518236 | TIP | ID3 |
| PDIA3 | 2.46E-07 | 0.309034184 | 0.842 | 0.681 | 0.007655075 | TIP | PDIA3 |
| THBS1 | 2.95E-07 | 0.282050308 | 0.586 | 0.378 | 0.009166179 | TIP | THBS1 |
| ENG | 3.64E-07 | 0.365084065 | 0.968 | 0.868 | 0.011312457 | TIP | ENG |
| PHGR1 | 6.63E-07 | 0.283043998 | 0.338 | 0.186 | 0.020624833 | TIP | PHGR1 |
| HSP90B1 | 7.21E-07 | 0.303102258 | 0.939 | 0.775 | 0.022423759 | TIP | HSP90B1 |
| HLA-B | 8.10E-07 | 0.287610644 | 1 | 0.979 | 0.025200273 | TIP | HLA-B |
| KDELR2 | 9.45E-07 | 0.264039529 | 0.755 | 0.578 | 0.029389829 | TIP | KDELR2 |
| EDNRB | 1.10E-06 | 0.326911613 | 0.165 | 0.07 | 0.034226522 | TIP | EDNRB |
| GJA4 | 2.11E-69 | 2.120217139 | 0.515 | 0.081 | 6.57E-65 | Artery | GJA4 |
| SEMA3G | 3.71E-66 | 1.122334304 | 0.365 | 0.019 | 1.15E-61 | Artery | SEMA3G |
| FBLN5 | 1.05E-65 | 1.35098978 | 0.318 | 0.006 | 3.27E-61 | Artery | FBLN5 |
| GJA5 | 2.48E-65 | 1.628337763 | 0.348 | 0.016 | 7.73E-61 | Artery | GJA5 |
| SRP14 | 3.61E-39 | 1.26656567 | 0.93 | 0.969 | 1.12E-34 | Artery | SRP14 |
| SLC9A3R2 | 6.46E-33 | 1.46753776 | 0.789 | 0.653 | 2.01E-28 | Artery | SLC9A3R2 |
| HEY1 | 7.31E-33 | 1.344003767 | 0.488 | 0.181 | 2.27E-28 | Artery | HEY1 |
| PLPP1 | 1.85E-30 | 1.451889034 | 0.656 | 0.423 | 5.75E-26 | Artery | PLPP1 |
| SRGN | 1.94E-29 | 1.274844156 | 0.793 | 0.781 | 6.04E-25 | Artery | SRGN |
| IGFBP31 | 1.59E-25 | 1.738166152 | 0.666 | 0.421 | 4.93E-21 | Artery | IGFBP3 |
| PLLP | 2.27E-25 | 0.800222504 | 0.271 | 0.061 | 7.07E-21 | Artery | PLLP |
| ARL15 | 2.35E-25 | 0.9823022 | 0.465 | 0.21 | 7.30E-21 | Artery | ARL15 |
| AIF1L | 8.30E-25 | 0.600950694 | 0.231 | 0.042 | 2.58E-20 | Artery | AIF1L |
| IFI27 | 6.21E-23 | 0.836066046 | 0.943 | 0.922 | 1.93E-18 | Artery | IFI27 |
| SULF1 | 1.32E-22 | 1.088246416 | 0.224 | 0.046 | 4.10E-18 | Artery | SULF1 |
| NUDT4 | 9.03E-21 | 1.300028496 | 0.525 | 0.325 | 2.81E-16 | Artery | NUDT4 |
| CXCL121 | 2.19E-20 | 1.306963221 | 0.475 | 0.239 | 6.81E-16 | Artery | CXCL12 |
| IGF21 | 1.18E-19 | 1.185636354 | 0.522 | 0.29 | 3.69E-15 | Artery | IGF2 |
| SLC14A1 | 1.34E-19 | 0.57440657 | 0.177 | 0.03 | 4.16E-15 | Artery | SLC14A1 |
| SLC45A4 | 1.51E-19 | 0.423136423 | 0.151 | 0.019 | 4.70E-15 | Artery | SLC45A4 |
| EFNB2 | 3.39E-19 | 1.193459581 | 0.545 | 0.372 | 1.05E-14 | Artery | EFNB2 |
| ALPL | 8.06E-18 | 0.426809548 | 0.177 | 0.034 | 2.51E-13 | Artery | ALPL |
| ATP2A3 | 9.79E-18 | 0.623864203 | 0.181 | 0.036 | 3.05E-13 | Artery | ATP2A3 |
| IFITM3 | 2.15E-17 | 0.515082312 | 0.98 | 0.99 | 6.68E-13 | Artery | IFITM3 |
| A2M | 1.86E-16 | 0.755974474 | 0.866 | 0.904 | 5.77E-12 | Artery | A2M |
| BTNL9 | 4.41E-16 | 0.672220501 | 0.151 | 0.027 | 1.37E-11 | Artery | BTNL9 |
| TSPAN2 | 5.23E-16 | 0.510387128 | 0.187 | 0.045 | 1.63E-11 | Artery | TSPAN2 |
| NOTCH41 | 5.46E-14 | 0.769592728 | 0.639 | 0.505 | 1.70E-09 | Artery | NOTCH4 |
| STMN1 | 1.05E-13 | 1.367760149 | 0.612 | 0.537 | 3.27E-09 | Artery | STMN1 |
| HES4 | 2.69E-13 | 0.988842205 | 0.405 | 0.239 | 8.37E-09 | Artery | HES4 |
| PPA1 | 3.32E-13 | 1.11261425 | 0.622 | 0.619 | 1.03E-08 | Artery | PPA1 |
| ASS1 | 7.41E-13 | 0.690605585 | 0.361 | 0.187 | 2.31E-08 | Artery | ASS1 |
| PODXL1 | 7.90E-13 | 0.87945572 | 0.692 | 0.634 | 2.46E-08 | Artery | PODXL |
| IFITM2 | 8.60E-13 | 0.47439079 | 0.926 | 0.946 | 2.68E-08 | Artery | IFITM2 |
| SLC7A11 | 1.81E-12 | 0.387107999 | 0.124 | 0.025 | 5.62E-08 | Artery | SLC7A11 |
| B2M | 3.41E-12 | 0.331529292 | 0.997 | 1 | 1.06E-07 | Artery | B2M |
| RBP7 | 5.10E-12 | 1.361701981 | 0.378 | 0.212 | 1.59E-07 | Artery | RBP7 |
| SAT1 | 6.24E-12 | 1.435740761 | 0.779 | 0.869 | 1.94E-07 | Artery | SAT1 |
| SLC6A6 | 2.67E-11 | 0.808773407 | 0.321 | 0.164 | 8.31E-07 | Artery | SLC6A6 |
| POMP1 | 3.07E-11 | 0.541479295 | 0.843 | 0.868 | 9.56E-07 | Artery | POMP |
| DLL41 | 9.31E-11 | 0.72361326 | 0.421 | 0.262 | 2.90E-06 | Artery | DLL4 |
| ARHGAP4 | 1.08E-10 | 0.51036841 | 0.271 | 0.127 | 3.35E-06 | Artery | ARHGAP4 |
| STOM | 1.40E-10 | 0.689823896 | 0.739 | 0.825 | 4.36E-06 | Artery | STOM |
| DEPTOR | 1.87E-10 | 0.33828775 | 0.144 | 0.041 | 5.81E-06 | Artery | DEPTOR |
| FLT11 | 1.88E-10 | 0.817224276 | 0.656 | 0.593 | 5.85E-06 | Artery | FLT1 |
| INSR1 | 3.24E-10 | 0.85194499 | 0.676 | 0.632 | 1.01E-05 | Artery | INSR |
| 1-Nov | 5.40E-10 | 0.374942309 | 0.171 | 0.057 | 1.68E-05 | Artery | NOV |
| PCSK5 | 5.67E-10 | 0.58681155 | 0.191 | 0.075 | 1.77E-05 | Artery | PCSK5 |
| PTP4A31 | 7.09E-10 | 0.744577032 | 0.492 | 0.359 | 2.21E-05 | Artery | PTP4A3 |
| RAMP2 | 7.93E-10 | 0.4653451 | 0.883 | 0.942 | 2.47E-05 | Artery | RAMP2 |
| CLIC5 | 8.71E-10 | 0.284564456 | 0.154 | 0.048 | 2.71E-05 | Artery | CLIC5 |
| JAM2 | 1.86E-09 | 0.859939741 | 0.565 | 0.486 | 5.80E-05 | Artery | JAM2 |
| EPAS1 | 2.55E-09 | 0.644149857 | 0.726 | 0.805 | 7.94E-05 | Artery | EPAS1 |
| ACE1 | 3.72E-09 | 0.649868402 | 0.344 | 0.201 | 0.000115602 | Artery | ACE |
| SOX17 | 6.04E-09 | 0.765452014 | 0.455 | 0.337 | 0.000187934 | Artery | SOX17 |
| PPP1R14A | 7.69E-09 | 0.507523584 | 0.117 | 0.033 | 0.000239273 | Artery | PPP1R14A |
| C10orf10 | 1.49E-08 | 1.177719016 | 0.465 | 0.345 | 0.000463597 | Artery | C10orf10 |
| ENPP2 | 2.21E-08 | 1.471392287 | 0.288 | 0.163 | 0.000686312 | Artery | ENPP2 |
| PRKCDBP1 | 3.03E-08 | 0.615319162 | 0.749 | 0.777 | 0.000942484 | Artery | PRKCDBP |
| BST2 | 3.67E-08 | 0.542421043 | 0.716 | 0.695 | 0.001142499 | Artery | BST2 |
| GSN1 | 4.26E-08 | 0.600298157 | 0.769 | 0.754 | 0.00132648 | Artery | GSN |
| SYNJ2 | 4.91E-08 | 0.402833074 | 0.284 | 0.155 | 0.001528696 | Artery | SYNJ2 |
| GABARAPL2 | 5.65E-08 | 0.587924118 | 0.719 | 0.804 | 0.001756716 | Artery | GABARAPL2 |
| MPPED2 | 6.17E-08 | 0.286334588 | 0.134 | 0.046 | 0.001921251 | Artery | MPPED2 |
| ADGRF51 | 6.48E-08 | 0.642152172 | 0.605 | 0.57 | 0.002015214 | Artery | ADGRF5 |
| MECOM | 1.59E-07 | 0.628027866 | 0.425 | 0.316 | 0.004943995 | Artery | MECOM |
| CLDN5 | 1.99E-07 | 1.063802231 | 0.689 | 0.691 | 0.006190599 | Artery | CLDN5 |
| LIMCH1 | 2.08E-07 | 0.537794043 | 0.361 | 0.236 | 0.006478392 | Artery | LIMCH1 |
| FAM124B | 2.11E-07 | 0.322572478 | 0.151 | 0.059 | 0.006572585 | Artery | FAM124B |
| VEGFC | 4.63E-07 | 0.544333944 | 0.368 | 0.249 | 0.01442181 | Artery | VEGFC |
| ATP13A3 | 5.07E-07 | 0.72036089 | 0.301 | 0.19 | 0.015789291 | Artery | ATP13A3 |
| PTPRB | 6.50E-07 | 0.626224943 | 0.592 | 0.577 | 0.020211988 | Artery | PTPRB |
| ICAM21 | 6.93E-07 | 0.654058712 | 0.659 | 0.683 | 0.021559775 | Artery | ICAM2 |
| GNG111 | 9.57E-07 | 0.345581369 | 0.91 | 0.936 | 0.029778557 | Artery | GNG11 |
| CLEC14A1 | 1.41E-06 | 0.548627939 | 0.729 | 0.747 | 0.043896686 | Artery | CLEC14A |
| S100A4 | 1.58E-06 | 1.173087427 | 0.485 | 0.399 | 0.049188375 | Artery | S100A4 |
| PDPN | 4.56E-191 | 2.688499677 | 0.862 | 0.009 | 1.42E-186 | Lymphatic | PDPN |
| CCL21 | 3.01E-176 | 8.14515394 | 0.983 | 0.026 | 9.38E-172 | Lymphatic | CCL21 |
| IGF1 | 5.10E-162 | 1.730276281 | 0.672 | 0.003 | 1.59E-157 | Lymphatic | IGF1 |
| ADGRG3 | 8.76E-147 | 1.169301069 | 0.569 | 0.001 | 2.73E-142 | Lymphatic | ADGRG3 |
| PROX1 | 4.79E-131 | 2.049388084 | 0.707 | 0.015 | 1.49E-126 | Lymphatic | PROX1 |
| RELN | 4.12E-128 | 1.087752834 | 0.483 | 0 | 1.28E-123 | Lymphatic | RELN |
| TBX1 | 1.75E-115 | 1.541183022 | 0.569 | 0.009 | 5.46E-111 | Lymphatic | TBX1 |
| PARD6G | 1.87E-103 | 1.03230933 | 0.466 | 0.004 | 5.81E-99 | Lymphatic | PARD6G |
| ACKR2 | 4.83E-87 | 0.899865552 | 0.362 | 0.002 | 1.50E-82 | Lymphatic | ACKR2 |
| SEMA3A | 2.78E-86 | 1.341757062 | 0.517 | 0.014 | 8.64E-82 | Lymphatic | SEMA3A |
| EFNA5 | 1.31E-79 | 1.181520732 | 0.397 | 0.006 | 4.06E-75 | Lymphatic | EFNA5 |
| MRC1 | 9.89E-77 | 1.091044824 | 0.397 | 0.007 | 3.08E-72 | Lymphatic | MRC1 |
| STON2 | 7.96E-74 | 0.638543628 | 0.31 | 0.002 | 2.48E-69 | Lymphatic | STON2 |
| SEMA3D | 2.52E-73 | 0.700682039 | 0.276 | 0 | 7.84E-69 | Lymphatic | SEMA3D |
| MIR99AHG | 8.80E-68 | 0.622617397 | 0.328 | 0.004 | 2.74E-63 | Lymphatic | MIR99AHG |
| EMILIN1 | 1.14E-67 | 0.945730569 | 0.431 | 0.014 | 3.56E-63 | Lymphatic | EMILIN1 |
| MAF | 3.79E-66 | 1.630614482 | 0.672 | 0.053 | 1.18E-61 | Lymphatic | MAF |
| CHRDL1 | 2.53E-55 | 0.653574622 | 0.224 | 0.001 | 7.86E-51 | Lymphatic | CHRDL1 |
| DOCK5 | 4.14E-54 | 1.101182916 | 0.397 | 0.017 | 1.29E-49 | Lymphatic | DOCK5 |
| NR2F1 | 3.51E-51 | 1.000752293 | 0.362 | 0.014 | 1.09E-46 | Lymphatic | NR2F1 |
| MPP7 | 3.96E-50 | 1.023637557 | 0.276 | 0.006 | 1.23E-45 | Lymphatic | MPP7 |
| TFPI | 2.44E-49 | 3.30801676 | 0.948 | 0.254 | 7.60E-45 | Lymphatic | TFPI |
| RAB11FIP1 | 5.18E-48 | 1.227897591 | 0.534 | 0.046 | 1.61E-43 | Lymphatic | RAB11FIP1 |
| DTX1 | 5.37E-48 | 0.387354476 | 0.224 | 0.003 | 1.67E-43 | Lymphatic | DTX1 |
| KBTBD11 | 2.66E-47 | 0.651273644 | 0.345 | 0.014 | 8.28E-43 | Lymphatic | KBTBD11 |
| COLEC12 | 1.02E-46 | 1.096435132 | 0.431 | 0.028 | 3.17E-42 | Lymphatic | COLEC12 |
| GPR182 | 2.75E-46 | 0.321898066 | 0.172 | 0 | 8.55E-42 | Lymphatic | GPR182 |
| EFEMP1 | 5.66E-46 | 2.289782657 | 0.586 | 0.061 | 1.76E-41 | Lymphatic | EFEMP1 |
| MMRN1 | 1.96E-45 | 2.295497779 | 0.741 | 0.116 | 6.11E-41 | Lymphatic | MMRN1 |
| TSPAN11 | 2.62E-43 | 0.413435826 | 0.276 | 0.009 | 8.15E-39 | Lymphatic | TSPAN11 |
| LYVE1 | 4.12E-43 | 2.159911543 | 0.707 | 0.117 | 1.28E-38 | Lymphatic | LYVE1 |
| C6orf141 | 6.14E-43 | 0.890578952 | 0.362 | 0.02 | 1.91E-38 | Lymphatic | C6orf141 |
| MEGF6 | 4.75E-42 | 0.88297771 | 0.345 | 0.018 | 1.48E-37 | Lymphatic | MEGF6 |
| RBP1 | 5.96E-42 | 2.681137614 | 0.897 | 0.263 | 1.85E-37 | Lymphatic | RBP1 |
| STAB2 | 8.42E-42 | 0.407040372 | 0.155 | 0 | 2.62E-37 | Lymphatic | STAB2 |
| KCNIP1 | 8.42E-42 | 0.389486189 | 0.155 | 0 | 2.62E-37 | Lymphatic | KCNIP1 |
| PLEKHA4 | 1.06E-41 | 0.412092231 | 0.259 | 0.008 | 3.29E-37 | Lymphatic | PLEKHA4 |
| ART4 | 2.12E-39 | 0.431052896 | 0.19 | 0.003 | 6.58E-35 | Lymphatic | ART4 |
| NR2F1-AS1 | 2.56E-39 | 0.355241531 | 0.19 | 0.003 | 7.96E-35 | Lymphatic | NR2F1-AS1 |
| GPR1 | 2.71E-38 | 0.474471674 | 0.172 | 0.002 | 8.42E-34 | Lymphatic | GPR1 |
| PGM5P2 | 2.55E-37 | 0.334386893 | 0.138 | 0 | 7.95E-33 | Lymphatic | PGM5P2 |
| LAPTM5 | 6.26E-37 | 2.090460596 | 0.914 | 0.3 | 1.95E-32 | Lymphatic | LAPTM5 |
| LRRN4CL | 1.89E-36 | 0.307817439 | 0.19 | 0.003 | 5.87E-32 | Lymphatic | LRRN4CL |
| DHODH | 6.04E-36 | 0.745476424 | 0.397 | 0.033 | 1.88E-31 | Lymphatic | DHODH |
| TFF3 | 1.63E-35 | 3.394831343 | 0.931 | 0.384 | 5.08E-31 | Lymphatic | TFF3 |
| STXBP6 | 7.72E-35 | 0.632929959 | 0.276 | 0.014 | 2.40E-30 | Lymphatic | STXBP6 |
| SLC9A9 | 2.19E-33 | 0.481754906 | 0.293 | 0.017 | 6.83E-29 | Lymphatic | SLC9A9 |
| SCN3A | 5.03E-33 | 0.50067562 | 0.138 | 0.001 | 1.57E-28 | Lymphatic | SCN3A |
| GAS7 | 5.08E-33 | 0.558484144 | 0.259 | 0.013 | 1.58E-28 | Lymphatic | GAS7 |
| RET | 7.68E-33 | 0.271881227 | 0.121 | 0 | 2.39E-28 | Lymphatic | RET |
| FAM162B | 2.12E-32 | 0.333544437 | 0.172 | 0.003 | 6.60E-28 | Lymphatic | FAM162B |
| FHL1 | 2.72E-32 | 1.116899847 | 0.586 | 0.094 | 8.47E-28 | Lymphatic | FHL1 |
| ADD31 | 3.56E-31 | 1.507622194 | 0.828 | 0.235 | 1.11E-26 | Lymphatic | ADD3 |
| MYZAP | 4.80E-31 | 0.7535691 | 0.293 | 0.02 | 1.49E-26 | Lymphatic | MYZAP |
| MFAP4 | 1.12E-30 | 1.353308239 | 0.207 | 0.008 | 3.48E-26 | Lymphatic | MFAP4 |
| IGSF3 | 4.75E-30 | 0.465707157 | 0.19 | 0.006 | 1.48E-25 | Lymphatic | IGSF3 |
| AKAP12 | 8.23E-30 | 1.596117859 | 0.638 | 0.117 | 2.56E-25 | Lymphatic | AKAP12 |
| PCSK6 | 1.71E-29 | 0.275785938 | 0.224 | 0.01 | 5.33E-25 | Lymphatic | PCSK6 |
| FAM174B | 3.92E-29 | 1.155768286 | 0.534 | 0.088 | 1.22E-24 | Lymphatic | FAM174B |
| DKK3 | 1.94E-28 | 1.023261611 | 0.431 | 0.056 | 6.04E-24 | Lymphatic | DKK3 |
| PLIN5 | 2.20E-28 | 0.640084532 | 0.155 | 0.003 | 6.84E-24 | Lymphatic | PLIN5 |
| PTX3 | 2.20E-28 | 0.369935054 | 0.155 | 0.003 | 6.84E-24 | Lymphatic | PTX3 |
| SLC38A4 | 2.30E-28 | 0.315655846 | 0.103 | 0 | 7.14E-24 | Lymphatic | SLC38A4 |
| CYB5RL | 2.79E-28 | 0.682983207 | 0.293 | 0.022 | 8.69E-24 | Lymphatic | CYB5RL |
| B3GNT7 | 3.57E-28 | 0.365906757 | 0.172 | 0.005 | 1.11E-23 | Lymphatic | B3GNT7 |
| SLCO2B1 | 1.31E-27 | 0.670443737 | 0.293 | 0.023 | 4.07E-23 | Lymphatic | SLCO2B1 |
| TNFRSF11A | 1.61E-27 | 0.5619077 | 0.207 | 0.009 | 5.02E-23 | Lymphatic | TNFRSF11A |
| TC2N | 2.59E-27 | 0.498269618 | 0.259 | 0.017 | 8.06E-23 | Lymphatic | TC2N |
| GLIPR2 | 5.60E-27 | 0.897879315 | 0.431 | 0.057 | 1.74E-22 | Lymphatic | GLIPR2 |
| REEP1 | 7.34E-25 | 0.476949284 | 0.172 | 0.007 | 2.28E-20 | Lymphatic | REEP1 |
| TMEM100 | 7.35E-25 | 0.524537601 | 0.293 | 0.026 | 2.29E-20 | Lymphatic | TMEM100 |
| PDE1A | 3.14E-24 | 0.337210212 | 0.103 | 0.001 | 9.78E-20 | Lymphatic | PDE1A |
| KIAA1324L | 3.21E-24 | 0.482288387 | 0.224 | 0.014 | 9.99E-20 | Lymphatic | KIAA1324L |
| OVOS2 | 8.71E-24 | 0.860606528 | 0.31 | 0.032 | 2.71E-19 | Lymphatic | OVOS2 |
| PIEZO2 | 3.16E-23 | 0.616540466 | 0.345 | 0.041 | 9.84E-19 | Lymphatic | PIEZO2 |
| FLT41 | 3.47E-23 | 1.35441405 | 0.655 | 0.18 | 1.08E-18 | Lymphatic | FLT4 |
| PTPN3 | 4.17E-23 | 0.657115184 | 0.19 | 0.01 | 1.30E-18 | Lymphatic | PTPN3 |
| TNFAIP8L3 | 4.31E-23 | 0.52063042 | 0.207 | 0.013 | 1.34E-18 | Lymphatic | TNFAIP8L3 |
| SLC38A1 | 4.75E-23 | 1.339363321 | 0.569 | 0.131 | 1.48E-18 | Lymphatic | SLC38A1 |
| FAM102A | 4.90E-23 | 0.607453791 | 0.293 | 0.029 | 1.52E-18 | Lymphatic | FAM102A |
| DMTN | 5.03E-23 | 0.893996442 | 0.466 | 0.083 | 1.57E-18 | Lymphatic | DMTN |
| AZIN2 | 2.01E-22 | 0.407049497 | 0.241 | 0.02 | 6.26E-18 | Lymphatic | AZIN2 |
| RGS17 | 5.89E-22 | 0.345361138 | 0.19 | 0.011 | 1.83E-17 | Lymphatic | RGS17 |
| ITM2C | 4.29E-21 | 0.841324785 | 0.397 | 0.062 | 1.34E-16 | Lymphatic | ITM2C |
| DENND2A | 1.81E-20 | 0.291554436 | 0.121 | 0.003 | 5.65E-16 | Lymphatic | DENND2A |
| BCHE | 3.08E-20 | 0.396431016 | 0.224 | 0.019 | 9.57E-16 | Lymphatic | BCHE |
| CCDC102B | 7.05E-20 | 0.25078719 | 0.155 | 0.008 | 2.19E-15 | Lymphatic | CCDC102B |
| IRF8 | 7.08E-20 | 0.530773458 | 0.259 | 0.026 | 2.20E-15 | Lymphatic | IRF8 |
| MXD3 | 7.57E-20 | 0.407499581 | 0.172 | 0.01 | 2.36E-15 | Lymphatic | MXD3 |
| CGNL1 | 8.17E-20 | 0.478905372 | 0.241 | 0.023 | 2.54E-15 | Lymphatic | CGNL1 |
| GYPC1 | 9.28E-20 | 1.505494393 | 0.845 | 0.409 | 2.89E-15 | Lymphatic | GYPC |
| FLNC | 1.02E-19 | 0.677563547 | 0.362 | 0.054 | 3.17E-15 | Lymphatic | FLNC |
| FCGR2A | 1.39E-19 | 0.309191418 | 0.207 | 0.016 | 4.32E-15 | Lymphatic | FCGR2A |
| KCTD17 | 5.34E-19 | 0.676741538 | 0.483 | 0.103 | 1.66E-14 | Lymphatic | KCTD17 |
| SCN3B | 5.42E-19 | 0.701492506 | 0.276 | 0.033 | 1.69E-14 | Lymphatic | SCN3B |
| CH17-189H20.1 | 5.84E-19 | 0.341677005 | 0.207 | 0.017 | 1.82E-14 | Lymphatic | CH17-189H20.1 |
| TLL2 | 6.47E-19 | 0.328467059 | 0.172 | 0.011 | 2.01E-14 | Lymphatic | TLL2 |
| NUPR1 | 6.93E-19 | 1.24717526 | 0.5 | 0.114 | 2.16E-14 | Lymphatic | NUPR1 |
| CD38 | 1.01E-18 | 0.400233519 | 0.121 | 0.004 | 3.15E-14 | Lymphatic | CD38 |
| FOXC2 | 1.12E-18 | 0.370303957 | 0.121 | 0.004 | 3.48E-14 | Lymphatic | FOXC2 |
| HPSE | 5.72E-18 | 0.337701882 | 0.155 | 0.009 | 1.78E-13 | Lymphatic | HPSE |
| PRKCZ | 9.84E-18 | 0.467033731 | 0.379 | 0.066 | 3.06E-13 | Lymphatic | PRKCZ |
| CMBL | 1.06E-17 | 0.349973721 | 0.224 | 0.022 | 3.30E-13 | Lymphatic | CMBL |
| FAM107B | 1.43E-17 | 1.023180754 | 0.466 | 0.105 | 4.46E-13 | Lymphatic | FAM107B |
| PPFIBP1 | 1.44E-17 | 1.570076763 | 0.776 | 0.382 | 4.48E-13 | Lymphatic | PPFIBP1 |
| ROGDI | 1.96E-17 | 0.609751653 | 0.379 | 0.07 | 6.09E-13 | Lymphatic | ROGDI |
| CCDC80 | 2.47E-17 | 0.924385603 | 0.466 | 0.108 | 7.69E-13 | Lymphatic | CCDC80 |
| RP11-322E11.5 | 2.68E-17 | 0.701289139 | 0.379 | 0.069 | 8.33E-13 | Lymphatic | RP11-322E11.5 |
| FLRT2 | 9.88E-17 | 0.646276498 | 0.362 | 0.065 | 3.08E-12 | Lymphatic | FLRT2 |
| PLEKHG3 | 9.98E-17 | 0.269658942 | 0.103 | 0.003 | 3.11E-12 | Lymphatic | PLEKHG3 |
| CEMIP | 1.73E-16 | 1.120640613 | 0.172 | 0.014 | 5.37E-12 | Lymphatic | CEMIP |
| NINL | 2.05E-16 | 0.413869504 | 0.241 | 0.029 | 6.38E-12 | Lymphatic | NINL |
| P2RX4 | 2.34E-16 | 0.529422748 | 0.241 | 0.029 | 7.27E-12 | Lymphatic | P2RX4 |
| TIMP31 | 3.15E-16 | 1.379291356 | 0.897 | 0.486 | 9.79E-12 | Lymphatic | TIMP3 |
| SYNM | 3.28E-16 | 0.821852808 | 0.345 | 0.063 | 1.02E-11 | Lymphatic | SYNM |
| SMYD2 | 4.25E-16 | 0.754118231 | 0.448 | 0.108 | 1.32E-11 | Lymphatic | SMYD2 |
| NFIX | 5.99E-16 | 0.485542677 | 0.259 | 0.034 | 1.86E-11 | Lymphatic | NFIX |
| FN1 | 1.73E-15 | 1.24655864 | 0.879 | 0.485 | 5.37E-11 | Lymphatic | FN1 |
| ATP5E | 2.56E-15 | 0.756908613 | 1 | 0.971 | 7.97E-11 | Lymphatic | ATP5E |
| MFAP2 | 3.10E-15 | 0.884401314 | 0.517 | 0.148 | 9.64E-11 | Lymphatic | MFAP2 |
| SCN9A | 3.35E-15 | 0.263659047 | 0.155 | 0.012 | 1.04E-10 | Lymphatic | SCN9A |
| THSD4 | 6.03E-15 | 0.459037831 | 0.224 | 0.027 | 1.88E-10 | Lymphatic | THSD4 |
| LINC01558 | 6.41E-15 | 0.576592344 | 0.276 | 0.043 | 2.00E-10 | Lymphatic | LINC01558 |
| SPHK1 | 1.07E-14 | 1.10636071 | 0.69 | 0.287 | 3.32E-10 | Lymphatic | SPHK1 |
| DSP | 1.08E-14 | 0.545395507 | 0.379 | 0.078 | 3.36E-10 | Lymphatic | DSP |
| PLSCR4 | 1.14E-14 | 1.078582093 | 0.414 | 0.095 | 3.55E-10 | Lymphatic | PLSCR4 |
| SLC45A3 | 1.29E-14 | 0.392910283 | 0.172 | 0.016 | 4.03E-10 | Lymphatic | SLC45A3 |
| SEMA6A | 1.63E-14 | 0.886546504 | 0.569 | 0.175 | 5.06E-10 | Lymphatic | SEMA6A |
| FABP5 | 4.55E-14 | 1.48495786 | 0.897 | 0.646 | 1.41E-09 | Lymphatic | FABP5 |
| SYT1 | 7.71E-14 | 0.401256146 | 0.172 | 0.017 | 2.40E-09 | Lymphatic | SYT1 |
| MAP4K2 | 7.80E-14 | 0.542471558 | 0.31 | 0.059 | 2.43E-09 | Lymphatic | MAP4K2 |
| TIMP1 | 1.00E-13 | 2.652028827 | 0.931 | 0.867 | 3.11E-09 | Lymphatic | TIMP1 |
| RCSD1 | 1.28E-13 | 0.370870101 | 0.241 | 0.036 | 3.98E-09 | Lymphatic | RCSD1 |
| SDPR | 1.61E-13 | 1.452302223 | 0.828 | 0.472 | 5.01E-09 | Lymphatic | SDPR |
| WFS1 | 2.24E-13 | 0.667692561 | 0.345 | 0.074 | 6.96E-09 | Lymphatic | WFS1 |
| MDK | 3.22E-13 | 1.362870939 | 0.724 | 0.358 | 1.00E-08 | Lymphatic | MDK |
| FABP41 | 5.37E-13 | 1.922634868 | 0.466 | 0.133 | 1.67E-08 | Lymphatic | FABP4 |
| COL9A3 | 7.38E-13 | 0.400214522 | 0.19 | 0.023 | 2.30E-08 | Lymphatic | COL9A3 |
| ARSJ | 9.17E-13 | 0.268115282 | 0.121 | 0.009 | 2.85E-08 | Lymphatic | ARSJ |
| NR1H3 | 1.15E-12 | 0.608149754 | 0.172 | 0.02 | 3.57E-08 | Lymphatic | NR1H3 |
| SLC24A1 | 1.82E-12 | 0.547881347 | 0.31 | 0.066 | 5.67E-08 | Lymphatic | SLC24A1 |
| MYH10 | 1.84E-12 | 0.750382234 | 0.414 | 0.113 | 5.74E-08 | Lymphatic | MYH10 |
| SSBP2 | 2.59E-12 | 0.324301585 | 0.276 | 0.049 | 8.06E-08 | Lymphatic | SSBP2 |
| CNN3 | 3.68E-12 | 1.203828897 | 0.862 | 0.646 | 1.15E-07 | Lymphatic | CNN3 |
| LAYN | 4.03E-12 | 0.813134631 | 0.552 | 0.209 | 1.25E-07 | Lymphatic | LAYN |
| EMID1 | 4.56E-12 | 0.413240444 | 0.19 | 0.025 | 1.42E-07 | Lymphatic | EMID1 |
| MBP | 5.15E-12 | 0.263902335 | 0.121 | 0.009 | 1.60E-07 | Lymphatic | MBP |
| SERPING1 | 5.89E-12 | 1.017022852 | 0.638 | 0.262 | 1.83E-07 | Lymphatic | SERPING1 |
| MAN1A1 | 6.90E-12 | 0.905333319 | 0.569 | 0.221 | 2.15E-07 | Lymphatic | MAN1A1 |
| HSPB1 | 7.12E-12 | 1.062968818 | 0.983 | 0.951 | 2.21E-07 | Lymphatic | HSPB1 |
| CYBA1 | 1.10E-11 | 1.119391759 | 0.845 | 0.574 | 3.42E-07 | Lymphatic | CYBA |
| WDFY2 | 1.20E-11 | 0.413861073 | 0.276 | 0.054 | 3.74E-07 | Lymphatic | WDFY2 |
| CISD1 | 1.22E-11 | 1.152286616 | 0.707 | 0.406 | 3.78E-07 | Lymphatic | CISD1 |
| RARRES3 | 1.22E-11 | 1.081337117 | 0.5 | 0.166 | 3.78E-07 | Lymphatic | RARRES3 |
| FXYD6 | 1.72E-11 | 1.032691423 | 0.466 | 0.156 | 5.36E-07 | Lymphatic | FXYD6 |
| EDNRB1 | 1.76E-11 | 0.546466893 | 0.345 | 0.079 | 5.47E-07 | Lymphatic | EDNRB |
| LIMS1 | 1.97E-11 | 1.046872637 | 0.828 | 0.554 | 6.14E-07 | Lymphatic | LIMS1 |
| SGIP1 | 2.00E-11 | 0.685240048 | 0.345 | 0.086 | 6.23E-07 | Lymphatic | SGIP1 |
| C16orf62 | 4.29E-11 | 0.880172241 | 0.466 | 0.158 | 1.33E-06 | Lymphatic | C16orf62 |
| EPB41L2 | 4.53E-11 | 0.779163508 | 0.483 | 0.175 | 1.41E-06 | Lymphatic | EPB41L2 |
| RARRES2 | 5.35E-11 | 0.903975235 | 0.379 | 0.106 | 1.66E-06 | Lymphatic | RARRES2 |
| KIAA1456 | 7.06E-11 | 0.261556457 | 0.138 | 0.014 | 2.20E-06 | Lymphatic | KIAA1456 |
| SLC7A7 | 7.46E-11 | 0.32929973 | 0.19 | 0.028 | 2.32E-06 | Lymphatic | SLC7A7 |
| SEPP1 | 1.28E-10 | 1.10307977 | 0.69 | 0.367 | 3.99E-06 | Lymphatic | SEPP1 |
| TSTA3 | 1.30E-10 | 0.887822224 | 0.603 | 0.283 | 4.05E-06 | Lymphatic | TSTA3 |
| NRP21 | 1.57E-10 | 1.265383723 | 0.724 | 0.427 | 4.89E-06 | Lymphatic | NRP2 |
| PGM5 | 3.24E-10 | 0.654253335 | 0.448 | 0.15 | 1.01E-05 | Lymphatic | PGM5 |
| KLHL4 | 4.39E-10 | 0.332011495 | 0.155 | 0.02 | 1.37E-05 | Lymphatic | KLHL4 |
| AKR1C3 | 6.37E-10 | 0.780607824 | 0.517 | 0.203 | 1.98E-05 | Lymphatic | AKR1C3 |
| THEMIS2 | 6.76E-10 | 0.433035662 | 0.259 | 0.057 | 2.10E-05 | Lymphatic | THEMIS2 |
| ARL6IP1 | 7.77E-10 | 1.112260294 | 0.776 | 0.517 | 2.42E-05 | Lymphatic | ARL6IP1 |
| RPL13A1 | 1.34E-09 | 0.451818619 | 1 | 0.994 | 4.17E-05 | Lymphatic | RPL13A |
| NR2F21 | 1.34E-09 | 0.923708748 | 0.69 | 0.363 | 4.17E-05 | Lymphatic | NR2F2 |
| ITGA91 | 1.59E-09 | 0.645021971 | 0.397 | 0.129 | 4.95E-05 | Lymphatic | ITGA9 |
| GPAT3 | 1.88E-09 | 0.297971806 | 0.103 | 0.009 | 5.84E-05 | Lymphatic | GPAT3 |
| ELN1 | 2.04E-09 | 0.603354873 | 0.362 | 0.106 | 6.36E-05 | Lymphatic | ELN |
| RP11-14N7.2 | 3.13E-09 | 0.569252453 | 0.414 | 0.145 | 9.75E-05 | Lymphatic | RP11-14N7.2 |
| TSPAN5 | 3.40E-09 | 0.759816562 | 0.31 | 0.087 | 0.000105753 | Lymphatic | TSPAN5 |
| CASK | 3.64E-09 | 0.488134461 | 0.293 | 0.076 | 0.000113172 | Lymphatic | CASK |
| SDC4 | 3.65E-09 | 0.747926754 | 0.293 | 0.078 | 0.000113473 | Lymphatic | SDC4 |
| DLG1 | 3.74E-09 | 0.814100973 | 0.448 | 0.166 | 0.000116292 | Lymphatic | DLG1 |
| MARCKS1 | 4.03E-09 | 0.800056161 | 0.862 | 0.662 | 0.000125366 | Lymphatic | MARCKS |
| CD47 | 4.29E-09 | 0.825478832 | 0.638 | 0.339 | 0.000133639 | Lymphatic | CD47 |
| MEDAG | 4.32E-09 | 0.454165129 | 0.31 | 0.078 | 0.000134296 | Lymphatic | MEDAG |
| TMEM140 | 4.81E-09 | 0.569231836 | 0.379 | 0.119 | 0.000149784 | Lymphatic | TMEM140 |
| SNCA | 4.84E-09 | 0.581313496 | 0.379 | 0.119 | 0.000150656 | Lymphatic | SNCA |
| LRP5 | 5.96E-09 | 0.522997063 | 0.293 | 0.077 | 0.000185471 | Lymphatic | LRP5 |
| MICAL2 | 6.19E-09 | 0.423490949 | 0.293 | 0.078 | 0.00019251 | Lymphatic | MICAL2 |
| GLT8D2 | 6.22E-09 | 0.347082511 | 0.276 | 0.067 | 0.000193606 | Lymphatic | GLT8D2 |
| SIRPA | 6.58E-09 | 0.487048513 | 0.345 | 0.1 | 0.00020473 | Lymphatic | SIRPA |
| SGCE | 7.00E-09 | 0.313835749 | 0.241 | 0.054 | 0.000217698 | Lymphatic | SGCE |
| RAB11A | 7.00E-09 | 0.663165419 | 0.897 | 0.759 | 0.0002179 | Lymphatic | RAB11A |
| CHCHD10 | 7.46E-09 | 0.678930799 | 0.603 | 0.277 | 0.000232069 | Lymphatic | CHCHD10 |
| PPP1R2 | 8.99E-09 | 0.898126772 | 0.707 | 0.431 | 0.000279749 | Lymphatic | PPP1R2 |
| EFCC1 | 1.01E-08 | 0.350525374 | 0.155 | 0.024 | 0.000312915 | Lymphatic | EFCC1 |
| FEZ1 | 1.07E-08 | 0.297772971 | 0.259 | 0.062 | 0.000332702 | Lymphatic | FEZ1 |
| LY96 | 1.10E-08 | 0.80545902 | 0.707 | 0.391 | 0.000343582 | Lymphatic | LY96 |
| TLE1 | 1.40E-08 | 0.466180767 | 0.362 | 0.111 | 0.000436146 | Lymphatic | TLE1 |
| GRAP1 | 1.42E-08 | 0.482403021 | 0.431 | 0.155 | 0.000442621 | Lymphatic | GRAP |
| DTX4 | 1.96E-08 | 0.261390489 | 0.103 | 0.011 | 0.000610991 | Lymphatic | DTX4 |
| SPA17 | 2.00E-08 | 0.279816369 | 0.172 | 0.031 | 0.000623572 | Lymphatic | SPA17 |
| GGA1 | 2.01E-08 | 0.712196941 | 0.483 | 0.203 | 0.000624495 | Lymphatic | GGA1 |
| NFIA-AS2 | 2.31E-08 | 0.276144546 | 0.121 | 0.015 | 0.000717932 | Lymphatic | NFIA-AS2 |
| CDC25B | 2.38E-08 | 0.655875276 | 0.431 | 0.162 | 0.000741773 | Lymphatic | CDC25B |
| BMP2K | 2.43E-08 | 0.576017597 | 0.328 | 0.102 | 0.000754769 | Lymphatic | BMP2K |
| C17orf67 | 2.55E-08 | 0.509240349 | 0.259 | 0.066 | 0.000793629 | Lymphatic | C17orf67 |
| FRMD6 | 2.65E-08 | 0.433678077 | 0.276 | 0.074 | 0.000825758 | Lymphatic | FRMD6 |
| VLDLR | 3.00E-08 | 0.266723393 | 0.138 | 0.02 | 0.000932211 | Lymphatic | VLDLR |
| KLHL3 | 3.38E-08 | 0.444256104 | 0.293 | 0.082 | 0.001050169 | Lymphatic | KLHL3 |
| CTDSPL | 3.64E-08 | 0.380780529 | 0.259 | 0.066 | 0.001131882 | Lymphatic | CTDSPL |
| SHC1 | 4.05E-08 | 0.858538121 | 0.776 | 0.493 | 0.001260319 | Lymphatic | SHC1 |
| ALDH1A1 | 4.64E-08 | 0.879441953 | 0.379 | 0.139 | 0.001442836 | Lymphatic | ALDH1A1 |
| NNMT1 | 4.64E-08 | 1.039483575 | 0.845 | 0.655 | 0.001444831 | Lymphatic | NNMT |
| LYN | 4.88E-08 | 0.733388869 | 0.517 | 0.233 | 0.001519353 | Lymphatic | LYN |
| UNC5B2 | 4.98E-08 | 0.522548153 | 0.534 | 0.218 | 0.001549301 | Lymphatic | UNC5B |
| STMN11 | 5.13E-08 | 0.545033805 | 0.828 | 0.542 | 0.001594789 | Lymphatic | STMN1 |
| PTPRE | 5.72E-08 | 1.234290787 | 0.724 | 0.433 | 0.001780669 | Lymphatic | PTPRE |
| NINJ1 | 5.80E-08 | 0.813790802 | 0.586 | 0.301 | 0.001805612 | Lymphatic | NINJ1 |
| TMEM123 | 6.39E-08 | 0.677187 | 0.741 | 0.471 | 0.001986952 | Lymphatic | TMEM123 |
| SMAD1 | 7.59E-08 | 0.791188246 | 0.621 | 0.322 | 0.002360952 | Lymphatic | SMAD1 |
| PHPT1 | 9.15E-08 | 0.788145008 | 0.776 | 0.622 | 0.002848434 | Lymphatic | PHPT1 |
| MYL6B | 9.43E-08 | 0.622360388 | 0.586 | 0.296 | 0.002934905 | Lymphatic | MYL6B |
| ENOSF1 | 1.06E-07 | 0.433457201 | 0.345 | 0.113 | 0.003288788 | Lymphatic | ENOSF1 |
| POLR2L | 1.13E-07 | 0.621930701 | 0.931 | 0.858 | 0.003502863 | Lymphatic | POLR2L |
| ATP9A | 1.15E-07 | 0.277640963 | 0.172 | 0.033 | 0.003583716 | Lymphatic | ATP9A |
| LOX1 | 1.18E-07 | 0.551546487 | 0.345 | 0.113 | 0.003665739 | Lymphatic | LOX |
| PCBD1 | 1.69E-07 | 0.623673501 | 0.517 | 0.252 | 0.005260041 | Lymphatic | PCBD1 |
| ABHD17B | 1.80E-07 | 0.485171256 | 0.293 | 0.089 | 0.005603002 | Lymphatic | ABHD17B |
| RAB3IL1 | 1.87E-07 | 0.253525139 | 0.103 | 0.013 | 0.005803386 | Lymphatic | RAB3IL1 |
| INO80C | 1.89E-07 | 0.635960656 | 0.534 | 0.26 | 0.005868342 | Lymphatic | INO80C |
| PALM | 2.07E-07 | 0.658876864 | 0.328 | 0.11 | 0.006431357 | Lymphatic | PALM |
| LTBP41 | 3.19E-07 | 0.482657512 | 0.345 | 0.12 | 0.009933501 | Lymphatic | LTBP4 |
| FHIT | 3.69E-07 | 0.262435135 | 0.155 | 0.03 | 0.011485009 | Lymphatic | FHIT |
| REXO2 | 3.83E-07 | 0.727518449 | 0.741 | 0.566 | 0.011920428 | Lymphatic | REXO2 |
| RP11-798M19.6 | 5.98E-07 | 0.460641507 | 0.31 | 0.1 | 0.018610654 | Lymphatic | RP11-798M19.6 |
| CEACAM1 | 6.53E-07 | 0.343134969 | 0.19 | 0.044 | 0.020322643 | Lymphatic | CEACAM1 |
| STOX2 | 6.87E-07 | 0.559160206 | 0.241 | 0.069 | 0.021364195 | Lymphatic | STOX2 |
| TMEM38B | 7.96E-07 | 0.262110385 | 0.172 | 0.037 | 0.024758018 | Lymphatic | TMEM38B |
| SDC3 | 8.62E-07 | 0.35614066 | 0.293 | 0.093 | 0.026820118 | Lymphatic | SDC3 |
| TMEM246 | 9.22E-07 | 0.404456663 | 0.276 | 0.086 | 0.028696319 | Lymphatic | TMEM246 |
| APOD1 | 9.25E-07 | 0.312218038 | 0.259 | 0.074 | 0.028788587 | Lymphatic | APOD |
| HOMER31 | 1.01E-06 | 0.634795467 | 0.431 | 0.196 | 0.031461949 | Lymphatic | HOMER3 |
| MIF | 1.13E-06 | 0.641139678 | 0.862 | 0.668 | 0.035036952 | Lymphatic | MIF |
| ASGR1 | 1.18E-06 | 0.464115427 | 0.293 | 0.099 | 0.036634799 | Lymphatic | ASGR1 |
